# Entry to 2-aminoprolines via electrochemical decarboxylative amidation of *N*‑acetylamino malonic acid monoesters

**DOI:** 10.3762/bjoc.21.50

**Published:** 2025-03-19

**Authors:** Olesja Koleda, Janis Sadauskis, Darja Antonenko, Edvards Janis Treijs, Raivis Davis Steberis, Edgars Suna

**Affiliations:** 1 Latvian Institute of Organic Synthesis, Aizkraukles 21, Riga LV-1006, Latvia; 2 Faculty of Medicine and Life Sciences, Department of Chemistry, University of Latvia, Jelgavas 1, Riga LV-1004, Latvia

**Keywords:** anodic oxidation, decarboxylation, electrosynthesis, Hofer–Moest reaction, non-proteinogenic amino acids

## Abstract

The electrochemical synthesis of 2-aminoprolines based on anodic decarboxylation–intramolecular amidation of readily available *N*-acetylamino malonic acid monoesters is reported. The decarboxylative amidation under Hofer–Moest reaction conditions proceeds in an undivided cell under constant current conditions in aqueous acetonitrile and provides access to *N*-sulfonyl, *N*-benzoyl, and *N*-Boc-protected 2-aminoproline derivatives.

## Introduction

**Figure 1:**
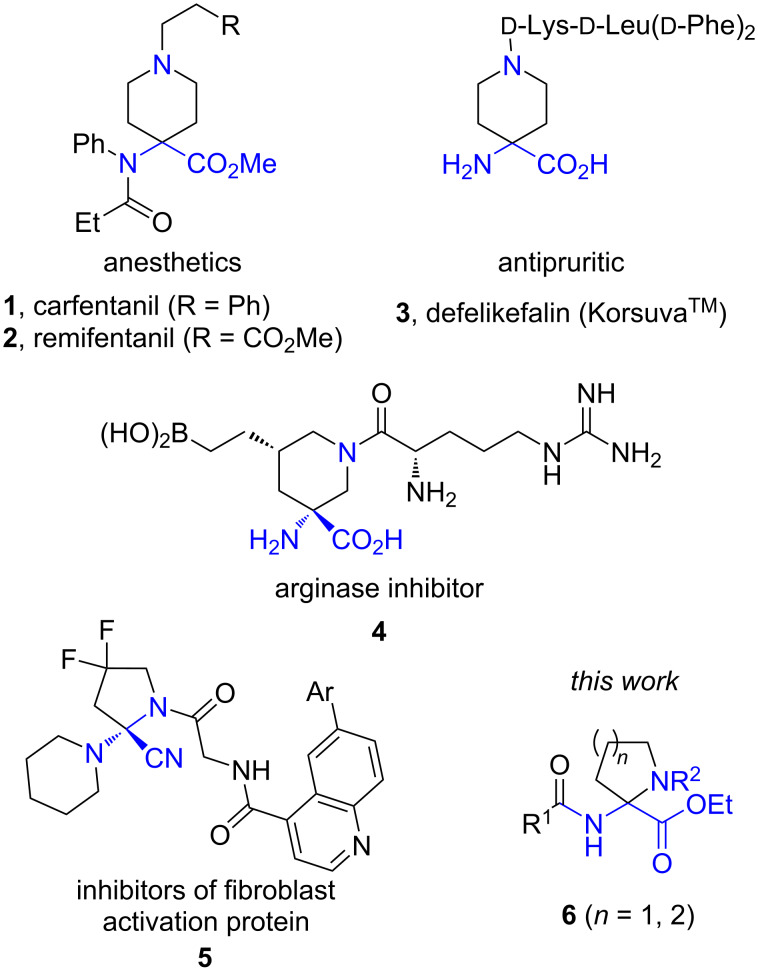
Selected examples of α,α-disubstituted cyclic amino acids in drug design.

Non-proteinogenic cyclic amino acids are common structural motifs in the design of small-molecule drugs and peptidomimetics [1]. For example, the clinically used anesthetics carfentanil (**1**) and remifentanil (**2**), the FDA-approved antipruritic medication defelikefalin (**3**), and the arginase inhibitor **4** [2] possess cyclic α,α-disubstituted piperidine-containing amino acid subunits. Likewise, a cyano-substituted cyclic aminal is a core structural unit of the fibroblast activation protein inhibitor **5** [3] (Figure 1). The widespread use of non-proteinogenic cyclic amino acids in drug discovery justifies both the design of new analogs and the development of efficient synthetic methods to access these medicinally relevant structural motifs. Herein, we report an electrochemical synthesis of 2-aminoproline and 2-aminopipecolic acid derivatives **6** (Figure 1).

**Figure 2:**
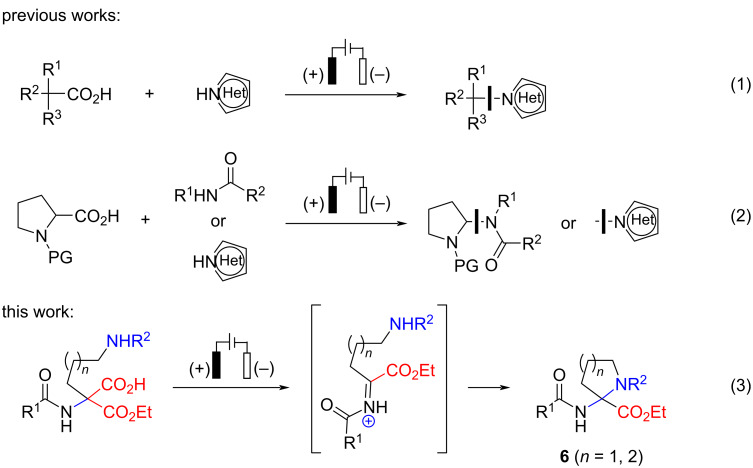
Electrochemical decarboxylative amination reactions.

Recently, we disclosed an electrochemical approach to tetrahydrofuran and tetrahydropyran-containing amino acid derivatives via anodic decarboxylation of *N*-acetylamino malonic acid monoesters to generate a stabilized carbocation (Hofer–Moest conditions), which were then reacted with a tethered oxygen nucleophile [4]. In this follow-up study, we demonstrate that *N*-protected amines are also suitable as nucleophiles for the cyclization into 2-aminoproline and 2-aminopipecolic acid derivatives **6** (Figure 2, reaction 3). The starting disubstituted malonic esters are readily available by *C*-alkylation of inexpensive and readily available diethyl acetamidomalonate, followed by monohydrolysis under basic conditions. The electrolysis proceeds in an undivided cell under galvanostatic control using low-cost graphite or stainless-steel electrodes, and the protocol was easily upscaled. Notably, an excellent diastereoselectivity (97:3 dr) could be achieved in the cyclization of a tethered chiral nitrogen nucleophile as shown below. To the best of our knowledge, the electrosynthesis of *gem*-α,α-diamino acid derivatives **6** has not been accomplished, and all published electrochemical amination examples under Hofer–Moest conditions [5] targeted either *N*-substituted heteroarenes [6] or aminals [7–8] (Figure 2, reactions 1 and 2, respectively).

## Results and Discussion

**Scheme 1:**
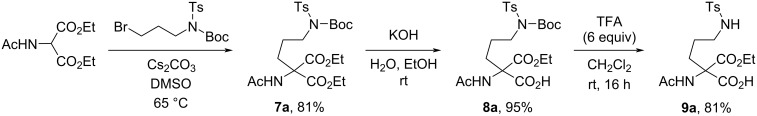
Preparation of malonic acid monoester **9a**.

*N*-Acetylamino malonic acid monoester **9a** possessing a tosyl-protected tethered amine was selected as a model substrate for the development of the intramolecular amidation under Hofer–Moest conditions. The acid **9a** was prepared in three steps (62% overall yield) from commercially available diethyl acetamidomalonate by an alkylation/hydrolysis/Boc-cleavage sequence (Scheme 1).

The development of decarboxylative amidation commenced by examining the published conditions for anodic decarboxylation/etherification [4]. Accordingly, the electrolysis of monoester **9a** in a 2:1 MeCN/H_2_O mixture in the presence of 0.025 M LiClO_4_ solution under constant current conditions (*j* = 12 mA/cm^2^) with graphite both as an anode and a cathode material afforded the desired *N*-tosylpyrrolidine **6a** in 67% yield (Table 1, entry 1). The water quench of a transient *N*-acyliminium species was found to be a major side-reaction as evidenced by the formation of an open-chain hemiaminal **10a** (the hemiaminal could not be isolated due to the instability on silica gel). Screening of other supporting electrolytes revealed that basic salts (K_2_CO_3_, Na_2_CO_3_, NaOAc) did not improve the efficiency of the anodic decarboxylation/cyclization reaction (Table 1, entries 2–4). Even though the amount of hemiaminal **10a** was slightly reduced, the formation of amino acid ester **11a** as side product was observed in the crude reaction mixture (Table 1, entries 2–4). The latter could be suppressed completely by using non-basic anion-containing tetraalkylammonium salts as the supporting electrolytes (Table 1, entries 5–7) with Et_4_N–BF_4_ providing the highest yield of the desired product **6a**. The anodic decarboxylation/cyclization reaction was similarly efficient when the amount of water was reduced from 33% to 17% (Table 1, entry 8 vs entry 7), an observation that might be useful for substrates of low aqueous solubility. However, further reduction of water amount to 5 equivalents completely inhibited the anodic oxidation of **9a**, and only traces of the desired **6a** were observed (see Supporting Information File 1, page S3). Decrease in supporting electrolyte concentration led to a drop in yields (Table 1, entry 9 vs entry 8), whereas current density deviations from 12 mA/cm^2^ did not affect the outcome of **6a** (see Supporting Information File 1, page S4). Interestingly, replacement of graphite with stainless steel (SS) [9] as the cathode material afforded similar yields of the desired heterocycle **6a** (72% and 70%, respectively; Table 1, entries 8 and 10), so both graphite and SS were subsequently used in the scope studies (vide infra). Other cathode materials such as Pt or BDD (boron-doped diamond) delivered **6a** in reduced yields (Table 1, entries 11 and 12). Finally, brief examination of passed charge returned 2.0 F as the optimal amount. The amount of charge could be increased to 2.5 F in case of incomplete conversion of the starting **6a**, however, further rise above 2.5 F led to a drop in the pyrrolidine **6a** yield due to the formation of a new side-product.

**Table 1:** Optimization of anodic decarboxylation/amidation reaction.

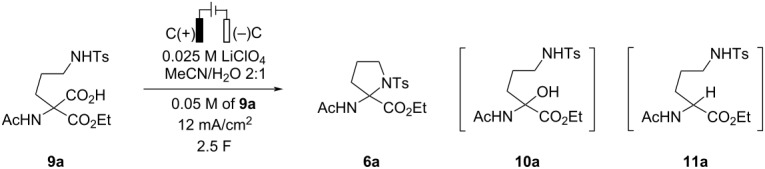			

Entry	Deviations from the starting conditions	Yield, %^a^	**6a**:**10a**:**11a**^b^

1	none	67	84:16:0
2	K_2_CO_3_, 2.0 F	54	86:3:11
3	Na_2_CO_3_, 2.0 F	54	86:4:10
4	NaOAc, 2.0 F	56	71:13:16
5	Bu_4_N–ClO_4_, 2.3 F	67	85:15:0
6	Et_4_N–PF_6_	66	85:15:0
7	Et_4_N–BF_4_	71	84:16:0
**8**	**Et** **_4_** **N–BF** **_4_** **, 5:1 MeCN/H** **_2_** **O**	**72**	**86:13:1**
9	0.05 M Et_4_N–BF_4_, 5:1 MeCN/H_2_O	67	85:13:2
**10**	**Et** **_4_** **N–BF** **_4_** **, 5:1 MeCN/H** **_2_** **O, 2.0 F, SS (−)**	**70**	**87:13:0**
11	Et_4_N–BF_4_, 5:1 MeCN/H_2_O, 2.0 F, Pt (−)	63	84:16:0
12	Et_4_N–BF_4_, 5:1 MeCN/H_2_O, 2.8 F, BDD(−)	62	86:12:2

^a^Yields were determined by ^1^H NMR post-electrolysis using CH_2_Br_2_ as an internal standard. The reactions were performed on a 0.15 mmol scale. ^b^Ratios determined by LC–MS (UV detection).

**Figure 3:**
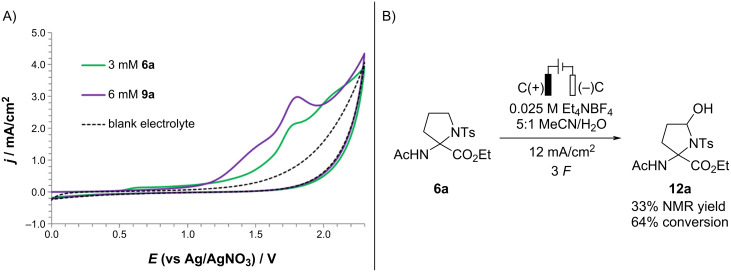
A) Cyclic voltammograms of **6a** and **9a** at 3 mM and 6 mM concentration, respectively, in 5:1 MeCN/H_2_O (0.1 M Et_4_N–BF_4_). B) Anodic oxidation of pyrrolidine **6a**.

We hypothesized that the side-product formation at increased amounts (>2.5 F) of passed charge results from undesired Shono oxidation of pyrrolidine **6a** [10–11]. Indeed, CV studies of **6a** revealed an irreversible feature at *E*_p_ = 1.78 V vs Ag/Ag^+^ (100 mV/s scan rate; see Figure 3A), and the electrolysis of pyrrolidine **6a** under the optimized anodic decarboxylative cyclization conditions (entry 8, Table 1) afforded cyclic hemiaminal **12a (**33% NMR yield), whose structure was proved by NMR experiments (Figure 3B). The relatively narrow potential window of 0.22 V between the desired decarboxylation of **9a** (*E*_p_ = 1.56 V vs Ag/Ag^+^) and the undesired Shono-type oxidation of the formed **6a** required careful control of the amount of passed charge to afford high yields of **6a**.

**Scheme 2:**
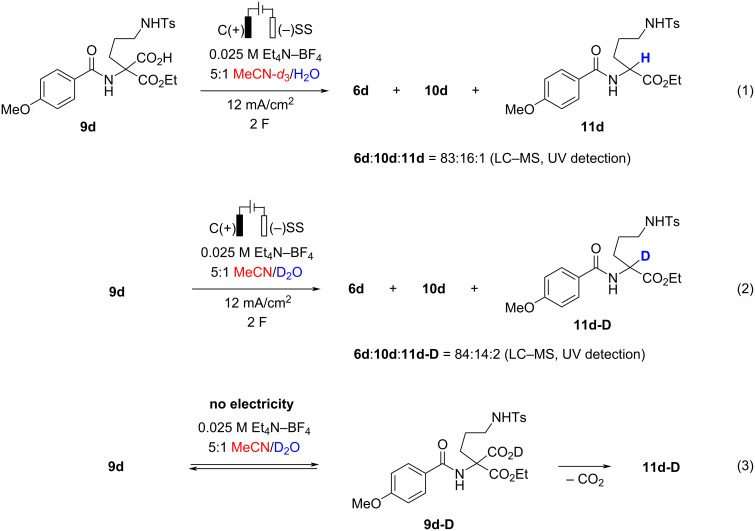
Electrolysis of acid **9d** in deuterated solvents.

Next, the formation of decarboxylation product **11a** was addressed. Initially, we hypothesized that **11a** may form by a single-electron oxidation/decarboxylation (Kolbe reaction) of **9a** to generate carbon-centered radical, followed by hydrogen abstraction from solvent. To verify the hypothesis, an electrolysis of acid **9d** was performed under optimized conditions (entry 10, Table 1) in deuterated solvents (Scheme 2; for details, see Supporting Information File 1, page S40). Surprisingly, the electrolysis in a 5:1 mixture of MeCN-*d_3_* and water delivered **11d** without deuterium incorporation (Scheme 2, reaction 1). In contrast, the formation of deuterated **11d-D** was observed by LC–MS when the electrolysis was performed in 5:1 MeCN/D_2_O (Scheme 2, reaction 2). The considerably higher O–H bond dissociation energy (119 kcal/mol) [12] as compared to that of the C–H bond in MeCN (86 kcal/mol) [13] renders the hydrogen atom abstraction from water by a carbon-centered radical a very unlikely mechanistic scenario. In the meantime, slow formation of **11d**-**D** was observed upon stirring of **9d** in the 5:1 MeCN/D_2_O mixture even without applying electric charge (Scheme 2, reaction 3). Apparently, **11d** was formed upon spontaneous loss of CO_2_ from equilibrating deuterated carboxylate **9d-D**. Furthermore, monoesters **9** are also prone to spontaneous decarboxylation upon storage. Therefore, freshly prepared material should be used in the electrolysis.

**Figure 4:**
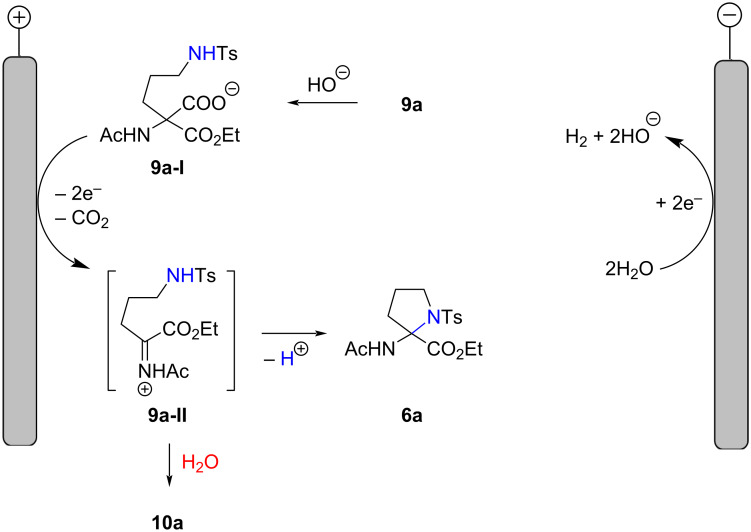
Plausible mechanism for formation of pyrrolidine **6a** and hemiaminal **10a**.

Based on experimental evidence, a working mechanism for the formation of 2-aminoproline **6a** is proposed (Figure 4). Accordingly, an initial deprotonation of carboxylic acid **9a** by cathodically generated hydroxide is followed by anodic oxidation/decarboxylation of the formed carboxylate **9a-I** to generate stabilized cation **9a-II**. The latter undergoes intramolecular cyclization with the tethered *N*-nucleophile into cyclic aminal **6a**. In a competing reaction, the cation **9a-II** reacts with water to form acyclic hemiaminal **10a**.

**Scheme 3:**
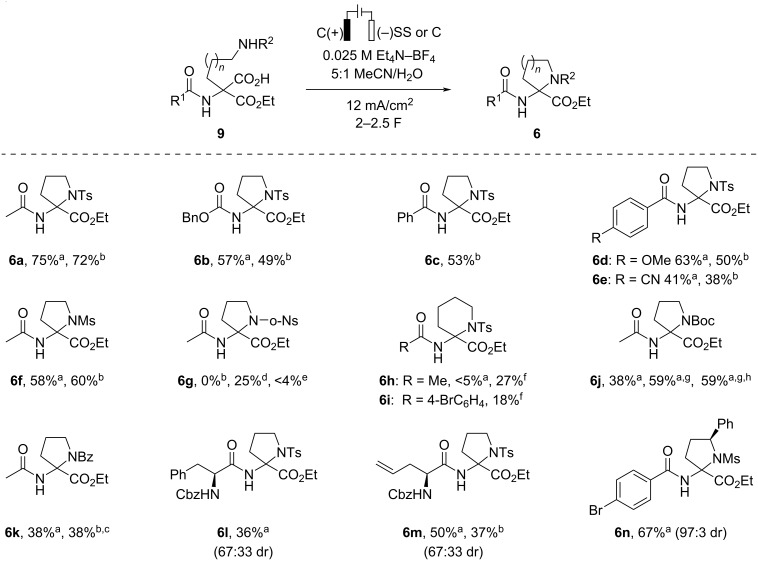
Scope of the decarboxylative amidation. ^a^Stainless-steel cathode; ^b^graphite cathode; ^c^yield determined by ^1^H NMR using CH_2_Br_2_ as an internal standard; ^d^electrolysis in divided cell with NaOH (1 equiv); ^e^Pt cathode; ^f^with KOH (1 equiv); ^g^with KOH (0.5 equiv); ^h^performed on 925 mg (2.7 mmol) scale.

With the optimized conditions in hand (Table 1, entries 8 and 10) the scope of the developed decarboxylative amidation was briefly explored (Scheme 3). *N*-Acetyl, *N*-Cbz, and *N*-Bz protecting groups are compatible with the decarboxylation/cyclization conditions, and the respective 2-aminoproline derivatives **6a**–**c** were obtained in 49–75% yield. Redox-sensitive 4-anisoyl and 4-cyanobenzoyl groups-containing monoesters **9d**,**e** are also suitable as substrates as evidenced by the formation of **6d**,**e** in 38–63% yields. Not only *N*-tosylates undergo the decarboxylative cyclization, but also *N*-mesyl-protected monoester **9f** could be converted into 2-aminoproline derivative **6f** in 60% yield using a graphite cathode. However, the *N*-*o*-nosyl-protecting group is not compatible with the developed electrolysis conditions, likely because it undergoes an undesired cathodic reduction. Indeed, trace amounts of 2-aminoproline derivative **6g** (<4%) could be obtained by replacing SS as the cathode material with platinum that has a low overpotential for hydrogen evolution reaction [14]. To avoid the undesired cathodic reduction of the nitro group, the electrolysis of *N*-*o*-nosyl-protected monoester **9g** was performed in a divided cell in the presence of NaOH as a base (1 equiv). Gratifyingly, by this route *N*-*o*-nosyl-protected **6g** was obtained in 25% yield.

The attempted synthesis of 2-aminopipecolic acid derivative **6h** under the developed conditions was unsuccessful, and afforded trace amounts of **6h** together with the corresponding acyclic hemiaminal **10h** as the major product. Such an outcome can be attributed to a slower formation of a 6-membered ring [15] from transient *N*-acyliminium species. Gratifyingly, the addition of KOH (1 equiv) to the electrolysis mixture facilitated the cyclization, and the 6-membered heterocycles **6h**,**i** could be obtained in 27% and 18% yield, respectively.

In addition to sulfonamides, carbamates such as *N*-Boc and benzamide are also suitable as nucleophiles for the anodic decarboxylation/cyclization reaction. However, the corresponding 2-aminoproline derivatives **6j**,**k** were obtained in considerably lower yields (38%) as compared to those of *N*-Ts analog **6a**. Surprisingly, the addition of KOH (0.5 equiv) to the electrolysis solution has helped to improve yield of *N*-Boc-protected 2-aminoproline derivative **6j** from 38% to 59%. However, the addition of KOH was not always beneficial. For instance, the anodic oxidation of benzamide **9k** in the presence of KOH afforded pyrrolidine **6k** only as a minor product and a mixture of **6k**/**10k**/**11k** in 15:32:53 ratio, respectively, was formed. Finally, the loading of **9j** was increased from 0.3 to 2.7 mmol to demonstrate the scalability of the method, and 470 mg of 2-aminoproline derivative **6j** was obtained in a single electrolysis batch.

The wide application of unnatural amino acids in the design of peptidomimetics prompted us to examine the suitability of the developed conditions for dipeptide synthesis. Gratifyingly, the cyclization of the amino acid fragment-containing monoesters **9l**,**m** afforded dipeptides **6l**,**m** in 36% and 50% yield, respectively. Notably, the decarboxylative cyclization is compatible with the alkene moiety (product **6m**). Both dipeptides **6l**,**m** were obtained as a 67:33 mixture of diastereomers. In the meantime, an excellent diastereoselectivity (97:3 dr) was achieved in the decarboxylative cyclization of *N*-mesylamide **9n** possessing an *S* stereogenic center in the α-position to the nitrogen. Unfortunately, the configuration of the newly formed quaternary stereogenic center in **6n** could not be established by NMR methods, and all attempts to obtain crystals suitable for X-ray crystallographic analysis were unsuccessful.

**Scheme 4:**
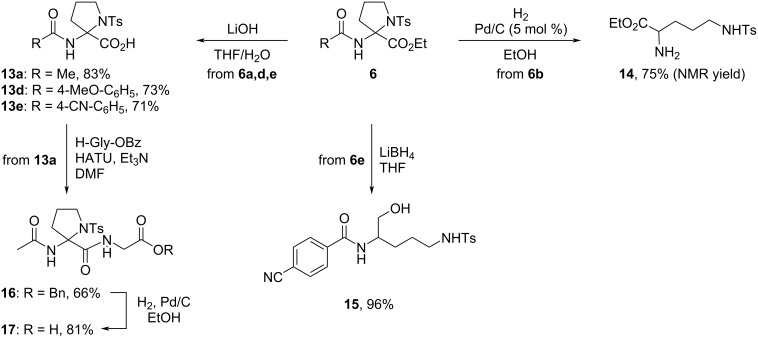
Synthetic modifications of 2-aminoproline derivatives **6**.

*N*-Protected 2-aminoproline derivatives **6** are relatively stable under basic conditions as evidenced by successful hydrolysis of the ester moiety in **6a**,**d**,**e** using aqueous LiOH to provide acids **13a**,**d**,**e** in 71–83% yield (Scheme 4). Carboxylic acid **13a** could be reacted with glycine benzyl ester in the presence of HATU and Et_3_N to form dipeptide **16** (66%). In contrast, *N*-unprotected 2-aminoprolines are unstable and could not be isolated. Thus, the cleavage of the *N*-Cbz protecting group in **6b** under Pd-catalyzed hydrogenolysis afforded diamino acid ester **14** (75% yield) that was likely formed by ring-opening of the unstable *N*-unprotected 2-aminoproline followed by the reduction of the open-chain imine tautomer. Likewise, the open-chain amino alcohol **15** was formed also upon the reduction of the ester moiety with LiBH_4_. In the meantime, the hydrogenolysis of the benzyl ester in dipeptide **16** proceeded smoothly and afforded carboxylic acid **17** in 81% yield (Scheme 4) [16].

## Conclusion

In summary, the developed electrochemical decarboxylative amidation of readily accessible malonic acid monoesters provides access to previously unreported 2-aminoproline derivatives. The decarboxylative amidation proceeds under constant current conditions in an undivided cell in aqueous acetonitrile and involves initial anodic decarboxylation followed by an intramolecular reaction of the formed stabilized cation with tethered nitrogen nucleophiles such as sulfonamides, carbamates, and benzamide. The decarboxylative cyclization of a stereogenic center-containing sulfonamide proceeds with excellent diastereoselectivity (97:3 dr). The *N*-protected 2-aminoproline derivatives can be incorporated into dipeptides by an ester hydrolysis/amide bond formation sequence, and therefore they are suitable for the design of peptidomimetics. Further work is in progress in our laboratory to expand the scope of nucleophiles in the decarboxylative functionalization of malonic acid monoesters.

## Experimental

General procedure for the electrochemical synthesis of pyrrolidines **6a**–**f**,**j**–**n** from the corresponding malonic acid monoesters **9a**–**f**,**j**–**n**.

An undivided electrochemical cell (5 mL, IKA ElectraSyn 2.0) was charged with starting carboxylic acid **9a**–**f**,**j**–**n** (0.2–0.3 mmol) and Et_4_N–BF_4_ (0.025 M), followed by addition of MeCN (2.5 mL) and H_2_O (0.5 mL). A graphite plate (8 × 52.5 × 2 mm; immersed electrode surface area *A* = 1.12 cm^2^) was used as a working electrode and stainless steel or graphite (8 × 52.5 × 2 mm; immersed electrode surface area *A* = 1.12 cm^2^) was used as a counter electrode. The electrolysis was carried out under galvanostatic conditions at room temperature, and 2.0 F charge (if not otherwise noticed) with current density of 12 mA/cm^2^ was passed through the colorless reaction solution. The resulting clear, colorless (sometimes pale yellow) solution was concentrated under reduced pressure and the crude product was purified by column chromatography.

### Cyclic voltammetry studies

CV experiments were carried out in an SVC-2 (ALS, Japan) three-electrode cell using a PalmSens4 (PalmSens). A glassy carbon disk (diameter: 1.6 mm) served as the working electrode and a platinum wire as the counter electrode. The glassy carbon disk was polished using polishing alumina (0.05 μm) prior to each experiment. As a reference, an Ag/AgNO_3_ electrode [silver wire in 0.1 M NBu_4_ClO_4_/MeCN solution; *c*(AgNO_3_) = 0.01 M; *E*_0_ = −87 mV vs Fc/Fc^+^ couple] [17] was used, and this compartment was separated from the rest of the cell with a Vycor frit. Et_4_NBF_4_ (0.1 M, electrochemical grade) was employed as the supporting electrolyte in 5:1 MeCN/H_2_O solution. The electrolyte was purged with argon for at least 3 min prior to recording. Compounds **6a** and **9a** were analyzed at a concentration of 3 mM or 6 mM and at a scan rate of 100 mV s^−1^. The peak potential *E*_p_ was not extracted from background-corrected voltammograms. All CV graphs are plotted using IUPAC polarographic convention.

## Supporting Information

File 1 Detailed experimental procedures, analytical and spectroscopic data for the synthesized compounds, and copies of NMR spectra.

## Data Availability

All data that supports the findings of this study is available in the published article and/or the supporting information of this article.
