# Supplementary material for: Entry to 2-aminoprolines via electrochemical decarboxylative amidation of N‑acetylamino malonic acid monoesters
Source: Beilstein J Org Chem. 2025 Mar 19;21:630–8. doi: 10.3762/bjoc.21.50 (PMC11931647; doi:10.3762/bjoc.21.50)

BEILSTEIN JOURNAL OF ORGANIC CHEMISTRY

Supporting Information

for

Entry to 2-aminoprolines via electrochemical decarboxylative amidation of *N*-acetylamino malonic acid monoesters

Olesja Koleda, Janis Sadauskis, Darja Antonenko, Edvards Janis Treijs, Raivis Davis Steberis and Edgars Suna

Beilstein J. Org. Chem. **2025,** *21,* 630–638. doi:10.3762/bjoc.21.50

Detailed experimental procedures, analytical and spectroscopic data for the synthesized compounds, and copies of NMR spectra

License and Terms: This is a supporting information file under the terms of the Creative Commons Attribution License (https://creativecommons.org/licenses/by/4.0). Please note that the reuse, redistribution and reproduction in particular requires that the author(s) and source are credited and that individual graphics may be subject to special legal provisions.
The license is subject to the *Beilstein Journal of Organic Chemistry* terms and conditions: (https://www.beilstein-journals.org/bjoc/terms)

Contents

General S2
Optimization of decarboxylative cyclization of acid **9a** S2
Synthesis of alkylating reagents **S2a**,**b**, **S3a**,**b**, and **S6** S5
General procedure A S5
General procedure B S5
General procedure C S6
Synthesis of malonic acid monoesters **9a**–**n** S9
General procedure D for synthesis of amidomalonates **S7e**–**g** S9
General procedure E the synthesis of amidomalonates **S7c**,**d** S10
General procedure F for the synthesis of alkylated malonates **7a**–**l** S13
General procedure I for the synthesis of alkylated malonates **S8a**,**b** S19
General procedure J for the synthesis of alkylated malonates **S8c**,**d** S20
General procedure G for the synthesis of acids **8a**–**i** and **9g**–**k** via malonate hydrolysis S21
General procedure H for the synthesis of compounds **9a**–**f**,**l**–**n** by cleavage of Boc group S27
Electrochemical decarboxylation-cyclization S30
General procedure K for electrochemical decarboxylation-cyclization S30
General procedure L for electrochemical decarboxylation-cyclization S31
General procedure M for electrochemical decarboxylation-cyclization S31
Anodic oxidation of pyrrolidine **6a** S39
Deuterium-labeling studies S40
Comparison of ^{1}H NMR spectra of compounds **11d**–***D*** and **11d** S41
Comparison of MS spectra of of compounds **11d**–***D*** and **11d** S42
Cyclic voltammetry S43
Synthetic transformations of compounds **6a**,**b**,**d**,**e** S45
References S49
NMR Spectra S50

General

Unless otherwise noted, all chemicals were used as received from commercial sources. Anhydrous THF, CH_2Cl_2, DMSO, DMF were received from commercial sources. Analytical thin-layer chromatography (TLC) was performed on pre-coated silica gel F-254 plates. Nuclear magnetic resonance spectra were recorded on NMR spectrometers at the following frequencies: ^{1}H, 300 MHz; $^{13}C\{^{1}H\}$, 75 MHz. Chemical shifts are reported in parts per million (ppm) relative to the residual solvent peak as an internal reference. High-resolution mass spectra (HRMS) were recorded on mass spectrometers with a time-of-flight (TOF) mass analyzer using ESI. Optical rotations were recorded on a Kruess P3000 polarimeter. The electrolysis was performed in the ElectraSyn vial (5 mL or 10 mL) with the ElectraSyn vial cap equipped with the anode (graphite) and cathode (graphite or stainless steel).

Optimization of decarboxylative cyclization of acid 9a

The optimization reactions were performed in the ElectraSyn vial (5 mL) with the ElectraSyn vial cap equipped with the anode and cathode (graphite, BDD, Pt, stainless steel). Carboxylic acid **9a** (0.15 mmol, 1 equiv) and a supporting electrolyte (0.075 mmol, 0.15 mmol, or 0.3 mmol) were dissolved in 3 mL of MeCN/H_2O mixture (MeCN/H_2O ratio 2:1 or 5:1), and the electrodes were immersed in the colorless reaction solution (immersed electrode surface area A = 1.12 cm^2). The electrolysis was carried out under galvanostatic conditions at room temperature. After completion, the reaction solution was evaporated, and the crude material was analyzed by ^{1}H NMR using CH_2Br_2 as an internal standard. Parameters such as supporting electrolyte, electrode material, current density, and concentration were optimized. The amount of passed charge was not optimized. The reaction progress was monitored by LC-MS analysis. Initially a charge of 2.0 *F* was applied. Additional amount of charge was applied in a case of incomplete conversion of acid **9a**, but usually not more than 2.5 *F*.

Table S1. Optimization of the supporting electrolyte.

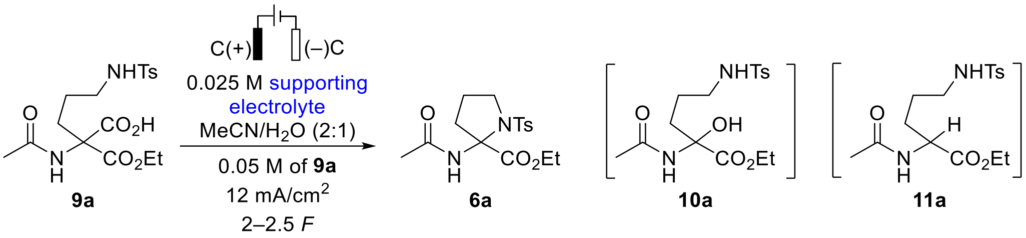

Entry	Supporting electrolyte	NMR yield, %[a]	6a:10a:11a (LC-MS ratio)
1[b]	$LiClO_4$	67[e]	84:16:0
2[c]	K_2CO_3	54	86:3:11
3[c]	Na_2CO_3	54	86:4:10
4[c]	NaOAc	56	71:13:16
5[d]	Bu_4NClO_4	67	85:15:0
6[b]	Et_4NPF_6	66	85:15:0
7[b]	Et_4NBF_4	71	84:16:0

[a] CH_2Br_2 was used as an internal standard. [b] 2.5 *F*. [c] 2.0 *F*. [d] 2.3 *F*. [e] The average yield of two runs.

Table S2. Optimization of the ratio of MeCN/H_2O.

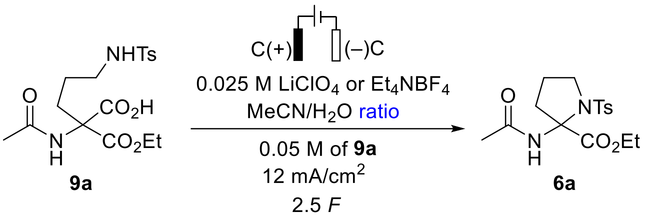

Entry	Supporting electrolyte	MeCN:H_2O	NMR yield, %[a]
1	$LiClO_4$	2:1	67[b]
2	$LiClO_4$	5:1	68
3	$LiClO_4$	5 equiv H_2O	0[c]
4	Et_4NBF_4	2:1	71
5	Et_4NBF_4	5:1	72

[a] CH_2Br_2 was used as an internal standard. [b] The average yield of two runs. [c] No conversion.

Table S3. Optimization of current density.

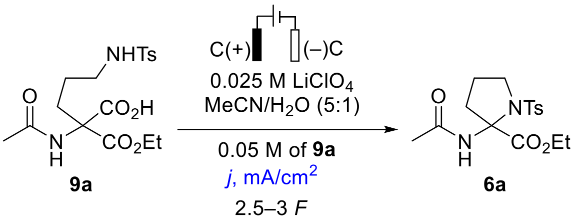

Entry	*j*, mA/cm^2	NMR yield, %[a]
1[b]	12	68
2[c]	4	66[d]
3[b]	14	63

[a] CH_2Br_2 was used as an internal standard. [b] 2.5 F. [c] 3.0 F. [d] Not full conversion of **9a**.

Table S4. Optimization of supporting electrolyte concentration.

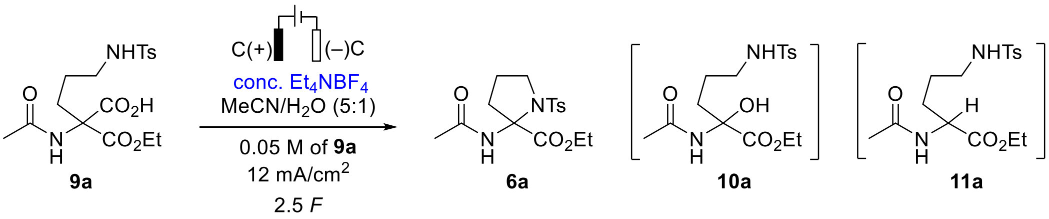

Entry	[Et_4NBF_4], mol/L	NMR yield, %[a]	6a:10a:11a (LC-MS ratio)
1	0.025	72	85:15:0
2	0.05	67	85:13:2
3	0.1	60	84:14:2

[a] CH_2Br_2 was used as an internal standard.

Table S5. Optimization of the cathode material.

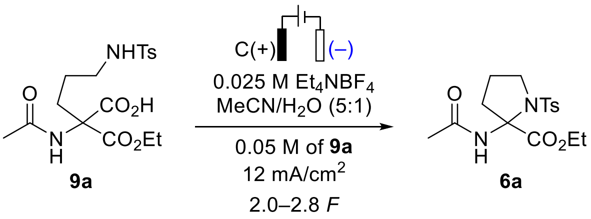

Entry	(–)	NMR yield, %[a]
1[b]	C	72
2[c]	Pt	63
3[c]	SS	70
4[d]	BDD	62

[a] CH_2Br_2 was used as an internal standard. [b] 2.5 *F*. [c] 2.0 *F*. [d] 2.8 *F*.

Synthesis of alkylating reagents S2a,b, S3a,b, and S6

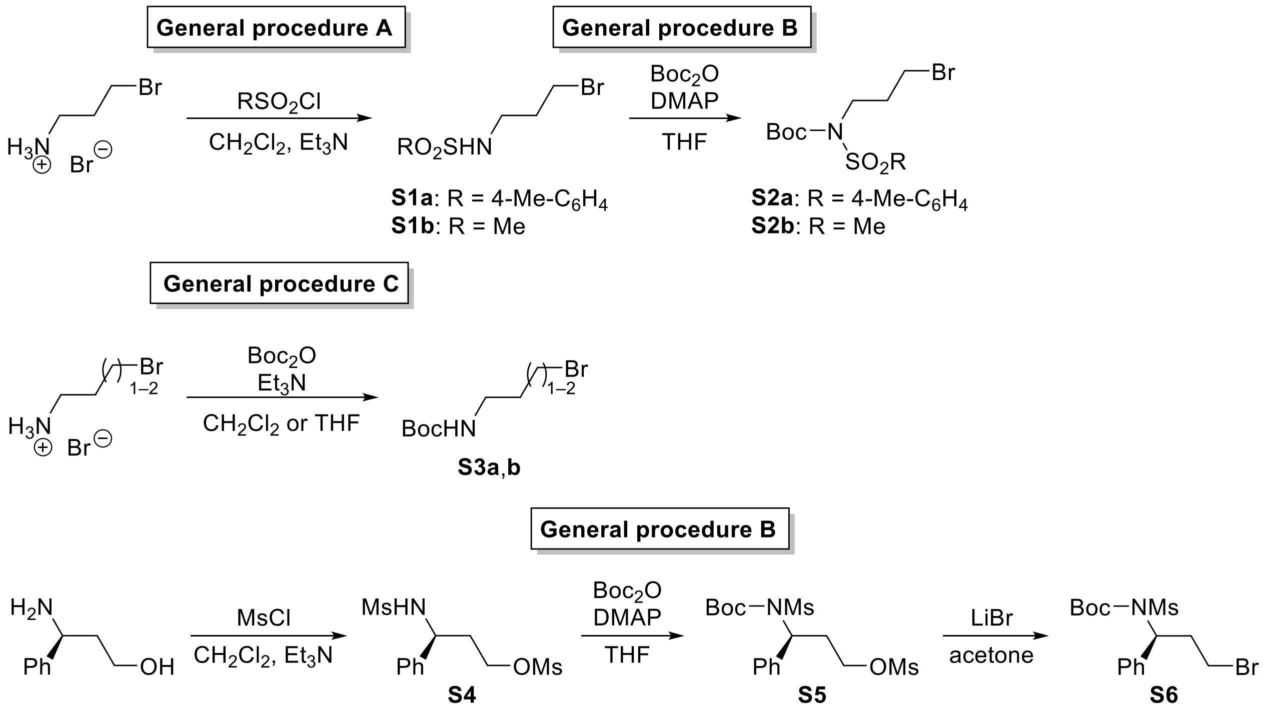

General procedure A

Commercially available 3-bromopropan-1-amine hydrobromide (1.0 equiv) and tosyl chloride or mesyl chloride (1.0 equiv) were dissolved in anhydrous CH_2Cl_2 (4 mL/mmol of the hydrobromide). The solution was cooled to 0 °C in an ice-bath followed by the dropwise addition of Et_3N (2.5 equiv). The resulting white suspension was stirred for 20 minutes at 0 °C (for **S1a**) or 60 minutes at room temperature (for **S1b**). After completion, the reaction mixture was quenched with 1 M aqueous HCl to pH 2. The layers were separated and the aqueous layer was extracted with CH_2Cl_2 (3×3 mL/mmol of hydrobromide). The combined organic layers were washed with water (4 mL/mmol of hydrobromide) and then with brine (4 mL/mmol of hydrobromide), dried over anhydrous Na_2SO_4, filtered and concentrated *in vacuo*. The product was used in the next step without additional purification.

General procedure B

A solution of sulfonamide **S1a**,**b** or **S4** (1 equiv), di-*tert*-butyl dicarbonate (1 equiv) and DMAP (0.2 equiv) in anhydrous THF (5.8 mL/mmol of amine or sulfonamide) was stirred at room temperature for 30–60 minutes. The resulting solution was diluted with water (4 mL/mmol of amine or sulfonamide) and the water layer was extracted with EtOAc (3×4 mL/mmol of amine or sulfonamide). The extracts were combined, washed with water (4 mL/mmol of amine or sulfonamide), dried over anhydrous Na_2SO_4, and concentrated *in vacuo*.

General procedure C

A solution of 3-bromopropan-1-amine hydrobromide or 4-brombutan-1-amine hydrobromide (1 equiv) and di-*tert*-butyl dicarbonate (1–1.2 equiv) was dissolved in CH_2Cl_2 (5 mL/mmol of hydrobromide). The reaction solution was cooled in an ice-bath, and Et_3N (1–1.3 equiv) was added slowly. The colorless solution was stirred at room temperature for 3–16 hours.

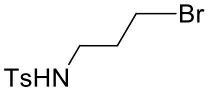

***N*-(3-Bromopropyl)-4-methylbenzene-1-sulfonamide (S1a)** was obtained as a white amorphous solid (5.15 g, 96%) according to the general procedure A from 3-bromopropan-1-amine hydrobromide (4.00 g, 18.3 mmol, 1.0 equiv) and TsCl (3.48 g, 18.3 mmol, 1.0 equiv). The product was used in the next step without additional purification.
^{1}H NMR (300 MHz, $CDCl_3$) δ 7.84 – 7.74 (m, 2H), 7.38 – 7.28 (m, 2H), 5.59 (br. s, 1H), 3.42 (t, *J* = 6.5 Hz, 2H), 3.08 (q, *J* = 6.5 Hz, 2H), 2.44 (s, 3H), 2.03 (p, *J* = 6.5 Hz, 2H). The ^{1}H NMR spectrum was in agreement with that reported in the literature [1].

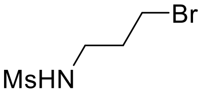

***N*-(3-Bromopropyl)methanesulfonamide (S1b)** was obtained as a colorless oil (986 mg, 100%) according to the general procedure A from 3-bromopropan-1-amine hydrobromide (1.00 g, 4.6 mmol, 1.0 equiv) and MsCl (0.37 mL, 4.8 mmol, 1.05 equiv). The product was used in the next step without additional purification.
^{1}H NMR (300 MHz, $CDCl_3$) δ 5.07 (t, *J* = 6.4 Hz, 1H), 3.47 (t, *J* = 6.4 Hz, 2H), 3.25 (q, *J* = 6.5 Hz, 2H), 2.95 (s, 3H), 2.08 (p, *J* = 6.5 Hz, 2H). The ^{1}H NMR spectrum was in agreement with that reported in the literature [2].

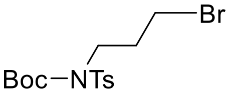

***Tert*-butyl *N*-(3-bromopropyl)-*N*-(4-methylbenzenesulfonyl)carbamate (S2a)** was obtained as a white amorphous solid (5.68 g, 94%) according to the general procedure B from sulfonamide **S1a** (4.50 g, 15.4 mmol). The product was used in the next step without the additional purification.
^{1}H NMR (300 MHz, $CDCl_3$) δ 7.83 – 7.72 (m, 2H), 7.36 – 7.26 (m, 2H), 4.01 – 3.90 (m, 2H), 3.45 (t, *J* = 6.6 Hz, 2H), 2.44 (s, 3H), 2.40 – 2.25 (m, 2H), 1.35 (s, 9H). The ^{1}H NMR spectrum was in agreement with that reported in the literature [3].

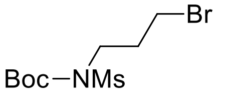

***Tert*-butyl *N*-(3-bromopropyl)-*N*-methanesulfonylcarbamate (S2b)** was obtained according to general procedure B from sulfonamide **S1b** (986 mg, 4.6 mmol). After extraction, the crude material was dissolved in $CHCl_3$ (10 mL) and ~6% aqueous NH_3 was added. The resulting emulsion was stirred at room temperature for 1 hour. The organic layer was separated, washed

with aqueous 0.1 M HCl (15 mL), dried over anhydrous Na_2SO_4, filtered and concentrated to afford the title product as a yellow oil (1.3 g, 90%). The product was used in the next step without the additional purification.

^{1}H NMR (300 MHz, $CDCl_3$) δ 3.86 – 3.76 (m, 2H), 3.40 (t, *J* = 6.6 Hz, 2H), 3.28 (s, 3H), 2.30 – 2.15 (m, 2H), 1.55 (s, 9H).

^{13}C NMR (75 MHz, $CDCl_3$) δ 151.5, 84.9, 45.4, 42.3, 32.8, 29.8, 28.0.

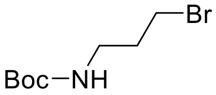

***Tert*-butyl *N*-(3-bromopropyl)carbamate (S3a)** was obtained according to general procedure C from 3-bromopropan-1-amine hydrobromide (500 mg, 2.3 mmol), di-*tert*-butyl dicarbonate (498 mg, 2.3 mmol) and Et_3N (0.32 mL, 2.3 mmol) in CH_2Cl_2 (20 mL). After completion, the reaction mixture was quenched with aqueous 5% NaOH (3×15 mL). The organic layer was separated and washed with water (3×15 mL), dried over Na_2SO_4, filtered and concentrated *in vacuo* to afford the title compound as a colorless oil (469 mg, 86%). The product was used in the next step without additional purification.

^{1}H NMR (300 MHz, $CDCl_3$) δ 4.73 – 4.55 (br. s, 1H), 3.44 (t, *J* = 6.5 Hz, 2H), 3.27 (q, *J* = 6.5 Hz, 2H), 2.11 – 1.96 (m, 2H), 1.44 (s, 9H). The ^{1}H NMR spectrum was in agreement with that reported in the literature [4].

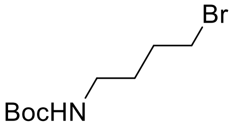

***Tert*-butyl *N*-(4-bromobutyl)carbamate (S3b)** was obtained according to general procedure C from 4-bromobutan-1-amine hydrobromide (930 mg, 4.0 mmol) and di-*tert*-butyl dicarbonate (1.05 g, 4.8 mmol) in presence of Et_3N (0.72 mL, 5.2 mmol). After completion, the reaction mixture was quenched with aqueous 5% HCl (28 mL). The organic layer was separated and washed with water (28 mL), dried over Na_2SO_4, filtered and concentrated *in vacuo*. The crude material was purified by flash column chromatography on silica gel using gradient elution from 9% to 17% EtOAc in petroleum ether to afford the title compound as a colorless oil (747 mg, 74%); analytical TLC on silica gel, 1:10 EtOAc/petroleum ether, R_f = 0.27.

^{1}H NMR (300 MHz, $CDCl_3$) δ 4.61 (s, 1H), 3.40 (t, *J* = 6.7 Hz, 2H), 3.19 – 3.05 (m, 2H), 1.94 – 1.76 (m, 2H), 1.68 – 1.53 (m, 2H), 1.41 (s, 9H). The ^{1}H NMR spectrum was in agreement with that reported in the literature [5].

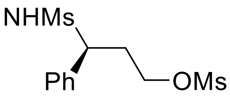

(3*S*)-3-Methanesulfonamido-3-phenylpropyl methanesulfonate (S4). Commercially available (*S*)-3-amino-3-phenyl-1-propanol (1.5 mL, 10.3 mmol, 1.0 equiv) was dissolved in anhydrous CH_2Cl_2 (30 mL), and the reaction solution was cooled in an ice-bath. Then, MsCl (4.0 mL, 51.8 mmol, 5 equiv) and Et_3N (4.3 mL, 31.1 mmol, 3 equiv) were sequentially

slowly added. The yellow suspension was stirred at room temperature overnight. After completion, all volatiles were removed *in vacuo*. The purification of the crude material by reversed phase flash column chromatography using gradient elution from 5% to 100% MeCN in water containing 0.01% TFA afforded the title compound as a colorless oil (2.27 g, 71%).

^{1}H NMR (300 MHz, $CDCl_3$) δ 7.47 – 7.28 (m, 5H), 5.25 (d, *J* = 8.8 Hz, 1H), 4.71 – 4.58 (m, 1H), 4.37 (ddd, *J* = 10.3, 7.5, 5.0 Hz, 1H), 4.24 (ddd, *J* = 10.3, 5.9, 5.0 Hz, 1H), 3.05 (s, 3H), 2.60 (s, 3H), 2.35 – 2.12 (m, 2H).

^{13}C NMR (75 MHz, $CDCl_3$) δ 140.2, 129.5, 128.7, 126.5, 66.6, 55.1, 41.9, 37.5, 36.9.

HRMS (ESI/Q-TOF) *m*/*z*: $[M+Na]^+$ Calcd for $C_{11}H_{17}NO_5S_2Na$ 430.0970; Found 430.0980.

$[\alpha]^{20}_{D}$ –40 (*c* 1.0, $CHCl_3$).

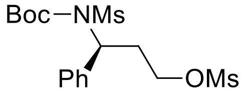

***Tert*-butyl *N*-methanesulfonyl-*N*-[(1*S*)-3-(methanesulfonyloxy)-1-phenylpropyl]carbamate (S5)** was obtained according to general procedure B form protected hydroxylamine **S4** (2.24 g, 7.3 mmol, 1.0 equiv). The crude material was purified by reversed phase flash column chromatography using gradient elution from 5% to 100% MeCN in water containing 0.01% TFA to afford the title compound as a colorless oil (2.43 g, 82%).

^{1}H NMR (300 MHz, $CDCl_3$) δ 7.40 – 7.27 (m, 5H), 5.67 (dd, *J* = 10.5, 5.1 Hz, 1H), 4.49 – 4.31 (m, 2H), 3.27 (s, 3H), 3.06 (s, 3H), 2.98 – 2.79 (m, 1H), 2.60 (ddt, *J* = 14.8, 8.4, 5.4 Hz, 1H), 1.40 (s, 9H).

^{13}C NMR (75 MHz, $CDCl_3$) δ 151.5, 138.7, 128.7, 128.1, 127.4, 85.5, 66.7, 56.8, 42.7, 37.6, 31.2, 28.0.

HRMS (ESI/Q-TOF) *m*/*z*: $[M+Na]^+$ Calcd for $C_{16}H_{25}NO_7S_2Na$ 430.0970; Found 430.0980.

$[\alpha]^{20}_{D}$ –40 (*c* 1.0, $CHCl_3$).

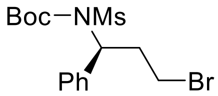

***Tert*-butyl *N*-[(1*S*)-3-bromo-1-phenylpropyl]-*N*-methanesulfonylcarbamate (S6)**. Mesylate **S5** (2.39 g, 5.8 mmol, 1.0 equiv) and LiBr (2.55 g, 29.4 mmol, 5 equiv) were heated under reflux in acetone (50 mL) for 4 hours. Then, all volatiles were removed *in vacuo*. The crude material was purified by flash column chromatography on silica gel using gradient elution from 0% to 80% EtOAc in petroleum ether to afford the title compound as a colorless thick oil (1.27 g, 55%); analytical TLC on silica gel, 1:1 EtOAc/petroleum ether, R_f = 0.71.

^{1}H NMR (300 MHz, $CDCl_3$) δ 7.44 – 7.24 (m, 5H), 5.68 (dd, *J* = 9.6, 5.8 Hz, 1H), 3.60 – 3.41 (m, 2H), 3.23 (s, 3H), 3.07 – 2.89 (m, 1H), 2.80 – 2.62 (m, 1H), 1.43 (s, 9H).

^{13}C NMR (75 MHz, $CDCl_3$) δ 151.6, 138.7, 128.7, 128.1, 127.6, 85.4, 59.1, 42.7, 35.1, 29.8, 28.0.

$[\alpha]^{20}_{D}$ –72 (*c* 1.0, $CHCl_3$).

Synthesis of malonic acid monoesters 9a–n

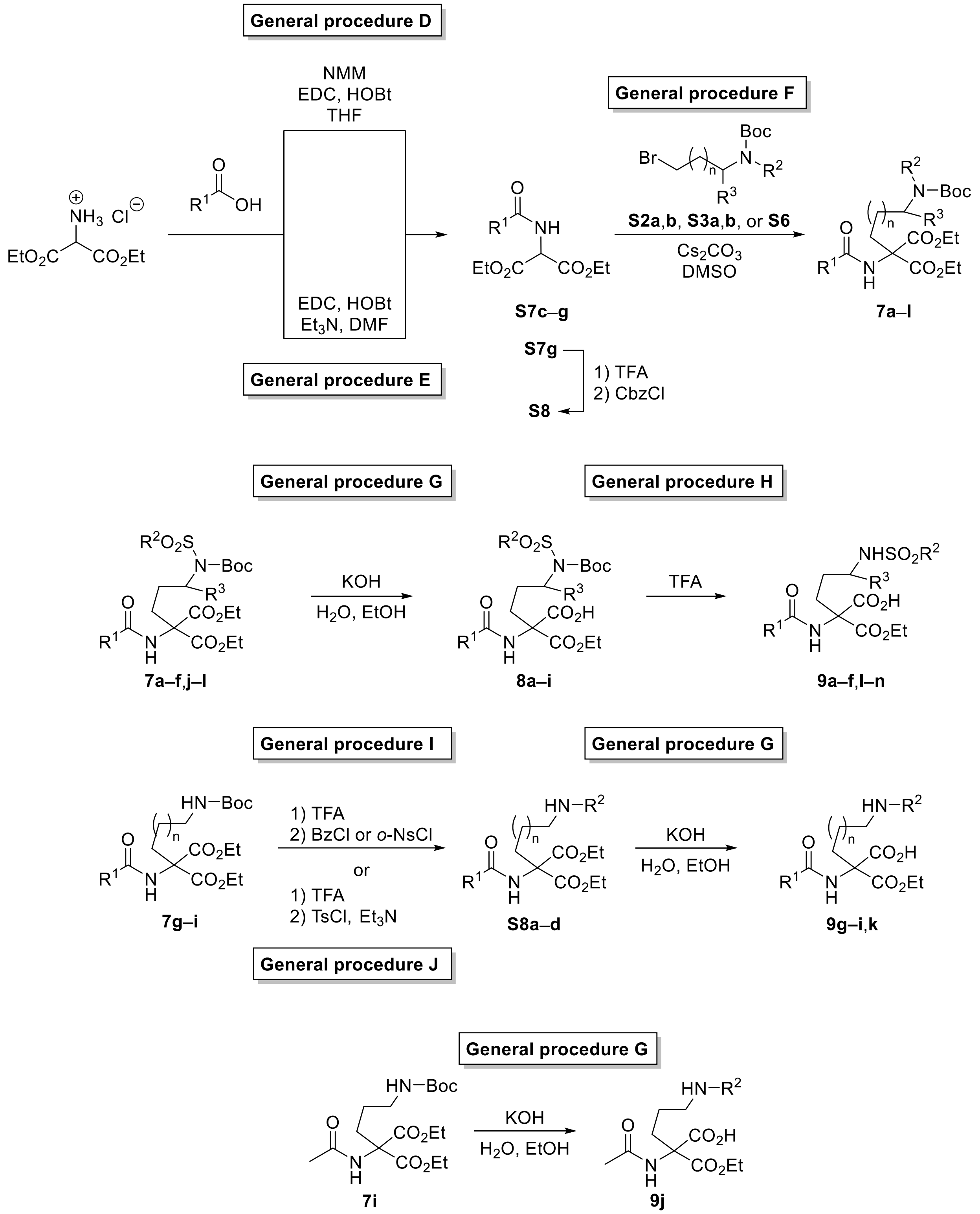

General procedure D for synthesis of amidomalonates S7e–g

N-Methylmorpholine (2.0 equiv) was added to a solution of carboxylic acid (1.0–1.1 equiv) and diethyl aminomalonate hydrochloride (1.0 equiv) in anhydrous THF (3 mL/mmol of diethyl aminomalonate

hydrochloride) at 0 °C. After 10 min HOBt (1.1 equiv) and EDC×HCl (1.1 equiv) were sequentially added. The resulting colorless slurry was warmed to room temperature and stirred until completion (usually 1.5 hours). The reaction mixture was treated with saturated aqueous NH_4Cl (10 mL/mmol of diethyl aminomalonate hydrochloride) and extracted with EtOAc (3×10 mL/mmol of diethyl aminomalonate hydrochloride). The organic layers were combined and sequentially washed with saturated aqueous $NaHCO_3$ (10 mL/mmol of diethyl aminomalonate hydrochloride) and brine (10 mL/mmol of diethyl aminomalonate hydrochloride). The aqueous layer was separated, and the organic phase was dried over anhydrous Na_2SO_4, filtered, and concentrated *in vacuo*.

General procedure E the synthesis of amidomalonates S7c,d

Et_3N (2.8 equiv) was added to a solution of carboxylic acid (1.0–1.1 equiv) and diethyl aminomalonate hydrochloride (1.0 equiv) in anhydrous DMF (3 mL/mmol of diethyl aminomalonate hydrochloride) at 0 °C. After stirring for 10 min at this temperature, HOBt (1.2 equiv) and EDC×HCl (1.2 equiv) were added consecutively. The reaction mixture was warmed to room temperature and stirred for 1.5 hours. The white suspension was treated with saturated aqueous NH_4Cl (10 mL/mmol of diethyl aminomalonate hydrochloride) and extracted with EtOAc (3×10 mL/mmol of diethyl aminomalonate hydrochloride). The organic layers were combined and sequentially washed with saturated aqueous $NaHCO_3$ (10 mL/mmol of diethyl aminomalonate hydrochloride) and brine (10 mL/mmol of diethyl aminomalonate hydrochloride). The aqueous layer was separated, and the organic phase was dried over anhydrous Na_2SO_4, filtered, and concentrated *in vacuo*.

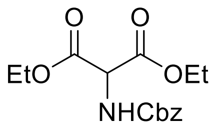

1,3-Diethyl 2-{[(benzyloxy)carbonyl]amino}propanedioate (S7a). Diethyl aminomalonate hydrochloride (635 mg, 3 mmol, 1.0 equiv) and Na_2CO_3 (382 mg, 3.6 mmol, 1.2 equiv) were dissolved in H_2O (5 mL). The colorless clear solution was cooled in an ice-bath followed by the addition of benzyl chloroformate (0.43 mL, 3 mmol, 1.0 equiv). After 2 hours, the mixture was warmed to room temperature and stirred overnight. The clear reaction solution was acidified by aqueous 4 M HCl to pH 2 and extracted with EtOAc (3×10 mL). The combined organic layers were washed with brine (10 mL), dried over anhydrous Na_2SO_4, filtered, and concentrated *in vacuo* to afford the title compound as a thick light yellow colorless oil (878 mg, 95%) that was used in the next step without purification.

^{1}H NMR (300 MHz, $CDCl_3$) δ 7.37 – 7.27 (m, 5H), 5.91 (d, *J* = 7.7 Hz, 1H), 5.12 (s, 2H), 5.00 (d, *J* = 7.7 Hz, 1H), 4.33 – 4.14 (m, 4H), 1.27 (t, *J* = 7.2 Hz, 5H). The ^{1}H NMR spectrum was in agreement with that reported in the literature [6].

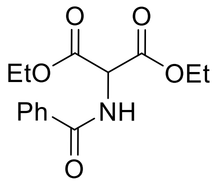

1,3-Diethyl 2-(phenylformamido)propanedioate (**S7b**). Diethyl aminomalonate hydrochloride (635 mg, 3.0 mmol, 1 equiv) was dissolved in pyridine (8 mL). The colorless solution was cooled in an ice-bath, and benzoyl chloride (0.35 mL, 2.9 mmol, 1 equiv) was added dropwise. The light-yellow solution was stirred overnight at room temperature, and the solvent was evaporated *in vacuo*. The residue was diluted with water (12 mL) and extracted with EtOAc (12 mL). The aqueous phase was extracted with EtOAc (2×10 mL). Organic layers were combined, dried over anhydrous Na_2SO_4, filtered and concentrated *in vacuo*. The product was purified by flash column chromatography on silica gel using isocratic elution with 20% EtOAc in petroleum ether to afford the title product as a white amorphous solid (652 mg, 78%); analytical TLC on silica gel, 1:4 EtOAc/petroleum ether, R_f = 0.26.

^{1}H NMR (300 MHz, $CDCl_3$) δ 7.87 – 7.78 (m, 2H), 7.54 – 7.46 (m, 1H), 7.46 – 7.37 (m, 2H), 7.22 (d, *J* = 6.9 Hz, 1H), 5.34 (d, *J* = 6.9 Hz, 1H), 4.36 – 4.16 (m, 4H), 1.28 (t, *J* = 7.1 Hz, 3H). The ^{1}H NMR spectrum was in agreement with that reported in the literature [6].

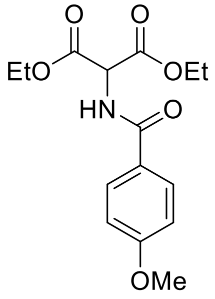

1,3-Diethyl 2-[(4-methoxyphenyl)formamido]propanedioate (S7c) was obtained as a white amorphous solid (928 mg, 99%) from diethyl aminomalonate hydrochloride (635 mg, 3.0 mmol) and 4-methoxybenzoic acid (502 mg, 3.3 mmol) according to general procedure E. The crude product was used in the next step without additional purification.

^{1}H NMR (300 MHz, $CDCl_3$) δ 7.85 – 7.78 (m, 2H), 7.03 (d, *J* = 6.8 Hz, 1H), 6.98 – 6.88 (m, 2H), 5.33 (d, *J* = 6.8 Hz, 1H), 4.40 – 4.21 (m, 4H), 3.86 (s, 3H), 1.32 (t, *J* = 7.1 Hz, 6H). The ^{1}H NMR spectrum was in agreement with that reported in the literature [6].

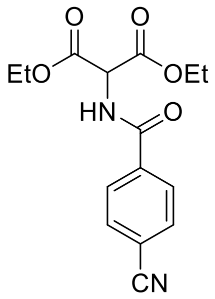

1,3-Diethyl 2-[(4-cyanophenyl)formamido]propanedioate (S7d) was obtained as a white amorphous solid (608 mg, 99%) from diethyl aminomalonate hydrochloride (423 mg, 2.0 mmol) and 4-cyanobenzoic acid (324 mg, 2.2 mmol) according to the general procedure E. The crude product was used in the next step without additional purification.

^{1}H NMR (300 MHz, $CDCl_3$) δ 7.99 – 7.89 (m, 2H), 7.81 – 7.71 (m, 2H), 7.20 (d, *J* = 6.7 Hz, 1H), 5.31 (d, *J* = 6.7 Hz, 1H), 4.42 – 4.20 (m, 4H), 1.32 (t, *J* = 7.1 Hz, 6H). The ^{1}H NMR spectrum was in agreement with that reported in the literature [6].

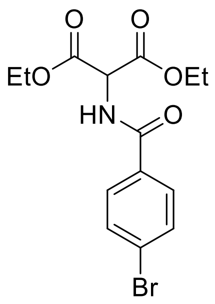

1,3-Diethyl 2-[(4-bromophenyl)formamido]propanedioate (S7e) was obtained as a white amorphous solid (5.1 g, 95%) from diethyl aminomalonate hydrochloride (3.2 g, 15 mmol) and 4-bromobenzoic acid (3.0 g, 15 mmol) according to the general procedure D. The crude product was used in the next step without additional purification.

^{1}H NMR (300 MHz, $CDCl_3$) δ 7.73 – 7.68 (m, 2H), 7.61 – 7.55 (m, 2H), 7.12 (d, *J* = 6.7 Hz, 1H), 5.31 (d, *J* = 6.7 Hz, 1H), 4.36 – 4.23 (m, 4H), 1.31 (t, *J* = 7.1 Hz, 6H).

The ^{1}H NMR spectrum was in agreement with that reported in the literature [6].

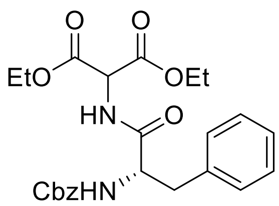

1,3-Diethyl 2-[(2*S*)-2-{[(benzyloxy)carbonyl]amino}-3-phenylpropanamido]propanedioate (S7f) was obtained as a white amorphous solid (1.08 g, 79%) from diethyl aminomalonate hydrochloride (635 mg, 3.0 mmol) and *N*-Cbz-*L*-phenylalanine (988 mg, 3.3 mmol) according to the general procedure D. The title product was used in the next step without additional purification; analytical TLC on silica gel, 1:5 EtOAc/petroleum ether, R_f = 0.12.

^{1}H NMR (300 MHz, $CDCl_3$) δ 7.40 – 7.13 (m, 10H), 6.96 (d, *J* = 6.7 Hz, 1H), 5.38 (d, *J* = 8.1 Hz, 1H), 5.15 – 5.04 (m, 3H), 4.65 – 4.51 (m, 1H), 4.34 – 4.10 (m, 4H), 3.18 – 3.01 (m, 2H), 1.28 (t, *J* = 7.1 Hz, 3H), 1.27 (t, *J* = 7.1 Hz, 3H).

^{13}C NMR (75 MHz, DMSO) δ 172.1, 166.3, 166.2, 155.9, 138.0, 137.0, 129.3, 128.3, 128.0, 127.7, 127.4, 126.3, 65.2, 61.9, 61.8, 56.2, 55.7, 37.3, 13.9.

HRMS (ESI/Q-TOF) *m*/*z*: $[M+H]^+$ Calcd for $C_{24}H_{28}N_2O_7S$ 457.1975; Found 457.1983.

$[\alpha]^{20}_D$ –2 (*c* 1.0, $CHCl_3$).

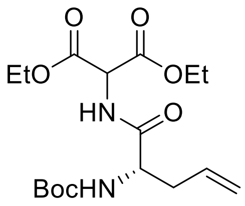

1,3-Diethyl 2-[(2*S*)-2-{[(*tert*-butoxy)carbonyl]amino}pent-4-enamido]propanedioate (S7g) was obtained from diethyl aminomalonate hydrochloride (634 mg, 3 mmol) and (*S*)-2-((*tert*-butoxycarbonyl)amino)pent-4-enoic acid (710 mg, 3.3 mmol) according to the general procedure D. The crude product was purified by flash column chromatography on silica gel using isocratic elution with 25% EtOAc in hexane to afford the title compound as a colorless semisolid (1.05 g, 94%); analytical TLC on silica gel, 1:4 EtOAc/hexane, R_f = 0.27.

^{1}H NMR (300 MHz, $CDCl_3$) δ 7.23 (d, *J* = 6.9 Hz, 1H), 5.71 (ddt, *J* = 17.3, 10.2, 7.1 Hz, 1H), 5.16 – 5.03 (m, 4H), 4.31 – 4.09 (m, 5H), 2.47 (t, *J* = 6.7 Hz, 2H), 1.38 (s, 9H), 1.23 (t, *J* = 7.1 Hz, 6H). The ^{1}H NMR spectrum was in agreement with that reported in the literature [6].

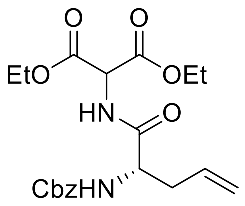

1,3-Diethyl 2-[(2*S*)-2-{[(benzyloxy)carbonyl]amino}pent-4-enamido]propane dioate (S8). The reaction was performed in a 25 mL round bottom flask under argon following the reported procedure [7]. The malonate derivative **S7g** (1.05 g, 2.8 mmol, 1 equiv) was dissolved in anhydrous CH_2Cl_2 (7.5 mL) followed by the addition of TFA (1.3 mL, 16.9 mmol, 6 equiv). The colorless reaction solution was stirred at room temperature for 17 hours. CH_2Cl_2 was removed from the colorless reaction solution *in vacuo*. The residue was suspended in 2 mL of anhydrous toluene and concentrated *in vacuo* (repeated twice). Then, the crude unprotected amine (as a thick oil) was dissolved in anhydrous CH_2Cl_2 (10 mL) followed by the addition of DIPEA (1.5 mL, 8.5 mmol, 3 equiv) and CbzCl (0.6 mL, 4.2 mmol, 1.5 equiv). The reaction solution was stirred at room temperature overnight whereupon it was washed with water (10 mL) and then with brine (10 mL). The organic layer was dried over anhydrous Na_2SO_4, filtered and concentrated *in vacuo*. The crude product was purified by flash column chromatography on silica gel using gradient elution from 20% to 33% EtOAc in petroleum ether to afford 822 mg (72%) of the title compound as a colorless semisolid; analytical TLC on silica gel, 1:2 EtOAc/petroleum ether, R_f = 0.39.

^{1}H NMR (300 MHz, $CDCl_3$) δ 7.39 – 7.29 (m, 5H), 7.02 (d, *J* = 6.8 Hz, 1H), 5.87 – 5.67 (m, 1H), 5.29 (d, *J* = 6.8 Hz, 1H), 5.21 – 5.17 (m, 1H), 5.16 – 5.08 (m, 4H), 4.40 – 4.32 (m, 1H), 4.33 – 4.18 (m, 4H), 2.56 (t, *J* = 6.7 Hz, 2H), 1.29 (t, *J* = 7.1 Hz, 6H).

^{13}C NMR (75 MHz, $CDCl_3$) δ 170.9, 166.0, 136.2, 132.5, 128.7, 128.4, 128.3, 119.9, 67.4, 62.9, 56.6, 54.0, 36.9, 14.1.

HRMS (ESI/Q-TOF) *m*/*z*: $[M+H]^+$ Calcd for $C_{20}H_{27}N_2O_7$ 407.1818; Found 407.1822.

$[\alpha]^{20}_D$ –12 (*c* 1.0, $CHCl_3$).

General procedure F for the synthesis of alkylated malonates 7a–l

Diethyl 2-acetamidomalonate (1 equiv) was dissolved in anhydrous DMSO (1.8 mL/mmol of diethyl 2-acetamidomalonate) in a pressure tube (25 mL or 75 mL) under argon and Cs_2CO_3 (1.2 equiv) was added. The reaction mixture was stirred at room temperature for 1 hour. Then, the corresponding alkyl bromide (1.1 equiv) was added at room temperature and well-stirred reaction suspension (usually light yellow) was heated at 65 °C in an oil bath for 2–4 hours. After completion, the yellow/orange reaction suspension was cooled to room temperature and ice-cold water (2 mL/mmol malonate) and EtOAc (1 mL/mmol malonate) were added. The formed solution was stirred at room temperature for 30 minutes, then transferred to a separatory funnel and extracted with EtOAc (3×5 mL/mmol malonate). The organic layers were combined, washed with brine (10 mL/mmol of malonate), dried over anhydrous Na_2SO_4, filtered, and concentrated. The crude material was purified by using flash column chromatography.

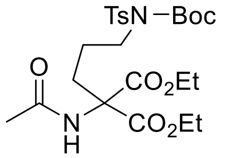

1,3-Diethyl 2-(3-{*N*-[(*tert*-butoxy)carbonyl]-4-methylbenzenesulfonamido}propyl)-2-acetamidopropanedioate (7a) was obtained from diethyl 2-acetamidomalonate (869 mg, 4.0 mmol) and alkyl bromide **S2a** (1.73 g, 4.4 mmol) according to general procedure F. The crude product was purified by flash column chromatography on silica gel using gradient elution from 10% to 80% EtOAc in petroleum ether to afford 1.71 g (81%) of the title compound as a thick pale-yellow oil; analytical TLC on silica gel, 2:1 EtOAc/petroleum ether, R_f = 0.15.

^{1}H NMR (300 MHz, $CDCl_3$) δ 7.79 – 7.70 (m, 2H), 7.34 – 7.25 (m, 2H), 6.83 (s, 1H), 4.26 (q, *J* = 7.1 Hz, 4H), 3.77 (t, *J* = 7.6 Hz, 2H), 2.43 (s, 3H), 2.42 – 2.35 (m, 2H)., 2.05 (s, 3H), 1.69 – 1.52 (m, 2H), 1.33 (s, 9H), 1.26 (t, *J* = 7.1 Hz, 3H).

^{13}C NMR (75 MHz, $CDCl_3$) δ 169.2, 168.0, 150.9, 144.2, 137.3, 129.3, 127.8, 84.3, 66.3, 62.7, 46.8, 29.4, 27.9, 25.0, 23.1, 21.7, 14.0.

HRMS (ESI/Q-TOF) *m*/*z*: $[M+Na]^+$ Calcd for $C_{24}H_{36}N_2O_9SNa$ 551.2039; Found 551.2051.

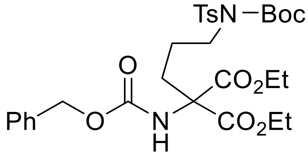

1,3-Diethyl 2-{[(benzyloxy)carbonyl]amino}-2-(3-{*N*-[(*tert*-butoxy)carbonyl]-4-methylbenzenesulfonamido}propyl)propanedioate (7b) was obtained from malonate **S7a** (818 mg, 2.6 mmol) and alkyl bromide **S2a** (1.14 g, 2.9 mmol) according to general procedure F. The crude product was purified by flash column chromatography on silica gel using gradient elution from 17% to 20% EtOAc in petroleum ether to afford 1.45 g (88%) of the title compound as a thick colorless oil; analytical TLC on silica gel, 1:4 EtOAc/petroleum ether, R_f = 0.33.

^{1}H NMR (300 MHz, $CDCl_3$) δ 7.80 – 7.70 (m, 2H), 7.40 – 7.23 (m, 7H), 6.23 (s, 1H), 5.10 (s, 2H), 4.24 (q, *J* = 7.0 Hz, 4H), 3.79 (t, *J* = 7.6 Hz, 2H), 2.43 (s, 3H), 2.41 – 2.32 (m, 2), 1.70 – 1.57 (m, 1H), 1.33 (s, 9H), 1.23 (t, *J* = 7.0 Hz, 6H).

^{13}C NMR (75 MHz, $CDCl_3$) δ 167.9, 154.5, 150.9, 144.2, 137.5, 129.4, 128.7, 128.3, 128.2, 128.0, 84.3, 67.1, 66.6, 62.9, 46.8, 29.9, 28.0, 24.8, 21.8, 14.1.

HRMS (ESI/Q-TOF) *m*/*z*: $[M+Na]^+$ Calcd for $C_{30}H_{40}N_2O_{10}SNa$ 643.2301; Found 643.2306.

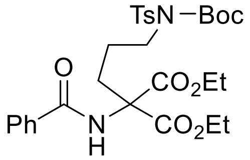

1,3-Diethyl 2-(3-{*N*-[(*tert*-butoxy)carbonyl]-4-methylbenzenesulfonamido} propyl)-2-(phenylformamido)propanedioate (7c) was obtained from malonate **S7b** (554 mg, 2.0 mmol) and alkyl bromide **S2a** (856 mg, 2.2 mmol) according to general procedure F. The crude product was purified by flash column chromatography on silica gel using gradient elution from 17% to 25% EtOAc in petroleum ether to

afford 853 mg (73%) of the title compound as a colorless semisolid; analytical TLC on silica gel, 1:4 EtOAc/petroleum ether, R_f = 0.17.

^{1}H NMR (300 MHz, $CDCl_3$) δ 7.90 – 7.80 (m, 2H), 7.77 – 7.67 (m, 2H), 7.60 – 7.48 (m, 2H), 7.52 – 7.40 (m, 2H), 7.28 – 7.19 (m, 2H), 4.29 (q, *J* = 7.1 Hz, 4H), 3.79 (t, *J* = 7.5 Hz, 2H), 2.59 – 2.47 (m, 2H), 2.40 (s, 3H), 1.75 – 1.58 (m, 2H), 1.30 (s, 9H), 1.27 (d, *J* = 7.1 Hz, 6H).

^{13}C NMR (75 MHz, $CDCl_3$) δ 168.2, 166.2, 150.9, 144.2, 137.4, 133.6, 132.1, 129.4, 128.8, 128.0, 127.4, 84.3, 66.6, 62.9, 46.9, 29.6, 28.0, 25.2, 21.7, 14.1.

HRMS (ESI/Q-TOF) *m*/*z*: $[M+Na]^+$ Calcd for $C_{29}H_{38}N_2O_9SNa$ 613.2196; Found 613.2211.

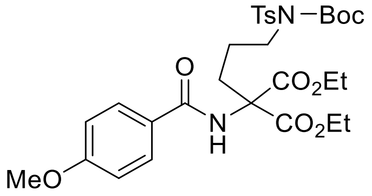

1,3-Diethyl 2-(3-{*N*-[(*tert*-butoxy)carbonyl]-4-methylbenzenesulfonamido}propyl)-2-[(4-methoxyphenyl)formamido]propanedioate (7d) was obtained from malonate **S7c** (464 mg, 1.5 mmol) and alkyl bromide **S2a** (647 mg, 1.6 mmol) according to general procedure F. The crude product was purified by flash column chromatography on silica gel using gradient elution from 40% to 60% EtOAc in petroleum ether to afford 690 mg (74%) of the title compound as a colorless amorphous solid; analytical TLC on silica gel, 1:1.5 EtOAc/petroleum ether, R_f = 0.18.

^{1}H NMR (300 MHz, $CDCl_3$) δ 7.87 – 7.76 (m, 2H), 7.77 – 7.66 (m, 2H), 7.44 (s, 1H), 7.28 – 7.18 (m, 2H), 6.99 – 6.88 (m, 2H), 4.28 (q, *J* = 7.1 Hz, 4H), 3.85 (s, 3H), 3.78 (t, *J* = 7.6 Hz, 2H), 2.57 – 2.45 (m, 2H), 2.40 (s, 3H), 1.73 – 1.57 (m, 2H), 1.29 (s, 9H), 1.25 (t, *J* = 7.1 Hz, 6H).

^{13}C NMR (75 MHz, $CDCl_3$) δ 168.3, 165.7, 162.7, 150.9, 144.2, 137.3, 129.4, 129.2, 128.0, 125.9, 113.9, 84.3, 66.5, 62.8, 55.6, 46.9, 29.7, 27.9, 25.2, 21.7, 14.1.

HRMS (ESI/Q-TOF) *m*/*z*: $[M+Na]^+$ Calcd for $C_{30}H_{40}N_2O_{10}SNa$ 643.2301; Found 643.2311.

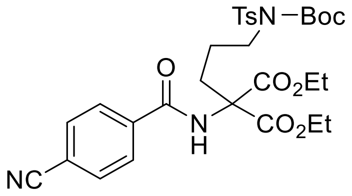

1,3-Diethyl 2-(3-{*N*-[(*tert*-butoxy)carbonyl]-4-methylbenzenesulfonamido}propyl)-2-[(4-cyanophenyl)formamido]propanedioate (7e) was obtained from malonate **S7d** (304 mg, 1.0 mmol) and alkyl bromide **S2a** (432 mg, 1.1 mmol) according to the general procedure F. The crude product was purified by flash column chromatography on silica gel using gradient elution from 25% to 50% EtOAc in petroleum ether to afford 390 mg (63%) of the title compound as a thick colorless oil; analytical TLC on silica gel, 1:3 EtOAc/petroleum ether, R_f = 0.25.

^{1}H NMR (300 MHz, $CDCl_3$) δ 7.99 – 7.89 (m, 2H), 7.79 – 7.69 (m, 2H), 7.74 – 7.64 (m, 2H), 7.58 (s, 1H), 7.29 – 7.20 (m, 2H), 4.28 (q, *J* = 7.1 Hz, 4H), 3.76 (t, *J* = 7.5 Hz, 2H), 2.57 – 2.45 (m, 2H), 2.40 (s, 3H), 1.74 – 1.57 (m, 2H), 1.28 (s, 9H), 1.26 (t, *J* = 7.1 Hz, 6H).

^{13}C NMR (75 MHz, $CDCl_3$) δ 167.7, 164.4, 150.8, 144.3, 137.4, 137.2, 132.6, 129.3, 128.0, 127.8, 118.0, 115.5, 84.3, 66.7, 63.1, 46.7, 29.3, 27.8, 25.1, 21.6, 14.0.

HRMS (ESI/Q-TOF) *m*/*z*: $[M+Na]^+$ Calcd for $C_{30}H_{37}N_3O_9SNa$ 638.2148; Found 638.2161.

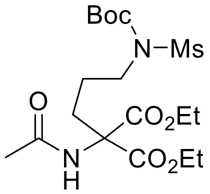

1,3-Diethyl 2-(3-{*N*-[(*tert*-butoxy)carbonyl]methanesulfonamido}propyl)-2-acetamido propanedioate (7f) was obtained from diethyl 2-acetamidomalonate (815 mg, 3.7 mmol) and alkyl bromide **S2b** (1.3 g, 4.1 mmol) according to the general procedure F. The crude product was purified by reversed phase flash column chromatography using gradient elution from 0% to 100% MeCN in water containing 0.01% TFA to afford 1.25 g (74%) of the title compound as a thick colorless oil.

^{1}H NMR (300 MHz, $CDCl_3$) δ 6.78 (s, 1H), 4.20 (q, *J* = 7.1 Hz, 4H), 3.62 (t, *J* = 7.2 Hz, 2H), 3.23 (s, 3H), 2.37 – 2.25 (m, 2H), 2.00 (s, 3H), 1.50 (s, 9H), 1.49 – 1.38 (m, 2H), 1.22 (t, *J* = 7.1 Hz, 6H).

^{13}C NMR (75 MHz, $CDCl_3$) δ 169.3, 167.9, 151.5, 84.7, 66.2, 62.7, 45.9, 42.3, 29.3, 28.0, 24.4, 23.1, 14.0.

HRMS (ESI/Q-TOF) *m*/*z*: $[M+Na]^+$ Calcd for $C_{18}H_{32}N_2O_9SNa$ 475.1726; Found 475.1731.

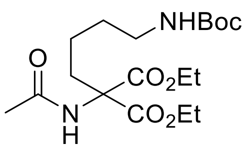

1,3-Diethyl 2-(4-{[(*tert*-butoxy)carbonyl]amino}butyl)-2-acetamidopropanedioate (7g) was obtained from diethyl 2-acetamidomalonate (260 mg, 1.2 mmol) and alkyl bromide **S3b** (330 mg, 1.3 mmol) according to the general procedure F. The crude product was purified by reversed phase flash column chromatography using gradient elution from 5% to 100% MeCN in water containing 0.01% TFA to afford 330 mg (71%) of the title compound as a thick colorless oil; analytical TLC on silica gel, 1:1 EtOAc/petroleum ether, R_f = 0.39.

^{1}H NMR (300 MHz, $CDCl_3$) δ 6.79 (s, 1H), 4.58 (t, *J* = 6.4 Hz, 1H), 4.19 (q, *J* = 7.2 Hz, 4H), 3.03 (q, *J* = 6.8 Hz, 2H), 2.32 – 2.21 (m, 2H), 1.99 (s, 3H), 1.49 – 1.34 (m, 2H), 1.38 (s, 9H), 1.20 (t, *J* = 7.1 Hz, 6H), 1.16 – 1.00 (m, 2H).

^{13}C NMR (75 MHz, $CDCl_3$) δ 169.2, 168.1, 156.0, 79.0, 66.5, 62.5, 40.3, 31.9, 29.8, 28.4, 23.0, 20.9, 14.0.

HRMS (ESI/Q-TOF) *m*/*z*: $[M+Na]^+$ Calcd for $C_{18}H_{32}N_2O_7Na$ 411.2107; Found 411.2123.

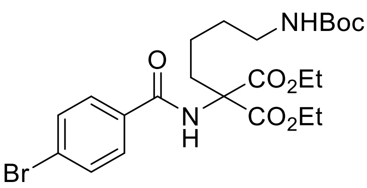

1,3-Diethyl 2-[(4-bromophenyl)formamido]-2-(4-{[(*tert*-butoxy)carbonyl]amino}butyl)propanedioate (7h) was obtained from **S7e** (978 mg, 2.7 mmol) and alkyl bromide **S3b** (757 mg, 3.0 mmol) according to the general procedure F. The crude product was purified by reversed phase flash column chromatography using gradient elution from 5% to 100% MeCN in water

containing 0.01% TFA to afford 480 mg (33%) of the title compound as a colorless semisolid; analytical TLC on silica gel, 1:1 EtOAc/petroleum ether, R_f = 0.55.

^{1}H NMR (300 MHz, $CDCl_3$) δ 7.74 – 7.65 (m, 2H), 7.63 – 6.55 (m, 2H), 7.46 (s, 1H), 4.46 (s, 1H), 4.27 (qd, *J* = 7.1, 1.3 Hz, 4H), 3.15 – 3.00 (m, 2H), 2.51 – 2.38 (m, 2H), 1.57 – 1.37 (m, 2H), 1.40 (s, 9H), 1.25 (t, *J* = 7.1 Hz, 6H), 1.23 – 1.09 (m, 2H).

^{13}C NMR (75 MHz, $CDCl_3$) δ 168.2, 165.2, 156.0, 132.4, 132.1, 128.9, 126.9, 79.3, 66.9, 62.9, 40.4, 32.0, 30.0, 28.5, 21.2, 14.2.

HRMS (ESI/Q-TOF) *m*/*z*: $[M+Na]^+$ Calcd for $C_{23}H_{33}N_2O_7BrNa$ 551.1369; Found 551.1390.

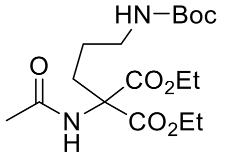

1,3-Diethyl 2-(3-{[(*tert*-butoxy)carbonyl]amino}propyl)-2-acetamidopropanedioate (7i) was obtained from diethyl 2-acetamidomalonate (1.55 g, 7.1 mmol) and alkyl bromide **S3a** (1.87 g, 7.8 mmol) according to the general procedure F. The crude product was purified by reversed phase flash column chromatography using gradient elution from 0% to 100% MeCN in water containing 0.01% TFA to afford 2.38 g, (89%) of the title compound as a colorless amorphous solid; analytical TLC on silica gel, 1:1 EtOAc/petroleum ether, R_f = 0.24.

^{1}H NMR (300 MHz, $CDCl_3$) δ 6.79 (s, 1H), 4.66 (br. s, 1H), 4.18 (q, *J* = 7.1 Hz, 4H), 3.04 (q, *J* = 6.6 Hz, 2H), 2.33 – 2.22 (m, 2H), 1.98 (s, 3H), 1.37 (s, 9H), 1.32 – 1.23 (m, 2H), 1.20 (t, *J* = 7.1 Hz, 6H).

^{13}C NMR (75 MHz, $CDCl_3$) δ 169.1, 168.0, 155.9, 79.1, 66.3, 62.6, 40.2, 29.7, 28.4, 24.3, 23.0, 14.0.

HRMS (ESI/Q-TOF) *m*/*z*: $[M+Na]^+$ Calcd for $C_{17}H_{30}N_2O_7Na$ 397.1951; Found 397.1956.

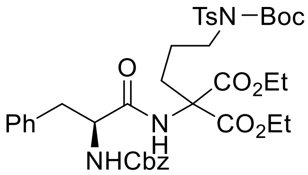

1,3-Diethyl 2-[(2*S*)-2-{[(benzyloxy)carbonyl]amino}-3-phenylpropanamido]-2-(3-{*N*-[(*tert*-butoxy)carbonyl]-4-methylbenzenesulfonamido}propyl)propanedioate (7j) was obtained from malonate **S7f** (970 mg, 2.1 mmol) and alkyl bromide **S2a** (814 mg, 2.1 mmol) according to the general procedure F. The crude product was purified by reversed phase flash column chromatography using gradient elution from 0% to 100% MeCN in water containing 0.01% TFA to afford 396 mg (24%) of the title compound as a colorless semisolid; analytical TLC on silica gel, 1:1 EtOAc/petroleum ether, R_f = 0.50.

^{1}H NMR (300 MHz, $CDCl_3$) δ 7.78 – 7.69 (m, 2H), 7.36 – 7.15 (m, 12H), 5.45 (d, *J* = 8.2 Hz, 1H), 5.13 – 5.00 (m, 2H), 4.61 – 4.44 (m, 1H), 4.32 – 4.14 (m, 4H), 3.81 – 3.56 (m, 2H), 3.18 – 2.98 (m, 2H), 2.42 (s, 3H), 2.41 – 2.30 (m, 2H), 1.56 – 1.40 (m, 2H), 1.31 (s, 9H), 1.30 – 1.17 (m, 6H).

^{13}C NMR (75 MHz, $CDCl_3$) δ 170.0, 167.7, 167.5, 156.1, 150.9, 144.2, 137.4, 136.4, 136.3, 129.6, 129.4, 128.8, 128.6, 128.2, 128.1, 127.9, 127.1, 84.4, 67.1, 66.3, 62.9, 56.0, 46.8, 38.1, 29.3, 27.9, 24.6, 21.7, 14.1.

HRMS (ESI/Q-TOF) *m*/*z*: $[M+Na]^+$ Calcd for $C_{39}H_{49}N_3O_{11}SNa$ 790.2985; Found 790.2978.

$[\alpha]^{20}_D$ –6 (*c* 1.0, $CHCl_3$).

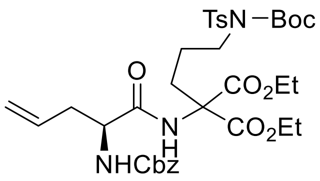

1,3-Diethyl 2-[(2*S*)-2-{[(benzyloxy)carbonyl]amino}pent-4-enamido]-2-(3-{*N*-[(*tert*-butoxy)carbonyl]-4-methylbenzenesulfonamido}propyl)-propanedioate (7k) was obtained from malonate **S8** (1.22 g, 3.0 mmol) and alkyl bromide **S2a** (1.30 g, 3.3 mmol) according to the general procedure F. The crude product was purified by flash column chromatography on silica gel using gradient elution from 25% to 33% EtOAc in petroleum ether and then by the reversed phase flash column chromatography using gradient elution from 5% to 100% MeCN in water containing 0.01% TFA to afford 1.21 g (56%) of the title compound as a white foam; analytical TLC on silica gel, 1:2 EtOAc/petroleum ether, R_f = 0.26.

^{1}H NMR (300 MHz, $CDCl_3$) δ 7.79 – 7.67 (m, 2H), 7.37 (s, 1H), 7.36 – 7.22 (m, 7H), 5.85 – 5.65 (m, 1H), 5.41 (d, *J* = 7.8 Hz, 1H), 5.21 – 5.04 (m, 4H), 4.35 – 4.28 (m, 1H), 4.28 – 4.17 (m, 4H), 3.74 (t, *J* = 7.6 Hz, 2H), 2.53 (t, *J* = 6.7 Hz, 2H), 2.45 – 2.32 (m, 2H), 2.40 (s, 3H), 1.65-1.50 (m, 2H), 1.30 (s, 9H), 1.24 (t, *J* = 7.1 Hz, 3H), 1.23 (t, *J* = 7.1 Hz, 3H).

^{13}C NMR (75 MHz, $CDCl_3$) δ 170.1, 167.6, 167.4, 156.0, 150.9, 144.2, 137.3, 136.3, 132.7, 129.3, 128.5, 128.1, 128.1, 127.8, 119.6, 84.3, 67.1, 66.2, 62.8, 62.8, 54.0, 46.7, 36.8, 29.3, 27.8, 24.7, 21.6, 14.0, 14.0.

HRMS (ESI/Q-TOF) *m*/*z*: $[M+Na]^+$ Calcd for $C_{35}H_{47}N_3O_{11}SNa$ 740.2829; Found 740.2827.

$[\alpha]^{20}_D$ –6 (*c* 1.0, $CHCl_3$).

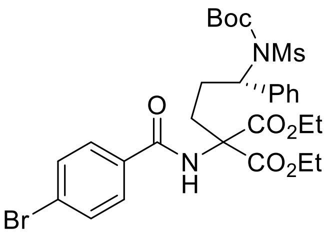

1,3-Diethyl 2-[(4-bromophenyl)formamido]-2-[(3*S*)-3-{*N*-[(*tert*-butoxy) carbonyl]methanesulfonamido}-3-phenylpropyl]propanedioate (7l) was obtained from malonate **S7e** (811 mg, 2.3 mmol) and alkyl bromide **S6** (977 mg, 1.1 mmol) according to the general procedure F. The crude product was purified by reversed phase flash column chromatography using gradient elution from 5% to 100% MeCN in water containing 0.01% TFA and then by flash column chromatography on silica gel using gradient elution from 20% to 50 % EtOAc in petroleum ether to afford 368 mg (24%) of the title compound as a white foam; analytical TLC on silica gel, 1:3 EtOAc/petroleum ether, R_f = 0.29.

^{1}H NMR (300 MHz, $CDCl_3$) δ 7.76 – 7.66 (m, 2H), 7.64 – 7.53 (m, 2H), 7.54 (s, 1H), 7.42 – 7.16 (m, 5H), 5.45 (dd, *J* = 9.7, 5.9 Hz, 1H), 4.39 – 4.15 (m, 4H), 3.26 (s, 3H), 2.70 – 2.46 (m, 2H), 2.38 – 2.18 (m, 1H), 2.12 – 1.94 (m, 1H), 1.40 – 1.19 (m, 15H).

^{13}C NMR (75 MHz, $CDCl_3$) δ 168.0, 167.9, 165.1, 151.2, 139.3, 132.2, 132.0, 128.9, 128.4, 127.6, 127.4, 126.9, 84.8, 66.5, 63.0, 63.0, 59.8, 42.4, 30.0, 27.8, 25.7, 14.1, 14.0.
HRMS (ESI/Q-TOF) *m*/*z*: $[M+Na]^+$ Calcd for $C_{29}H_{37}N_2O_9SBrNa$ 691.1301; Found 691.1313.
$[\alpha]^{20}_D$ –36 (*c* 1.0, $CHCl_3$).

General procedure I for the synthesis of alkylated malonates S8a,b

The reaction was performed in a 25 mL round bottom flask under argon atmosphere by following the reported procedure [8]. The malonate derivative **7i** (1 equiv) was dissolved in anhydrous CH_2Cl_2 (4.6 mL/mmol of malonate derivative) followed by the dropwise addition of TFA (8 equiv). The colorless reaction solution was stirred at room temperature for 2 hours. The reaction solution was cooled in an ice-bath. Et_3N (10 equiv) was slowly added to the reaction solution followed by the addition of *o*-nosyl chloride or benzoyl chloride (1.2 equiv). The colorless reaction solution was stirred at room temperature for one hour. After the reaction was completed, it was washed with brine (15 mL/mmol of malonate). The organic layer was separated, dried over anhydrous Na_2SO_4, filtered and concentrated *in vacuo*. The crude material was purified by using flash column chromatography.

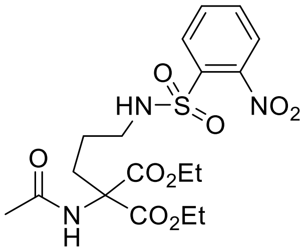

1,3-Diethyl 2-acetamido-2-[3-(2-nitrobenzenesulfonamido)propyl]propanedioate (S8a) was obtained from malonate derivative **7i** (500 mg, 1.3 mmol) according to the general procedure I. The crude material was purified by flash column chromatography on silica gel using isocratic elution with 25% EtOAc in petroleum ether to afford 536 mg (87%) of the title compound as a colorless foam; analytical TLC on silica gel, 3:1 EtOAc/hexane, R_f = 0.21.
^{1}H NMR (300 MHz, $CDCl_3$) δ 8.15 – 8.03 (m, 1H), 7.91 – 7.79 (m, 1H), 7.80 – 7.68 (m, 2H), 6.78 (s, 1H), 5.43 (t, *J* = 6.1 Hz, 1H), 4.22 (q, *J* = 7.3 Hz, 2H), 3.11 – 2.98 (m, 1H), 2.36 – 2.24 (m, 1H), 2.00 (s, 3H), 1.50 – 1.33 (m, 2H), 1.23 (t, *J* = 7.1 Hz, 6H).
^{13}C NMR (75 MHz, $CDCl_3$) δ 169.4, 167.9, 148.2, 133.8, 133.5, 132.9, 131.2, 125.6, 66.1, 62.9, 43.6, 29.6, 24.6, 23.1, 14.1.
HRMS (ESI/Q-TOF) *m*/*z*: $[M+H]^+$ Calcd for $C_{18}H_{26}N_3O_9S$ 460.1403; Found 460.1390.

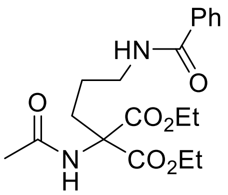

1,3-Diethyl 2-acetamido-2-[3-(phenylformamido)propyl]propanedioate (S8b) was obtained from malonate derivative **7i** (500 mg, 1.3 mmol) according to the general procedure I. The crude material was purified by flash column chromatography on silica gel using isocratic elution with 25% EtOAc in petroleum

ether to afford 413 mg (82%) of the title compound as a white foam; analytical TLC on silica gel, 3:1 EtOAc/hexane, R_f = 0.21.

^{1}H NMR (300 MHz, $CDCl_3$) δ 7.80 – 7.70 (m, 2H), 7.53 – 7.34 (m, 3H), 6.82 (s, 1H), 6.46 (t, *J* = 5.6 Hz, 1H), 4.22 (q, *J* = 7.1 Hz, 4H), 3.42 (q, *J* = 6.6 Hz, 2H), 2.47 – 2.36 (m, 2H), 2.00 (s, 3H), 1.57 – 1.40 (m, 2H), 1.22 (t, *J* = 7.1 Hz, 6H).

^{13}C NMR (75 MHz, $CDCl_3$) δ 169.4, 168.1, 167.6, 134.7, 131.5, 128.7, 127.0, 66.4, 62.8, 39.7, 29.9, 23.9, 23.2, 14.1.

HRMS (ESI/Q-TOF) *m*/*z*: $[M+H]^+$ Calcd for $C_{19}H_{27}N_2O_6$ 379.1877; Found 379.1869.

General procedure J for the synthesis of alkylated malonates S8c,d

Trifluoroacetic acid (19 equiv) was added to a 0.07 M solution of carbamate **7g** or **7h** (1 equiv) in dry CH_2Cl_2 under argon and at rt. The reaction solution was stirred for 2.5 hours. After completion, the solvent was evaporated *in vacuo*, and the resulting crude amine was dissolved in CH_2Cl_2 (5.5 mL/mmol of carbamate) followed by the addition of TsCl (1.3 equiv) and Et_3N (2.5 equiv). The reaction mixture was stirred at room temperature for 18 hours. The colorless reaction solution was concentrated *in vacuo* and the residue was purified by using flash column chromatography.

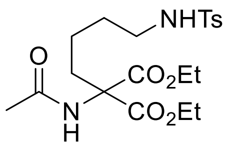

1,3-Diethyl 2-acetamido-2-[4-(4-methylbenzenesulfonamido)butyl]propanedioate (S8c) was obtained from carbamate **7g** (281 mg, 0.72 mmol) according to the general procedure J. The product was purified by flash column chromatography on silica gel using gradient elution from 33% to 50% EtOAc in petroleum ether and then by reversed phase flash column chromatography using gradient elution from 5% to 100% MeCN in water containing 0.01% TFA to afford 182 mg (57%) of the title compound as a thick colorless oil; analytical TLC on silica gel, 1:1 EtOAc/petroleum ether, R_f = 0.18.

^{1}H NMR (300 MHz, $CDCl_3$) δ 7.75 – 7.65 (m, 2H), 7.33 – 7.23 (m, 2H), 6.79 (s, 1H), 4.87 (t, *J* = 6.1 Hz, 1H), 4.28 – 4.12 (m, 4H), 2.86 (q, *J* = 6.8 Hz, 2H), 2.41 (s, 3H), 2.30 – 2.18 (m, 2H), 2.00 (s, 3H), 1.52 – 1.36 (m, 2H), 1.22 (t, *J* = 7.1 Hz, 6H), 1.19 – 1.02 (m, 2H).

^{13}C NMR (75 MHz, $CDCl_3$) δ 169.4, 168.1, 143.4, 137.0, 129.8, 127.1, 66.4, 62.7, 42.9, 31.8, 29.3, 23.1, 21.6, 20.7, 14.1.

HRMS (ESI/Q-TOF) *m*/*z*: $[M+H]^+$ Calcd for $C_{20}H_{30}N_2O_7S$ 443.1852; Found 443.1862.

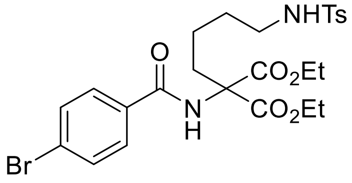

1,3-Diethyl 2-[(4-bromophenyl)formamido]-2-[4-(4-methylbenzene sulfonamido)butyl]propanedioate (S8d) was obtained from carbamate **7h** (630 mg, 1.2 mmol) according to the general procedure J. The crude material was purified by flash column chromatography on silica gel using gradient elution from 10% to 30% EtOAc in petroleum ether to afford 235 mg (34%) of the title compound as a thick colorless oil; analytical TLC on silica gel, 1:2 EtOAc/petroleum ether, R_f = 0.26.

^{1}H NMR (300 MHz, $CDCl_3$) δ 7.73 – 7.63 (m, 4H), 7.64 – 7.54 (m, 2H), 7.44 (s, 1H), 7.32 – 7.23 (m, 2H), 4.39 (t, *J* = 6.4 Hz, 1H), 4.35 – 4.16 (m, 4H), 2.89 (q, *J* = 6.8 Hz, 2H), 2.41 (s, 3H), 2.44 – 2.33 (m, 2H), 1.48 (p, *J* = 7.2 Hz, 2H), 1.25 (t, *J* = 7.0 Hz, 6H), 1.24 – 1.10 (m, 2H).

^{13}C NMR (75 MHz, $CDCl_3$) δ 168.1, 165.3, 143.6, 137.0, 132.3, 132.1, 129.9, 128.9, 127.2, 127.0, 66.8, 63.0, 43.0, 31.8, 29.4, 21.7, 20.9, 14.1.

HRMS (ESI/Q-TOF) *m*/*z*: $[M+H]^+$ Calcd for $C_{25}H_{32}N_2O_7SBr$ 583.1114; Found 583.1132.

General procedure G for the synthesis of acids 8a–i and 9g–k via malonate hydrolysis

Malonate derivative (1 equiv) was dissolved in EtOH (2 mL/1 mmol of malonate derivatives **7a**–**j**,**l** and **S8a**–**c** or 4 mL/1 mmol of malonate derivative **7k** and **S8d**) followed by the addition of KOH (1–3 equiv) in H_2O (2 mL/ 1 mmol of malonate derivative). The colorless emulsion was stirred at room temperature. Reaction progress was monitored by LC/MS; usually, the reaction takes 1–3 hours. Upon completion, the clear (colorless or light yellow) reaction solution was treated with 1 M HCl to pH 3–4 and EtOH was removed *in vacuo*. The remained aqueous phase was diluted with H_2O (5 mL/mmol of malonate derivative) and extracted with EtOAc (4 mL/mmol of malonate derivative). Organic layers were combined, washed with brine (5 mL/mmol of malonate derivative), dried over anhydrous Na_2SO_4, filtered and concentrated *in vacuo*. The obtained product was used in the next step without purification or it was purified by using reversed phase flash column chromatography using gradient elution from 0% to 100% MeCN in water containing 0.01% TFA.

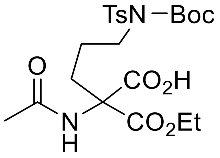

5-{*N*-[(*Tert*-butoxy)carbonyl]-4-methylbenzenesulfonamido}-2-acetamido-2-(ethoxycarbonyl)pentanoic acid (8a) was obtained as a colorless thick oil (1.22 g, 95%) from malonate **7a** (1.36 g, 2.6 mmol) in presence of KOH (1.4 equiv) according to general procedure G. The obtained product was used in the next step without additional purification.

^{1}H NMR (300 MHz, $CDCl_3$) δ 9.36 (br. s, 1H), 7.78 – 7.70 (m, 2H), 7.33 – 7.26 (m, 2H), 7.10 (s, 1H), 4.35 – 4.21 (m, 2H), 3.77 (t, *J* = 7.5 Hz, 2H), 2.43 (s, 3H), 2.40 – 2.29 (m, 2H), 2.10 (s, 3H), 1.76 – 1.54 (m, 1H), 1.32 (s, 9H), 1.25c (t, *J* = 7.1 Hz, 3H).

^{13}C NMR (75 MHz, $CDCl_3$) δ 171.5, 169.2, 168.5, 151.0, 144.4, 137.3, 129.4, 127.9, 84.5, 66.3, 63.2, 46.8, 29.7, 28.0, 24.9, 23.0, 21.7, 14.0.

HRMS (ESI/Q-TOF) *m*/*z*: $[M+Na]^+$ Calcd for $C_{22}H_{32}N_2O_9SNa$ 523.1726; Found 523.1740.

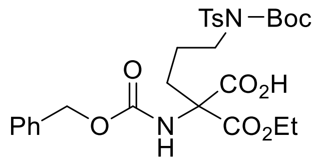

2-{[(Benzyloxy)carbonyl]amino}-5-{*N*-[(*tert*-butoxy)carbonyl]-4-methylbenzenesulfonamido}-2-(ethoxycarbonyl)pentanoic acid (8b) was obtained from malonate **7b** (980 mg, 1.6 mmol) in presence of KOH (1.3 equiv) according to general procedure G. The crude product was purified by reversed phase flash column chromatography using gradient elution from 5% to 100% MeCN in water containing 0.01% TFA to afford 733 mg (78 %) of the title compound as a thick colorless oil.

^{1}H NMR (300 MHz, $CDCl_3$) δ 7.80 – 7.70 (m, 2H), 7.39 – 7.24 (m, 7H), 6.22 (s, 1H), 5.12 (s, 2H), 4.28 (q, *J* = 6.7 Hz, 2H), 3.80 (t, *J* = 7.1 Hz, 2H), 2.42 (s, 3H), 2.40 – 2.25 (m, 2H), 1.93 – 1.60 (m, 2H), 1.32 (s, 9H), 1.26 (t, *J* = 7.1 Hz, 3H).

^{13}C NMR (75 MHz, $CDCl_3$) δ 170.2, 168.3, 155.0, 150.9, 144.2, 137.2, 135.9, 129.3, 128.6, 128.3, 128.1, 127.9, 84.4, 67.4, 66.2, 63.3, 46.6, 30.2, 27.9, 24.6, 21.6, 13.9.

HRMS (ESI/Q-TOF) *m*/*z*: $[M+Na]^+$ Calcd for $C_{28}H_{36}N_2O_{10}SNa$ 515.1464; Found 515.1475.

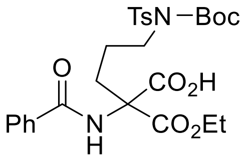

5-{*N*-[(*Tert*-butoxy)carbonyl]-4-methylbenzenesulfonamido}-2-(ethoxycarbonyl)-2-(phenylformamido)pentanoic acid (8c) was obtained from malonate **7c** (853 mg, 1.4 mmol) in presence of KOH (1.3 equiv) according to general procedure G. The crude product was purified by reversed phase flash column chromatography using gradient elution from 5% to 100% MeCN in water containing 0.01% TFA to afford 607 mg (75%) of the title compound as a colorless semisolid.

^{1}H NMR (300 MHz, $CDCl_3$) δ 10.98 (s, 1H), 7.90 – 7.80 (m, 2H), 7.77 – 7.66 (m, 2H), 7.66 (s, 1H), 7.59 – 7.47 (m, 1H), 7.44 (t, *J* = 7.4 Hz, 2H), 7.27 – 7.18 (m, 2H), 4.31 (q, *J* = 7.1 Hz, 2H), 3.79 (t, *J* = 7.5 Hz, 2H), 2.62 – 2.40 (m, 2H), 2.38 (s, 3H), 1.84 – 1.56 (m, 2H), 1.28 (s, 9H), 1.28 (t, *J* = 7.1 Hz, 3H).

^{13}C NMR (75 MHz, $CDCl_3$) δ 170.0, 168.4, 167.4, 151.0, 144.3, 137.1, 132.9, 132.4, 129.4, 128.8, 127.9, 127.5, 84.5, 66.5, 63.4, 46.8, 29.7, 27.9, 25.0, 21.7, 14.0.

HRMS (ESI/Q-TOF) *m*/*z*: $[M+Na]^+$ Calcd for $C_{27}H_{34}N_2O_9SNa$ 585.1883; Found 585.1907.

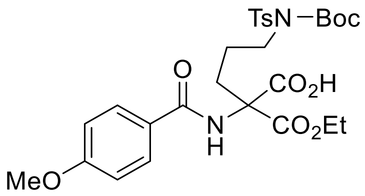

5-{*N*-[(*Tert*-Butoxy)carbonyl]-4-methylbenzenesulfonamido}-2-(ethoxycarbonyl)-2-[(4-methoxyphenyl)formamido]pentanoic acid (8d) was obtained from malonic acid monoester **7d** (690 mg, 1.1 mmol) in presence of KOH (1.3 equiv) according to general procedure G. The crude product was purified by reversed phase flash column chromatography using gradient elution from 5% to 100% MeCN in water containing 0.01% TFA to afford 355 mg (54%) of the title compound as a white amorphous solid.

^{1}H NMR (300 MHz, $CDCl_3$) δ 7.88 – 7.77 (m, 2H), 7.78 – 7.67 (m, 2H), 7.51 (s, 1H), 7.27 – 7.22 (m, 2H), 6.99 – 6.88 (m, 2H), 4.41 – 4.25 (m, 2H), 3.85 (s, 3H), 3.80 (t, *J* = 7.4 Hz, 2H), 2.61 – 2.41 (m, 2H), 2.40 (s, 3H), 1.29 – 1.60 (m, 2H), 1.30 (t, *J* = 7.1 Hz, 3H), 1.29 (s, 9H).

^{13}C NMR (75 MHz, $CDCl_3$) δ 169.6, 168.9, 167.3, 163.1, 151.0, 144.3, 137.2, 129.6, 129.4, 128.0, 125.0, 114.1, 84.5, 66.6, 63.4, 55.6, 46.8, 30.0, 27.9, 25.0, 21.7, 14.1.

HRMS (ESI/Q-TOF) *m*/*z*: $[M+Na]^+$ Calcd for $C_{28}H_{36}N_2O_7SNa$ 615.1988; Found 615.2000.

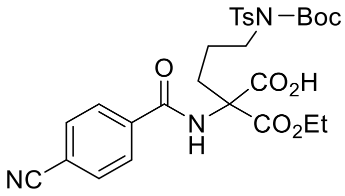

5-{*N*-[(*Tert*-butoxy)carbonyl]-4-methylbenzenesulfonamido}-2-[(4-cyanophenyl)formamido]-2-(ethoxycarbonyl)pentanoic acid (8e) was obtained from malonic acid monoester **7e** (343 mg, 0.6 mmol) in presence of KOH (1.5 equiv) according to the general procedure G. The crude product was purified by reversed phase flash column chromatography using gradient elution from 5% to 100% MeCN in water containing 0.01% TFA to afford 201 mg (61%) of the title compound as a white semisolid.

^{1}H NMR (300 MHz, $CDCl_3$) δ 8.24 (br. s, 1H), 8.01 – 7.91 (m, 2H), 7.79 – 7.70 (m, 2H), 7.75 – 7.66 (m, 3H), 7.29 – 7.23 (m, 2H), 4.41 – 4.25 (m, 2H), 3.79 (t, *J* = 7.4 Hz, 2H), 2.57 – 2.44 (m, 2H), 2.41 (s, 3H), 1.82 – 1.61 (m, 2H), 1.29 (t, *J* = 7.1 Hz, 3H), 1.28 (s, 9H).

^{13}C NMR (75 MHz, $CDCl_3$) δ 169.8, 168.3, 165.4, 151.0, 144.5, 137.2, 137.0, 132.6, 129.4, 128.2, 127.9, 118.0, 115.8, 84.7, 66.6, 63.6, 46.8, 29.6, 27.9, 25.1, 21.7, 14.0.

HRMS (ESI/Q-TOF) *m*/*z*: $[M+Na]^+$ Calcd for $C_{28}H_{33}N_3O_9SNa$ 610.1835; Found 610.1850.

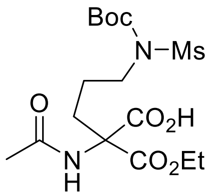

5-{*N*-[(*Tert*-butoxy)carbonyl]methanesulfonamido}-2-acetamido-2-(ethoxycarbonyl)pentanoic acid (8f) was obtained as a white foam (427 mg, 91%) from malonic acid monoester **7f** (498 mg, 1.1 mmol) in presence of KOH (1.3 equiv) according to the general procedure G. The product was used in the next step without additional purification.

^{1}H NMR (300 MHz, $CDCl_3$) δ 10.04 (s, 1H), 7.04 (s, 1H), 4.26 (q, *J* = 7.1 Hz, 2H), 3.66 (t, *J* = 7.2 Hz, 2H), 3.27 (s, 3H), 2.39 – 2.21 (m, 2H), 2.09 (s, 3H), 1.61 – 1.43 (m, 2H), 1.53 (s, 9H), 1.27 (t, *J* = 7.1 Hz, 3H).

^{13}C NMR (75 MHz, $CDCl_3$) δ 171.5, 169.1, 168.3, 151.6, 85.0, 66.3, 63.3, 46.0, 42.4, 29.6, 28.1, 24.4, 23.0, 14.0.

HRMS (ESI/Q-TOF) *m*/*z*: $[M+Na]^+$ Calcd for $C_{16}H_{28}N_2O_9SNa$ 447.1413; Found 447.1427.

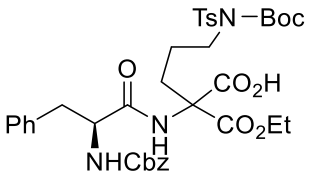

2-[(2*S*)-2-{[(Benzyloxy)carbonyl]amino}-3-phenylpropanamido]-5-{*N*-[(*tert*-butoxy)carbonyl]-4-methylbenzenesulfonamido}-2-(ethoxycarbonyl)pentanoic acid (8g) was obtained from malonic acid monoester **7j** (360 mg, 0.47 mmol) in presence of KOH (1.5 equiv) according to the general procedure G. The crude product was purified by reversed phase flash column chromatography using gradient elution from 5% to 100% MeCN in water containing 0.01% TFA to afford 203 mg (59%) of the title compound as a white semisolid.

^{1}H NMR (300 MHz, $CDCl_3$) (mixture of diastereomers 59:43) δ 9.15 (s, 1H), 7.78 – 7.71 (m, 2H), 7.62 (s, 0.4H), 7.57 (s, X0.6H), 7.36 – 7.14 (m, 12H), 5.79 (d, *J* = 8.7 Hz, 1H), 5.14 – 4.91 (m, 2H), 4.86 – 4.73 (m, 0.57H), 4.72 – 4.61 (m, 0.43H), 4.33 – 4.07 (m, 2H), 3.82 – 3.63 (m, 2H), 3.23 – 3.07 (m, 1H), 3.08 – 2.90 (m, 1H), 2.45 – 2.30 (m, 4H), 1.70 – 1.41 (m, 2H), 1.31 (s, 9H), 1.28 – 1.11 (m, 6H).

^{13}C NMR (75 MHz, $CDCl_3$) (mixture of diastereomers 59:43) δ 171.5, 169.5, 169.4, 167.8, 156.5, 151.0, 144.2, 137.3, 136.4, 136.3, 136.1, 129.5, 129.4, 128.8, 128.7, 128.6, 128.5, 128.2, 128.1, 128.0, 127.1, 127.1, 84.5, 67.3, 66.3, 63.2, 63.0, 55.8, 46.7, 38.5, 29.7, 27.9, 24.6, 21.7, 14.0, 13.9.

HRMS (ESI/Q-TOF) *m*/*z*: $[M+Na]^+$ Calcd for $C_{37}H_{45}N_3O_{11}SNa$ 762.2672; Found 762.2687.

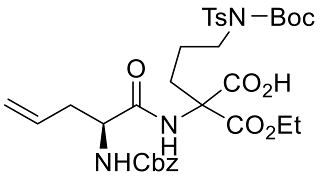

2-[(2*S*)-2-{[(Benzyloxy)carbonyl]amino}pent-4-enamido]-5-{*N*-[(*tert*-butoxy)carbonyl]-4-methylbenzenesulfonamido}-2-(ethoxycarbonyl) pentanoic acid (8h) was obtained from malonic acid monoester **7k** (1.21 g, 1.7 mmol) in presence of KOH (1.5 equiv) according to the general procedure G. The crude product was purified by reversed phase flash column chromatography using gradient elution from 5% to 100% MeCN in water containing 0.01% TFA to afford 1.16 g (99%) of the title compound as a white foam.

^{1}H NMR (300 MHz, $CDCl_3$) (mixture of diastereomers 1:1) δ 9.59 (br. s, 1H), 7.78-7.74 (m, 1H), 7.74-7.70 (m, 1H), 7.63 (br. s, 1H), 7.37 – 7.23 (m, 7H), 5.88 – 5.57 (m, 2H), 5.21-5.00 (m, 4H), 4.59-4.48 (m, 0.5H), 4.48-4.37 (m, 0.5H), 4.34 – 4.06 (m, 2H), 3.88 – 3.66 (m, 2H), 2.64 – 2.44 (m, 2H), 2.45 – 2.31 (m, 5H), 1.77 – 1.55 (m, 2H), 1.31 (s, 9H), 1.26 (t, *J* = 7.2 Hz, 1.5H), 1.18 (t, *J* = 7.2 Hz, 1.5H).

^{13}C NMR (75 MHz, $CDCl_3$) δ 171.6, 171.5, 169.4, 169.2, 168.0, 168.0, 156.6, 151.0, 144.3, 137.3, 136.0, 132.7, 132.5, 129.4, 128.6, 128.3, 128.2, 128.0, 119.8, 84.5, 67.5, 66.2, 63.3, 63.0, 54.0, 46.8, 37.1, 29.8, 28.0, 24.9, 24.7, 21.7, 14.0, 13.9.

HRMS (ESI/Q-TOF) *m*/*z*: $[M+Na]^+$ Calcd for $C_{33}H_{43}N_3O_{11}SNa$ 712.2516; Found 712.2535.

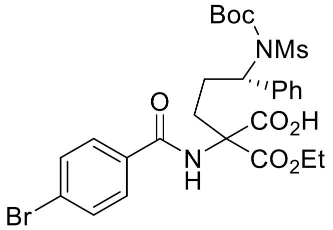

(5*S*)-2-[(4-Bromophenyl)formamido]-5-{*N*-[(*tert*-butoxy)carbonyl]methanesulfonamido}-2-(ethoxycarbonyl)-5-phenylpentanoic acid (8i) was obtained as a white amorphous solid (216 mg, 64%) from malonic acid monoester **7l** (353 mg, 0.5 mmol) in presence of KOH (1.5 equiv) according to the general procedure G. The crude product was used in the next step after extraction without additional purification.

^{1}H NMR (300 MHz, $CDCl_3$) (mixture of diastereomers 38:62) δ 7.77 – 7.69 (m, 2H), 7.63 – 7.54 (m, 3H), 7.36 – 7.22 (m, 5H), 5.51 – 5.38 (m, 1H), 4.39 – 4.25 (m, 2H), 3.24 (s, 1.15H), 3.19 (s, 1.85H), 2.67 – 2.30 (m, 3H), 2.24 – 1.97 (m, 1H), 1.37 – 1.23 (m, 12H).

^{13}C NMR (75 MHz, $CDCl_3$) δ (mixture of diastereomers 38:62) 169.5, 169.0, 168.8, 166.8, 166.6, 165.4, 151.6, 151.4, 139.2, 139.0, 132.1, 131.5, 131.5, 129.1, 128.6, 128.6, 128.0, 127.5, 127.4, 127.4, 85.3, 85.3, 66.4, 66.3, 63.7, 63.7, 60.2, 42.5, 30.7, 30.5, 27.9, 27.8, 26.2, 25.9, 14.1, 14.0.

HRMS (ESI/Q-TOF) *m*/*z*: $[M+Na]^+$ Calcd for $C_{27}H_{33}N_2O_9SBrNa$ 663.0988; Found 663.0994.

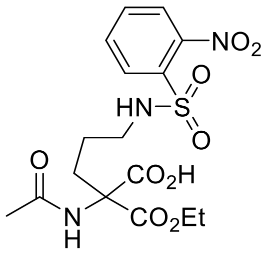

2-Acetamido-2-(ethoxycarbonyl)-5-(2-nitrobenzenesulfonamido)pentanoic acid (9g) was obtained as a white amorphous solid (361mg, 72%) from malonate **S8a** (536 mg, 1.2 mmol) according to the general procedure G. The product was used in the next step without additional purification.

^{1}H NMR (300 MHz, CD_3OD) δ 8.12 – 8.00 (m, 1H), 7.89 – 7.74 (m, 3H), 4.18 (q, *J* = 7.1 Hz, 1H), 3.04 (t, *J* = 7.1 Hz, 1H), 2.35 – 2.12 (m, 2H), 1.98 (s, 3H), 1.51 – 1.31 (m, 2H), 1.22 (t, *J* = 7.1 Hz, 3H).

^{13}C NMR (75 MHz, CD_3OD) δ 172.4, 170.3, 169.5, 149.6, 134.9, 134.9, 133.6, 131.5, 125.9, 67.6, 63.2, 44.1, 31.0, 25.7, 22.4, 14.2.

HRMS (ESI/Q-TOF) *m*/*z*: $[M+H]^+$ Calcd for $C_{16}H_{22}N_3O_9S$ 432.1086; Found 432.1077.

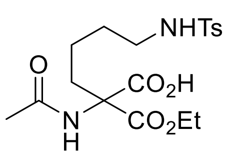

2-Acetamido-2-(ethoxycarbonyl)-6-(4-methylbenzenesulfonamido)hexanoic acid (9h) was obtained from malonic acid monoester **S8c** (178 mg, 0.4 mmol) in presence of KOH (3.0 equiv) according to the general procedure G. The crude product was purified by reversed phase flash column chromatography using gradient elution from 5%

to 100% MeCN in water containing 0.01% TFA to afford 132 mg (79%) of the title compound as a white foam.

^{1}H NMR (300 MHz, $CDCl_3$) δ 9.52 (s, 1H), 7.76 – 7.66 (m, 2H), 7.33 – 7.27 (m, 2H), 7.26 (s, 1H), 5.31 (s, 1H), 4.24 (q, *J* = 7.1 Hz, 2H), 3.02 –2.81 (m, 2H), 2.42 (s, 3H), 2.24 (t, *J* = 8.5 Hz, 2H), 2.07 (s, 3H), 1.55 – 1.38 (m, 2H), 1.32 – 1.17 (m, 2H), 1.25 (t, *J* = 7.1 Hz, 3H).

^{13}C NMR (75 MHz, $CDCl_3$) δ 171.7, 169.7, 168.5, 143.6, 136.9, 129.9, 127.2, 66.6, 63.0, 42.6, 32.1, 29.0, 22.8, 21.7, 20.6, 14.1.

HRMS (ESI/Q-TOF) *m*/*z*: $[M+H]^+$ Calcd for $C_{18}H_{27}N_2O_7S$ 415.1539; Found 415.1552.

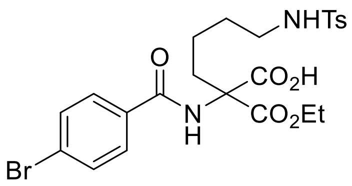

2-[(4-Bromophenyl)formamido]-2-(ethoxycarbonyl)-6-(4-methylbenzenesulfonamido)hexanoic acid (9i) was obtained from malonic acid monoester **S8d** (235 mg, 0.4 mmol) in presence of KOH (1.5 equiv) according to the general procedure G. The crude product was purified by reversed phase flash column chromatography using gradient elution from 5% to 100% MeCN in water containing 0.01% TFA to afford 206 mg (92%) of the title compound as a colorless amorphous solid.

^{1}H NMR (300 MHz, $CDCl_3$) δ 7.76 – 7.65 (m, 4H), 7.64 – 7.55 (m, 3H), 7.31 – 7.23 (m, 2H), 4.92 (br. s, 1H), 4.31 (qd, *J* = 7.1, 2.1 Hz, 2H), 3.04 – 2.84 (m, 2H), 2.49 – 2.25 (m, 2H), 2.44 (s, 3H), 1.61 – 1.35 (m, 3H), 1.35 – 1.19 (m, 1H), 1.28 (t, *J* = 7.1 Hz, 3H).

^{13}C NMR (75 MHz, $CDCl_3$) δ 169.6, 168.8, 166.8, 143.7, 136.8, 132.1, 131.6, 129.9, 129.2, 127.4, 127.1, 66.7, 63.4, 42.4, 32.2, 29.0, 21.7, 20.4, 14.1.

HRMS (ESI/Q-TOF) *m*/*z*: $[M+H]^+$ Calcd for $C_{23}H_{28}N_2O_7SBr$ 555.0801; Found 555.0824.

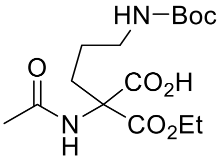

5-{[(*Tert*-butoxy)carbonyl]amino}-2-acetamido-2-(ethoxycarbonyl)pentanoic acid (9j) was obtained as a white amorphous solid (446 mg, 96%) from malonic acid monoester **7i** (500 mg, 0.4 mmol) in presence of KOH (1.8 equiv) according to the general procedure G. The product was used in the next step after extraction without additional purification.

^{1}H NMR (300 MHz, $CDCl_3$) (2:3 mixture of rotamers) δ 11.30 (br. s, 1H), 7.23 (s, 0.7H), 7.06 (s,0.3H), 6.24 (s, 0.4H), 4.86 (s, 0.6H), 4.23 (q, *J* = 7.1 Hz, 2H), 3.16 – 3.00 (m, 2H), 2.41 – 2.24 (s, 2H), 2.06 (s, 3H), 1.50 – 1.32 (m, 11H), 1.25 (t, *J* = 7.1 Hz, 3H).

^{13}C NMR (75 MHz, $CDCl_3$) (2:3 mixture of rotamers) δ 171.0, 169.9, 168.4, 160.0, 158.6, 156.5, 81.7, 79.8, 66.5, 62.8, 62.7, 41.0, 40.3, 29.8, 28.5, 24.5, 22.9, 14.1.

HRMS (ESI/Q-TOF) *m*/*z*: $[M+Na]^+$ Calcd for $C_{16}H_{22}N_6O_3Na$ 369.1646; Found 369.1651.

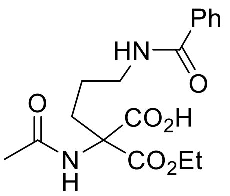

2-Acetamido-2-(ethoxycarbonyl)-5-(phenylformamido)pentanoic acid (9k) was obtained as a white foam (294 mg, 73%) from malonate **S8b** (413mg, 1.1 mmol) in presence of KOH (1.6 equiv) according to the general procedure G. The product was used in the next step without additional purification.

^{1}H NMR (300 MHz, $CDCl_3$) δ 11.46 (s, 1H), 7.78 – 7.69 (m, 2H), 7.50 – 7.38 (m, 1H), 7.41 – 7.28 (m, 3H), 7.20 (t, J = 5.9 Hz, 1H), 4.18 (q, J = 7.1 Hz, 2H), 3.39 (q, J = 6.6 Hz, 2H), 2.48 – 2.25 (m, 2H), 1.99 (s, 3H), 1.52 (p, J = 7.7 Hz, 2H), 1.19 (t, J = 7.1 Hz, 3H).

^{13}C NMR (75 MHz, $CDCl_3$) δ 171.2, 169.3, 168.7, 168.4, 134.0, 131.8, 128.7, 127.2, 66.5, 62.8, 40.0, 30.1, 23.9, 22.9, 14.0.

HRMS (ESI/Q-TOF) m/z: $[M+H]^+$ Calcd for $C_{17}H_{23}N_2O_6$ 351.1559; Found 351.1556.

General procedure H for the synthesis of compounds 9a–f,l–n by cleavage of Boc group

N-Boc-protected sulfonamide **9a**–**e**,**l**–**n** (1 equiv) was dissolved in CH_2Cl_2 (2.5 mL/mmol of compound **9a**,**c**,**d** or 14 mL/mmol of compound **9b**,**e**,**j**–**l**) followed by the dropwise addition of TFA (6 equiv for compounds **9a**,**c**,**d** or 19 equiv for compounds **9b**,**e**,**l**,**m**,**n**). The colorless reaction solution was stirred at room temperature for 16 hours (for compounds **9a**,**c**,**d**) or for 3 hours (for compounds **9b**,**e**,**j**–**l**). After completion, the reaction solution was concentrated *in vacuo*, and the residue was purified by reversed phase flash column chromatography using gradient elution from 0% to 100% MeCN in water containing 0.01% TFA.

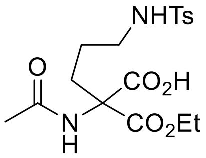

2-Acetamido-2-(ethoxycarbonyl)-5-(4-methylbenzenesulfonamido)pentanoic acid (9a) was obtained as a white amorphous solid (1.08 g, 81%) from malonic acid monoester **8a** (1.36 g, 3.4 mmol) according to general procedure H.

^{1}H NMR (300 MHz, $CDCl_3$) δ 10.45 (br. s, 1H), 7.74 – 7.65 (m, 2H), 7.37 (s, 1H), 7.33 – 7.24 (m, 2H), 5.52 (s, 1H), 4.22 (q, J = 7.1 Hz, 2H), 2.94 – 2.83 (m, 2H), 2.41 (s, 3H), 2.38 – 2.22 (m, 2H), 2.04 (s, 3H), 1.49 – 1.33 (m, 2H), 1.23 (t, J = 7.1 Hz, 3H).

^{13}C NMR (75 MHz, DMSO-d_6) δ 169.1, 168.9, 168.0, 142.6, 137.6, 129.6, 126.5, 65.8, 61.2, 42.6, 30.0, 24.0, 22.2, 21.0, 13.9.

HRMS (ESI/Q-TOF) m/z: $[M+H]^+$ Calcd for $C_{17}H_{25}N_2O_7S$ 401.1382; Found 401.1375.

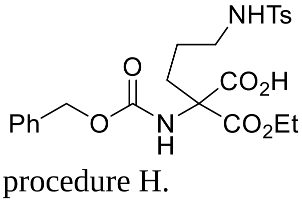

2-{[(Benzyloxy)carbonyl]amino}-2-(ethoxycarbonyl)-5-(4-methylbenzene sulfonamido)pentanoic acid (9b) was obtained as white foam (783 mg, 69%) from malonic acid monoester **8b** (1.37 g, 2.3 mmol) according to the general procedure H.

^{1}H NMR (300 MHz, DMSO-d_6) δ 7.69 – 7.61 (m, 2H), 7.54 (t, *J* = 6.0 Hz, 1H), 7.42 – 7.26 (m, 7H), 7.20 (s, 1H), 5.07 (d, *J* = 12.8 Hz, 1H), 5.00 (d, *J* = 12.8 Hz, 1H), 4.08 (q, *J* = 7.1 Hz, 2H), 2.66 (q, *J* = 6.7 Hz, 2H), 2.37 (s, 3H), 2.07 (t, *J* = 8.4 Hz, 2H), 1.39 – 1.17 (m, 2H), 1.09 (t, *J* = 7.1 Hz, 3H).

^{13}C NMR (75 MHz, DMSO-d_6) δ 168.8, 167.9, 154.3, 142.6, 137.5, 136.8, 129.6, 128.4, 127.8, 127.5, 126.5, 66.0, 65.6, 61.5, 42.5, 30.4, 23.8, 21.0, 13.8.

HRMS (ESI/Q-TOF) *m*/*z*: $[M+Na]^+$ Calcd for $C_{23}H_{28}N_2O_8SNa$ 615.1988; Found 615.1996.

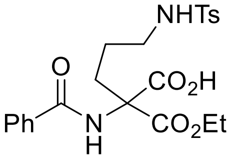

2-(Ethoxycarbonyl)-5-(4-methylbenzenesulfonamido)-2-(phenylformamido) pentanoic acid (9c) was obtained as a white amorphous solid (262 mg, 53%) from malonic acid monoester **8c** (600 mg, 1.1 mmol) according to general procedure H.

^{1}H NMR (300 MHz, $CDCl_3$) δ 9.04 (s, 1H), 7.84 – 7.74 (m, 2H), 7.71 – 7.61 (m, 3H), 7.58 – 7.47 (m, 1H), 7.48 – 7.36 (m, 2H), 7.27 – 7.16 (m, 2H), 5.41 (s, 1H), 4.26 (qd, *J* = 7.1, 1.7 Hz, 2H), 2.91 (t, *J* = 6.6 Hz, 2H), 2.52 – 2.39 (m, 2H), 2.35 (s, 3H), 1.54 – 1.37 (m, 2H), 1.24 (t, *J* = 7.1 Hz, 3H).

^{13}C NMR (75 MHz, $CDCl_3$) δ 169.8, 168.5, 167.6, 143.6, 136.6, 132.7, 132.5, 129.8, 128.9, 127.5, 127.2, 66.4, 63.3, 42.8, 30.1, 24.2, 21.6, 14.0.

HRMS (ESI/Q-TOF) *m*/*z*: $[M+H]^+$ Calcd for $C_{22}H_{27}N_2O_7S$ 463.1539; Found 463.1550.

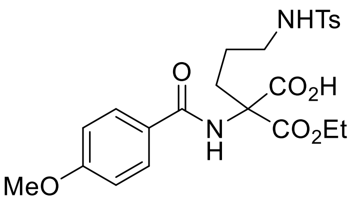

2-(Ethoxycarbonyl)-2-[(4-methoxyphenyl)formamido]-5-(4-methyl benzenesulfonamido)pentanoic acid (9d) was obtained as a white amorphous solid (207 mg, 65%) from malonic acid monoester **8d** (382 mg, 0.6 mmol) according to the general procedure H.

^{1}H NMR (300 MHz, $CDCl_3$) δ 8.36 (br. s, 1H), 7.81 – 7.72 (m, 2H), 7.71 – 7.64 (m, 2H), 7.56 (s, 1H), 7.25 – 7.19 (m, 2H), 6.94 – 6.86 (m, 2H), 5.33 (s, 1H), 4.33 – 4.18 (m, 2H), 3.84 (s, 3H), 2.96 – 2.86 (m, 2H), 2.49 – 2.40 (m, 2H), 2.37 (s, 3H), 1.55 – 1.39 (m, 2H), 1.24 (t, *J* = 7.1 Hz, 3H).

^{13}C NMR (75 MHz, $CDCl_3$) δ 169.7, 168.9, 167.3, 163.1, 143.7, 136.5, 129.9, 129.5, 127.2, 124.8, 114.1, 66.5, 63.3, 55.6, 42.8, 30.2, 24.2, 21.6, 14.1.

HRMS (ESI/Q-TOF) *m*/*z*: $[M+H]^+$ Calcd for $C_{23}H_{29}N_2O_8S$ 493.1645; Found 493.1655.

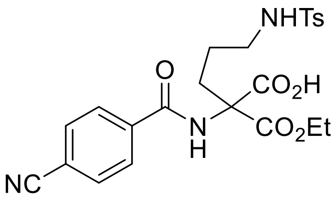

2-[(4-Cyanophenyl)formamido]-2-(ethoxycarbonyl)-5-(4-methylben zenesulfonamido)pentanoic acid (9e) was obtained as a white amorphous solid (73 mg, 80%) from malonic acid monoester **8e** (110 mg, 0.2 mmol) according to the general procedure H.

^{1}H NMR (300 MHz, $CDCl_3$) δ 7.95 – 7.89 (m, 2H), 7.78 (s, 1H), 7.76 – 7.63 (m, 2H), 7.31 – 7.22 (m, 2H), 5.42 (s, 1H), 4.29 (q, *J* = 7.1 Hz, 2H), 2.98 – 2.85 (m, 2H), 2.54 – 2.42 (m, 2H), 2.40 (s, 3H), 1.59 – 1.45 (m, 2H), 1.26 (t, *J* = 7.1 Hz, 3H).

^{13}C NMR (75 MHz, $CDCl_3$) δ 169.6, 168.5, 165.7, 143.8, 136.7, 136.3, 132.6, 129.9, 128.2, 127.1, 118.0, 115.8, 66.4, 63.5, 42.7, 30.1, 24.2, 21.6, 14.0.

HRMS (ESI/Q-TOF) *m*/*z*: $[M+H]^+$ Calcd for $C_{23}H_{26}N_3O_7S$ 488.1491; Found 488.1500.

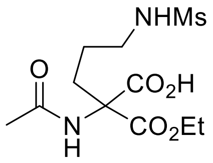

2-Acetamido-2-(ethoxycarbonyl)-5-methanesulfonamidopentanoic acid (9f). *N*-Boc-protected sulfonamide **8f** (617 mg, 1.4 mmol) was dissolved in neat TFA (2 mL), and the reaction solution was stirred at room temperature for 1 hour. Then, TFA was removed *in vacuo*. The residue was dissolved in CH_2Cl_2 (4 mL), and the solvent was removed in *vacuo*. The dissolution/evaporation procedure was repeated for three times to afford the title compound as a white amorphous solid (423 mg, 90%) that was used in the next step without additional purification.

^{1}H NMR (300 MHz, CD_3OD) δ 4.20 (q, *J* = 7.1 Hz, 2H), 3.05 (t, *J* = 6.9 Hz, 2H), 2.91 (s, 3H), 2.43 – 2.20 (m, 2H), 2.01 (s, 3H), 1.57 – 1.34 (m, 2H), 1.24 (t, *J* = 7.1 Hz, 3H).

^{13}C NMR (75 MHz, CD_3OD) δ 172.5, 170.4, 169.6, 67.7, 63.2, 43.8, 39.8, 31.1, 25.9, 22.4, 14.3.

HRMS (ESI/Q-TOF) *m*/*z*: $[M+H]^+$ Calcd for $C_{11}H_{21}N_2O_7S$ 325.1069; Found 325.1071.

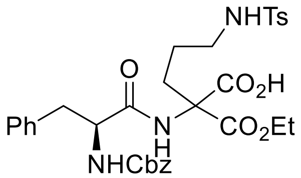

2-[(2*S*)-2-{[(Benzyloxy)carbonyl]amino}-3-phenylpropanamido]-2-(ethoxycarbonyl)-5-(4-methylbenzenesulfonamido)pentanoic acid (9l) was obtained as a colorless amorphous solid (153 mg, 87%) from malonic acid monoester **8g** (203 g, 0.27 mmol) according to the general procedure H.

^{1}H NMR (300 MHz, $CDCl_3$) (mixture of diastereomers 54:46) δ 8.15 (br. s, 1H), 7.76 – 7.73 (m, 2H), 7.50 (s, 1H), 7.39 – 7.02 (m, 12H), 5.90 – 5.65 (m, 1H), 5.65 – 5.27 (m, 1H), 5.10 – 4.91 (m, 2H), 4.79 – 4.52 (m, 1H), 4.29 – 4.05 (m, 2H), 3.25 – 2.88 (m, 2H), 2.85 – 2.68 (m, 2H), 2.39 (s, 3H), 2.34 – 2.15 (m, 2H), 1.34 – 1.05 (m, 5H).

^{13}C NMR (75 MHz, $CDCl_3$) (mixture of diastereomers 54:46) δ 169.5, 169.4, 167.9, 167.6, 156.7, 143.6, 143.5, 136.6, 136.2, 136.1, 129.9, 129.5, 128.8, 128.8, 128.6, 128.3, 128.0, 127.2, 127.1, 67.4, 66.1, 63.1, 56.1, 42.8, 38.1, 29.9, 23.5, 21.6, 14.1, 14.0.

HRMS (ESI/Q-TOF) *m*/*z*: $[M+H]^+$ Calcd for $C_{32}H_{38}N_3O_9S$ 640.2329; Found 640.2360.

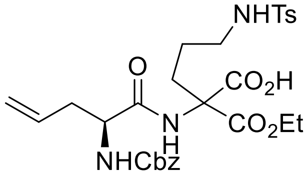

2-[(2*S*)-2-{[(Benzyloxy)carbonyl]amino}pent-4-enamido]-2-(ethoxycarbonyl)-5-(4-methylbenzenesulfonamido)pentanoic acid (9m) was obtained as a white amorphous solid (618 mg, 62%) from malonic acid monoester **8h** (1.16 g, 1.7 mmol) according to the general procedure H.

^{1}H NMR (300 MHz, CD_3OD) (mixture of diastereomers 1:1) δ 8.06 (s, 0.5H), 7.99 (s, 0.5H), 7.74 – 7.65 (m, 2H), 7.46 – 7.20 (m, 7H), 7.89 – 7.70 (m, 1H), 5.25 – 4.99 (m, 4H), 4.32 – 4.03 (m, 3H), 2.82 – 2.63 (m, H), 2.58 – 2.46 (m,1H), 3.44 – 2.31 (m, 2H), 2.41 (s, 3H), 2.30 – 2.14 (m, 2H), 1.42 – 1.14 (m, 5H).

^{13}C NMR (75 MHz, $CDCl_3$) (mixture of diastereomers 1:1) δ 171.6, 171.2, 169.5, 168.0, 167.7, 156.7, 143.5, 143.5, 136.6, 136.1, 132.5, 129.8, 128.6, 128.3, 128.1, 128.0, 127.2, 119.6, 67.5, 67.4, 66.2, 66.1, 63.1, 63.1, 54.3, 54.0, 42.7, 36.7, 30.0, 29.9, 23.8, 21.6, 14.0.

HRMS (ESI/Q-TOF) *m*/*z*: $[M+H]^+$ Calcd for $C_{28}H_{36}N_3O_9S$ 590.2172; Found 590.2188.

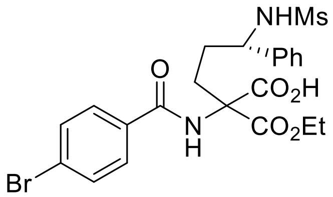

(5*S*)-2-[(4-Bromophenyl)formamido]-2-(ethoxycarbonyl)-5-methanesulfonamido-5-phenylpentanoic acid (9n) was obtained as a white amorphous solid (154 mg, 85%) from malonic acid monoester **8i** (214 mg, 0.3 mmol) according to the general procedure H.

^{1}H NMR (300 MHz, $CDCl_3$) (mixture of diastereomers 41:59) δ 8.64 (s, 1H), 7.76 – 7.57 (m, 3H), 7.56 – 7.48 (m, 2H), 7.35 – 7.18 (m, 5H), 6.06 (d, *J* = 8.3 Hz, 0.41H), 5.96 (d, *J* = 8.3 Hz, 0.59H), 4.53 – 4.33 (m, 1H), 4.29 – 4.11 (m, 2H), 2.76 – 2.28 (m, 5H), 1.92 – 1.62 (m, 2H), 1.18 (t, *J* = 7.1 Hz, 1.23H,) 1.17 (t, *J* = 7.1 Hz, 1.77H).

^{13}C NMR (75 MHz, $CDCl_3$) (mixture of diastereomers 41:59) δ 169.7, 168.7, 168.2, 166.8, 166.5, 141.2, 140.8, 132.0, 132.0, 131.6, 131.4, 129.1, 128.2, 127.4, 127.2, 126.7, 126.6, 66.4, 66.2, 63.4, 63.2, 58.1, 57.9, 41.8, 31.7, 29.7, 29.5, 14.0, 14.0

HRMS (ESI/Q-TOF) *m*/*z*: $[M+H]^+$ Calcd for $C_{22}H_{26}N_2O_7SBr$ 541.0644; Found 541.0624.

Electrochemical decarboxylation-cyclization

General procedure K for electrochemical decarboxylation-cyclization

An undivided electrochemical cell (5 mL, IKA ElectraSyn 2.0) was charged with starting carboxylic acid **9a**,**b**,**d**–**f**,**j**–**n** (1 equiv) and Et_4NBF_4 (0.025 M), followed by addition of MeCN (2.5 mL) and H_2O (0.5 mL). Graphite plate 8×52.5×2 mm (immersed electrode surface area A = 1.12 cm^2) was used as the working electrode and stainless steel 8×52.5×2 mm (immersed electrode surface area A = 1.12 cm^2) was used as the counter electrode. The electrolysis was carried out under galvanostatic conditions at room

temperature, and 2.0 *F* charge (if not otherwise noted) with current density of 12 mA/cm^2 was passed through the colorless reaction solution. The resulting clear, colorless (sometimes pale yellow) solution was concentrated *in vacuo* and the crude product was purified by flash column chromatography.

General procedure L for electrochemical decarboxylation-cyclization

An undivided electrochemical cell (5 mL, IKA ElectraSyn 2.0) was charged with starting carboxylic acid **2a**–**f**,**m** (1 equiv) and Et_4NBF_4 (0.025 M), followed by addition of MeCN (2.5 mL) and H_2O (0.5 mL). Graphite plates 8×52.5×2 mm (immersed electrode surface area A = 1.12 cm^2) were used as the working electrode and as the counter electrode. The electrolysis was carried out under galvanostatic conditions at room temperature, and 2.0 *F* charge (if not otherwise noted) with current density of 12 mA/cm^2 was passed through the colorless reaction solution. The resulting clear, colorless (sometimes pale yellow) solution was concentrated *in vacuo* and the crude product was purified by flash column chromatography.

General procedure M for electrochemical decarboxylation-cyclization

An undivided electrochemical cell (5 mL, IKA ElectraSyn 2.0) was charged with starting carboxylic acid **1h**–**j** (1 equiv) and Et_4NBF_4 (0.025 M), followed by addition of MeCN (2.5 mL) and KOH (0.5 equiv or 1 equiv) solution in H_2O (0.5 mL). Graphite plate 8×52.5×2 mm (immersed electrode surface area A = 1.12 cm^2) was used as the working electrode and stainless steel 8×52.5×2 mm (immersed electrode surface area A = 1.12 cm^2) was used as the counter electrode. The electrolysis was carried out under galvanostatic conditions at room temperature, and 2.0 *F* charge (if not otherwise noted) with current density of 12 mA/cm^2 was passed through the colorless reaction solution. The resulting clear, colorless (sometimes pale yellow) solution was concentrated *in vacuo* and the crude product was purified by flash column chromatography.

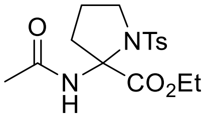

Ethyl 2-acetamido-1-(4-methylbenzenesulfonyl)pyrrolidine-2-carboxylate (6a) was obtained from malonic acid monoester **9a** (120 mg, 0.3 mmol) according to general procedure L. The crude product was purified by reversed phase flash column chromatography using gradient elution from 5% to 100% MeCN in water containing 0.01% TFA to afford the title compound as a white amorphous solid (77 mg, 72%); analytical TLC on silica gel, 2:1 EtOAc/petroleum ether, R_f = 0.12. The title compound was also isolated in 75% yield from malonic acid monoester **9a** (95 mg, 0.24 mmol), following the general procedure K by passing charge of 2.5 *F*.

^{1}H NMR (300 MHz, $CDCl_3$) δ 7.69 – 7.57 (m, 21H), 7.28 – 7.21 (m, 2H), 4.41 – 4.23 (m, 2H), 3.85 – 3.67 (m, 2H), 2.88 – 2.70 (m, 1H), 2.39 (s, 3H), 2.24 – 2.08 (m, 3H), 1.70 (s, 3H), 1.36 (t, J = 7.1 Hz, 3H).

^{13}C NMR (75 MHz, $CDCl_3$) δ 172.0, 168.7, 143.3, 137.3, 129.5, 127.0, 76.9, 63.2, 49.9, 37.1, 24.8, 23.9, 21.7, 14.1.

HRMS (ESI/Q-TOF) *m*/*z*: $[M+Na]^+$ Calcd for $C_{16}H_{22}N_2O_5SNa$ 377.1147; Found 377.1158.

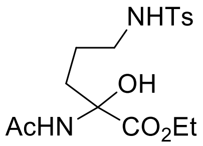

Ethyl 2-acetamido-2-hydroxy-5-(4-methylbenzene-1-sulfonamido)pentanoate (10a) was formed during the electrolysis of malonic acid monoester **9a** and was detected by LC-MS assay (UV detection). The title hemiaminal **10a** could not be isolated due to the instability on silica gel.

ESI-MS: *m*/*z*: 395.1 $[M+Na]^+$; 411.2 $[M+K]^+$.

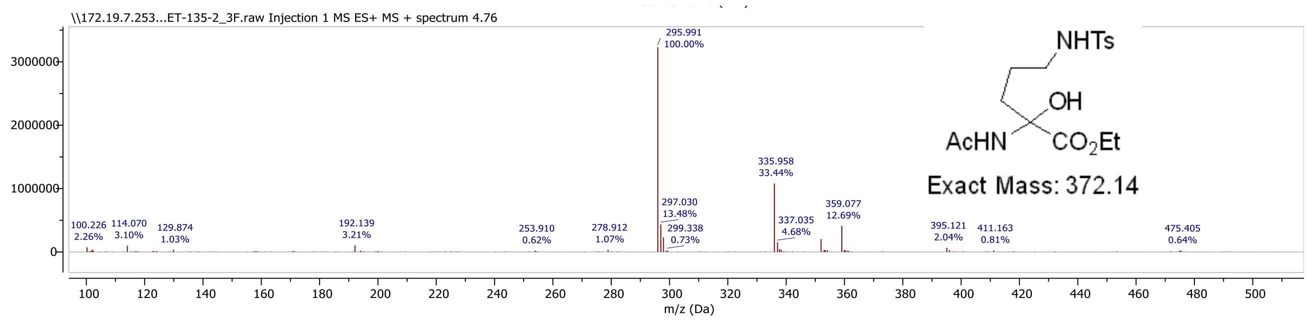

Figure S1. Mass spectrum of hemiaminal **10a**

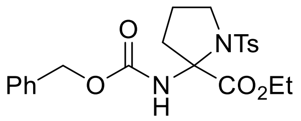

Ethyl 2-{[(benzyloxy)carbonyl]amino}-1-(4-methylbenzenesulfonyl)pyrrolidine-2-carboxylate (6b) was obtained from malonic acid monoester **9b** (148 mg, 0.3 mmol) according to general procedure L by passing charge of 2.5 *F*. The crude product was purified by reversed phase flash column chromatography using gradient elution from 5% to 100% MeCN in water containing 0.01% TFA to afford the title compound as a off-white amorphous solid (66 mg, 49%); analytical TLC on silica gel, 1:4 EtOAc/petroleum ether, R_f = 0.21. The title product was also obtained according to general procedure K (76 mg, 57%) from malonic acid monoester **9b** (148 mg, 0.3 mmol) by passing charge of 2.5 *F*.

^{1}H NMR (300 MHz, $CDCl_3$) δ 7.72 – 7.63 (m, 2H),7.42 – 7.32 (m, 3H), 7.32 – 7.23 (m, 2H),7.21 – 7.11 (m, 2H), 6.58 (s, 1H), 4.81 (d, J = 12.3 Hz, 1H), 4.55 (d, J = 12.3 Hz, 1H), 4.43 – 4.34 (m, 2H), 3.88 – 3.77 (m, 1H), 3.75 – 3.60 (m, 1H), 2.85 – 2.65 (m, 1H), 2.36 (s, 3H), 2.27 – 2.07 (m, 3H), 1.37 (t, J = 7.0 Hz, 3H).

^{13}C NMR (75 MHz, $CDCl_3$) δ 171.5, 153.5, 143.0, 137.4, 136.0, 129.4, 128.7, 128.3, 127.8, 127.1, 77.2, 66.2, 63.2, 49.6, 37.5, 24.5, 21.6, 14.1.

HRMS (ESI/Q-TOF) *m*/*z*: $[M+Na]^+$ Calcd for $C_{22}H_{26}N_2O_6SNa$ 469.1409; Found 469.1420.

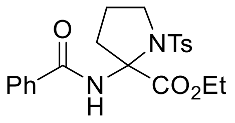

Ethyl 2-benzamido-1-(4-methylbenzenesulfonyl)pyrrolidine-2-carboxylate (6c) was obtained from malonic acid monoester **9c** (139 mg, 0.3 mmol) according to general procedure L by passing charge of 2.5 *F*. The crude product was purified by reversed phase flash column chromatography using gradient elution from 5% to 100% MeCN in water containing 0.01% TFA to afford the title compound as a colorless amorphous solid (66 mg, 53%); analytical TLC on silica gel, 1:4 EtOAc/petroleum ether, R_f = 0.18.

^{1}H NMR (300 MHz, $CDCl_3$) δ 7.88 (s, 1H), 7.59 – 7.46 (m, 5H), 7.44 – 7.35 (m, 2H), 7.00 – 6.92 (m, 2H), 4.49 – 4.29 (m, 2H), 3.97 – 3.78 (m, 2H), 3.01 – 2.81 (m, 1H), 2.35 – 2.14 (m, 3H), 2.25 (s, 3H), 1.41 (t, *J* = 7.1 Hz, 3H).

^{13}C NMR (75 MHz, $CDCl_3$) δ 172.2, 165.2, 143.0, 136.9, 133.7, 132.0, 129.4, 128.4, 127.0, 77.1, 63.3, 49.9, 37.2, 24.9, 21.5, 14.1.

HRMS (ESI/Q-TOF) *m*/*z*: $[M+Na]^+$ Calcd for $C_{21}H_{24}N_2O_5SNa$ 439.1304; Found 439.1301.

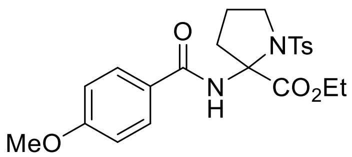

Ethyl 2-(4-methoxybenzamido)-1-(4-methylbenzenesulfonyl)pyrrolidine-2-carboxylate (6d) was obtained from malonic acid monoester **9d** (131 mg, 0.3 mmol) according to the general procedure L. The crude product was purified by reversed phase flash column chromatography using gradient elution from 5% to 100% MeCN in water containing 0.01% TFA to afford the title compound as a colorless amorphous solid (59 mg, 50%); analytical TLC on silica gel, 1:4 EtOAc/petroleum ether, R_f = 0.25. The title product was also obtained according to general procedure K (41 mg, 63%) from malonic acid monoester **9d** (72 mg, 0.15 mmol).

^{1}H NMR (300 MHz, $CDCl_3$) δ 7.77 (s, 1H), 7.56 – 7.46 (m, 4H), 7.00 – 6.93 (m, 2H), 6.91 – 6.84 (m, 2H), 4.48 – 4.28 (m, 2H), 3.94 – 3.75 (m, 2H), 3.86 (s, 3H), 2.99 – 2.80 (m, 1H), 2.33 – 2.12 (m, 3H), 2.26 (s, 3H), 1.40 (t, *J* = 7.1 Hz, 3H).

^{13}C NMR (75 MHz, $CDCl_3$) δ 172.4, 164.8, 162.6, 142.9, 136.8, 129.4, 128.9, 127.0, 126.2, 126.2, 113.6, 77.1, 63.3, 55.6, 49.9, 37.2, 24.9, 21.5, 14.1.

HRMS (ESI/Q-TOF) *m*/*z*: $[M+Na]^+$ Calcd for $C_{22}H_{26}N_2O_6SNa$ 469.1409; Found 469.1416.

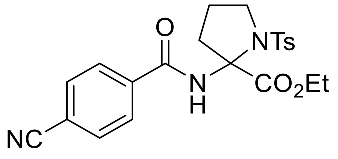

Ethyl 2-(4-cyanobenzamido)-1-(4-methylbenzenesulfonyl)pyrrolidine-2-carboxylate (6e) was obtained from malonic acid monoester **9e** (82 mg, 0.17 mmol) according to the general procedure L. The crude product was purified by reversed phase flash column chromatography using gradient elution from 5% to 100% MeCN

in water containing 0.01% TFA to afford the title compound as a white amorphous solid (28 mg, 38%); analytical TLC on silica gel, 1:2 EtOAc/petroleum ether, R_f = 0.36. The title product was also obtained according to general procedure K (30 mg, 41%) from malonic acid monoester **9e** (80 mg, 0.16 mmol).

^{1}H NMR (300 MHz, $CDCl_3$) δ 7.93 (s, 1H), 7.76 – 7.61 (m, 4H), 7.57 – 7.47 (m, 2H), 7.05 – 6.96 (m, 2H), 4.47 – 4.26 (m, 2H), 3.95 – 3.77 (m, 2H), 2.95 – 2.81 (m, 1H), 2.37 – 2.15 (m, 6H), 1.39 (t, *J* = 7.2 Hz, 3H).

^{13}C NMR (75 MHz, $CDCl_3$) δ 171.9, 163.5, 143.3, 137.7, 136.9, 132.4, 129.5, 127.7, 127.0, 118.0, 115.6, 77.2, 63.6, 50.0, 37.3, 24.8, 21.5, 14.1.

HRMS (ESI/Q-TOF) *m/z*: $[M+Na]^+$ Calcd for $C_{22}H_{23}N_3O_5SNa$ 464.1256; Found 464.1266.

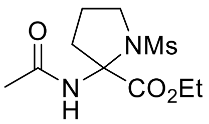

Ethyl 2-acetamido-1-methanesulfonylpyrrolidine-2-carboxylate (6f) was obtained from malonic acid monoester **9f** (97 mg, 0.3 mmol) according to the general procedure L by passing charge of 2.3 *F*. The crude product was purified by flash column chromatography on silica gel using gradient elution from 20% to 65% EtOAc in petroleum ether followed by isocratic elution with 65% EtOAc in petroleum ether to afford the title compound as a colorless amorphous solid (50 mg, 60%). The title product was also obtained according to the general procedure K (48 mg, 58%) from malonic acid monoester **9f** (177 mg, 0.3 mmol) by passing charge of 2.3 *F*; analytical TLC on silica gel, 2:1 EtOAc/petroleum ether, R_f = 0.25.

^{1}H NMR (300 MHz, DMSO-d_6) δ 8.26 (s, 1H), 4.16 (qd, *J* = 7.1, 1.4 Hz, 2H), 3.69 – 3.59 (m, 1H), 3.50 (td, *J* = 8.3, 3.6 Hz, 1H), 2.83 (s, 3H), 2.63 (ddd, *J* = 12.8, 9.8, 8.0 Hz, 1H), 2.14 (ddd, *J* = 12.9, 8.1, 3.6 Hz, 1H), 2.08 – 1.97 (m, 1H), 1.96 – 1.81 (m, 1H), 1.93 (s, 3H), 1.21 (t, *J* = 7.1 Hz, 3H).

^{13}C NMR (75 MHz, DMSO-d_6) δ 170.1, 169.9, 77.2, 61.8, 48.8, 39.1, 37.2, 23.3, 22.7, 13.8.

HRMS (ESI/Q-TOF) *m/z*: $[M+Na]^+$ Calcd for $C_{10}H_{18}N_2O_5SNa$ 301.0841; Found 301.0834.

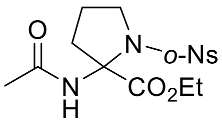

Ethyl 2-acetamido-1-(2-nitrobenzenesulfonyl)pyrrolidine-2-carboxylate (6g). Anodic and cathodic chambers of a divided cell were each charged with Et_4NBF_4 (43 mg, 0.2 mmol, 1 equiv) followed by addition of MeCN (6.5 mL) and NaOH (8 mg, 0.2 mmol, 1 equiv) in H_2O (2.3 mL). To the anodic chamber was added carboxylic acid **9g** (86 mg, 0.2 mmol, 1 equiv). Two graphite rods with diameter of 6 mm and length of 153 mm (immersed electrode surface area A = 2.07 cm^2) were used as the working electrode and as the counter electrode. The electrolysis was carried out under galvanostatic conditions at current density of 12 mA/cm^2 at room temperature by passing charge of 2.0 *F*. The resulting transparent, pale-yellow solution from the anodic chamber was concentrated *in vacuo* and purified by reversed phase flash column chromatography using gradient elution from 5% to 100% MeCN in water containing 0.01% TFA. This was followed by another

purification by flash column chromatography on silica gel using gradient elution from 10% to 80% EtOAc in petroleum ether to afford the title compound as a thick pale-yellow oil (20 mg, 25%); analytical TLC on silica gel, 2:1 EtOAc/petroleum ether, R_f = 0.26.

^{1}H NMR (300 MHz, $CDCl_3$) δ 7.99 – 7.90 (m, 1H), 7.70 – 7.60 (m, 2H), 7.65 – 7.55 (m, 1H), 7.15 (s, 1H), 4.33 (qd, *J* = 7.1, 3.0 Hz, 2H), 4.05 – 3.87 (m, 2H), 2.98 – 2.80 (m, 1H), 2.31 – 2.08 (m, 3H), 1.71 (s, 3H), 1.35 (t, *J* = 7.1 Hz, 3H).

^{13}C NMR (75 MHz, $CDCl_3$) δ 171.5, 169.1, 148.1, 133.5, 133.5, 131.6, 130.1, 124.1, 77.6, 63.5, 51.1, 36.8, 24.9, 23.9, 14.1.

HRMS (ESI/Q-TOF) *m*/*z*: $[M+Na]^+$ Calcd for $C_{15}H_{19}N_3O_9NaS$ 408.0857; Found 408.0841.

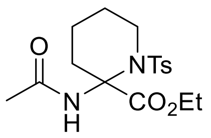

Ethyl 2-acetamido-1-(4-methylbenzenesulfonyl)piperidine-2-carboxylate (6h) was obtained from malonic acid monoester **9h** (62 mg, 0.15 mmol) according to the general procedure M by passing charge of 4.0 *F*. The crude product was purified by reversed phase flash column chromatography using gradient elution from 5% to 100% MeCN in water containing 0.01% TFA to afford the title compound as a colorless semisolid (15 mg, 27%); analytical TLC on silica gel, 1:1 EtOAc/petroleum ether, R_f = 0.32.

^{1}H NMR (300 MHz, $CDCl_3$) δ 7.68 (d, *J* = 8.2 Hz, 2H), 7.31 – 7.21 (m, 2H), 6.88 (s, 1H), 4.35 (q, *J* = 7.1 Hz, 2H), 3.71 – 3.55 (m, 2H), 3.05 – 2.88 (m, 1H), 2.40 (s, 3H), 1.88 – 1.64 (m, 5H), 1.76 (s, 3H), 1.38 (t, *J* = 7.1 Hz, 3H).

^{13}C NMR (75 MHz, $CDCl_3$) δ 171.7, 169.4, 143.6, 137.2, 129.5, 127.4, 72.8, 63.1, 43.3, 31.0, 24.3, 22.2, 21.7, 17.1, 14.1.

HRMS (ESI/Q-TOF) *m*/*z*: $[M+Na]^+$ Calcd for $C_{17}H_{24}N_2O_5SNa$ 391.1304; Found 391.1296.

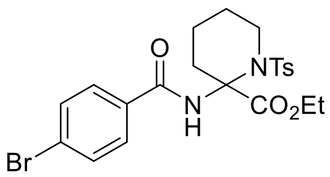

Ethyl 2-(4-bromobenzamido)-1-(4-methylbenzenesulfonyl)piperidine-2-carboxylate (6i) was obtained from malonic acid monoester **9i** (62 mg, 0.15 mmol) according to general procedure M by passing charge of 3.0 *F*. The crude product was purified by reversed phase flash column chromatography using gradient elution from 5% to 100% MeCN in water containing 0.01% TFA to afford the title compound as a white amorphous solid (13 mg, 18%); analytical TLC on silica gel, 1:1 EtOAc/petroleum ether, R_f = 0.65.

^{1}H NMR (300 MHz, $CDCl_3$) δ 7.71 (s, 1H), 7.60 – 7.50 (m, 4H), 7.48 – 7.41 (m, 2H), 7.07 – 7.00 (m, 2H), 4.40 (q, *J* = 7.1 Hz, 2H), 3.76 – 3.66 (m, 2H), 3.12 – 2.94 (m, 1H), 2.30 (s, 3H), 1.97 – 1.70 (m, 5H), 1.41 (t, *J* = 7.1 Hz, 3H).

^{13}C NMR (75 MHz, $CDCl_3$) δ 172.0, 165.0, 143.4, 136.9, 133.2, 131.8, 129.5, 128.7, 127.4, 126.6, 73.0, 63.4, 43.4, 31.1, 22.3, 21.6, 17.2, 14.2.

HRMS (ESI/Q-TOF) *m*/*z*: $[M+Na]^+$ Calcd for $C_{22}H_{25}N_2O_5SBrNa$ 531.0565; Found 531.0579.

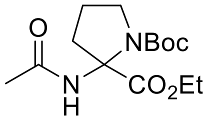

1-*Tert*-butyl 2-ethyl 2-acetamidopyrrolidine-1,2-dicarboxylate (6j) was obtained from malonic acid monoester **9j** (104 mg, 0.3 mmol) according to general procedure K by passing charge of 2.3 *F*. The colorless reaction solution was concentrated *in vacuo* and purified by flash column chromatography on silica gel using gradient elution from 33% to 80% EtOAc in petroleum ether to afford the title compound as a thick colorless oil (34 mg, 38%); analytical TLC on silica gel, 1:1 EtOAc/petroleum ether, R_f = 0.30. The title product was also obtained according to the general procedure M (53 mg, 59%) from malonic acid monoester **9j** (104 mg, 0.3 mmol) in presence of 0.5 equiv of KOH.

Upscale synthesis of pyrrolidine 6j. An undivided cell (IKA) was charged with carboxylic acid **9j** (925 mg, 2.7 mmol) and Et_4NBF_4 (123 mg, 0.4 mmol; 0.025M), followed by addition of MeCN (12.5 mL) and KOH (0.5 equiv) solution in H_2O (2.5 mL). Graphite plate (8×52.5×2 mm; immersed electrode surface area A = 2.24 cm^2) was used as the working electrode and stainless steel plate (8×52.5×2 mm; immersed electrode surface area A = 2.24 cm^2) was used as the counter electrode. The electrolysis was carried out under galvanostatic conditions at room temperature with a current density of 12 mA/cm^2. After 2.0 *F* charge was passed through the colorless solution, precipitation of white compound was observed at the working electrode. The working electrode was changed to a clean one, and another 0.5 *F* charge was passed through the electrolysis solution. After completion, the colorless reaction solution was concentrated *in vacuo*, and the residue was purified by flash column chromatography on silica gel using gradient elution from 33% to 80% EtOAc in petroleum ether to afford the title compound as a white amorphous solid (475 mg, 59%).

^{1}H NMR (300 MHz, $CDCl_3$) (mixture of rotamers) δ 7.03 (s, 0.6H), 6.85 (s, 0.4H), 4.37 – 4.07 (m, 2H), 3.81 – 3.51 (m, 2H), 2.88 – 2.65 (m, 1H), 2.26 – 2.05 (m, 2H), 2.02 – 1.87 (m, 1H), 1.96 (s, 3H), 1.39 (s, 5.4H), 1.36 (s, 3.6H), 1.33 – 1.18 (m, 3H).

^{13}C NMR (75 MHz, $CDCl_3$) (mixture of rotamers) δ 172.3, 172.1, 169.4, 168.6, 153.6, 152.1, 80.5, 80.3, 76.1, 75.4, 62.6, 62.4, 48.1, 47.9, 37.2, 36.0, 28.4, 24.3, 24.2, 23.4, 14.1.

HRMS (ESI/Q-TOF) *m*/*z*: $[M+Na]^+$ Calcd for $C_{14}H_{24}N_2ONa$ 369.1646; Found 369.1651.

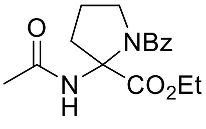

Ethyl 1-benzoyl-2-acetamidopyrrolidine-2-carboxylate (6k) was obtained from malonic acid monoester **9k** (105 mg, 0.3 mmol) according to the general procedure K by passing charge of 2.2 *F*. The crude product was purified by reversed phase flash

column chromatography using gradient elution from 0% to 100% MeCN in water containing 0.01% TFA to afford the title compound as a white amorphous solid (35 mg, 38%); analytical TLC on silica gel, 1:1 EtOAc/petroleum ether, R_f = 0.24.

^{1}H NMR (300 MHz, $CDCl_3$) δ 7.56 – 7.49 (m, 2H), 7.46 – 7.33 (m, 3H), 7.25 (s, 1H), 4.42 – 4.20 (m, 2H), 3.62 – 3.50 (m, 1H), 3.05 – 2.86 (m, 1H), 2.15 – 2.03 (m, 3H), 2.01 (s, 3H), 1.31 (t, *J* = 7.1 Hz, 3H).

^{13}C NMR (75 MHz, CD_3CN) δ 171.9, 170.1, 169.0, 137.3, 131.3, 129.3, 128.0, 77.1, 63.0, 51.8, 36.0, 25.8, 24.0, 14.4.

HRMS (ESI/Q-TOF) *m*/*z*: $[M+Na]^+$ Calcd for $C_{16}H_{20}N_2O_4Na$ 327.1324; Found 327.1321.

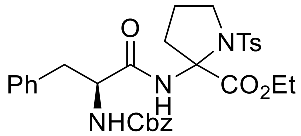

Ethyl 2-[(2*S*)-2-{[(benzyloxy)carbonyl]amino}-3-phenylpropanamido]-1-(4-methylbenzenesulfonyl)pyrrolidine-2-carboxylate (6l) was obtained from malonic acid monoester **9l** (153 mg, 0.2 mmol) according to general procedure K. The crude product was purified by reversed phase flash column chromatography using gradient elution from 5% to 100% MeCN in water containing 0.01% TFA to afford the title compound as a colorless semisolid (51 mg, 36%); analytical TLC on silica gel, 1:1 EtOAc/petroleum ether, R_f = 0.54.

^{1}H NMR (300 MHz, $CDCl_3$) (mixture of diastereomers 37:63) δ 7.82 (s, 0.4H, minor), 7.72 – 7.63 (m, 0.7H, minor), 7.62 –7.50 (m, 1.3H, major), 7.42 – 7.02 (m, 12.6H), 5.22 – 5.00 (m, 2.6H), 4.95 (d, *J* = 8.7 Hz, 0.4H, minor), 4.38 – 4.15 (m, 3H), 3.83 – 3.70 (m, 1.3H, major), 3.73 – 3.58 (m, 0.7H, minor), 3.03 – 2.79 (m, 2H), 2.75 – 2.63 (m, 0.4H, minor), 2.60 – 2.45 (m, 0.6H, major), 2.41 (s, 1.1H, minor), 2.24 (s, 1.9H,major), 2.20 – 2.00 (m, 3H), 1.34 (t, *J* = 7.1 Hz, 1.1H, minor) 1.34 (t, *J* = 7.1 Hz, 1.9H, major).

^{13}C NMR (75 MHz, $CDCl_3$) (mixture of diastereomers 37:63) δ 171.4, 171.2, 169.8, 169.0, 156.1, 155.5, 143.5, 137.1, 137.0, 136.3, 136.0, 129.7, 129.5, 129.3, 128.9, 128.8, 128.7, 128.7, 128.4, 128.3, 128.2, 128.1, 127.2, 126.9, 77.1, 76.8, 67.1, 63.2, 56.1, 49.8, 49.6, 39.2, 37.4, 37.0, 24.8, 21.7, 21.5, 14.1.

HRMS (ESI/Q-TOF) *m*/*z*: $[M+Na]^+$ Calcd for $C_{31}H_{35}N_3O_7SNa$ 616.2093; Found 616.2097.

HPLC/csp: 63:37 d.r., HPLC/csp assay: Daicel CHIRALPAK IA, 250 mm × 4.6 mm, 5µm, mobile phase 20% IPA:80%Heptane, flow rate 1 mL/min, detector UV 231 nm, retention time **minor-6l**, 13.5 min, and **major-6l**, 23.3 min.

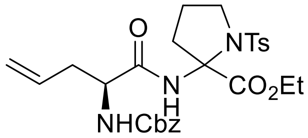

Ethyl 2-[(2*S*)-2-{[(benzyloxy)carbonyl]amino}pent-4-enamido]-1-(4-methyl benzenesulfonyl)pyrrolidine-2-carboxylate (6m) was obtained from malonic acid monoester **9m** (177 mg, 0.3 mmol) according to the general

procedure L. The crude product was purified by reversed phase flash column chromatography using gradient elution from 5% to 100% MeCN in water containing 0.01% TFA to obtain the title compound as a thick colorless oil (61 mg, 37%); analytical TLC on silica gel, 1:1 EtOAc/petroleum ether, R_f = 0.55. The title product (81 mg, 50%) was also obtained from malonic acid monoester **9m** (177 mg, 0.3 mmol) according to the general procedure K.

^{1}H NMR (300 MHz, $CDCl_3$) (mixture of diastereomers 37:63) δ 7.83 (s, 0.33H), 7.73 – 7.49 (m, 2.66H), 7.43 – 7.23 (m, 5.63H), 7.22 – 7.09 (m, 1.37H), 5.75 – 5.54 (m, 1H), 5.27 – 4.96 (m, 5H), 4.39 – 4.22 (m, 2H), 4.21 – 3.97 (m, 1H), 3.82 – 3.62 (m, 2H), 2.77 – 2.57 (m, 1H), 2.55 – 2.02 (m, 8H), 1.35 (t, *J* = 7.1 Hz, 2.01H), 1.34 (t, *J* = 7.1 Hz, 0.99H).

^{13}C NMR (75 MHz, $CDCl_3$) (mixture of diastereomers 37:63) δ 171.5, 171.3, 170.0, 169.3, 156.2, 155.6, 143.5, 137.0, 136.9, 136.3, 136.2, 132.7, 132.4, 129.6, 129.5, 128.6, 128.3, 128.2, 127.2, 126.9, 119.6, 119.4, 77.0, 67.4, 67.1, 63.3, 63.2, 54.1, 49.7, 37.4, 37.3, 36.2, 24.7, 21.6, 21.4, 14.0.

HRMS (ESI/Q-TOF) *m*/*z*: $[M+Na]^+$ Calcd for $C_{27}H_{33}N_3O_7SNa$ 566.1937; Found 566.1949.

HPLC/csp: 63:37 d.r., HPLC/csp assay: Daicel CHIRALPAK IA, 250 mm × 4.6 mm, 5μm, mobile phase 20% IPA:80%Heptane, flow rate 1 mL/min, detector UV 231 nm, retention time **minor-6m**, 11.2 min, and **major-6m**, 23.0 min.

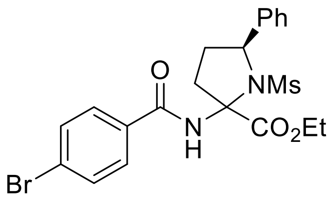

Ethyl (5*S*)-2-(4-bromobenzamido)-1-methanesulfonyl-5-phenylpyrrolidine-2-carboxylate (6n) was obtained according to the general procedure K from malonic acid monoester **9n** (151mg, 0.3 mmol) by passing charge of 2.2 *F*. The crude product was purified by reversed phase flash column chromatography using gradient elution from 5% to 100% MeCN in water containing 0.01% TFA to afford the title compound as a white amorphous solid (92 mg, 67%); analytical TLC on silica gel, 1:3 EtOAc/petroleum ether, R_f = 0.32.

^{1}H NMR (300 MHz, $CDCl_3$) δ 7.81 (s, 1H), 7.76 – 7.66 (m, 2H), 7.66 – 7.50 (m, 4H), 7.44 – 7.26 (m, 3H), 5.25 (dd, *J* = 8.7, 4.4 Hz, 1H), 4.42 (qd, *J* = 7.1, 1.1 Hz, 2H), 3.16 (ddd, *J* = 12.4, 8.1, 4.0 Hz, 1H), 3.01 (dq, *J* = 12.4, 8.7 Hz, 1H), 2.73 (dt, *J* = 12.9, 8.9 Hz, 1H), 2.49 (s, 3H), 2.19 – 2.04 (m, 1H), 1.42 (t, *J* = 7.1 Hz, 3H).

^{13}C NMR (75 MHz, $CDCl_3$) δ 171.0, 166.1, 141.9, 133.5, 132.1, 128.8, 128.8, 128.3, 127.7, 126.9, 81.0, 65.8, 63.6, 41.6, 36.3, 34.7, 14.2.

HRMS (ESI/Q-TOF) *m*/*z*: $[M+Na]^+$ Calcd for $C_{21}H_{23}N_2O_5SBrNa$ 517.0409; Found 517.0407.

$[\alpha]^{20}_D$ –167 (*c* 1.0, $CHCl_3$).

Anodic oxidation of pyrrolidine 6a

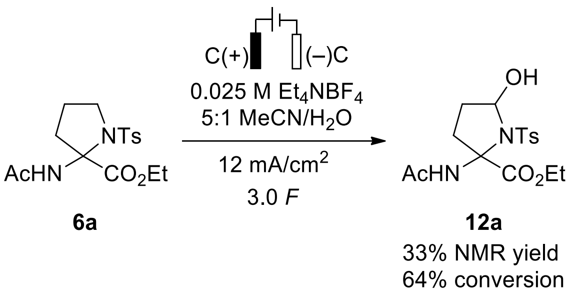

Ethyl 2-acetamido-5-hydroxy-1-(4-methylbenzenesulfonyl)pyrrolidine-2-carboxylate (12a). Pyrrolidine **6a** (51 mg, 0.14 mmol) and Et_4NBF_4 (16 mg, 0.75 mmol, 0.025 M in the reaction solution) were dissolved in 5:1 $MeCN/H_2O$ mixture (3 mL). Graphite plates (8×52.5×2 mm; immersed electrode surface area A = 1.12 cm^2) were used both as the working electrode and the counter electrode. The electrolysis was carried out under galvanostatic conditions at room temperature, and 3.0 *F* charge with a current density of 12 mA/cm^2 was passed through the colorless solution. The resulting transparent, light-yellow solution was concentrated *in vacuo,* and the crude product was analyzed by ^1H NMR using CH_2Br_2 as an internal standard. The title compound **12a** was formed in 33% NMR yield. The purification by reversed phase flash column chromatography using gradient elution from 5% to 100% MeCN in water containing 0.01% TFA resulted in the inseparable mixture of pyrrolidine **6a** and hemiaminal **12a**. The structure of the product **7** was elucidated from the results of NMR and HRMS assays.

^{1}H NMR (300 MHz, $CDCl_3$) δ 7.86 – 7.75 (m, 2H), 7.29 – 7.19 (m, 2H), 7.07 (s, 1H), 5.88 (br. s, 1H), 5.81 (br. s, 1H), 4.43 – 4.23 (m, 2H), 3.03 (td, *J* = 13.3, 7.7 Hz, 1H), 2.37 – 2.27 (m, 1H), 2.11 – 1.91 (m, 2H), 1.59 (s, 3H), 1.36 (t, *J* = 7.2 Hz, 3H).

^{13}C NMR (75 MHz, $CDCl_3$) δ 172.0, 171.3, 170.0, 168.8, 143.6, 143.3, 137.8, 137.2, 129.4, 129.3, 127.9, 127.0, 85.6, 76.9, 76.0, 63.5, 63.2, 49.8, 37.0, 33.8, 33.0, 24.8, 23.9, 21.6, 14.0.

HRMS (ESI/Q-TOF) *m*/*z*: $[M+Na]^+$ Calcd for $C_{16}H_{22}N_2O_6SNa$ 393.1096; Found 393.1095.

Deuterium-labeling studies

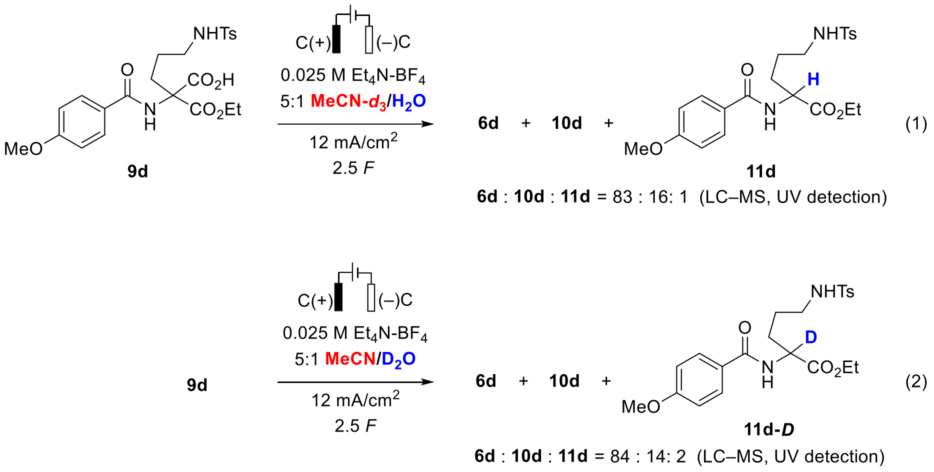

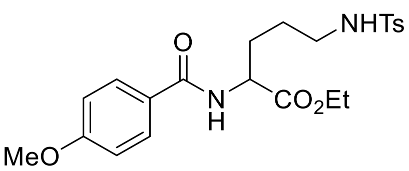

Ethyl 2-[(4-methoxyphenyl)formamido]-5-(4-methylbenzenesulfonamido)pentanoate (11d) was formed during storage of malonic acid monoester **9d** for two months at room temperature under air. Analytically pure sample of the title compound was obtained by reversed phase flash column chromatography using gradient elution from 5% to 100% MeCN in water containing 0.01% TFA; TLC on silica gel, 2:1 EtOAc/petroleum ether, R_f = 0.39.

^{1}H NMR (300 MHz, $CDCl_3$) δ 7.81 – 7.65 (m, 4H), 7.30 – 7.21 (m, 2H), 6.96 – 6.86 (m, 2H), 6.76 (d, *J* = 7.4 Hz, 1H), 5.08 (s, 1H), 4.78 – 4.65 (m, 1H), 4.21 (q, *J* = 7.1 Hz, 2H), 3.84 (s, 3H), 3.02 – 2.91 (m, 2H), 2.39 (s, 3H), 2.06 – 1.87 (m, 1H), 1.88 – 1.70 (m, 1H), 1.68 – 1.51 (m, 2H), 1.28 (t, *J* = 7.1 Hz, 3H).

^{13}C NMR (75 MHz, $CDCl_3$) δ 172.6, 167.0, 162.6, 143.5, 136.9, 129.8, 129.1, 127.2, 125.9, 113.9, 61.9, 55.6, 52.2, 42.8, 30.1, 25.6, 21.6, 14.3.

HRMS (ESI/Q-TOF) *m*/*z*: $[M+H]^+$ Calcd for $C_{22}H_{29}N_2O_6S$ 449.1746; Found 449.1751.

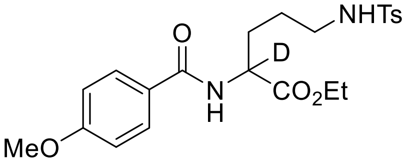

Ethyl 2-[(4-methoxyphenyl)formamido]-5-(4-methylbenzenesulfonamido)(2-2*H*)pentanoate (11d-*D*). An undivided electrochemical cell (IKA ElectraSyn 2.0) was charged with carboxylic acid **9d** (341 mg, 0.69 mmol, 1 equiv) and Et_4NBF_4 (37 mg, 0.025 M), followed by addition of MeCN (5.6 mL) and D_2O (1.2 mL). Graphite plate (8×52.5×2 mm; immersed electrode surface area A = 1.48 cm^2) was used as the working electrode and stainless steel plate (8×52.5×2 mm; immersed electrode surface area A = 1.48 cm^2) was used as the counter electrode. The electrolysis was carried out under galvanostatic conditions at room temperature, and 2.0 *F* charge with current density of 12 mA/cm^2 was passed through the colorless reaction solution. The

resulting transparent pale yellow solution was concentrated *in vacuo*. Purification of the crude material by reversed phase flash column chromatography using gradient elution from 5% to 100% MeCN in water containing 0.01% TFA afforded ~2 mg (0.6%) of the title compound **11d-*D*** as a pale-yellow semisolid and 150 mg (49%) of pyrrolidine **6d**.

HRMS (ESI/Q-TOF) *m*/*z*: $[M+H]^+$ Calcd for $C_{22}H_{28}N_2O_6SD$ 450.1809; Found 450.1820.

Comparison of ^1H NMR spectra of compounds 11d–*D* and 11d

The amount of deuterium incorporation was determined by comparing integrals of signals A (<u>CH</u>) and B (O<u>CH$_2$</u>CH$_3$) in ^1H NMR spectra for 11d-*D* and 11d (see the spectra below). For **11d** (0 % deuterium incorporation) the A:B integral ratio was 1:2. In contrast, for **11d-*D***, the A:B integral ratio is 0.35:2, indicating 65% of deuterium incorporation.

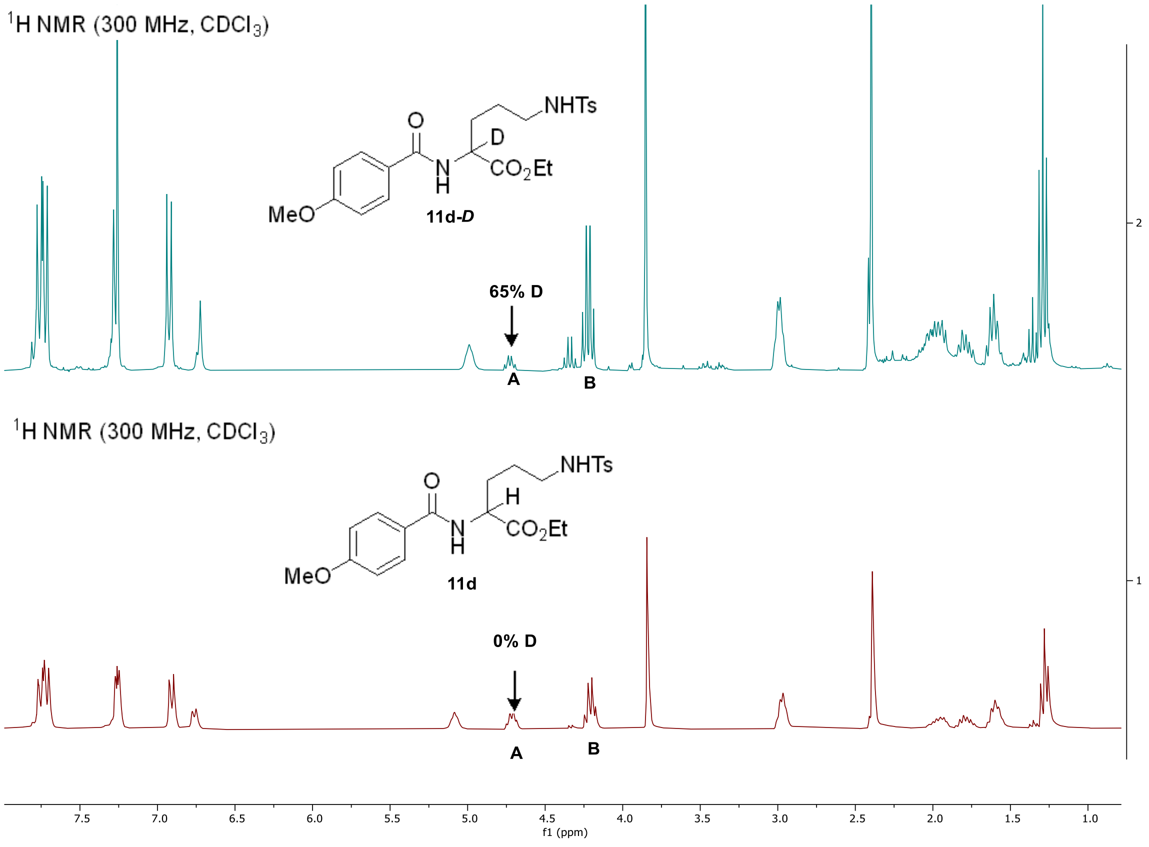

Comparison of MS spectra of of compounds 11d–*D* and 11d

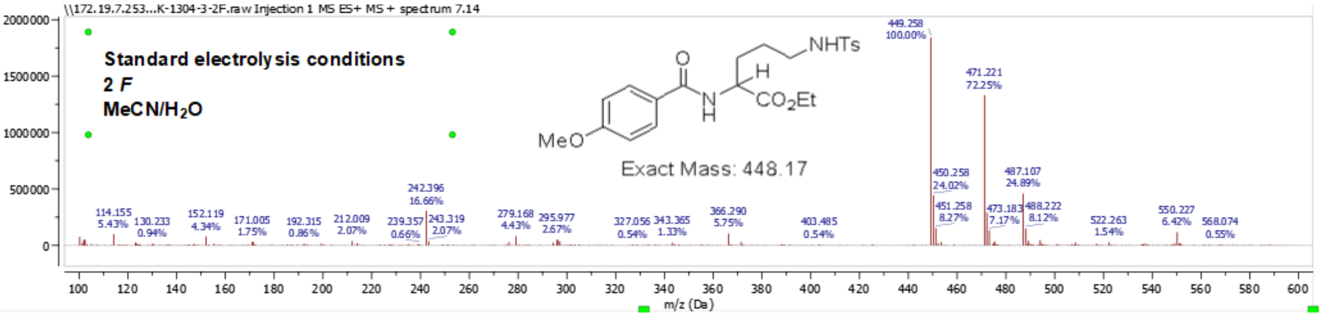

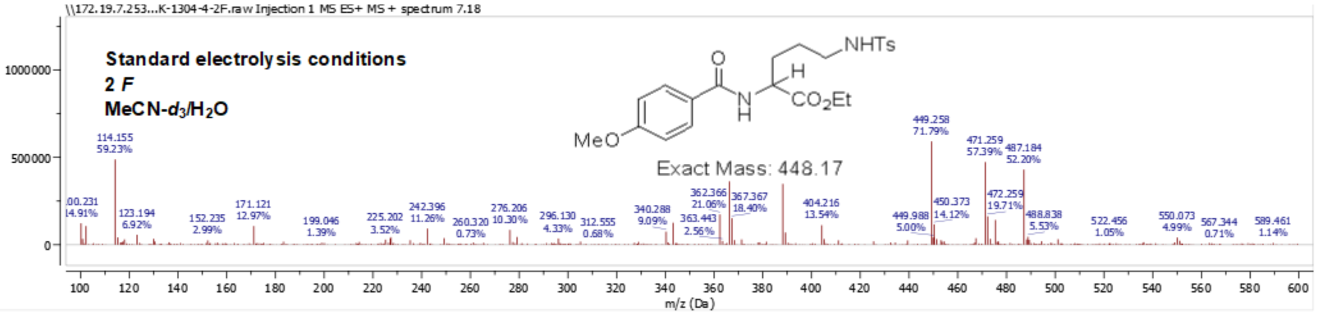

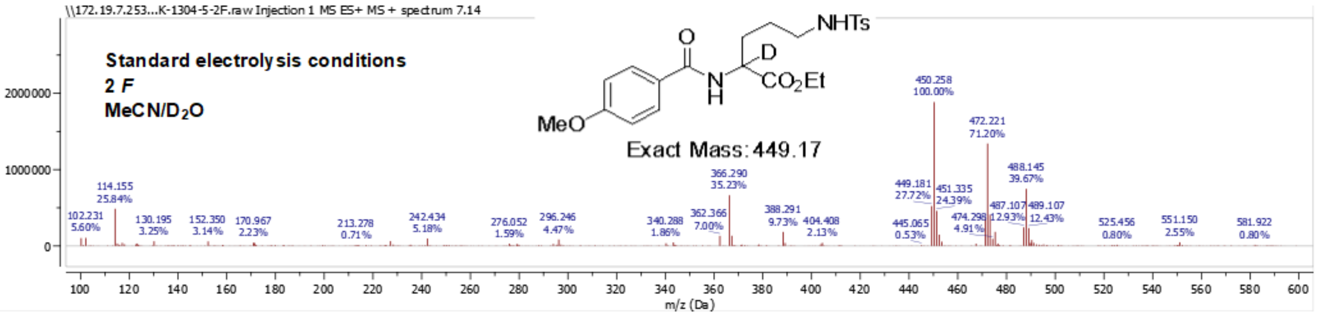

Cyclic voltammetry

CV experiments were carried out in an SVC-2 (ALS, Japan) three-electrode cell using a PalmSens4 (PalmSens). A glassy carbon disk (diameter: 1.6 mm) served as the working electrode, and a platinum wire as the counter electrode. The glassy carbon disk was polished using polishing alumina (0.05 μm) prior to each experiment. As a reference, $Ag/AgNO_3$ electrode [silver wire in 0.1 $MNBu_4ClO_4/CH_3CN$ solution; $c(AgNO_3)$ = 0.01 M; E_0 = −87 mV vs Fc/Fc^+ couple] [9] was used, and this compartment was separated from the rest of the cell with a Vycor frit. Et_4NBF_4 (0.1 M, electrochemical grade) was employed as the supporting electrolyte in 5:1 $MeCN/H_2O$ solution. The electrolyte was purged with Ar for at least 3 min prior to recording. Compounds **S1a**,**9a**,**6a** were analyzed at a concentration of 3 mM or 6mM and scan rate of 100 V s^{-1}). The peak potential E_P was not extracted from background-corrected voltammograms. All CV graphs are plotted using IUPAC polarographic convention.

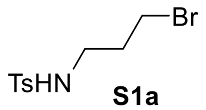

S1a

E_P = +1.83 V
v = 100 mV/s
c = 3 mM
Solvent: $MeCN/H_2O$, 5:1
Start point = 0.0 V, scanned in positive direction

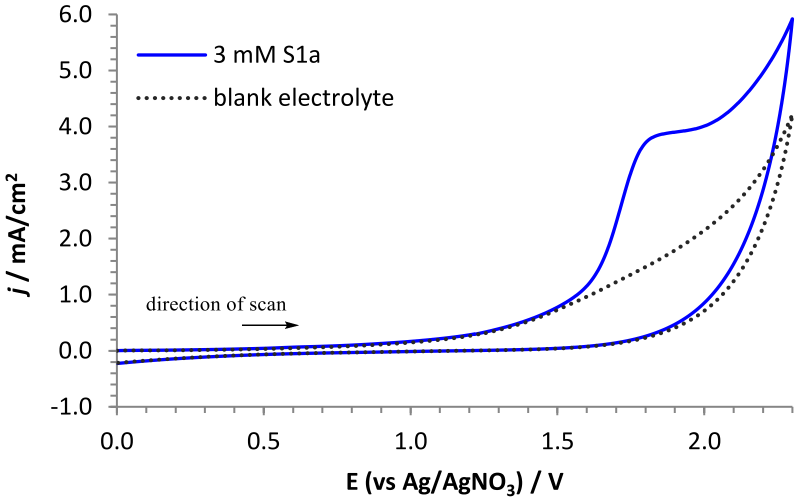

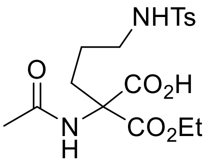

9a

E_{P1} = +1.56 V
E_{P2} = +1.76 V
v = 100 mV/s
c = 6 mM
Solvent: MeCN/H_2O, 5:1
Start point = 0.0 V, scanned in positive direction

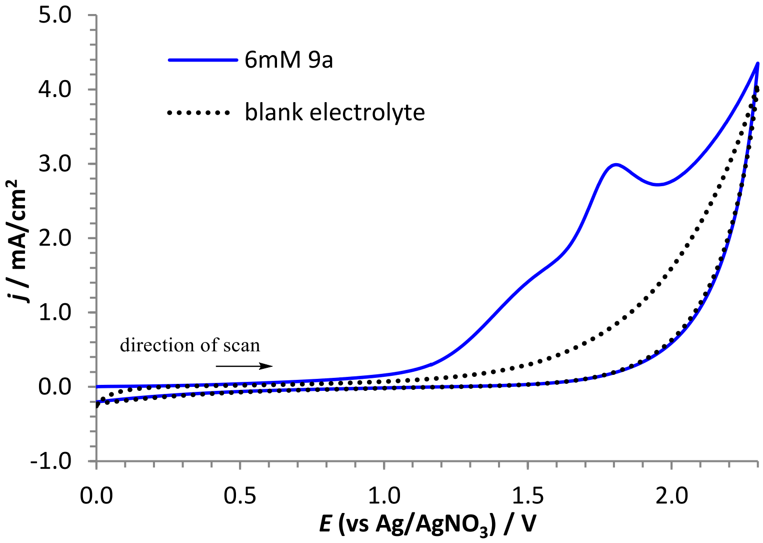

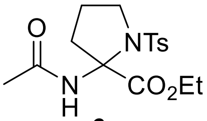

6a

E_P = +1.78 V
v = 100 mV/s
c = 3 mM
Solvent: MeCN/H_2O, 5:1
Start point = 0.0 V, scanned in positive direction

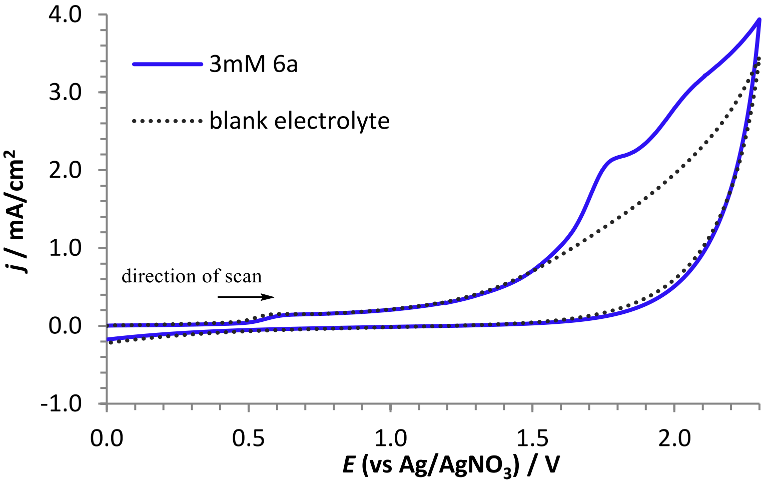

Synthetic transformations of compounds 6a,b,d,e

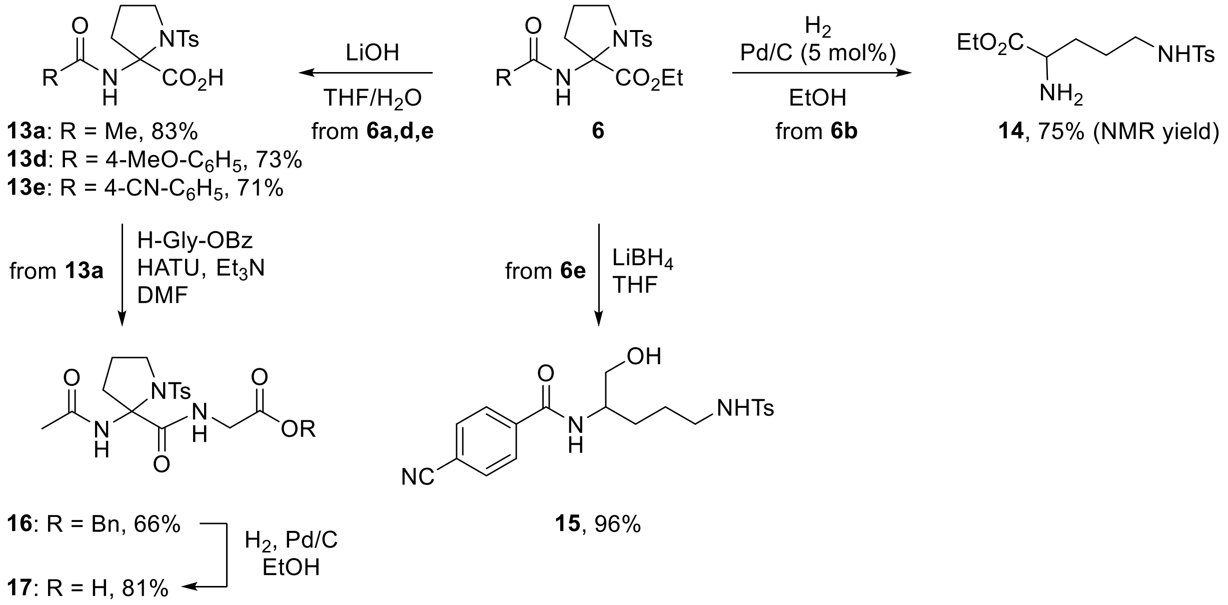

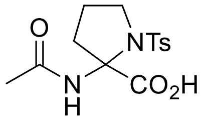

2-Acetamido-1-(4-methylbenzenesulfonyl)pyrrolidine-2-carboxylic acid (13a). To a solution of pyrrolidine **6a** (126 mg, 0.36 mmol, 1 equiv) in THF (1.2 mL) was added a solution of LiOH×H_2O (18 mg, 0.42 mmol, 1.5 equiv) in water (2.5 mL). The colorless solution was stirred at room temperature for 16 hours, THF was removed *in vacuo,* and aqueous 1M HCl solution was added until pH ~4. The resulting white suspension was diluted with water (10 mL) and extracted with EtOAc (3×8 mL). The organic layers were combined, dried over anhydrous Na_2SO_4, filtered and concentrated. The crude residue as purified by reversed phase flash column chromatography using gradient elution from 5% to 100% MeCN in water containing 0.01% TFA to afford the title compound (97 mg, 83%) as a white amorphous solid.

^{1}H NMR (300 MHz, CD_3OD) δ 8.02 (s, 1H), 7.70 – 7.62 (m, 2H), 7.37 – 7.30 (m, 2H), 3.78 – 3.63 (m, 2H), 2.80 – 2.67 (m, 1H), 2.42 (s, 3H), 2.26 – 2.07 (m, 3H), 1.74 (s, 3H).

^{13}C NMR (75 MHz, DMSO-*d*$_6$) δ 172.5, 168.8, 142.6, 136.9, 129.3, 126.8, 76.6, 49.3, 36.6, 24.1, 23.2, 21.0.

HRMS (ESI/Q-TOF) *m/z*: $[M+Na]^+$ Calcd for $C_{14}H_{18}N_2O_5SNa$ 349.0834; Found 349.0825.

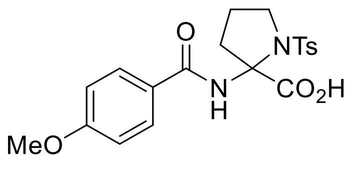

2-(4-Methoxybenzamido)-1-(4-methylbenzenesulfonyl)pyrrolidine-2-carboxylic acid (13d). To a solution of pyrrolidine **6d** (126 mg, 0.28 mmol, 1 equiv) in THF (1.2 mL) was added a solution of LiOH×H_2O (22 mg, 0.53 mmol, 1.5 equiv) in water (0.45 mL). The colorless solution was stirred at room temperature for 14 hours, THF was removed *in vacuo* and aqueous 1M HCl solution was

added until pH ~4. The resulting white suspension was diluted with water (10 mL) and extracted with EtOAc (4×8 mL). The organic layers were combined, dried over anhydrous Na_2SO_4, filtered and concentrated *in vacuo* to obtain the title compound as a white amorphous solid (85 mg, 73%).

^{1}H NMR (300 MHz, DMSO-d_6) δ 7.81 (s, 1H), 7.51 – 7.43 (m, 4H), 7.09 – 7.03 (m, 2H), 7.03 – 6.97 (m, 2H), 3.82 (s, 3H), 3.71 – 3.60 (m, 2H), 2.77 – 2.64 (m, 1H), 2.29 – 2.19 (m, 1H), 2.26 (s, 3H), 2.18 – 1.99 (m, 1H).

^{13}C NMR (75 MHz, DMSO-d_6) δ 172.8, 163.9, 162.0, 142.6, 136.5, 129.2, 128.6, 126.7, 125.9, 113.7, 76.5, 55.4, 49.5, 36.7, 24.1, 20.9.

HRMS (ESI/Q-TOF) *m*/*z*: $[M+Na]^+$ Calcd for $C_{20}H_{22}N_2O_6SNa$ 441.1096; Found 441.1096.

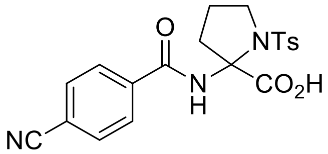

2-(4-Cyanobenzamido)-1-(4-methylbenzenesulfonyl)pyrrolidine-2-carboxylic acid (13e). To a solution of pyrrolidine **6e** (50 mg, 0.11 mmol, 1 equiv) in THF (4 mL) was added a solution of LiOH×H_2O (7 mg, 0.17 mmol, 1.5 equiv) in water (1 mL). The colorless solution was stirred at room temperature for 16 hours, THF was removed *in vacuo*, and aqueous 1M HCl solution was added until pH ~4. The resulting white suspension was diluted with water (6 mL) and extracted with EtOAc (3×6 mL). The organic layers were combined, dried over anhydrous Na_2SO_4, filtered and concentrated *in vacuo*. The crude residue was purified by using reversed phase flash column chromatography using gradient elution from 5% to 50% MeCN in water containing 0.01% TFA to afford the title compound (33 mg, 71%) as a white amorphous solid.

^{1}H NMR (300 MHz, CD_3OD) δ 8.36 (s, 1), 7.86 – 7.80 (m, 2H), 7.74 – 7.67 (m, 2H), 7.59 – 7.52 (m, 2H), 7.12 – 7.05 (m, 2H), 3.90 – 3.76 (m, 2H), 2.84 (dt, *J* = 12.8, 8.9 Hz, 1H), 2.43 – 2.17 (m, 3H), 2.31, s, 3H).

^{13}C NMR (75 MHz, CD_3OD) δ 174.2, 165.8, 144.7, 139.1, 138.3, 133.5, 130.5, 129.1, 128.2, 119.0, 116.5, 79.5, 51.2, 38.3, 25.6, 21.4.

HRMS (ESI/Q-TOF) *m*/*z*: $[M-H]^-$ Calcd for $C_{20}H_{18}N_3O_5S$ 412.0967; Found 412.0980.

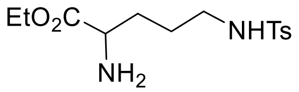

Ethyl 2-amino-5-(4-methylbenzenesulfonamido)pentanoate (14). Aminal **6b** (65 mg, 0.16 mmol, 1 equiv) was dissolved in EtOH (2.5 mL) and 10% Pd/C (8 mg, 0.007 mmol, 5 mol%) was added under argon. The resulting black suspension was flushed with argon, followed by attachment of a balloon filled with H_2. Hydrogen gas was bubbled through the reaction mixture for 30 s and the reaction was stirred under H_2 atmosphere for 2 hours. Filtration through a syringe filter (25 mm, 0.45 μm PTFE hydrophobic filter) followed by the

concentration of the filtrate *in vacuo* afforded the title compound as light-yellow oil (75% NMR yield; CH_2Br_2 was used as an internal standard).

^{1}H NMR (300 MHz, CD_3OD) δ 7.78 – 7.67 (m, 2H), 7.43 – 7.34 (m, 2H), 4.29 (q, *J* = 7.1 Hz, 2H), 4.00 (t, *J* = 6.5 Hz, 1H), 2.87 (t, *J* = 6.6 Hz, 2H), 2.43 (s, 3H), 2.06 – 1.87 (m, 2H), 1.80 – 1.49 (m, 2H), 1.31 (t, *J* = 7.1 Hz, 3H).

^{13}C NMR (75 MHz, CD_3OD) δ 170.4, 144.7, 138.6, 130.8, 128.0, 63.6, 53.6, 43.1, 28.8, 26.4, 21.5, 14.4.

HRMS (ESI/Q-TOF) *m*/*z*: $[M+H]^+$ Calcd for $C_{14}H_{22}N_2O_4S$ 315.1379; Found 315.1393.

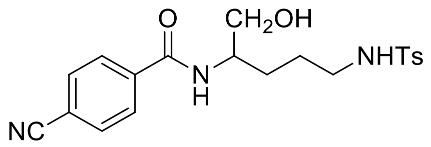

4-Cyano-*N*-[1-hydroxy-5-(4-methylbenzenesulfonamido)pentan-2-yl]benzamide (15). $LiBH_4$ (9 mg, 0.4 mmol, 4 equiv) was added to a solution of pyrrolidine **6e** (43 mg, 0.1 mmol, 1 equiv) in anhydrous THF (2 mL). The colorless solution was stirred at room temperature for 2 hours whereupon water (90 mL) and CH_2Cl_2 (90 mL) were added. The organic phase was separated, dried over anhydrous Na_2SO_4, filtered and concentrated *in vacuo* to afford the title product as a white amorphous solid (38 mg, 96%).

^{1}H NMR (300 MHz, CD_3OD) δ 8.00 – 7.90 (m, 2H), 7.88 – 7.78 (m, 2H), 7.75 – 7.65 (m, 2H), 7.39 – 7.29 (m, 2H), 4.13 – 3.99 (m, 1H), 3.57 (dd, *J* = 5.6, 1.3 Hz, 2H), 2.87 (t, *J* = 6.6 Hz, 2H), 2.39 (s, 3H), 1.75 – 1.61 (m, 1H), 1.60 – 1.45 (m, 3H).

^{13}C NMR (75 MHz, CD_3OD) δ 168.6, 144.5, 140.1, 139.0, 133.4, 130.7, 129.3, 128.0, 119.1, 115.9, 65.0, 53.1, 43.8, 29.1, 27.4, 21.4.

HRMS (ESI/Q-TOF) *m*/*z*: $[M+H]^+$ Calcd for $C_{20}H_{24}N_3O_5S$ 402.1488; Found 402.1493.

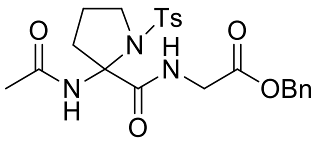

Benzyl 2-{[2-acetamido-1-(4-methylbenzenesulfonyl)-pyrrolidin-2-yl] formamido}acetate (16). A solution of carboxylic acid **13a** (40 mg, 0.12 mmol, 1 equiv) in DMF (1.5 mL) was cooled to 0 °C (crushed ice bath) and then sequentially treated with glycine benzyl ester hydrochloride (49 mg, 0.25 mmol, 2 equiv), HATU (47 mg, 0.12 mmol, 1 equiv), and Et_3N (51 µL, 0.37 mmol, 3 equiv). After warming to room temperature, the reaction was left to stir for 2 hours. The resulting light-yellow suspension was treated with saturated aqueous $NaHCO_3$ (10 mL) and extracted with EtOAc (15 mL). The organic layer was washed with brine (10 mL), separated, dried over Na_2SO_4, filtered, and concentrated *in vacuo*. Purification of the crude material by reversed phase flash column chromatography using gradient elution from 5% to 65% MeCN in water containing 0.01% TFA afforded 38 mg (66%) of the title compound as a colorless semisolid; analytical TLC on silica gel, 1:1 EtOAc/ petroleum ether, R_f = 0.18.

^{1}H NMR (300 MHz, $CDCl_3$) δ 7.68 – 7.57 (m, 2H), 7.41 – 7.31 (m, 5H), 7.31 – 7.22 (m, 3H), 6.97 (dd, *J* = 6.9, 4.2 Hz, 1H), 5.20 (s, 2H), 4.37 (dd, *J* = 6.9, 18.2 Hz, 1H), 3.98 (dd, *J* = 4.2, 18.2 Hz, 1H), 3.88 – 3.72 (m, 2H), 2.83 – 2.69 (m, 1H), 2.41 (s, 3H), 2.26 – 1.94 (m, 3H), 1.69 (s, 3H).

^{13}C NMR (75 MHz, $CDCl_3$) δ 172.4, 169.3, 169.0, 143.9, 135.4, 135.2, 129.5, 128.8, 128.8, 128.6, 127.7, 77.8, 67.5, 50.2, 42.0, 37.6, 24.6, 23.9, 21.7.

HRMS (ESI/Q-TOF) *m*/*z*: $[M+H]^+$ Calcd for $C_{23}H_{28}N_3O_6S$ 474.1699; Found 474.1700.

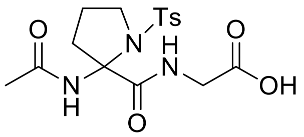

2-{[2-Acetamido-1-(4-methylbenzenesulfonyl)-pyrrolidin-2-yl]formamido}acetic acid (17). Protected acid **16** (20 mg, 0.04 mmol, 1 equiv) was dissolved in EtOH (1.5 mL) and 10% Pd/C (2 mg, 0.01 mmol, 25 mol%) was added under argon. The resulting black suspension was flushed with argon, followed by attachment of a balloon filled with H_2. Hydrogen gas was bubbled through the reaction mixture for 60 s and then the reaction was stirred under H_2 atmosphere for 1 hour. Filtration through a Celite plug followed by the filtrate concentration *in vacuo* afforded the title product as a colorless semisolid (13 mg, 81%).

^{1}H NMR (300 MHz, CD_3OD) δ 7.70 – 7.62 (m, 2H), 7.39 – 7.31 (m, 2H), 4.17 (d, *J* = 17.6 Hz, 1H), 3.88 – 3.64 (m, 3H), 2.82 – 2.63 (m, 1H), 2.43 (s, 3H), 2.39 – 2.04 (m, 3H), 1.72 (s, 3H).

^{13}C NMR (75 MHz, CD_3OD) δ 173.8, 172.7, 172.0, 145.1, 137.2, 130.5, 128.8, 79.2, 51.4, 39.2, 38.5, 24.9, 23.6, 21.5.

HRMS (ESI/Q-TOF) *m*/*z*: $[M+Na]^+$ Calcd for $C_{16}H_{21}N_3O_6SNa$ 406.1049; Found 406.1055.

References

[1] Liu, K.; Wang, G.; Zhang, Z.-W.; Shi, Y.-Y.; Ye, Z.-S. C–C Bond Activation of Cyclopropanes Enabled by Phosphine-Catalyzed *In Situ* Formation of High-Strain Methylenecycopropane Intermediate. *Org. Lett.* **2022**, *24*, 6489–6493.
https://doi.org/10.1021/acs.orglett.2c02201

[2] Zartman, C. B.; Bell, I. M.; Gallicchio, S. N.; Graham, S. L.; Kane, S. A.; Mallee, J. J.; Rutledge, R. Z.; Salvatore, C. A.; Vacca, J. P.; Williams, T. M. Identification of a Novel RAMP-Independent CGRP Receptor Antagonist. *Bioorganic & Medicinal Chemistry Letters* **2011**, *21*, 6705–6708.
https://doi.org/10.1016/j.bmcl.2011.09.056

[3] Li, X.; Jin, J.; Chen, P.; Liu, G. Catalytic Remote Hydrohalogenation of Internal Alkenes. *Nat. Chem.* **2022**, *14*, 425–432.
https://doi.org/10.1038/s41557-021-00869-x

[4] An, T.; Lee, Y. Nucleophilic Substitution at the Guanidine Carbon Center via Guanidine Cyclic Diimide Activation. *Org. Lett.* **2021**, *23*, 9163–9167.
https://doi.org/10.1021/acs.orglett.1c03473

[5] Segretti, M. C. F.; Vallerini, G. P.; Brochier, C.; Langley, B.; Wang, L.; Hancock, W. W.; Kozikowski, A. P. Thiol-Based Potent and Selective HDAC6 Inhibitors Promote Tubulin Acetylation and T-Regulatory Cell Suppressive Function. *ACS Med. Chem. Lett.* **2015**, *6*, 1156–1161.
https://doi.org/10.1021/acsmedchemlett.5b00303

[6] Koleda, O.; Prane, K.; Suna, E. Electrochemical Synthesis of Unnatural Amino Acids via Anodic Decarboxylation of *N* -Acetylamino Malonic Acid Derivatives. *Org. Lett.* **2023**, *25*, 7958–7962.
https://doi.org/10.1021/acs.orglett.3c02687

[7] Xin, B.-T.; De Bruin, G.; Verdoes, M.; Filippov, D. V.; Van Der Marel, G. A.; Overkleeft, H. S. Exploring Dual Electrophiles in Peptide-Based Proteasome Inhibitors: Carbonyls and Epoxides. *Org. Biomol. Chem.* **2014**, *12*, 5710–5718.
https://doi.org/10.1039/C4OB00893F

[8] Zhou, M.; Zhao, H.-Y.; Zhang, S.; Zhang, Y.; Zhang, X. Nickel-Catalyzed Four-Component Carbocarbonylation of Alkenes under 1 Atm of CO. *J. Am. Chem. Soc.* **2020**, *142*, 18191–18199.
https://doi.org/10.1021/jacs.0c08708

[9] Pavlishchuk, V. V.; Addison, A. W. Conversion Constants for Redox Potentials Measured versus Different Reference Electrodes in Acetonitrile Solutions at 25°C. *Inorganica Chimica Acta* **2000**, *298*, 97–102.
https://doi.org/10.1016/S0020-1693(99)00407-7

NMR Spectra

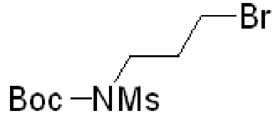

S2b

^{1}H NMR (300 MHz, $CDCl_3$)

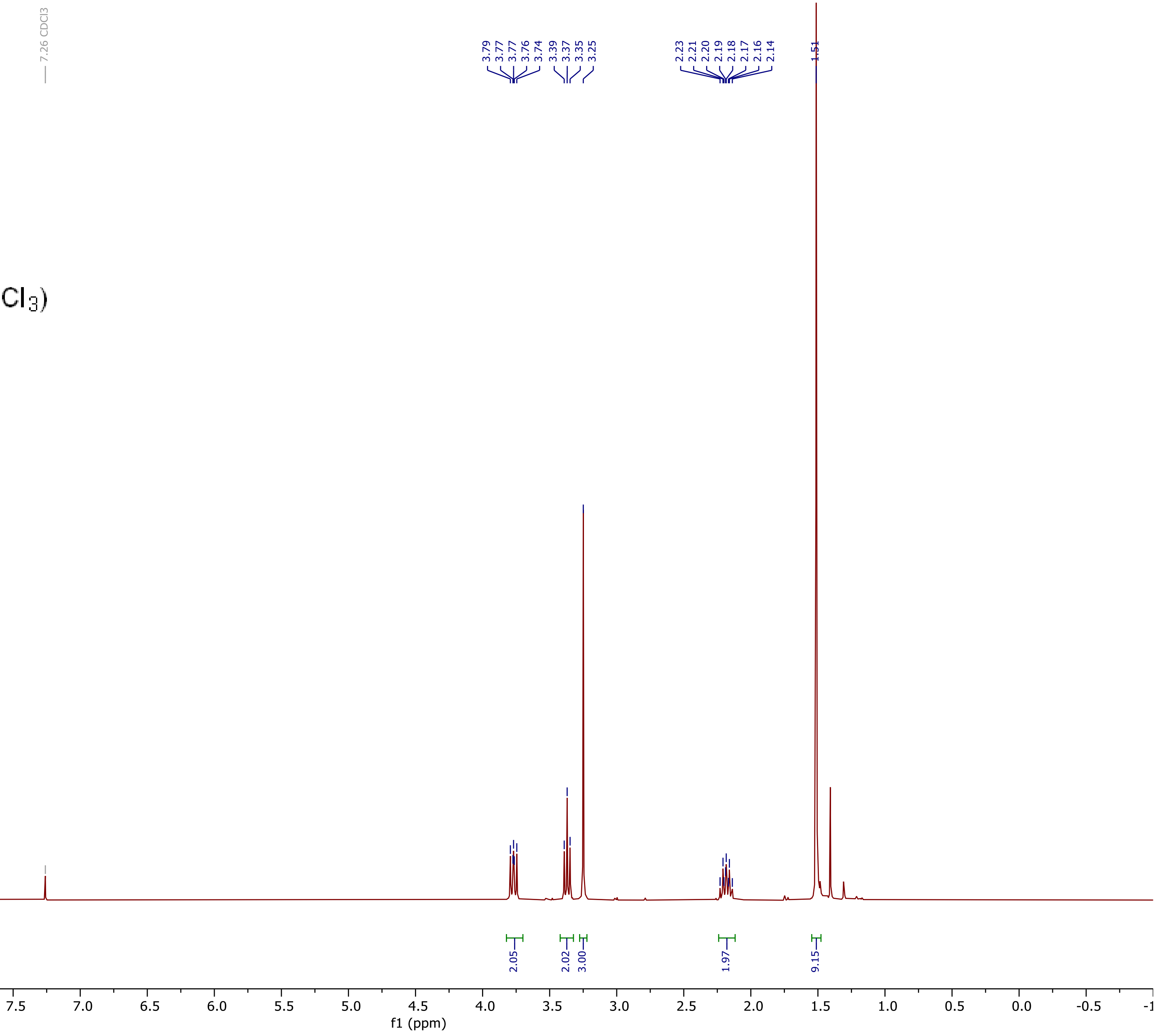

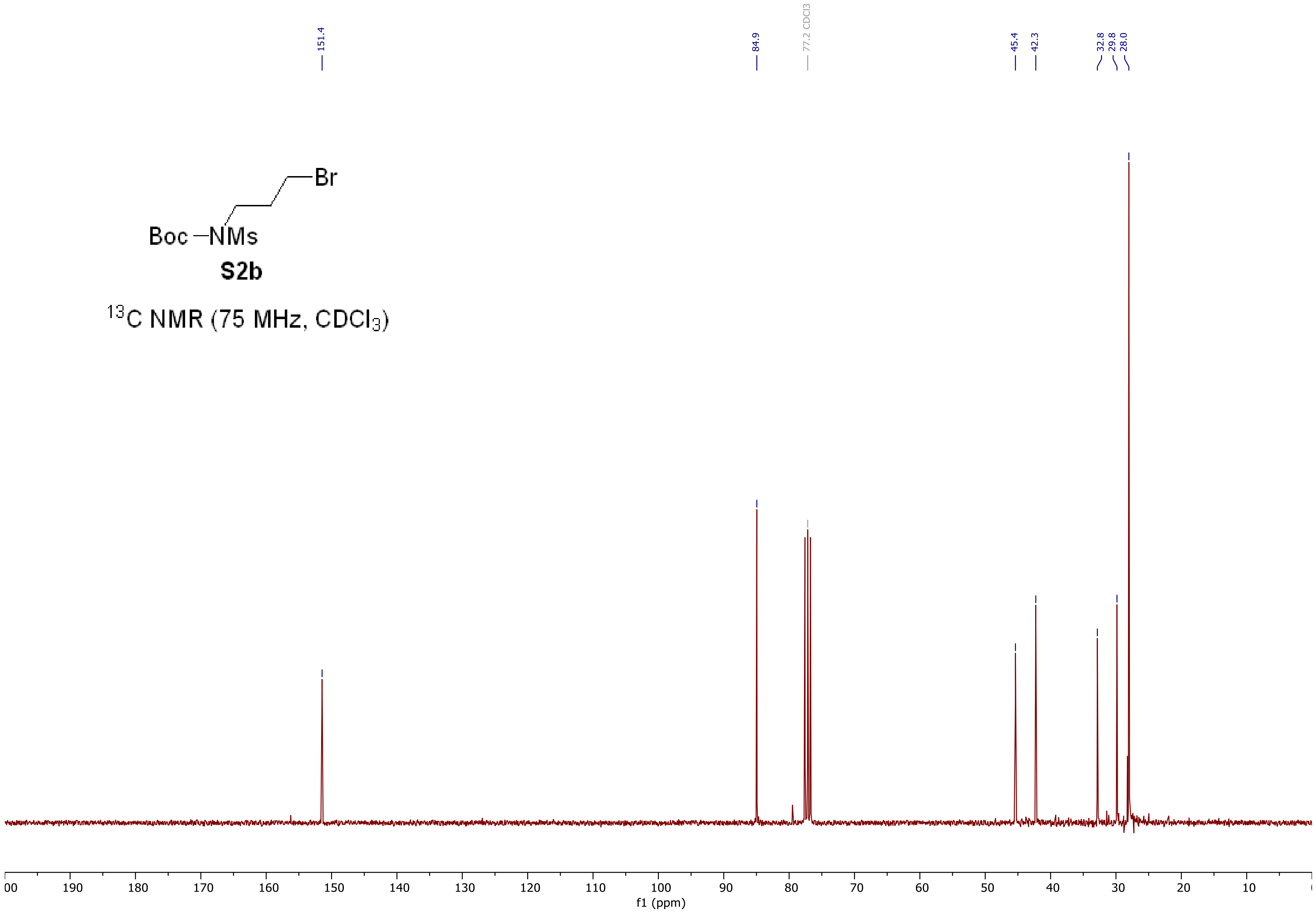

151.4
84.9
77.2 CDCl3
45.4
42.3
32.8
29.8
28.0
Br
Boc
NMs
S2b
13C NMR (75 MHz, CDCl3)
00
190
180
170
160
150
140
130
120
110
100
90
80
70
60
50
40
30
20
10
f1 (ppm)

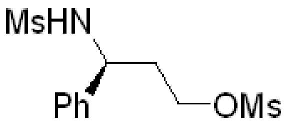

S4

^{1}H NMR (300 MHz, $CDCl_3$)

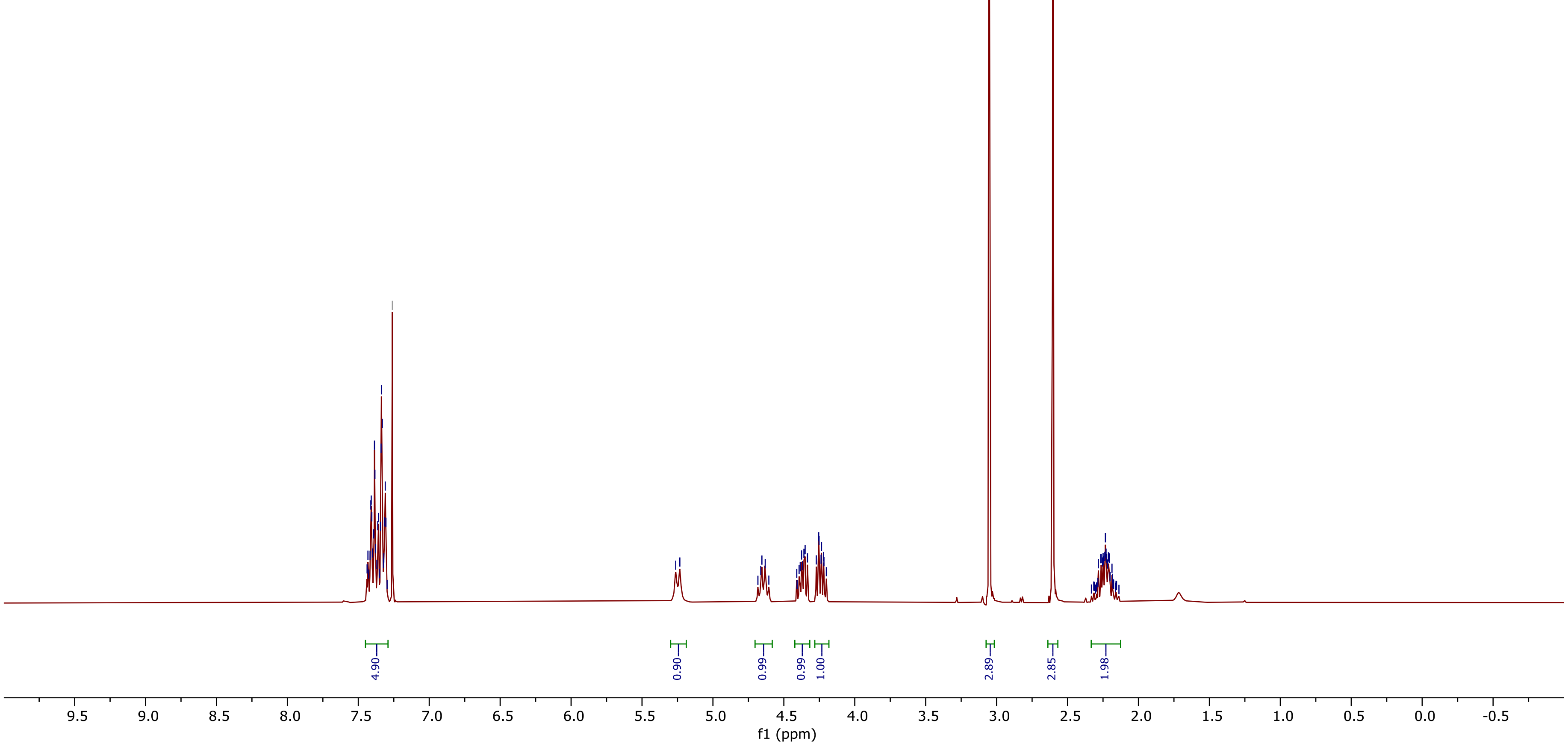

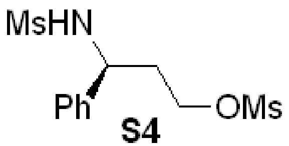

S4

^{13}C NMR (75 MHz, $CDCl_3$)

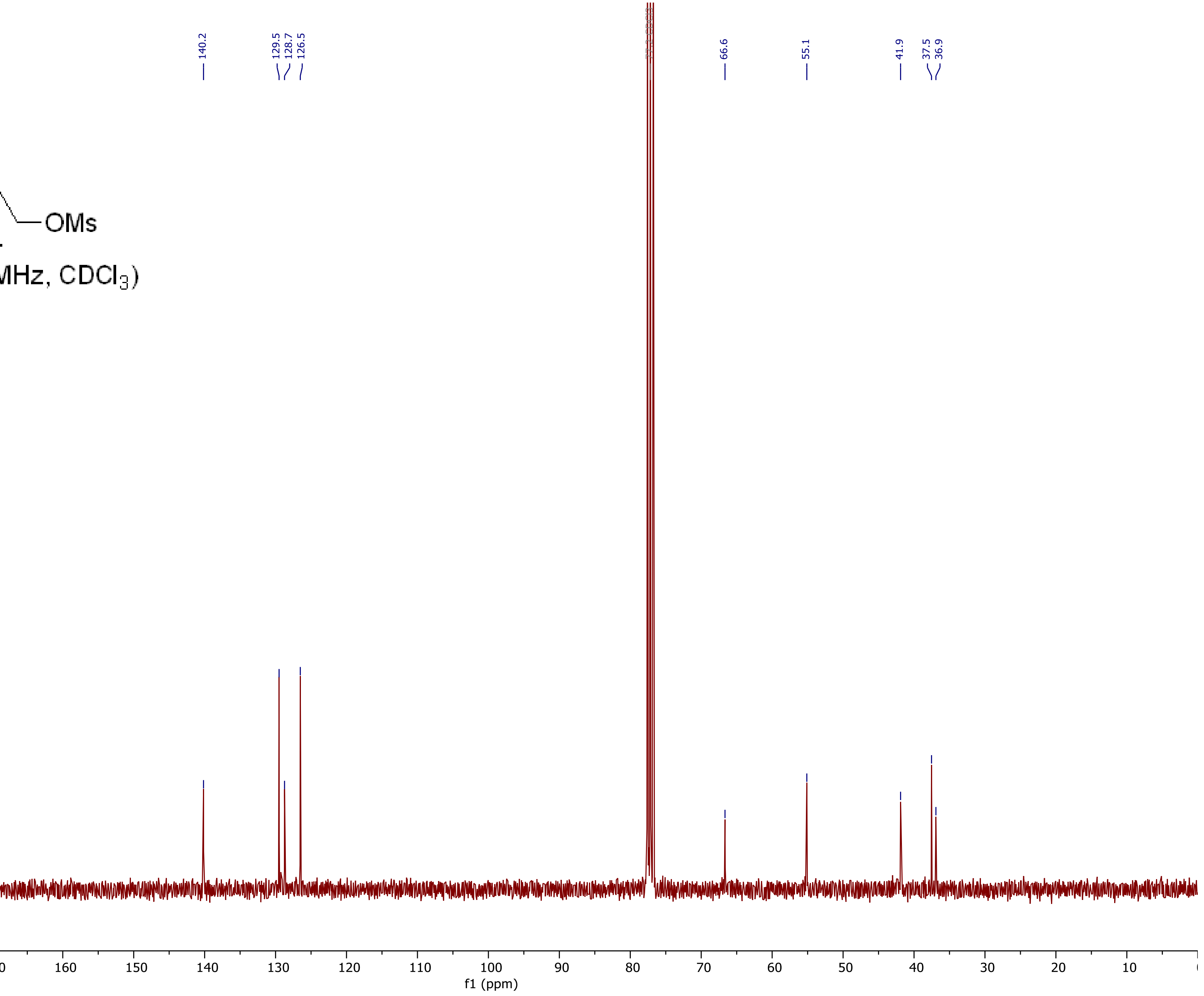

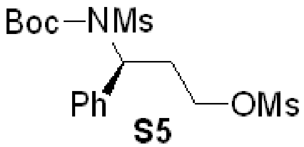

S5

^{1}H NMR (300 MHz, $CDCl_3$)

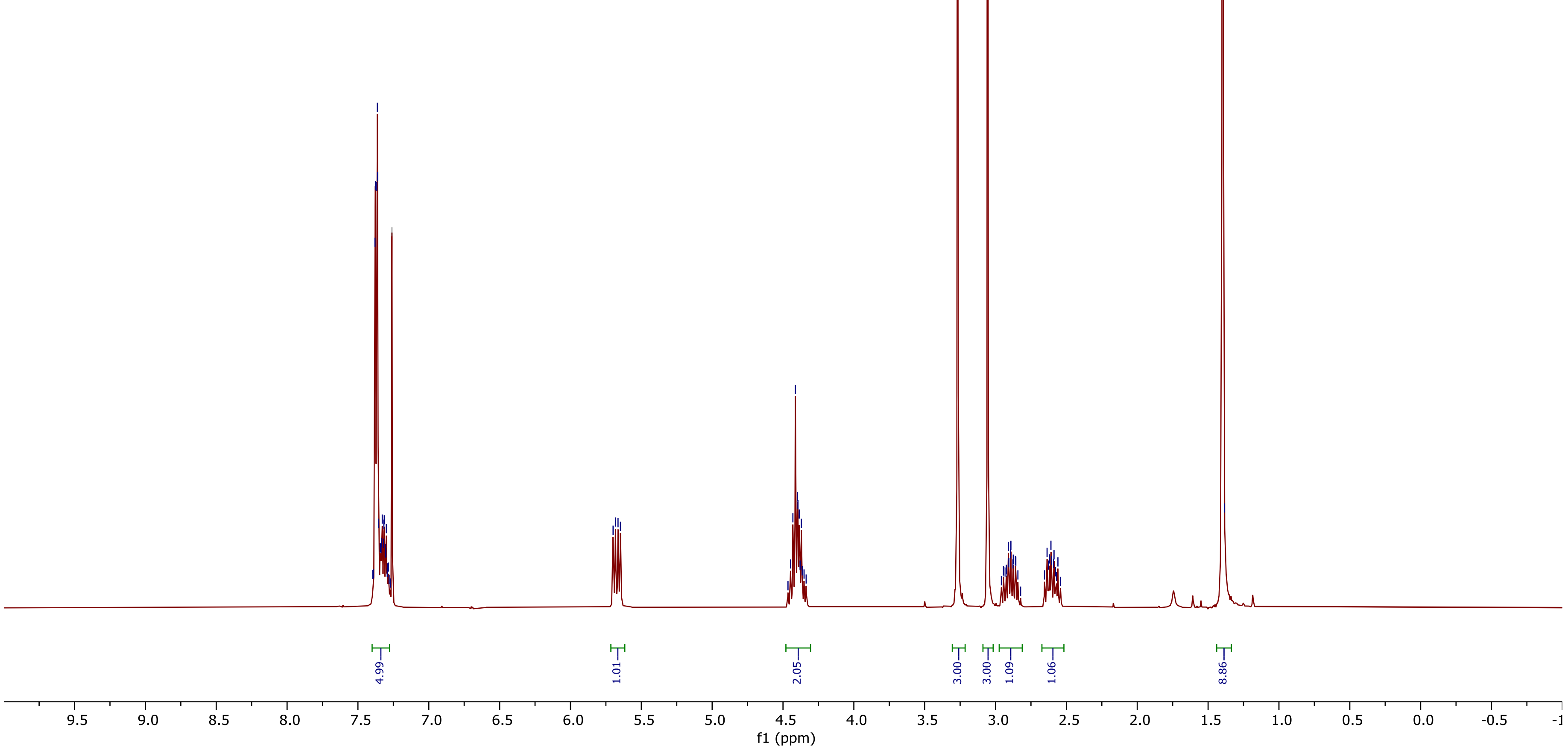

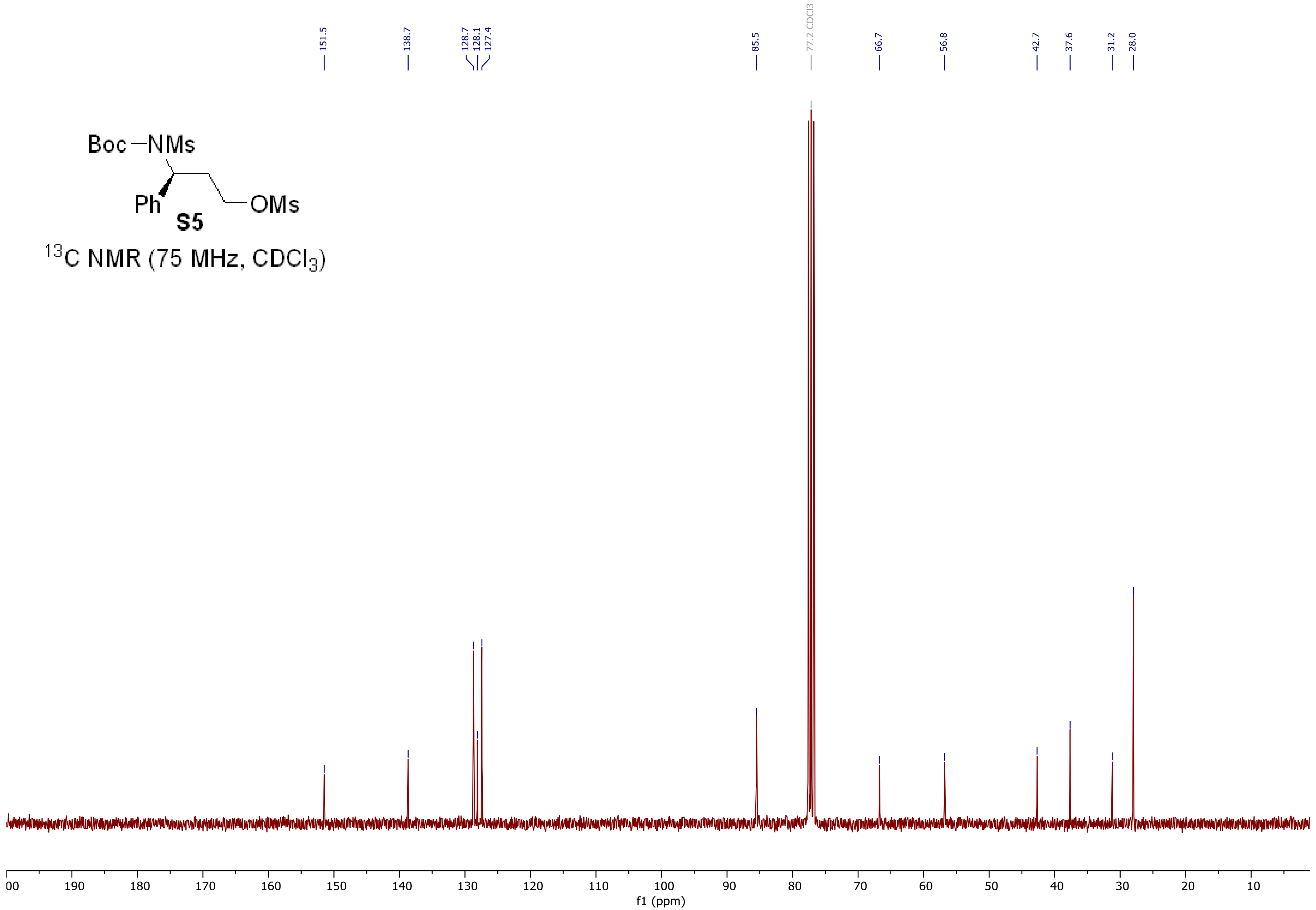

S5

^{13}C NMR (75 MHz, $CDCl_3$)

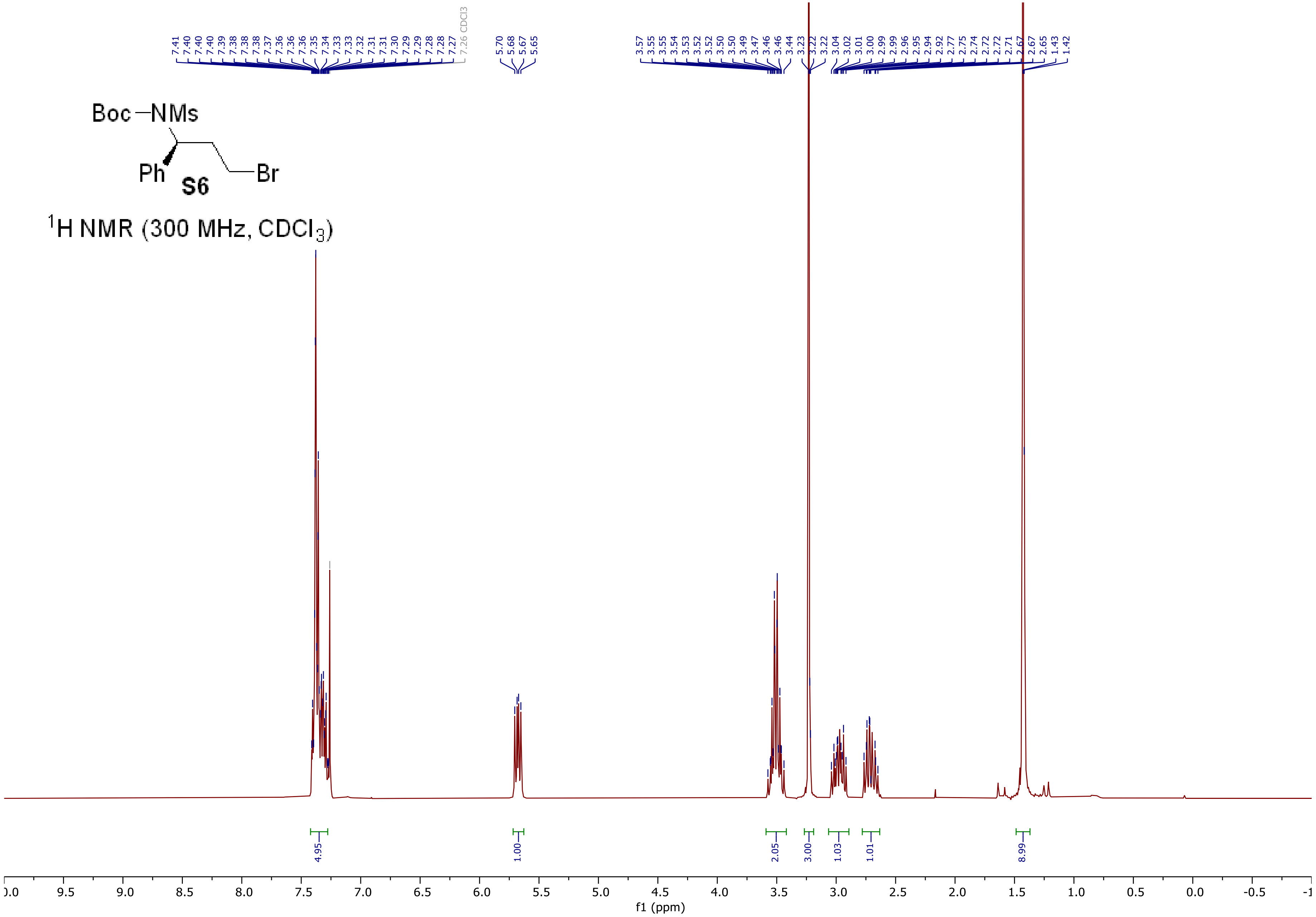
Boc—NMs
Ph
S6
Br
1H NMR (300 MHz, CDCl3)
f1 (ppm)

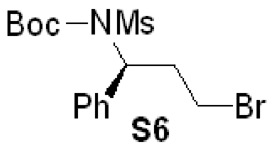

S6

^{13}C NMR (75 MHz, $CDCl_3$)

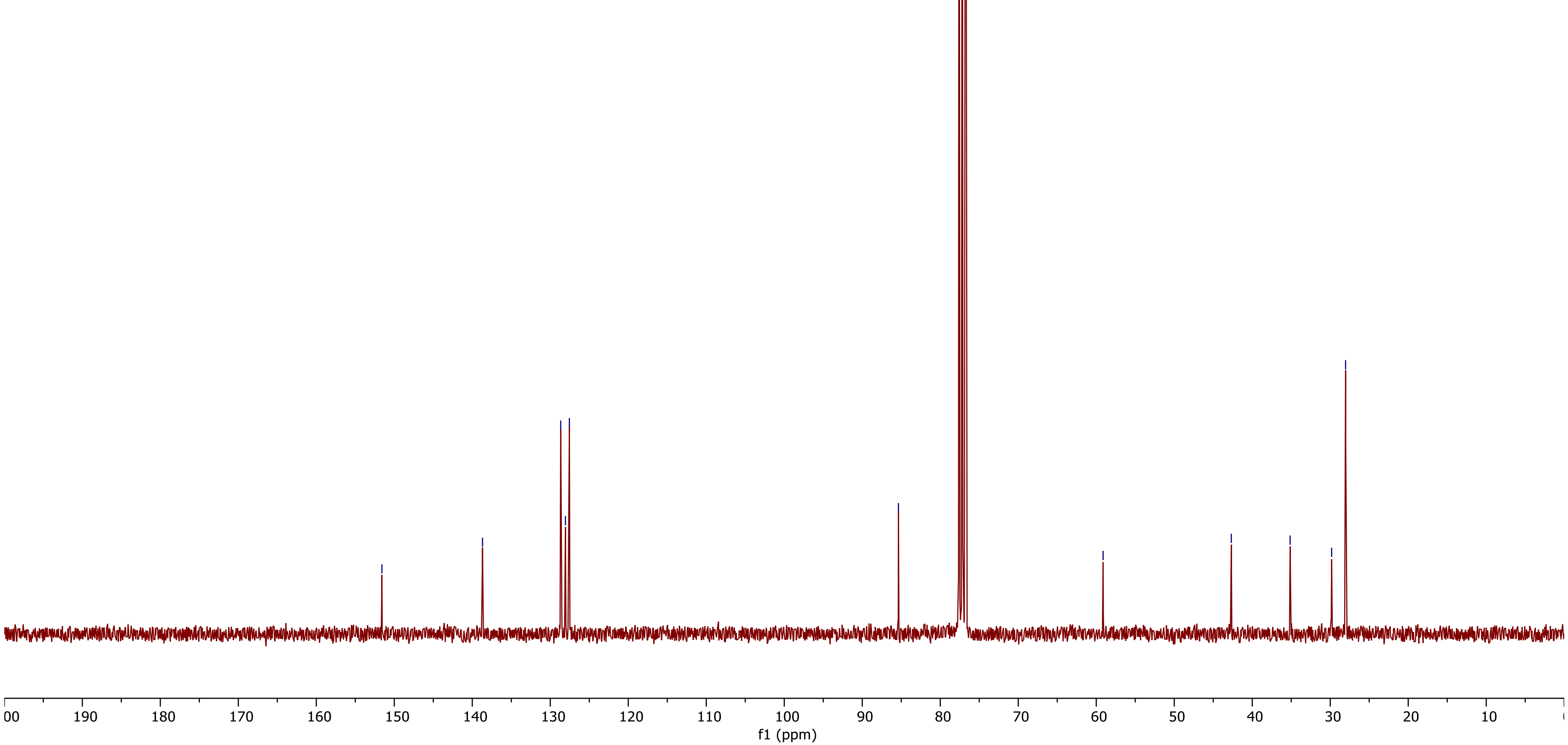

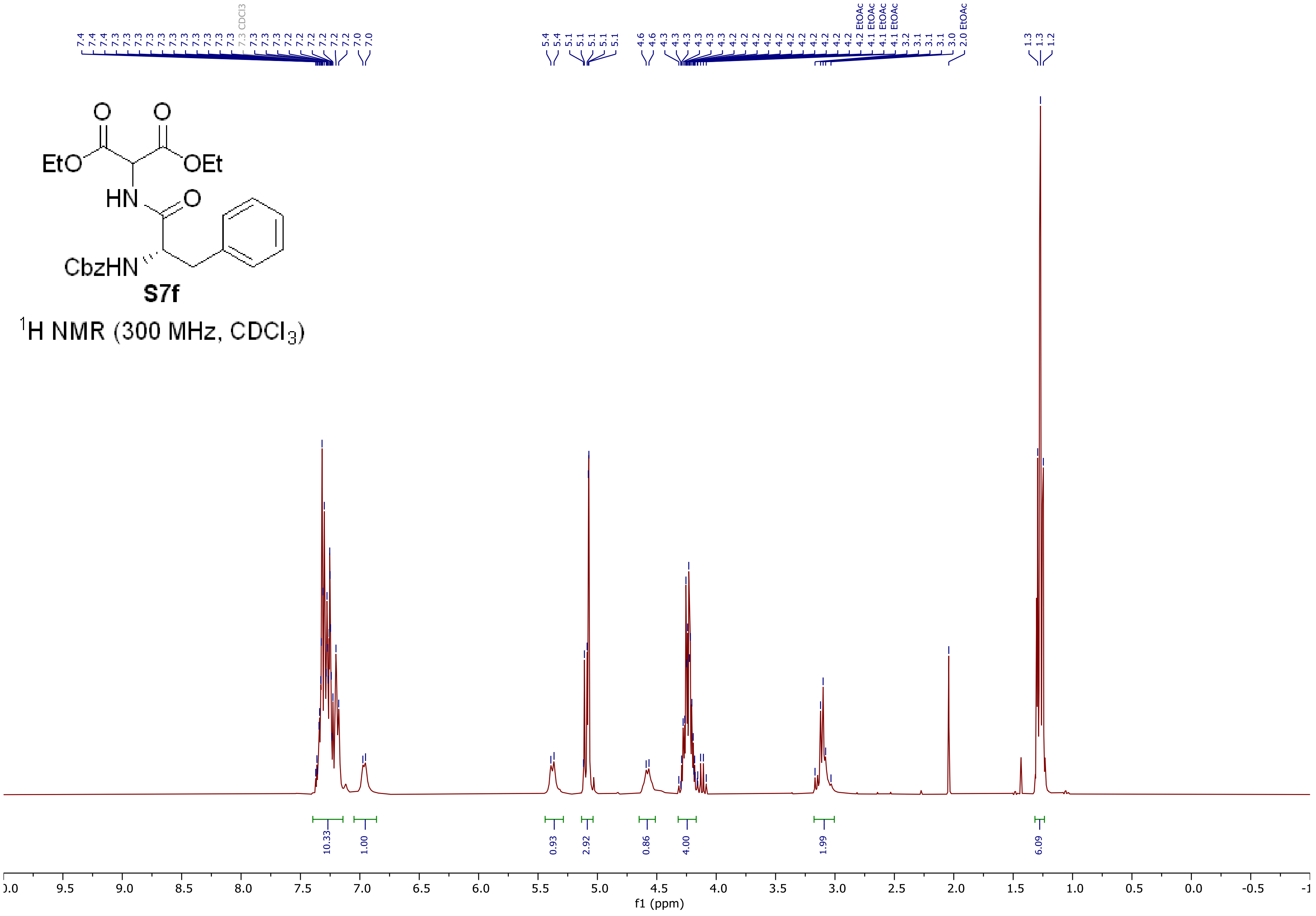
EtO
OEt
HN
CbzHN
S7f
^{1}H NMR (300 MHz, CDCl$_3$)
f1 (ppm)
10.33
1.00
0.93
2.92
0.86
4.00
1.99
6.09

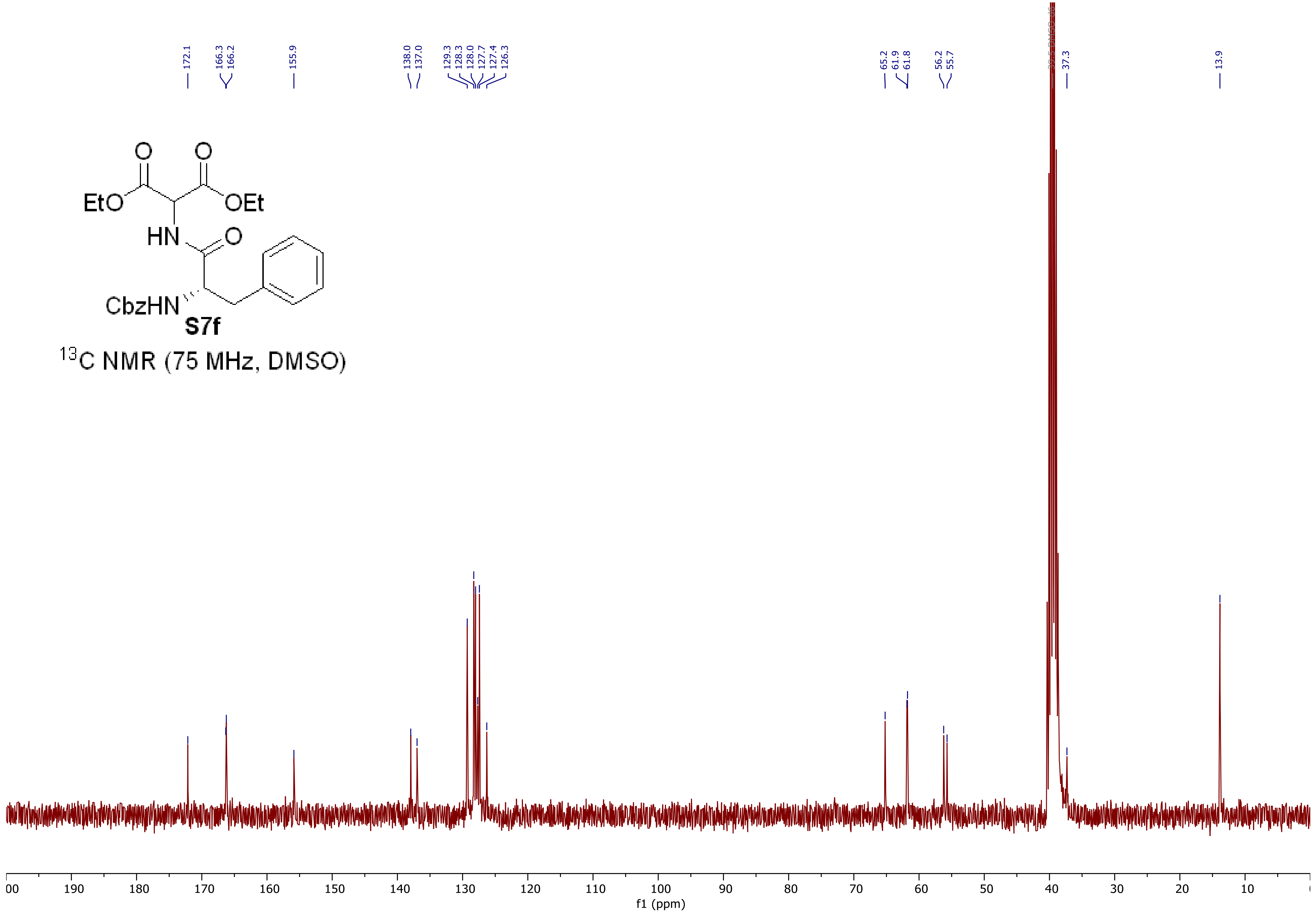
S7f

^{13}C NMR (75 MHz, DMSO)

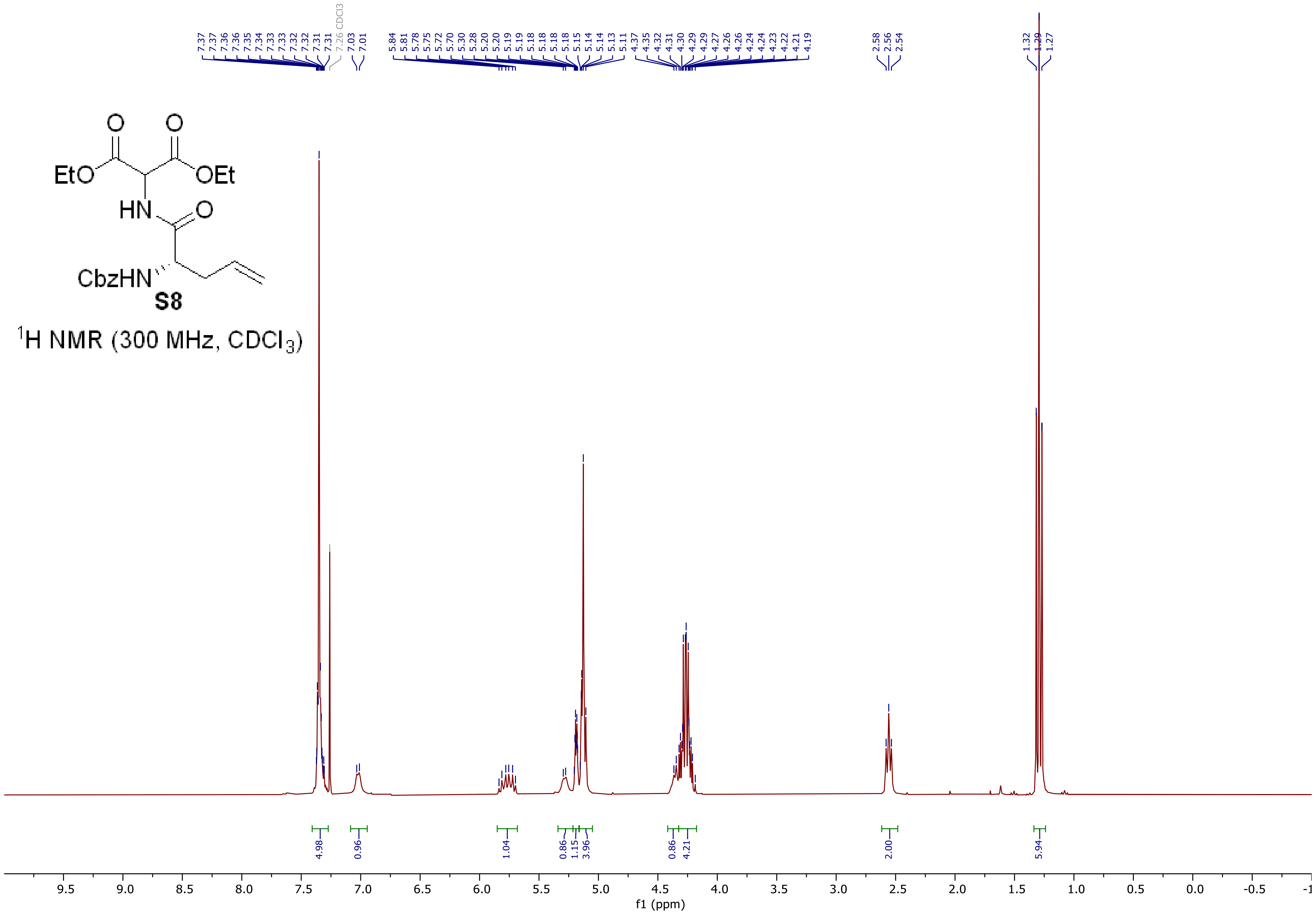
EtO
OEt
HN
CbzHN
S8
¹H NMR (300 MHz, CDCl₃)
f1 (ppm)

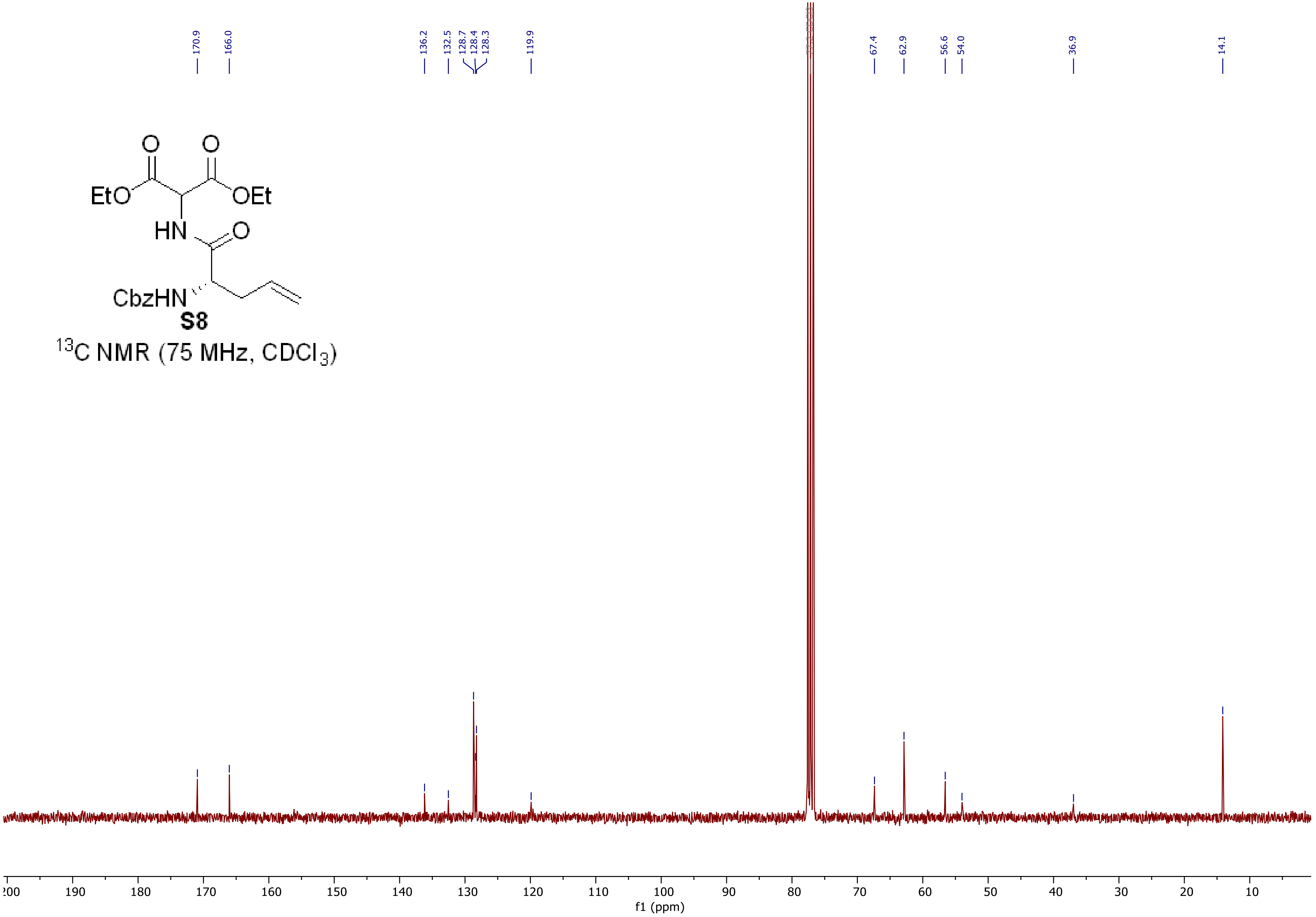
170.9
166.0
136.2
132.5
128.7
128.4
128.3
119.9
67.4
62.9
56.6
54.0
36.9
14.1
EtO
OEt
HN
O
CbzHN
S8
^{13}C NMR (75 MHz, $CDCl_3$)
200
190
180
170
160
150
140
130
120
110
100
90
80
70
60
50
40
30
20
10
f1 (ppm)

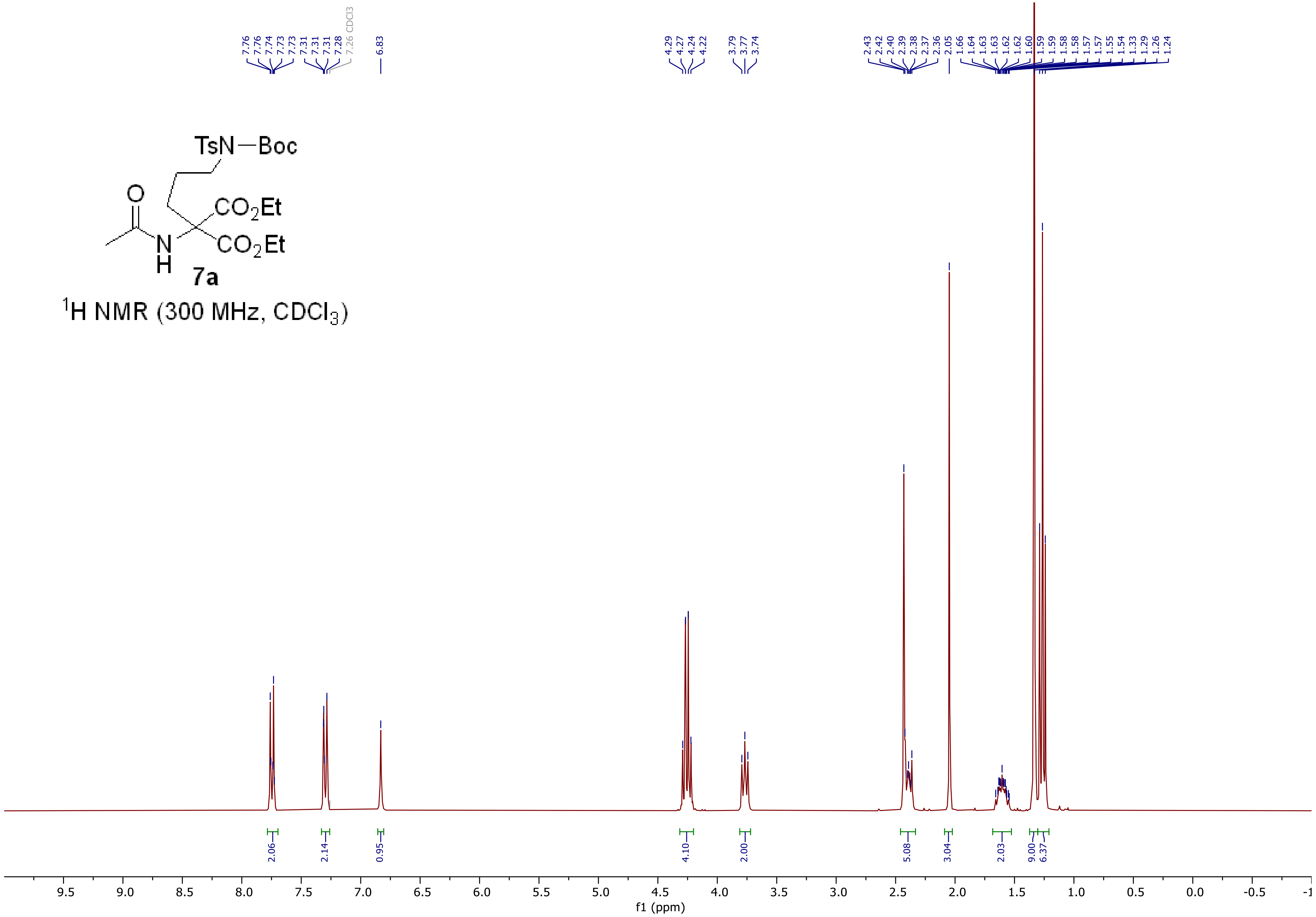
TsN—Boc
O
N
H
CO2Et
CO2Et
7a
1H NMR (300 MHz, CDCl3)
f1 (ppm)

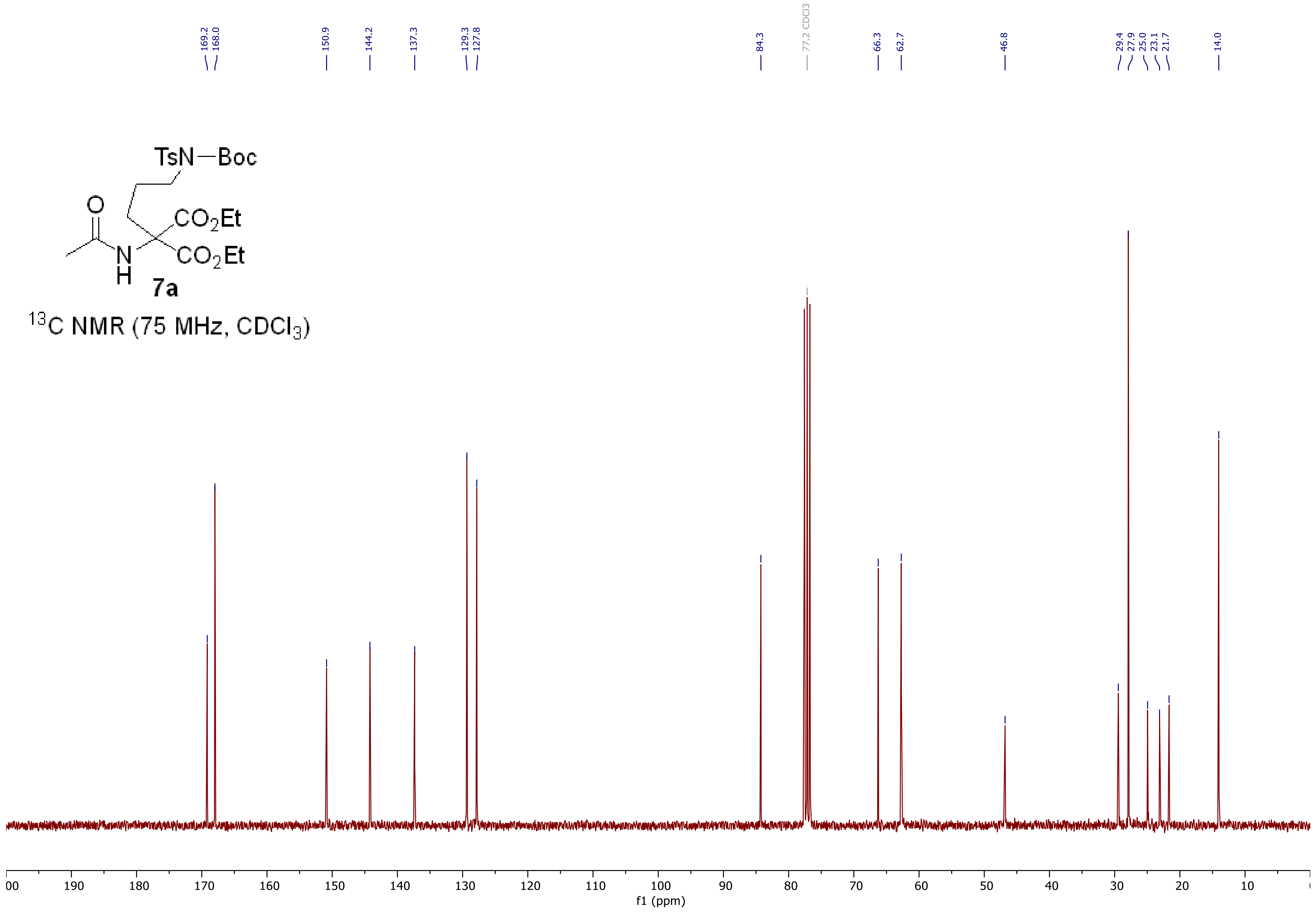
169.2
168.0
150.9
144.2
137.3
129.3
127.8
84.3
77.2 CDCl3
66.3
62.7
46.8
29.4
27.9
25.0
23.1
21.7
14.0
TsN—Boc
CO2Et
CO2Et
O
N
H
7a
13C NMR (75 MHz, CDCl3)
00
190
180
170
160
150
140
130
120
110
100
90
80
70
60
50
40
30
20
10
f1 (ppm)

7b

^{1}H NMR (300 MHz, $CDCl_3$)

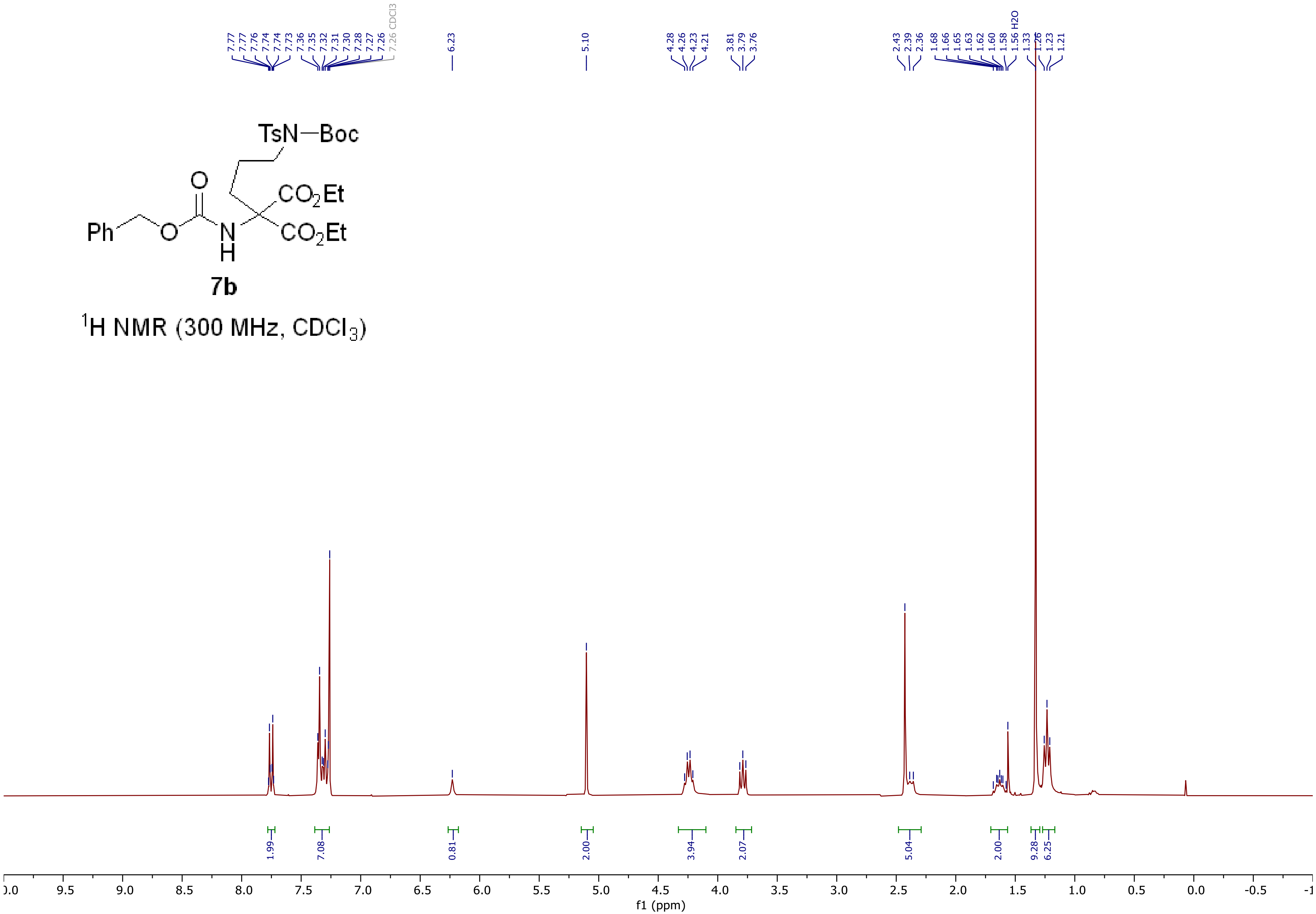

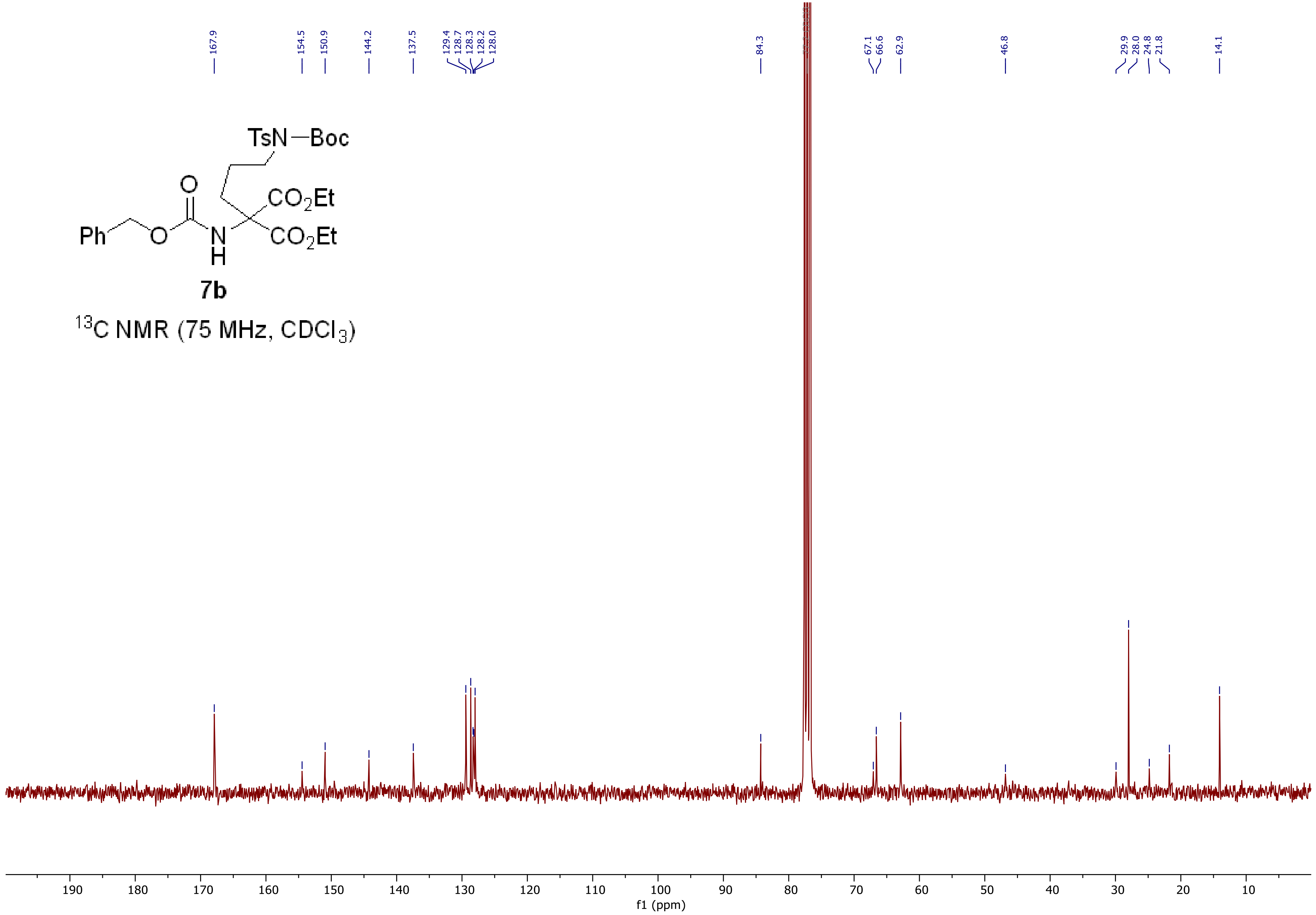

TsN—Boc
Ph
O
O
N
H
CO2Et
CO2Et
7b
13C NMR (75 MHz, CDCl3)
167.9
154.5
150.9
144.2
137.5
129.4
128.7
128.3
128.2
128.0
84.3
67.1
66.6
62.9
46.8
29.9
28.0
24.8
21.8
14.1
190
180
170
160
150
140
130
120
110
100
90
80
70
60
50
40
30
20
10
f1 (ppm)

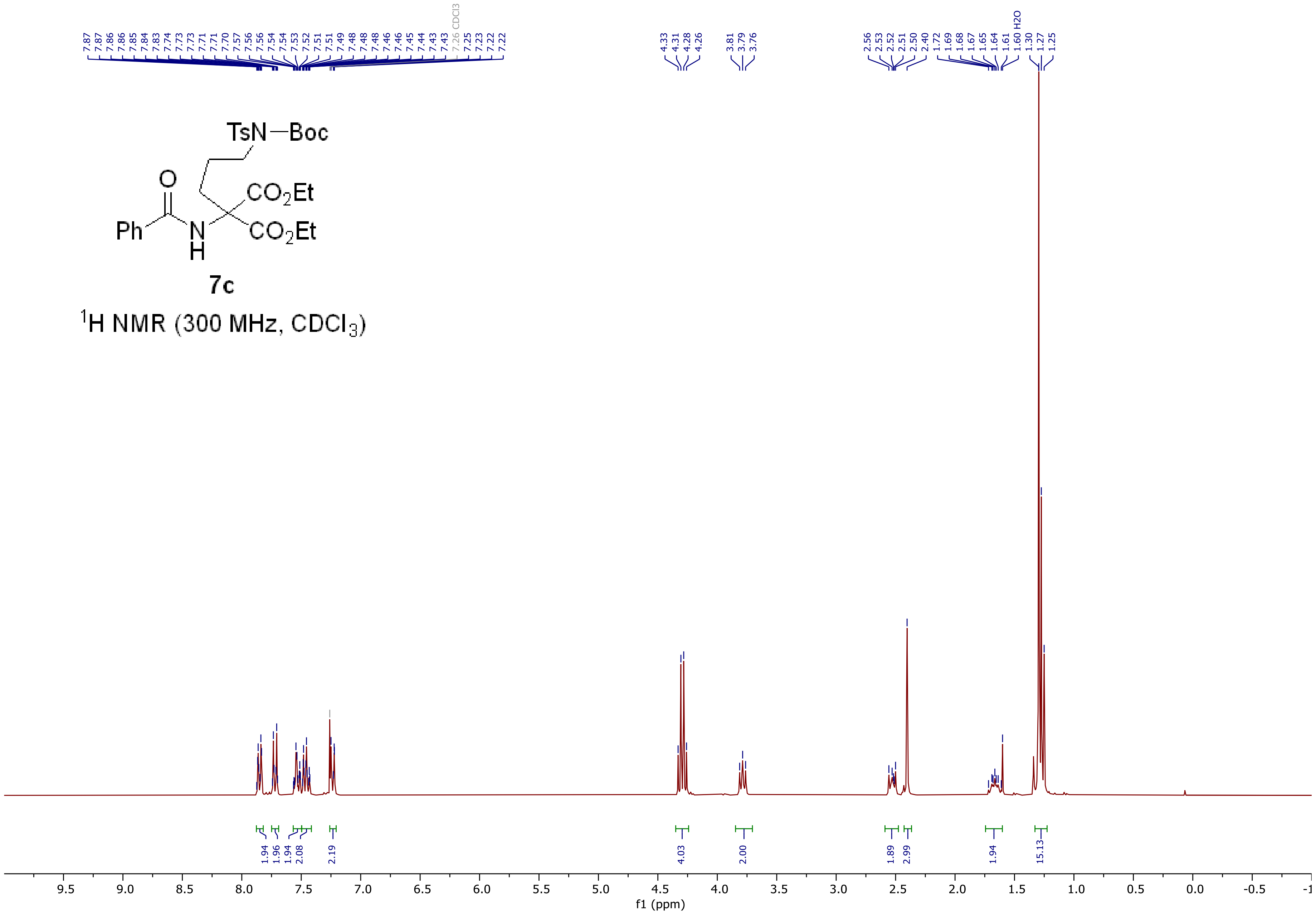

TsN—Boc
O
Ph
N
H
CO2Et
CO2Et
7c
1H NMR (300 MHz, CDCl3)
7.87 7.87 7.86 7.85 7.84 7.83 7.74 7.73 7.73 7.71 7.71 7.70 7.57 7.56 7.56 7.54 7.54 7.53 7.52 7.51 7.51 7.49 7.48 7.48 7.48 7.46 7.46 7.45 7.44 7.43 7.43 7.26 CDCl3 7.25 7.23 7.22 7.22
4.33 4.31 4.28 4.26
3.81 3.79 3.76
2.56 2.53 2.52 2.51 2.50 2.40 1.72 1.69 1.68 1.67 1.65 1.64 1.61 1.60 H2O 1.30 1.27 1.25
1.94 1.96 1.94 2.08 2.19 4.03 2.00 1.89 2.99 1.94 15.13
9.5 9.0 8.5 8.0 7.5 7.0 6.5 6.0 5.5 5.0 4.5 4.0 3.5 3.0 2.5 2.0 1.5 1.0 0.5 0.0 -0.5
f1 (ppm)

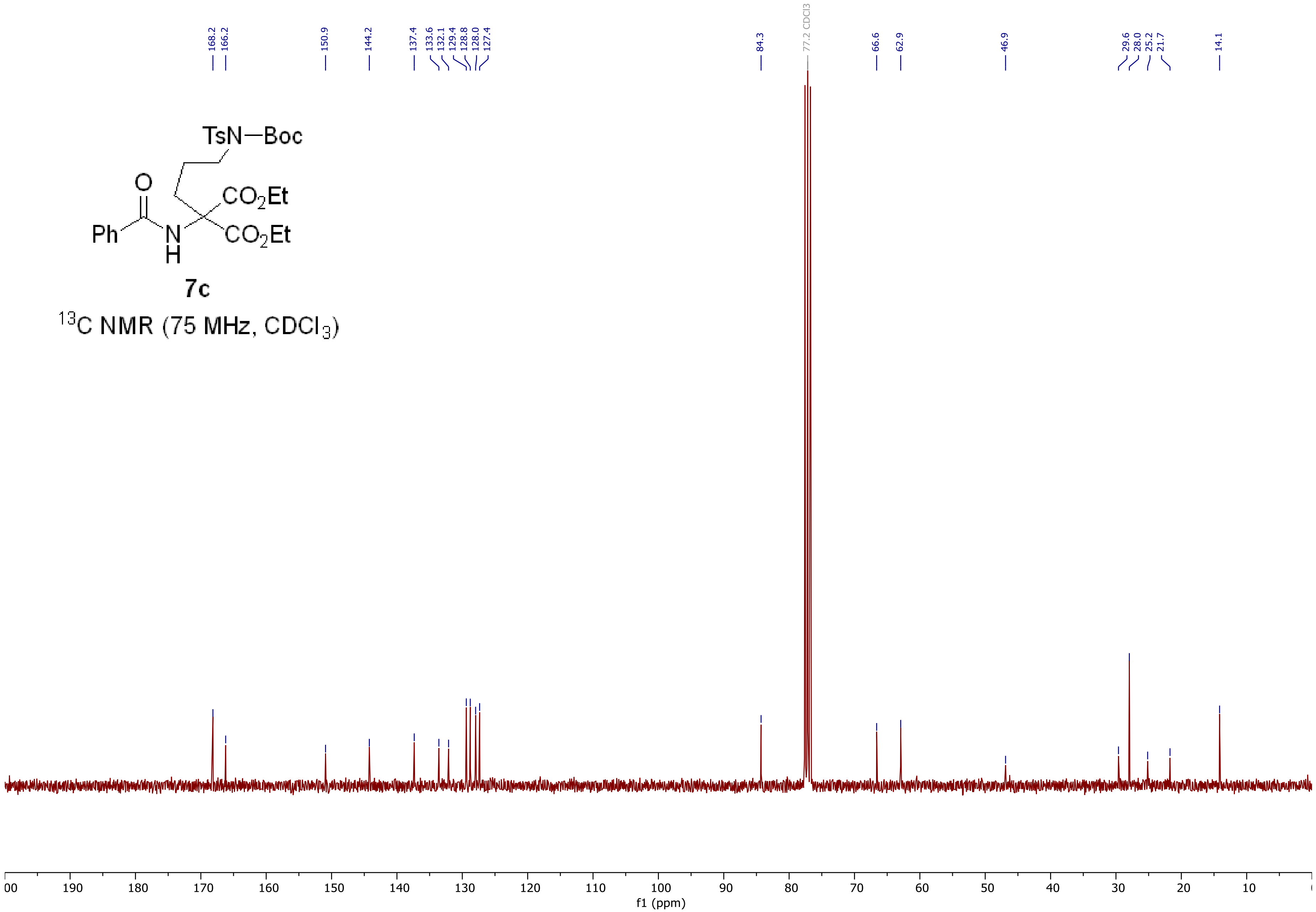

TsN—Boc
Ph
N
H
CO_2Et
CO_2Et
7c
^{13}C NMR (75 MHz, $CDCl_3$)
168.2
166.2
150.9
144.2
137.4
133.6
132.1
129.4
128.8
128.0
127.4
84.3
77.2 CDCl3
66.6
62.9
46.9
29.6
28.0
25.2
21.7
14.1
190
180
170
160
150
140
130
120
110
100
90
80
70
60
50
40
30
20
10
f1 (ppm)

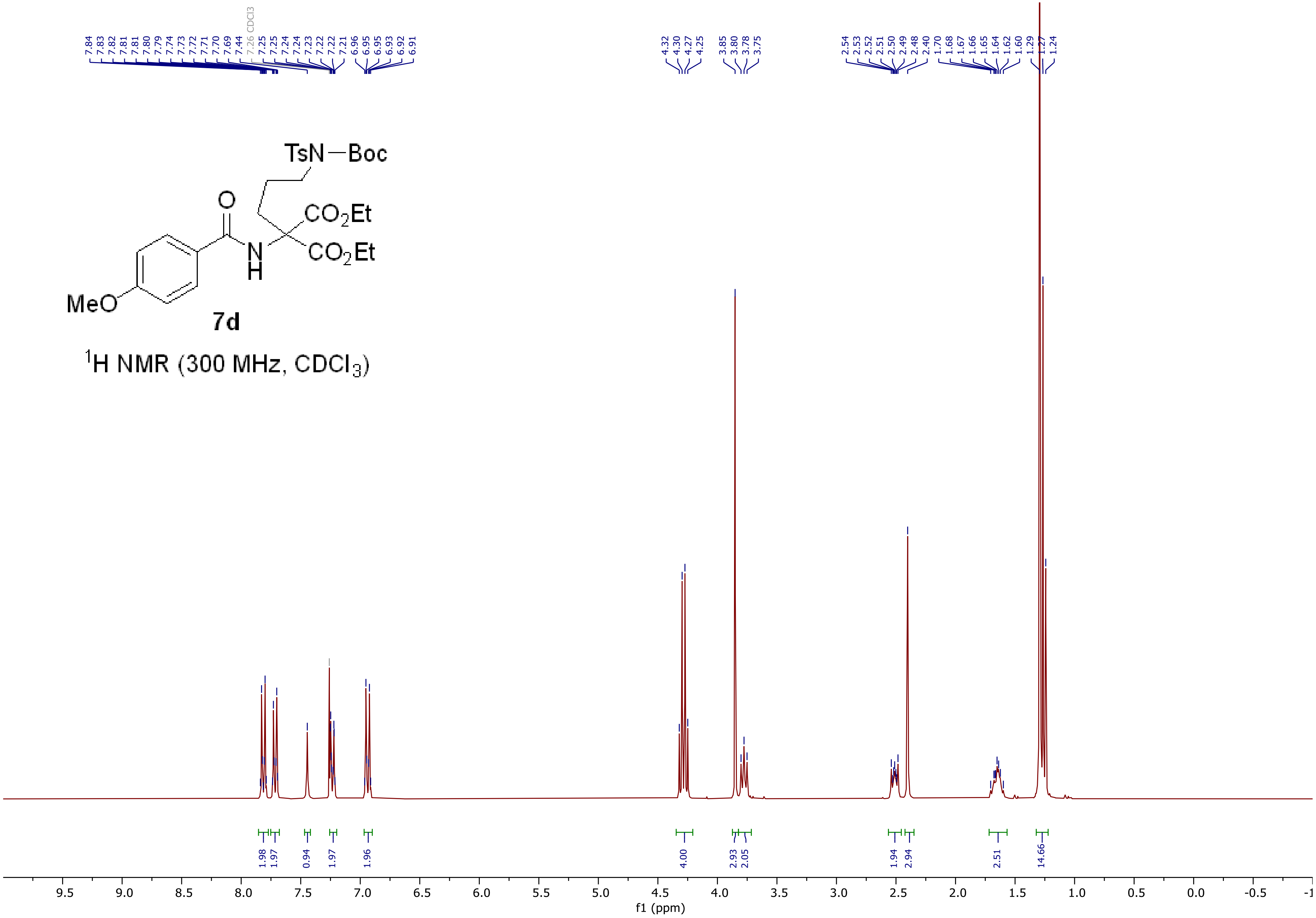
TsN—Boc
O
CO2Et
CO2Et
N
H
MeO
7d
1H NMR (300 MHz, CDCl3)
7.84
7.83
7.82
7.81
7.81
7.80
7.79
7.74
7.73
7.72
7.71
7.70
7.69
7.44
7.26 CDCl3
7.25
7.25
7.24
7.24
7.23
7.22
7.22
7.21
6.96
6.95
6.95
6.93
6.92
6.91
4.32
4.30
4.27
4.25
3.85
3.80
3.78
3.75
2.54
2.53
2.52
2.51
2.50
2.49
2.48
2.40
1.70
1.68
1.67
1.66
1.65
1.64
1.62
1.60
1.29
1.27
1.24
1.98
1.97
0.94
1.97
1.96
4.00
2.93
2.05
1.94
2.94
2.51
14.66
f1 (ppm)

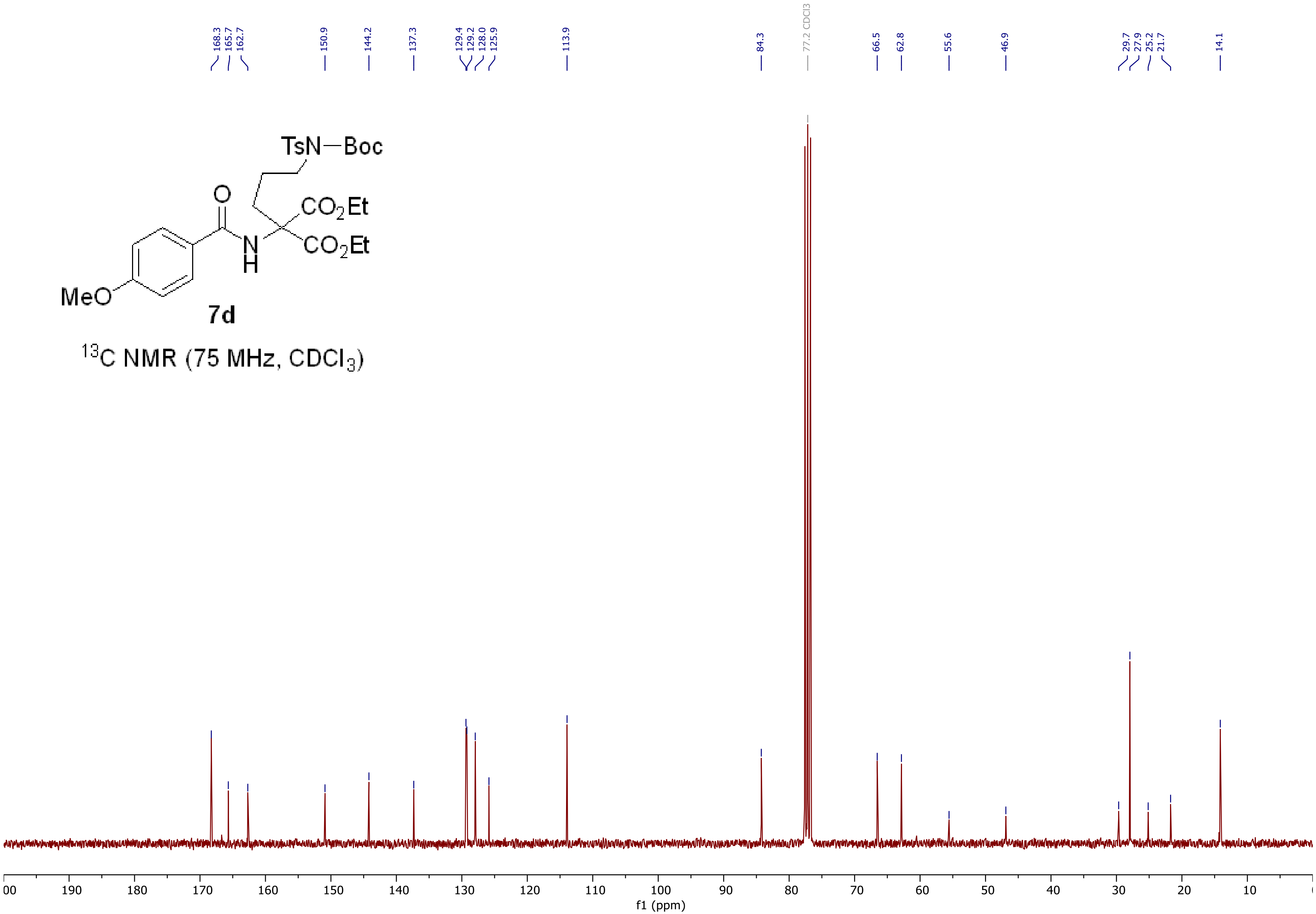
TsN—Boc
O
CO2Et
CO2Et
N
H
MeO
7d
13C NMR (75 MHz, CDCl3)
168.3
165.7
162.7
150.9
144.2
137.3
129.4
129.2
128.0
125.9
113.9
84.3
77.2 CDCl3
66.5
62.8
55.6
46.9
29.7
27.9
25.2
21.7
14.1
f1 (ppm)

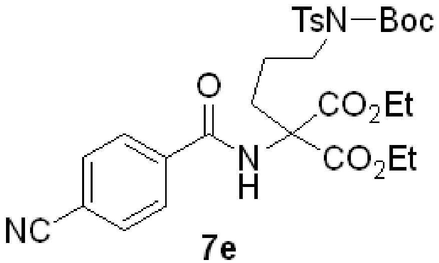

7e

^{1}H NMR (300 MHz, $CDCl_3$)

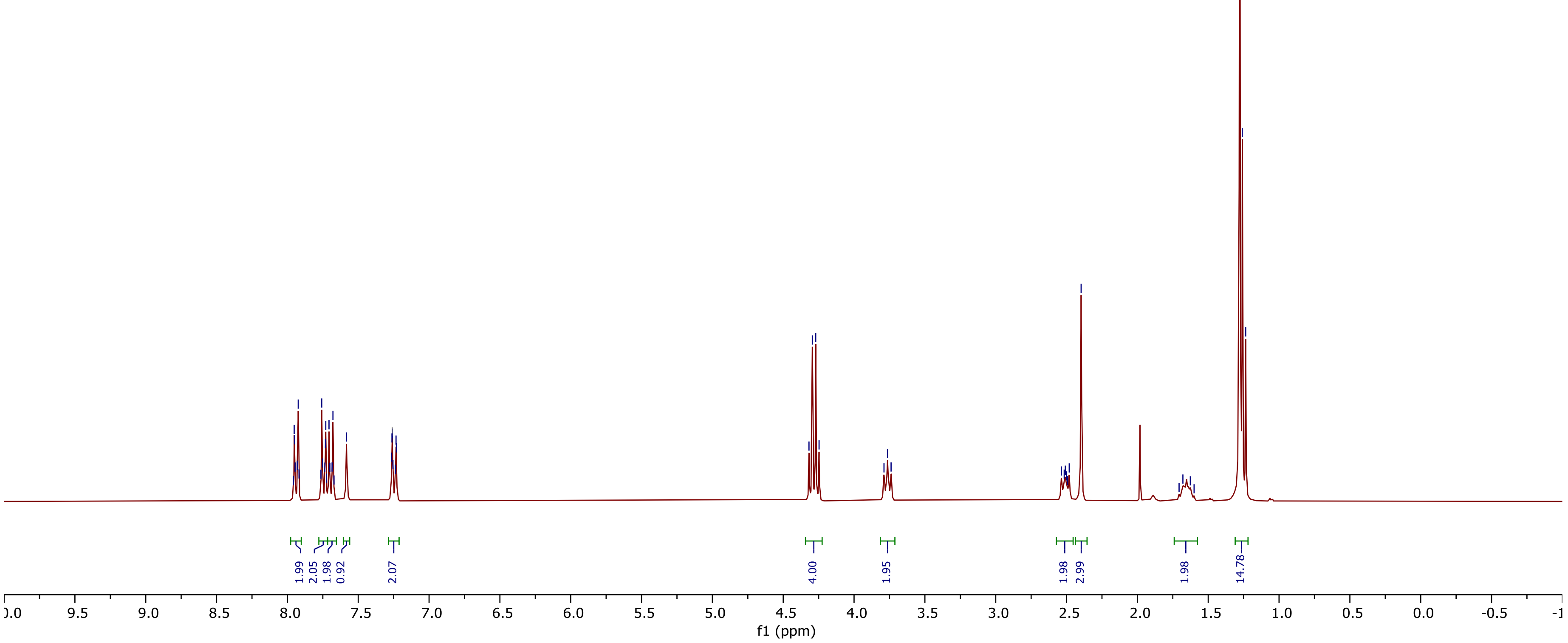

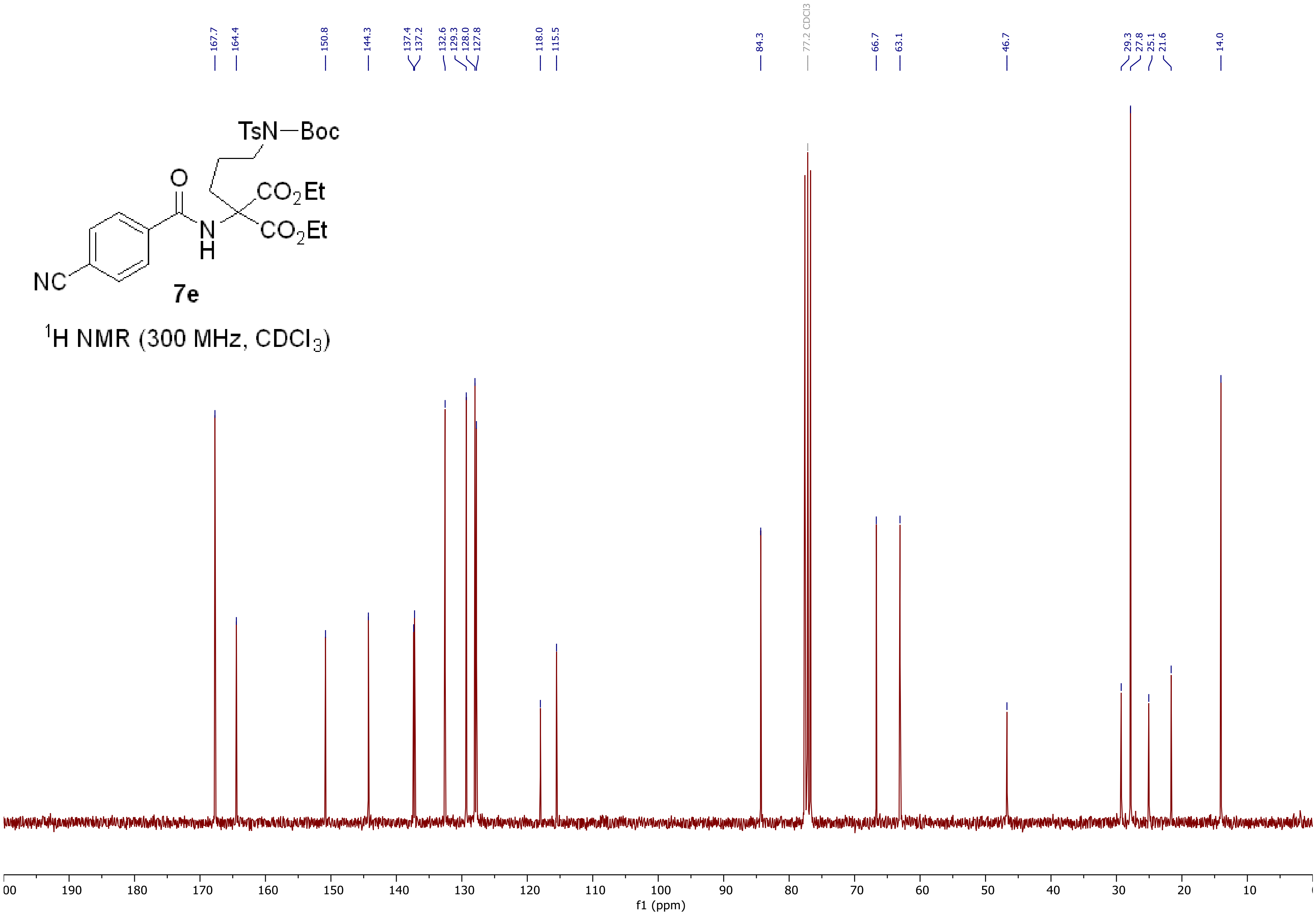
TsN—Boc
CO2Et
CO2Et
NC
N
H
O
7e
1H NMR (300 MHz, CDCl3)
167.7
164.4
150.8
144.3
137.4
137.2
132.6
129.3
128.0
127.8
118.0
115.5
84.3
77.2 CDCl3
66.7
63.1
46.7
29.3
27.8
25.1
21.6
14.0
f1 (ppm)

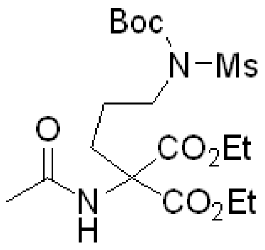

7f

^{1}H NMR (300 MHz, $CDCl_3$)

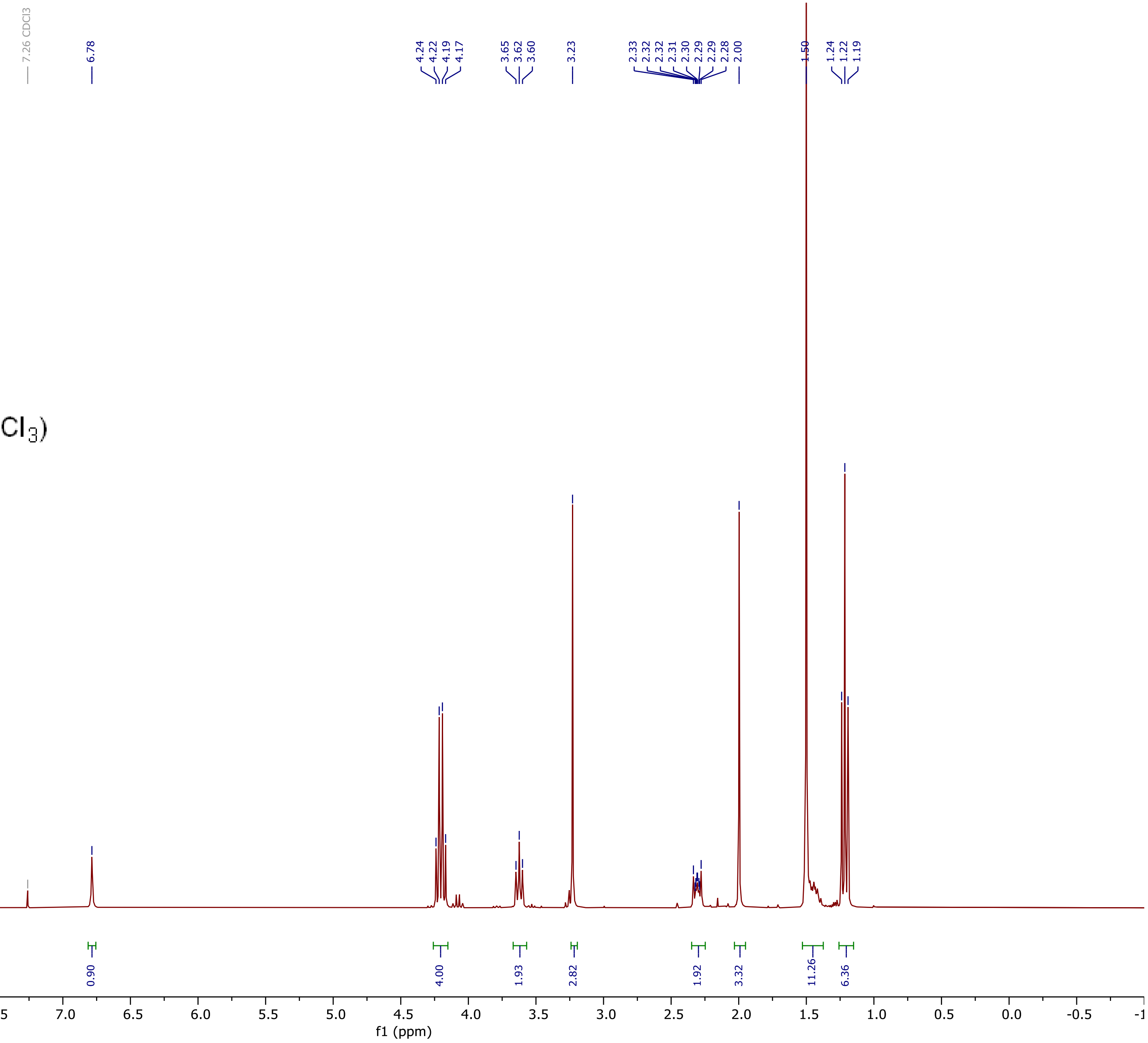

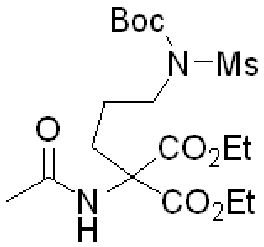

7f

^{13}C NMR (75 MHz, $CDCl_3$)

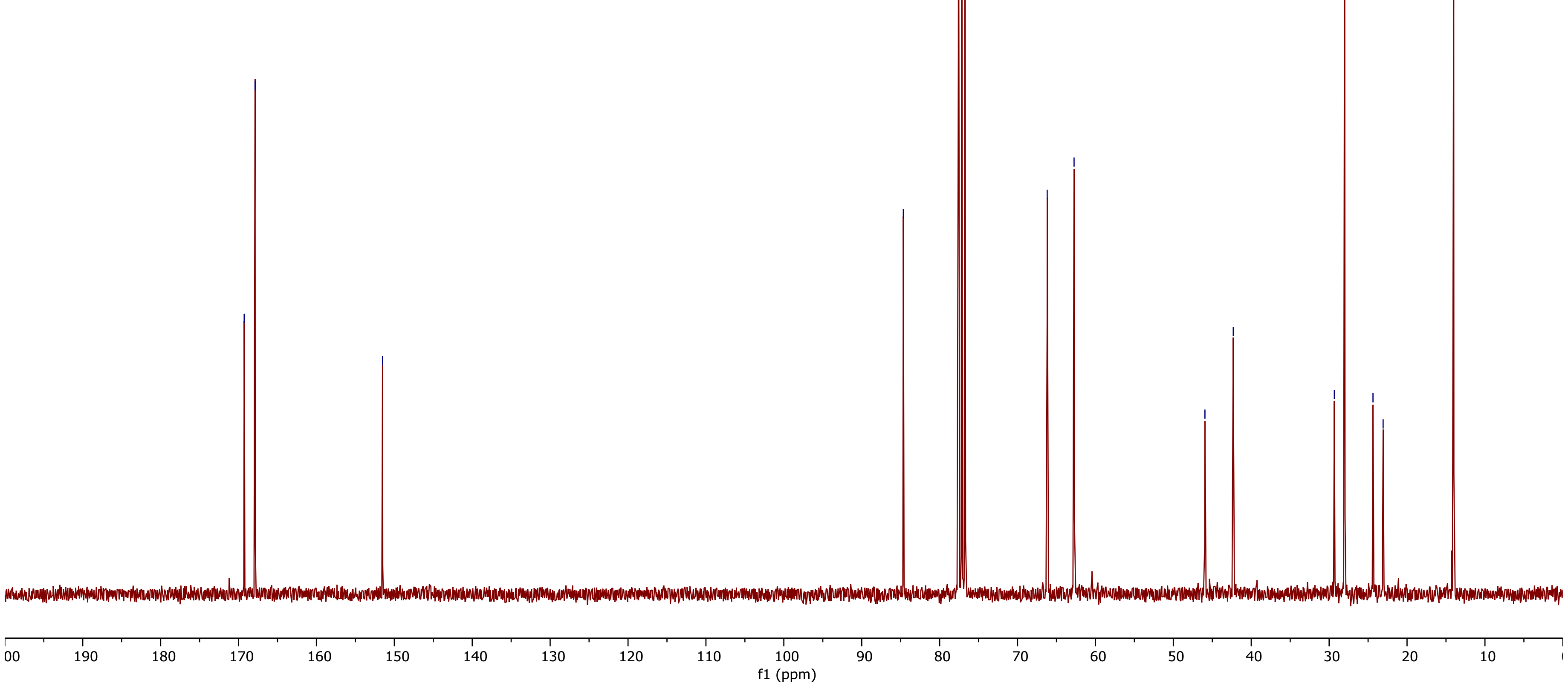

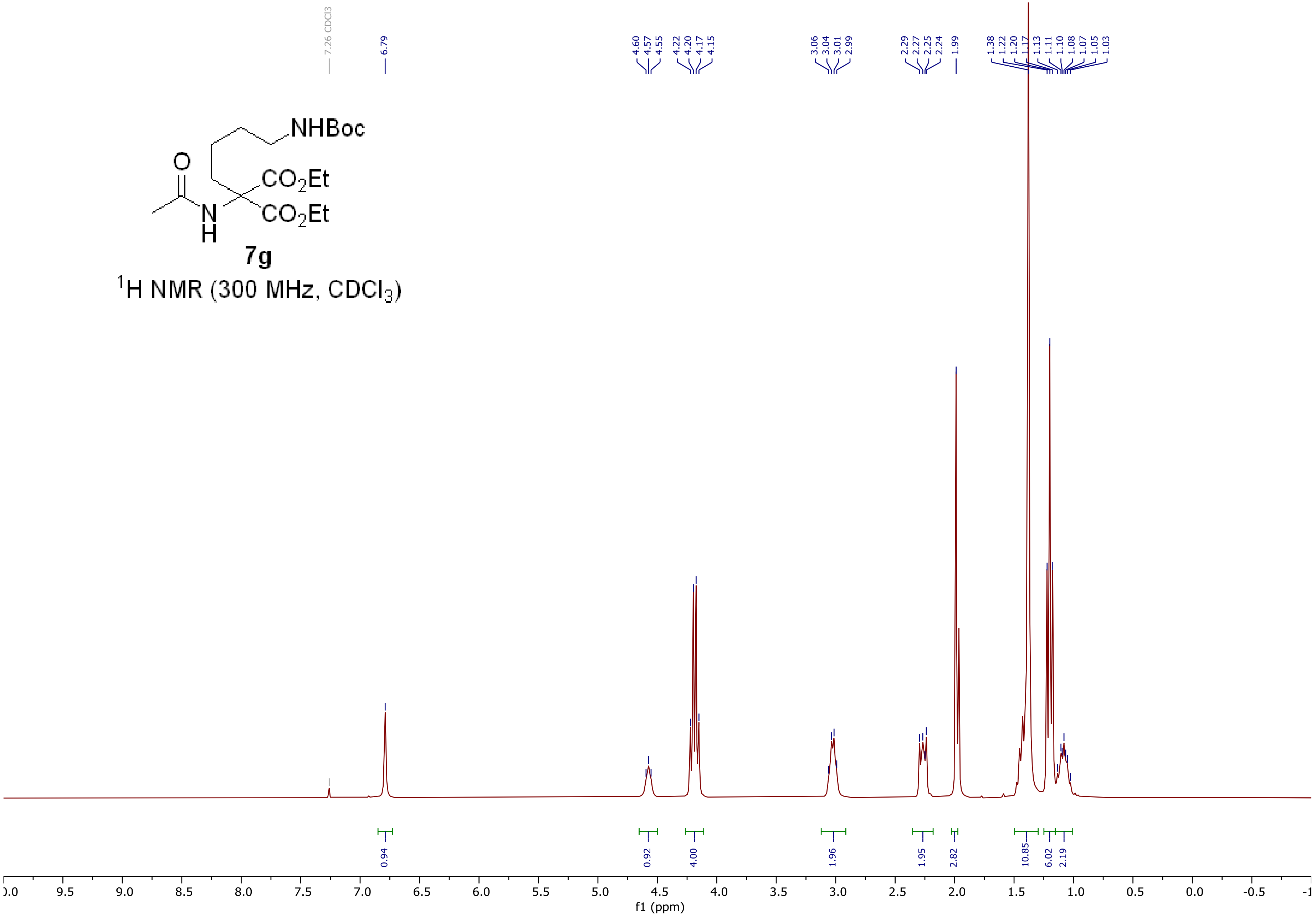
NHBoc
CO2Et
CO2Et
7g
1H NMR (300 MHz, CDCl3)
7.26 CDCl3
6.79
4.60 4.57 4.55 4.22 4.20 4.17 4.15
3.06 3.04 3.01 2.99
2.29 2.27 2.25 2.24 1.99
1.38 1.22 1.20 1.17 1.13 1.11 1.10 1.08 1.07 1.05 1.03
0.94 0.92 4.00 1.96 1.95 2.82 10.85 6.02 2.19
f1 (ppm)

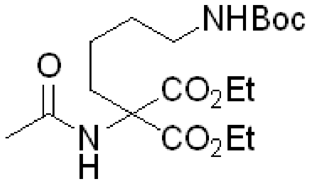

7g

^{13}C NMR (75 MHz, $CDCl_3$)

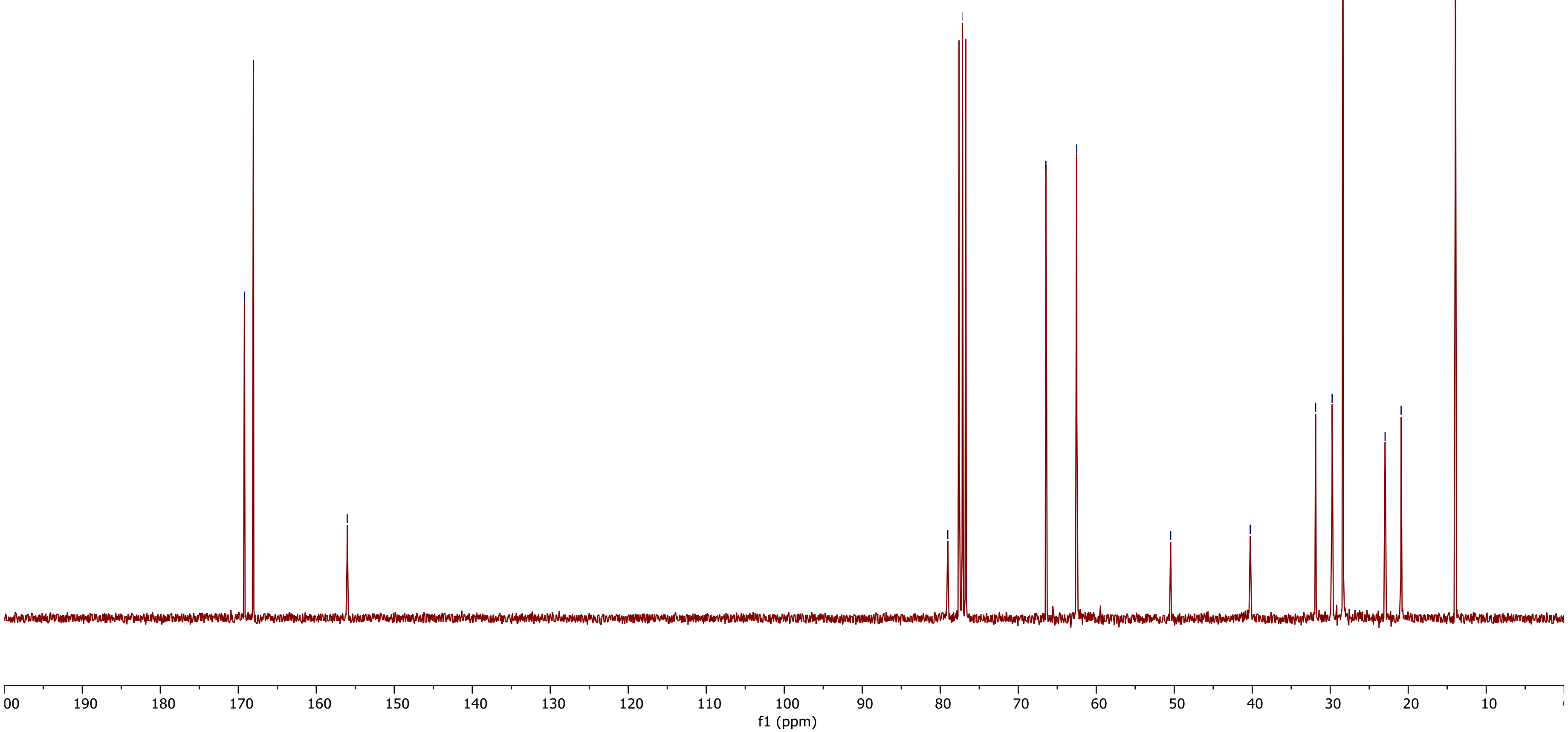

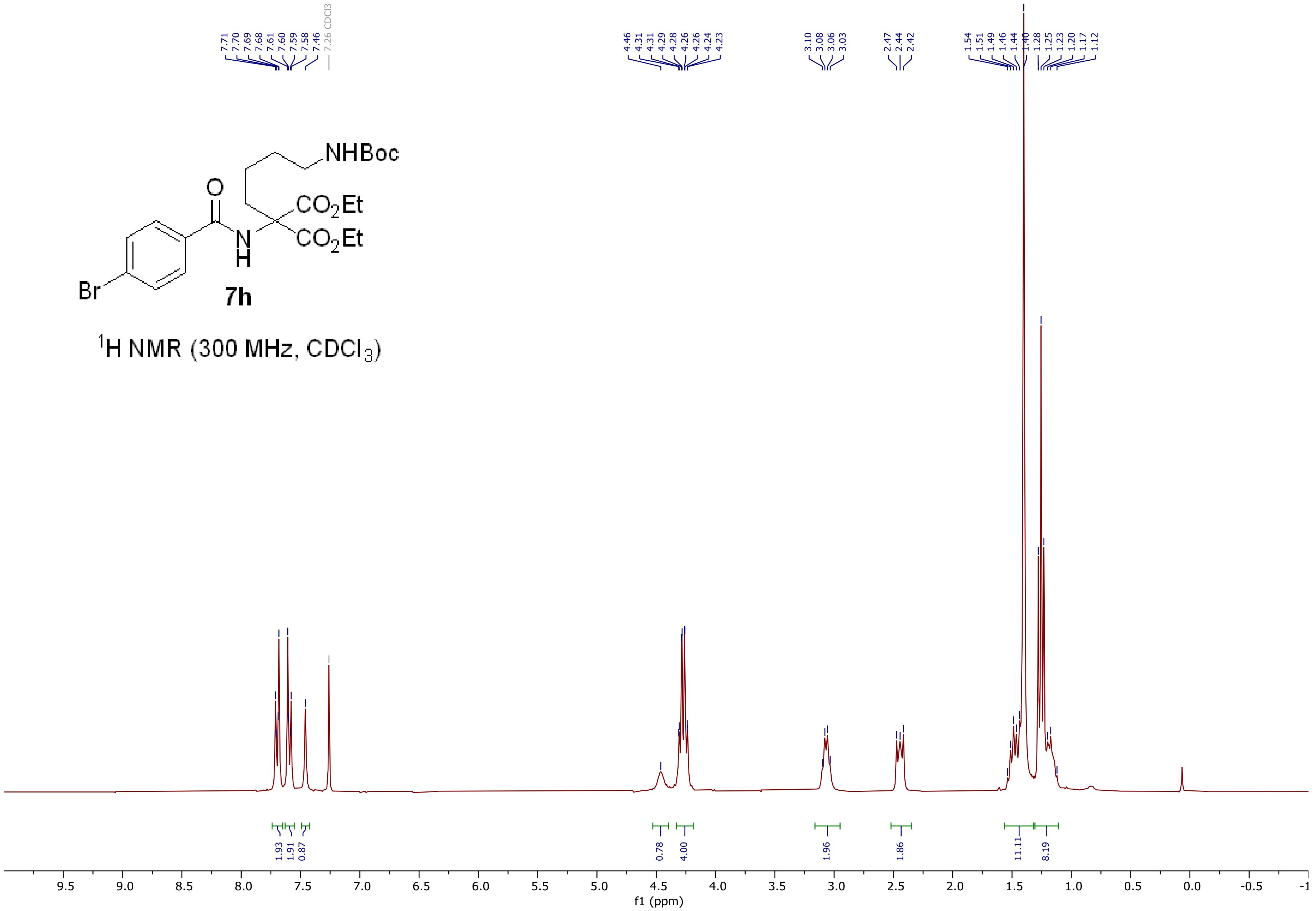
7.71
7.70
7.69
7.68
7.61
7.60
7.59
7.58
7.46
7.26 CDCl3
4.46
4.31
4.31
4.29
4.28
4.26
4.26
4.24
4.23
3.10
3.08
3.06
3.03
2.47
2.44
2.42
1.54
1.51
1.49
1.46
1.44
1.40
1.28
1.25
1.23
1.20
1.17
1.12
NHBoc
O
CO2Et
CO2Et
N
H
Br
7h
1H NMR (300 MHz, CDCl3)
1.93
1.91
0.87
0.78
4.00
1.96
1.86
11.11
8.19
9.5 9.0 8.5 8.0 7.5 7.0 6.5 6.0 5.5 5.0 4.5 4.0 3.5 3.0 2.5 2.0 1.5 1.0 0.5 0.0 -0.5 -1
f1 (ppm)

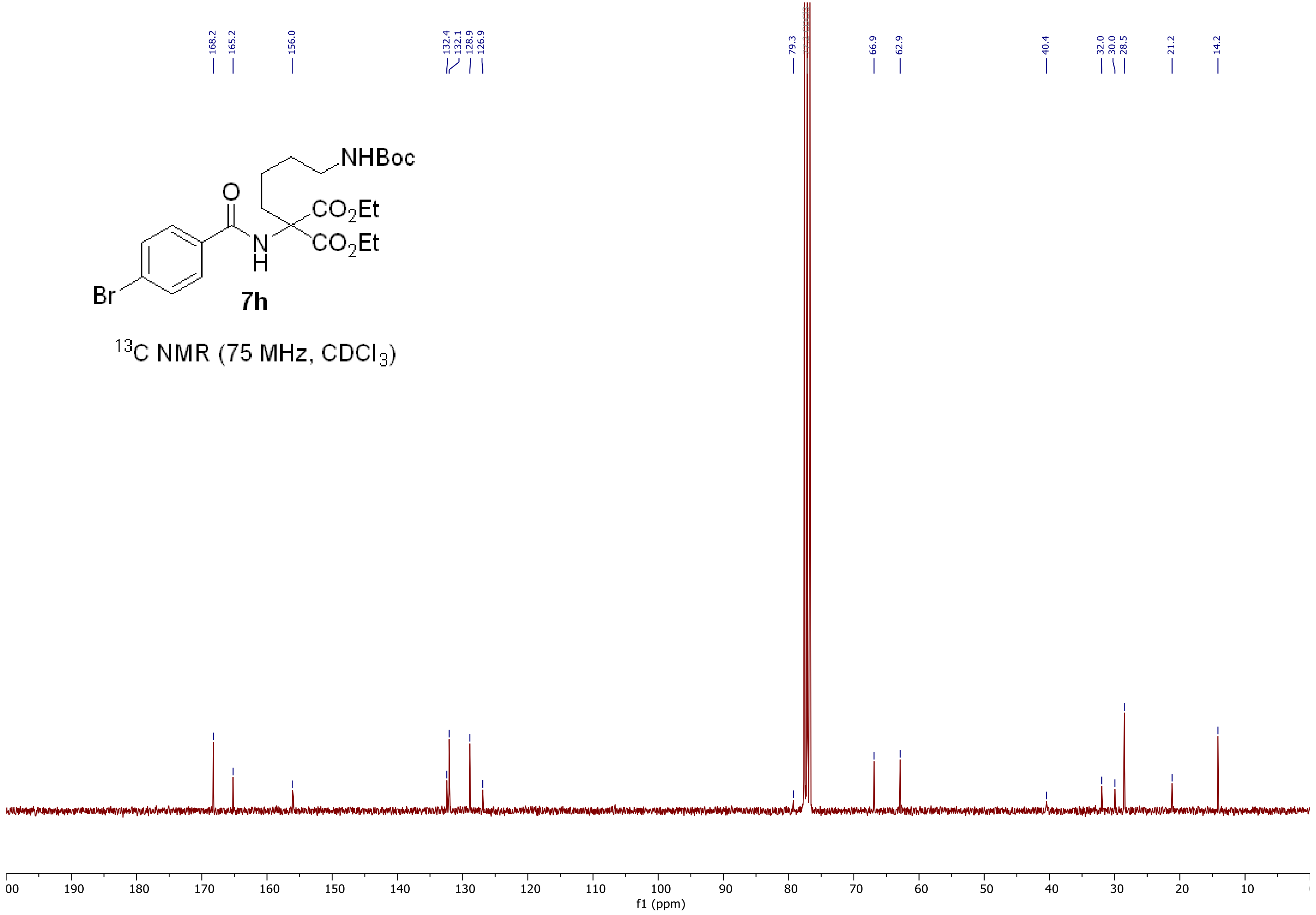

NHBoc
O
CO2Et
CO2Et
N
H
Br
7h
13C NMR (75 MHz, CDCl3)
168.2
165.2
156.0
132.4
132.1
128.9
126.9
79.3
66.9
62.9
40.4
32.0
30.0
28.5
21.2
14.2
f1 (ppm)

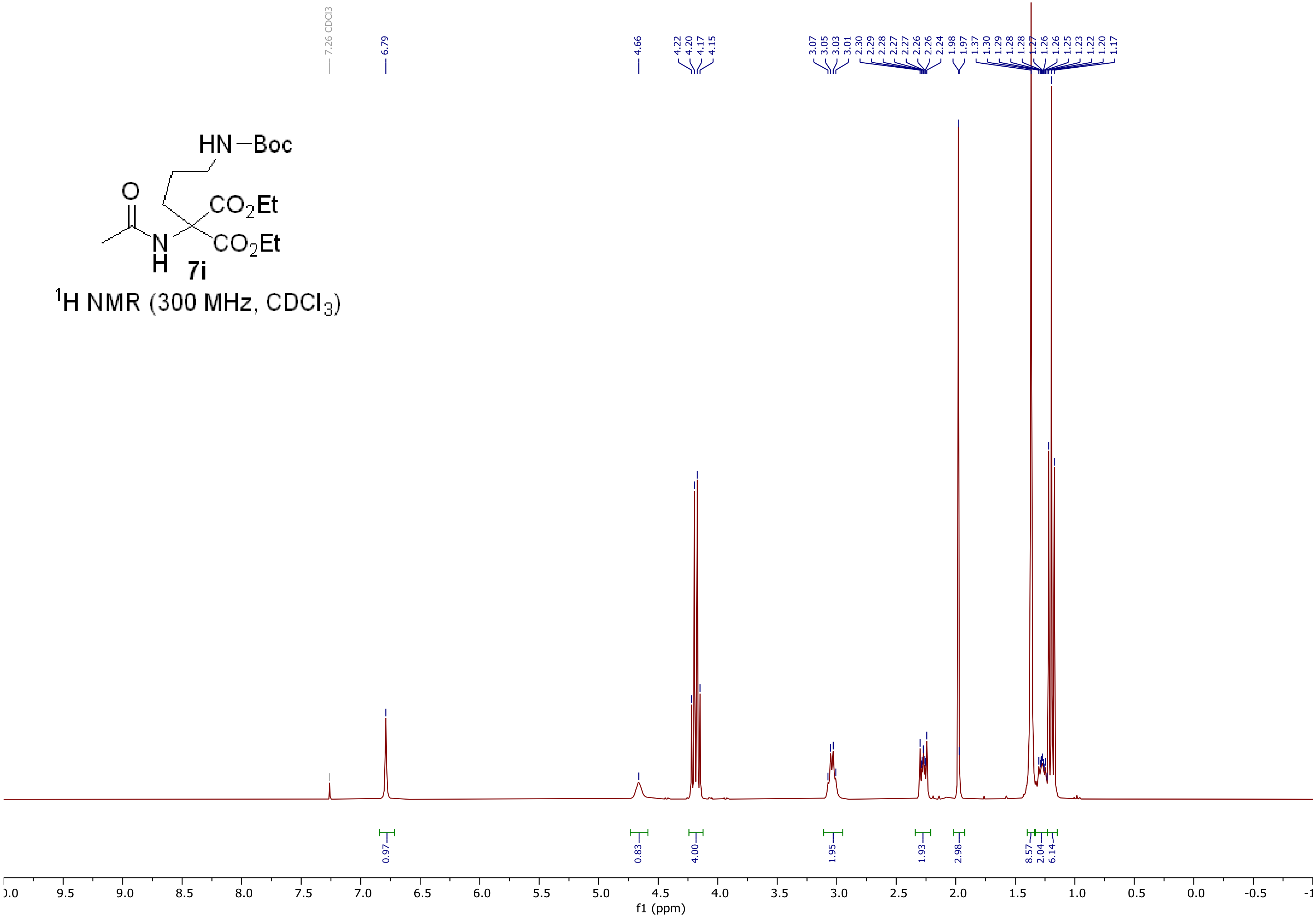

7i

^{1}H NMR (300 MHz, $CDCl_3$)

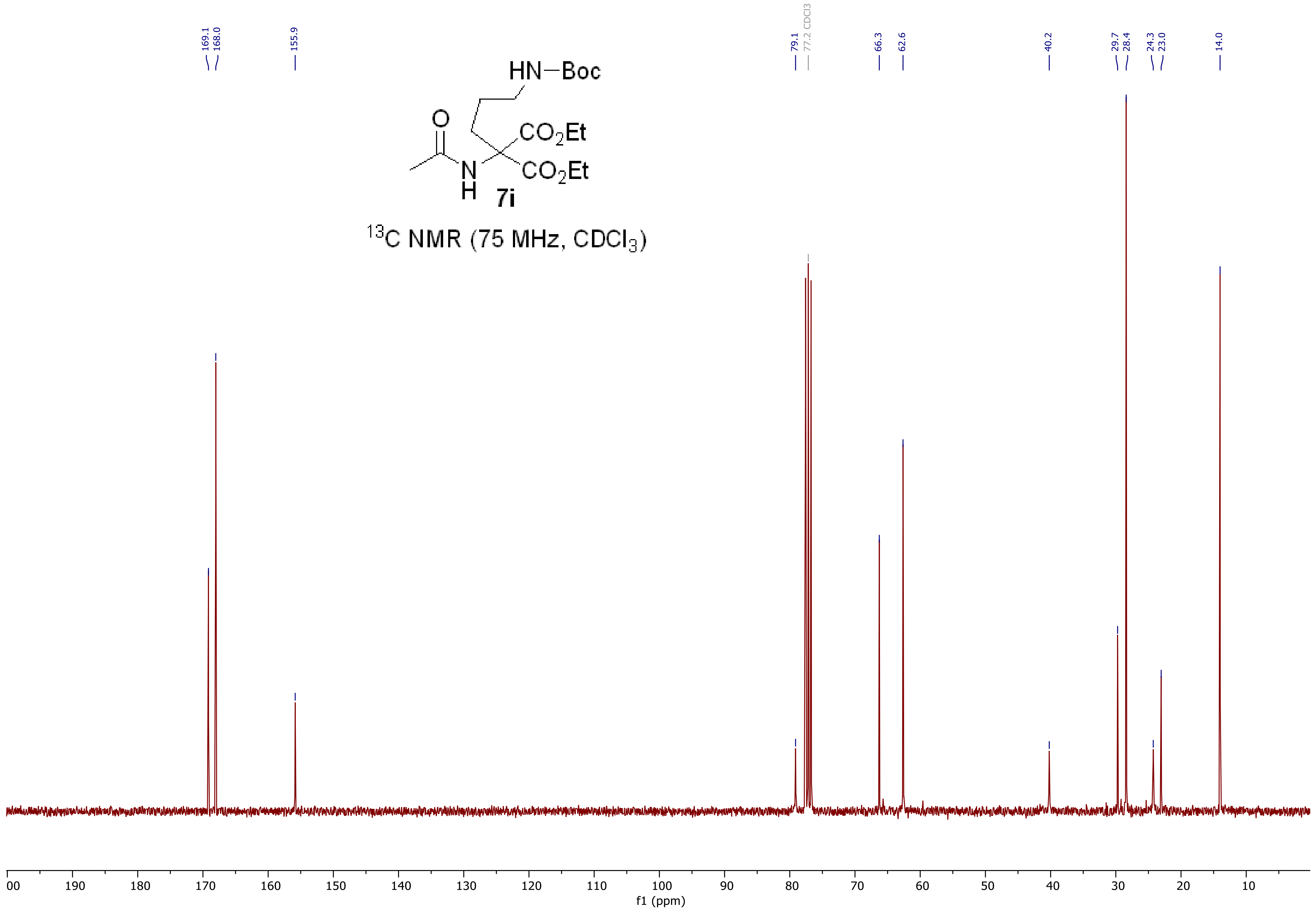
HN—Boc
O
N
H
CO2Et
CO2Et
7i
13C NMR (75 MHz, CDCl3)
169.1
168.0
155.9
79.1
77.2 CDCl3
66.3
62.6
40.2
29.7
28.4
24.3
23.0
14.0
00
190
180
170
160
150
140
130
120
110
100
90
80
70
60
50
40
30
20
10
f1 (ppm)

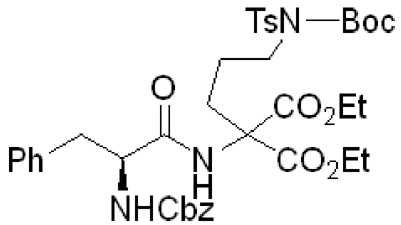

7j

^{1}H NMR (300 MHz, $CDCl_3$)

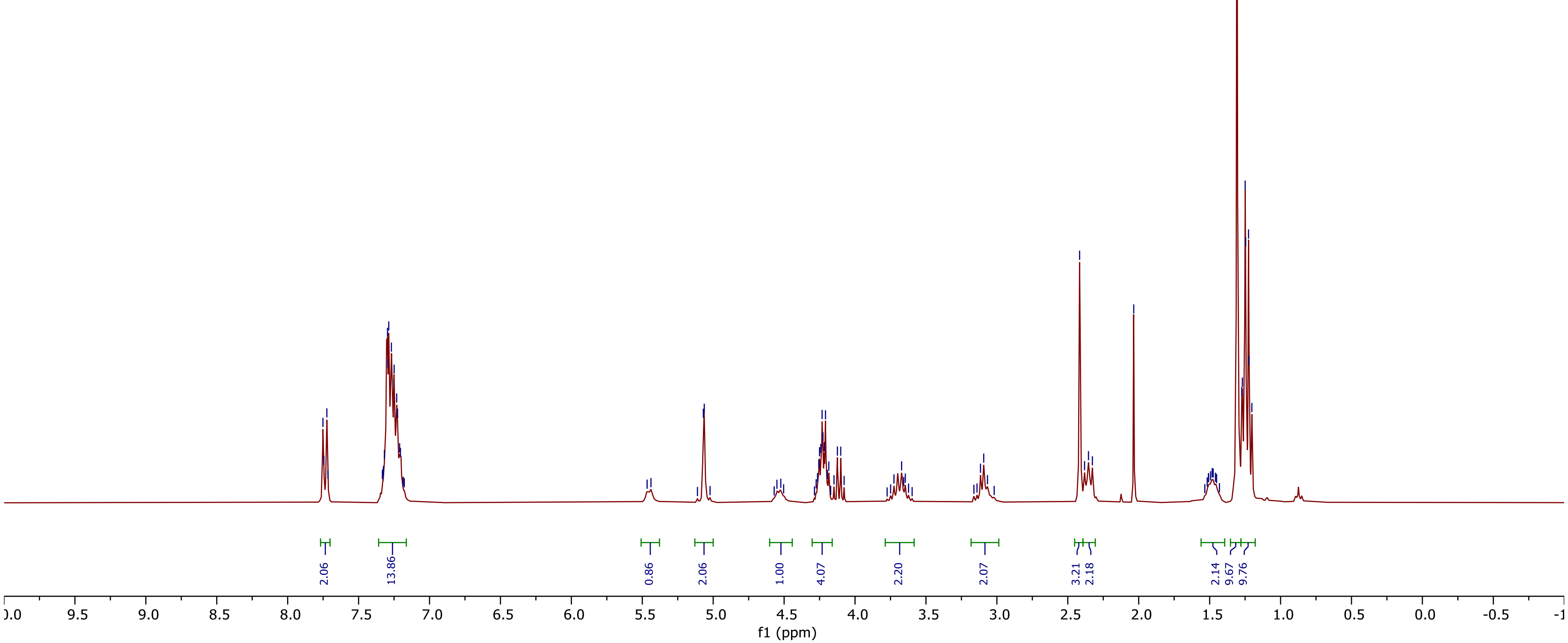

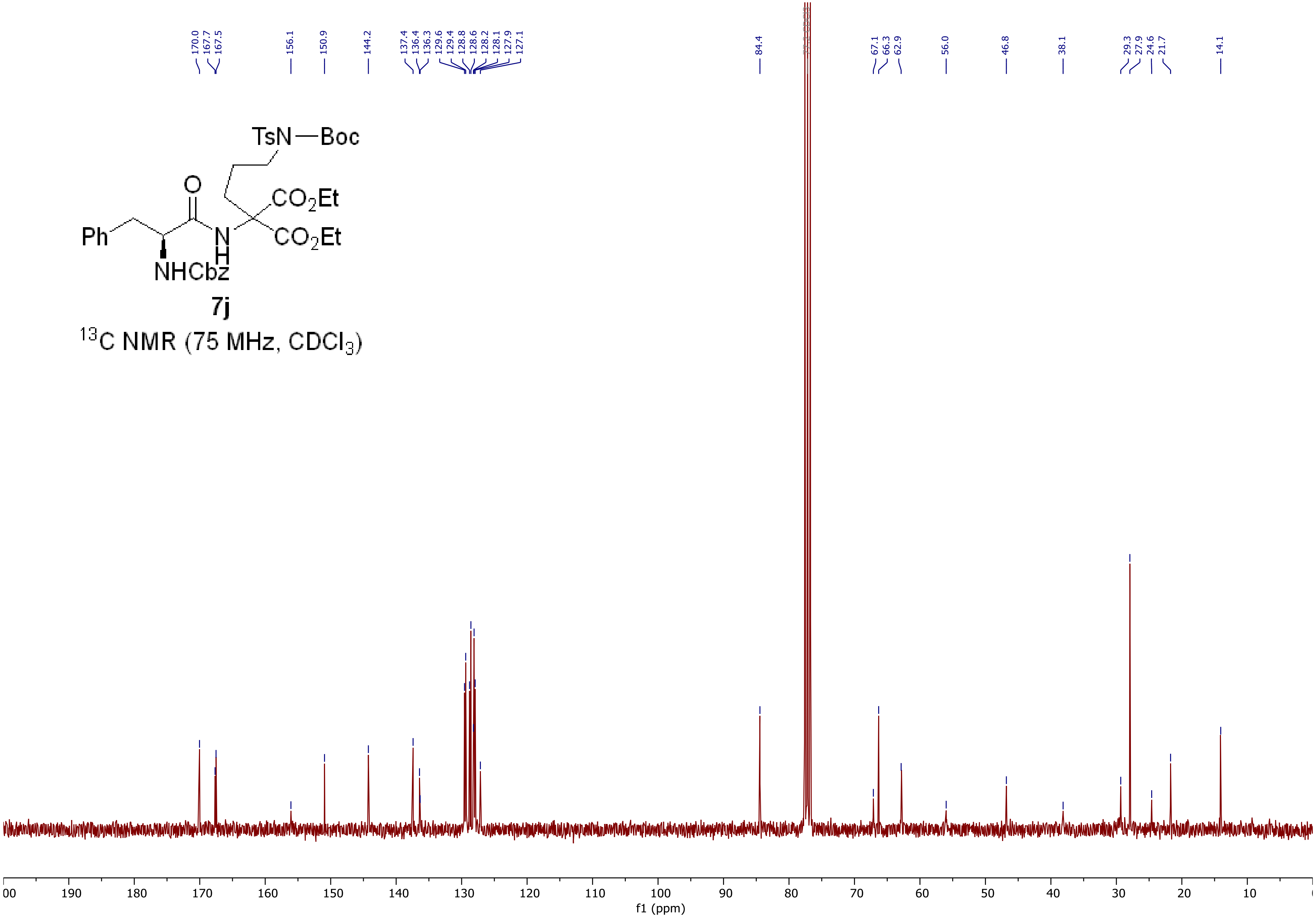
170.0
167.7
167.5
156.1
150.9
144.2
137.4
136.4
136.3
129.6
129.4
128.8
128.6
128.2
128.1
127.9
127.1
84.4
67.1
66.3
62.9
56.0
46.8
38.1
29.3
27.9
24.6
21.7
14.1
TsN—Boc
CO2Et
CO2Et
O
Ph
N
H
NHCbz
7j
13C NMR (75 MHz, CDCl3)
f1 (ppm)

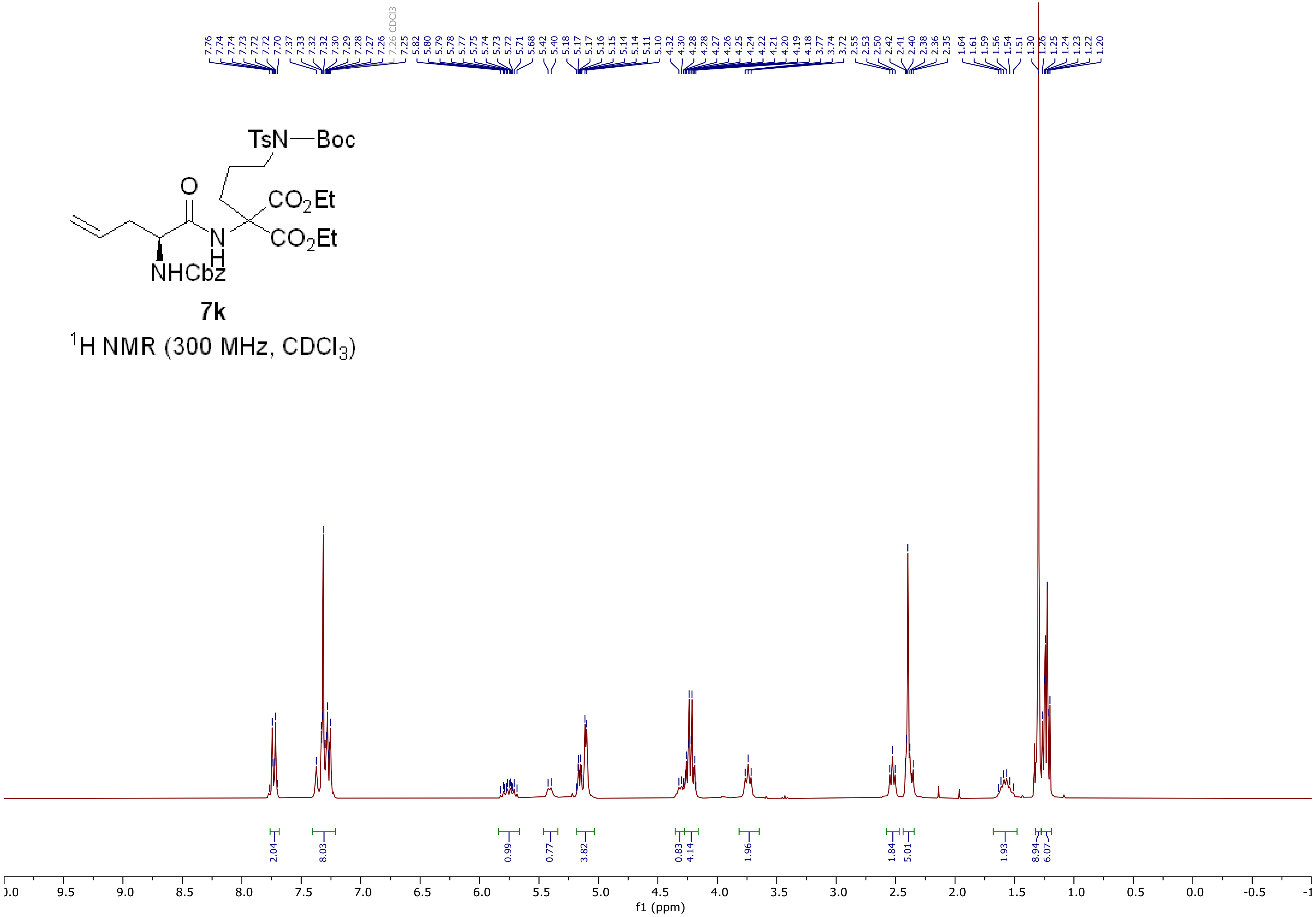

TsN—Boc
CO_2Et
CO_2Et
NHCbz
7k
^{1}H NMR (300 MHz, $CDCl_3$)
f1 (ppm)

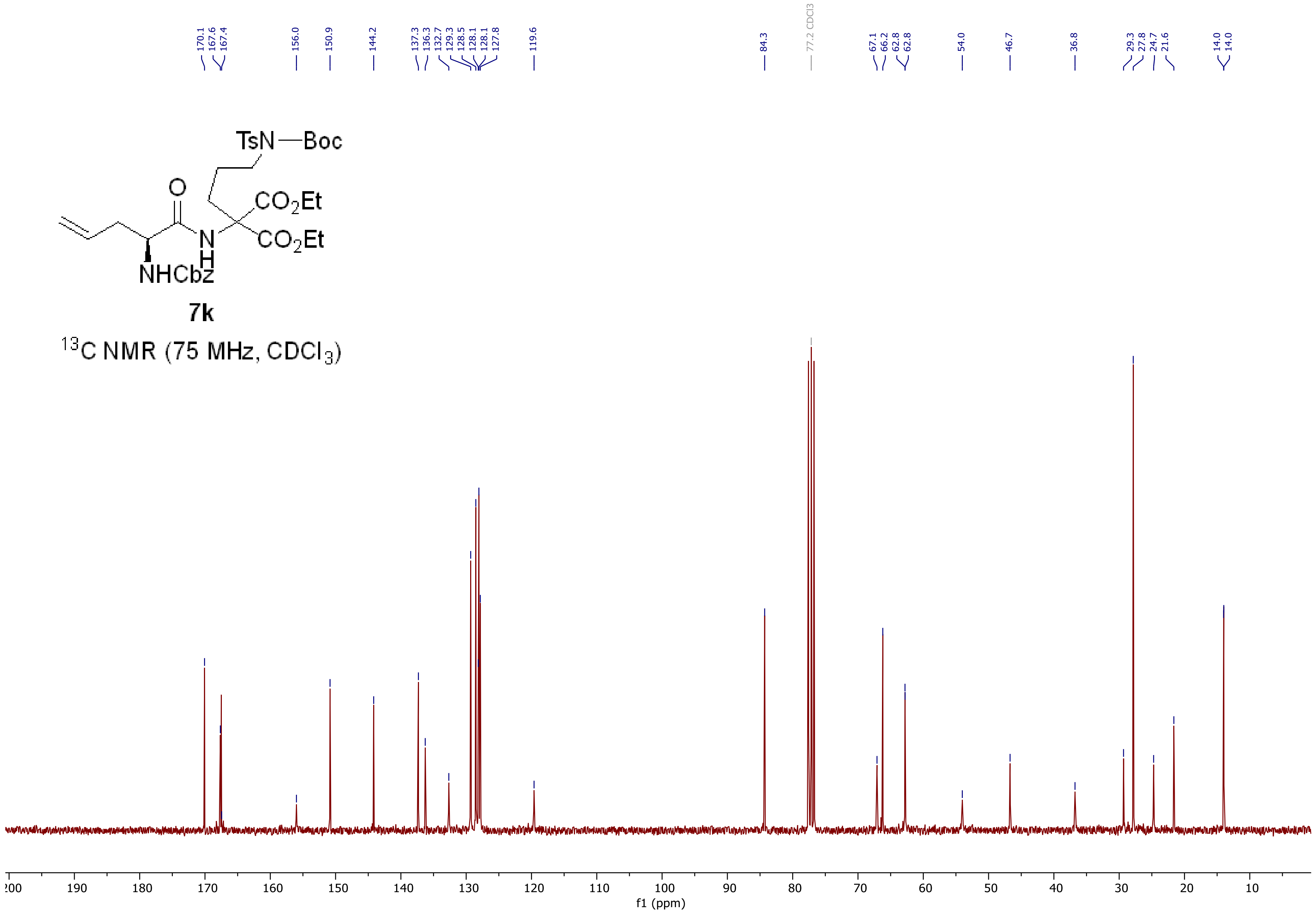
170.1
167.6
167.4
156.0
150.9
144.2
137.3
136.3
132.7
129.3
128.5
128.1
128.1
127.8
119.6
84.3
77.2 CDCl3
67.1
66.2
62.8
62.8
54.0
46.7
36.8
29.3
27.8
24.7
21.6
14.0
14.0
TsN—Boc
O
CO2Et
CO2Et
N
H
NHCbz
7k
13C NMR (75 MHz, CDCl3)
200
190
180
170
160
150
140
130
120
110
100
90
80
70
60
50
40
30
20
10
f1 (ppm)

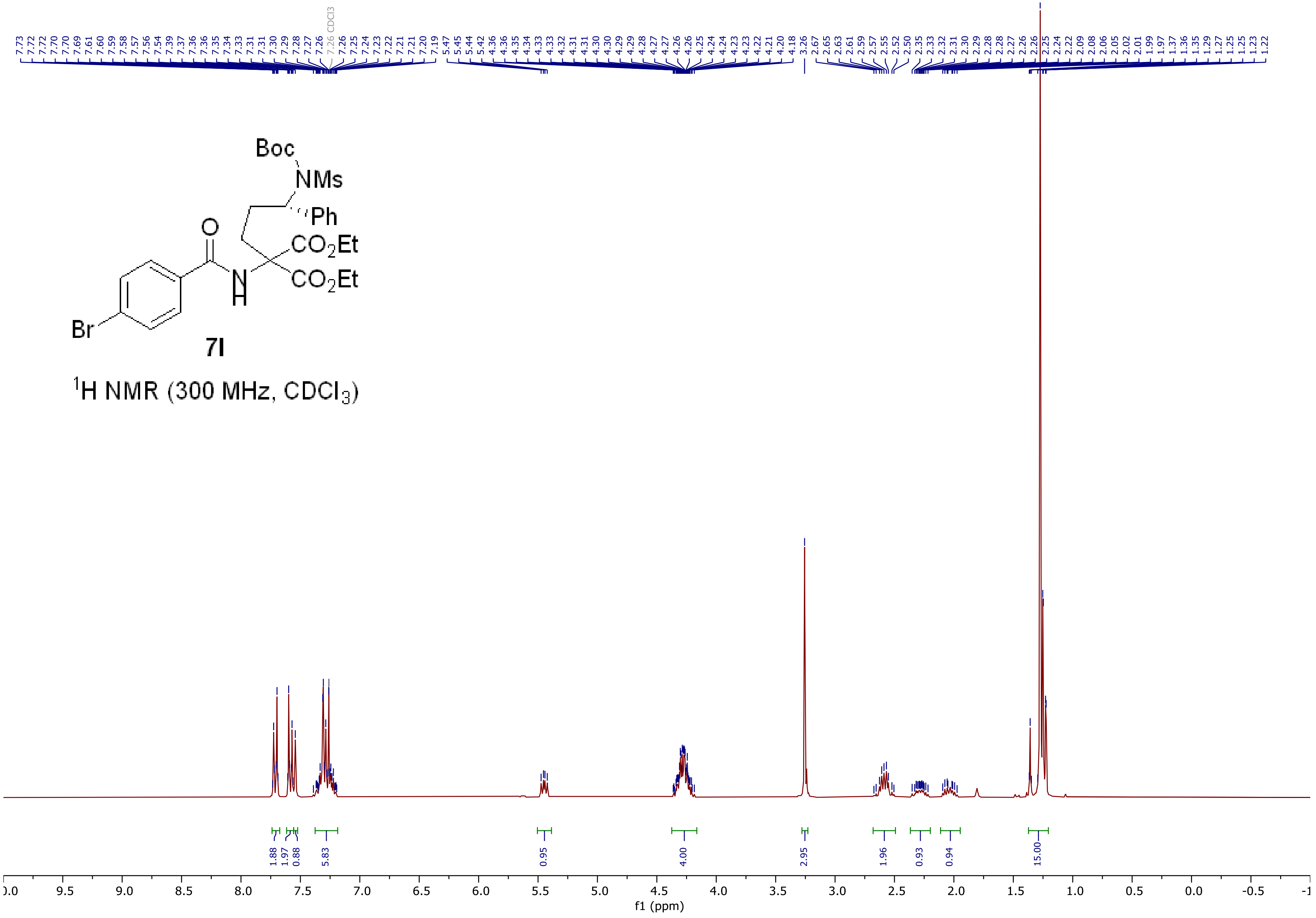

Boc
NMs
Ph
CO_2Et
CO_2Et
O
N
H
Br
7l
^1H NMR (300 MHz, $CDCl_3$)
f1 (ppm)

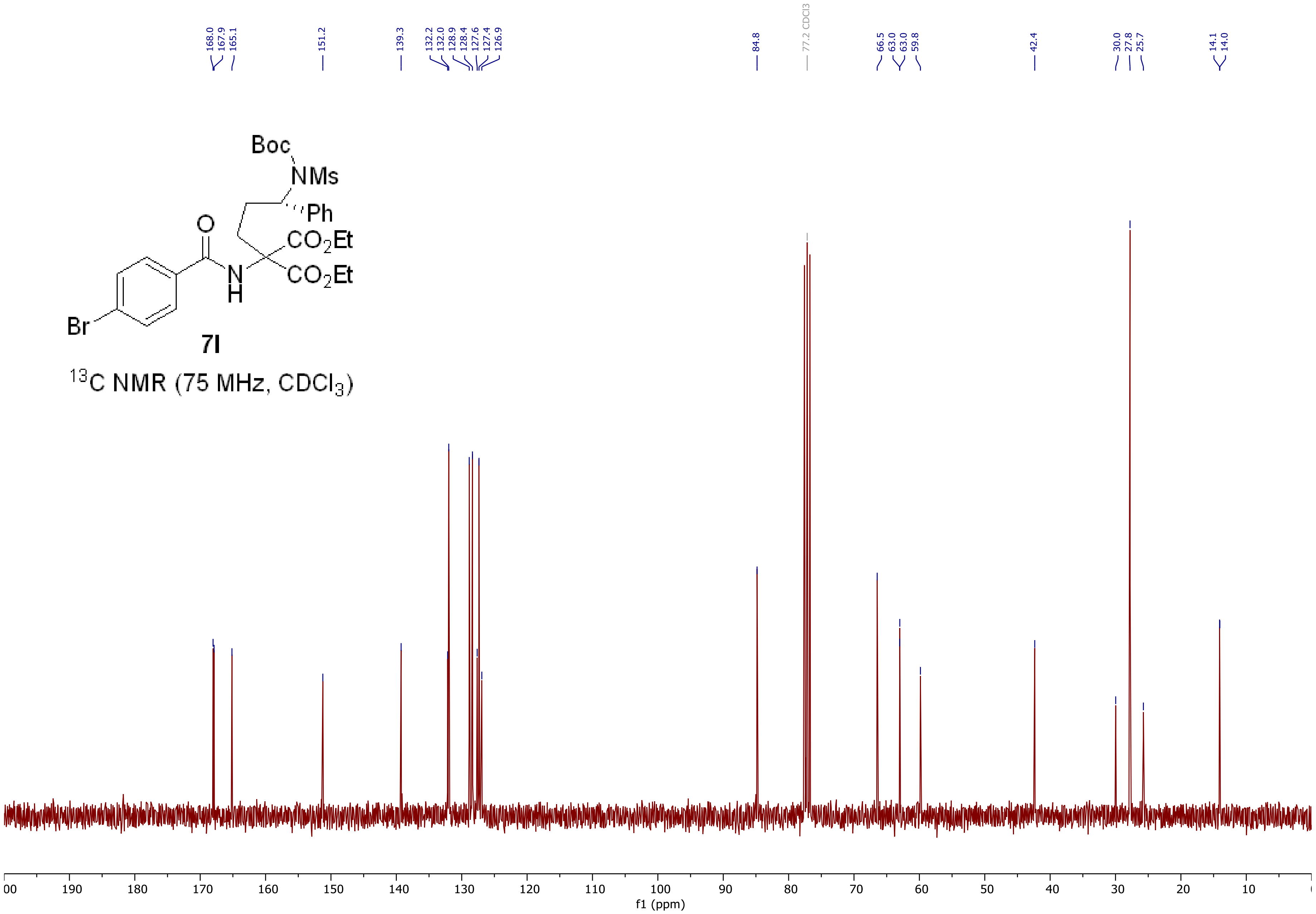

7l

^{13}C NMR (75 MHz, $CDCl_3$)

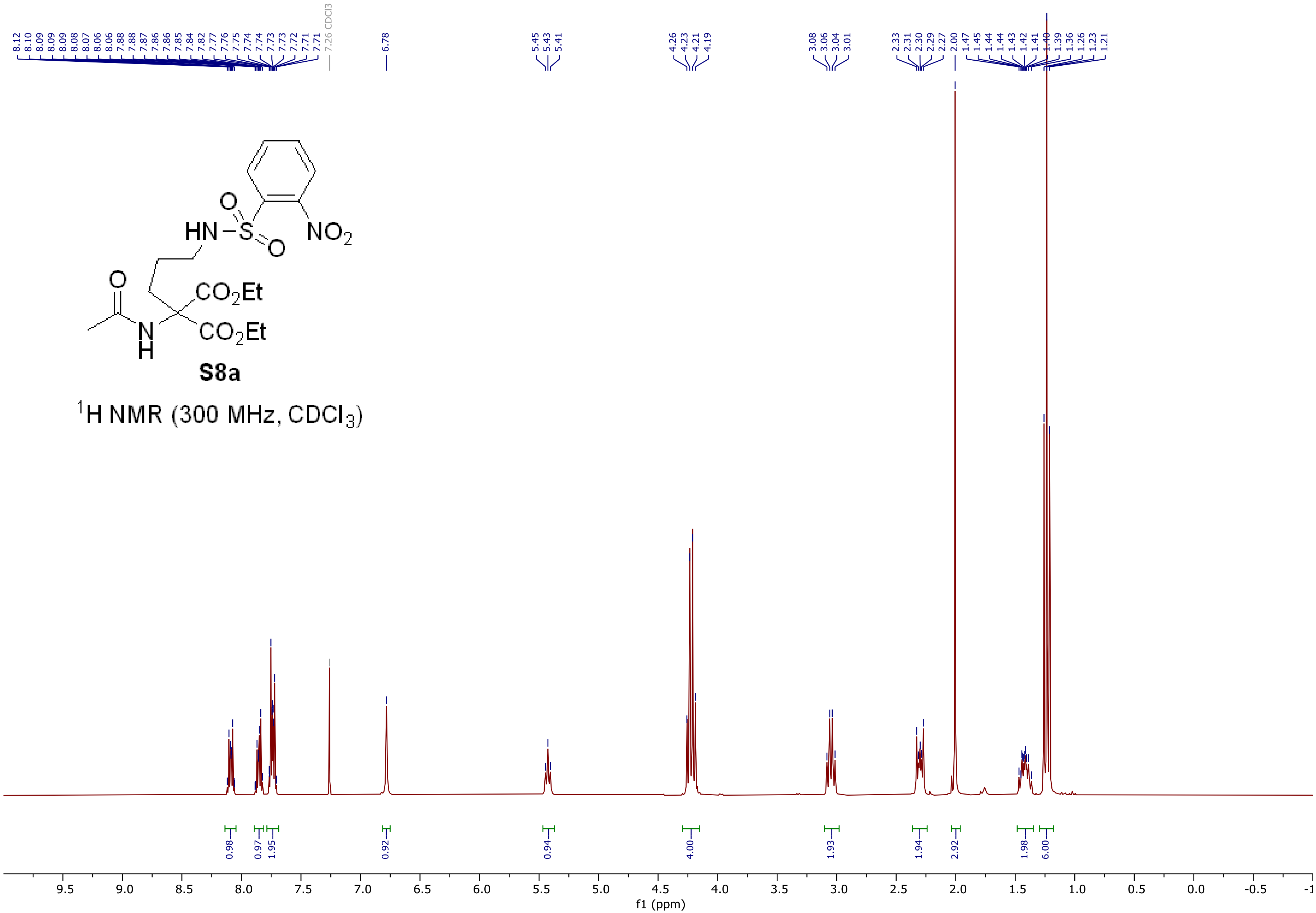
S8a
^{1}H NMR (300 MHz, $CDCl_3$)
f1 (ppm)

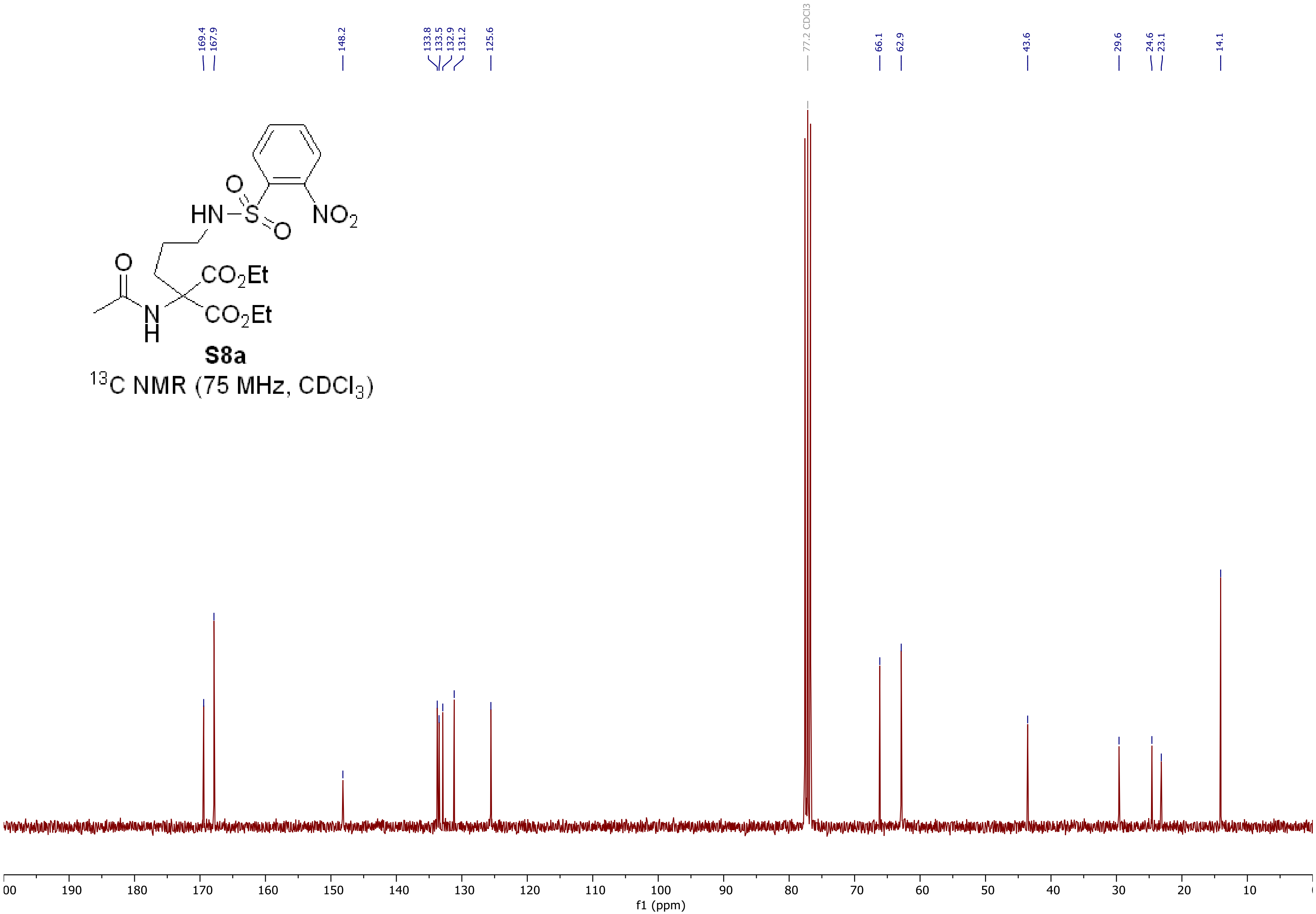

S8a

^{13}C NMR (75 MHz, $CDCl_3$)

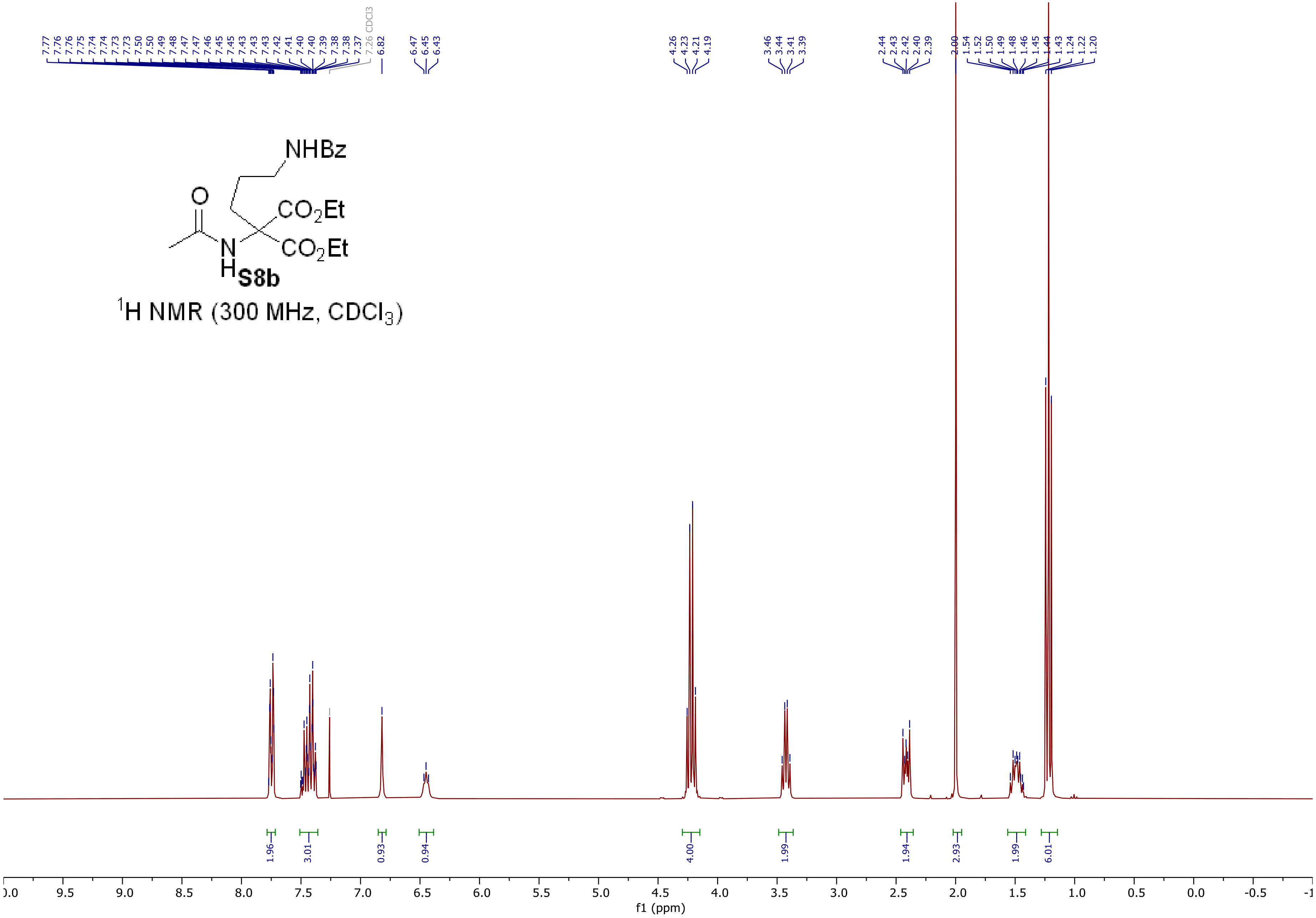
NHBz
O
CO2Et
N
H
CO2Et
S8b
1H NMR (300 MHz, CDCl3)
7.77 7.76 7.76 7.75 7.74 7.74 7.73 7.73 7.50 7.49 7.48 7.47 7.47 7.46 7.45 7.45 7.43 7.43 7.43 7.42 7.41 7.40 7.40 7.39 7.38 7.38 7.37 7.26 CDCl3 6.82 6.47 6.45 6.43
4.26 4.23 4.21 4.19 3.46 3.44 3.41 3.39 2.44 2.43 2.42 2.40 2.39 2.00 1.54 1.52 1.50 1.49 1.48 1.46 1.45 1.44 1.43 1.24 1.22 1.20
1.96 3.01 0.93 0.94 4.00 1.99 1.94 2.93 1.99 6.01
f1 (ppm)

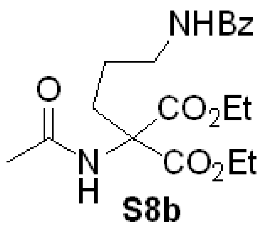

S8b

^{13}C NMR (75 MHz, $CDCl_3$)

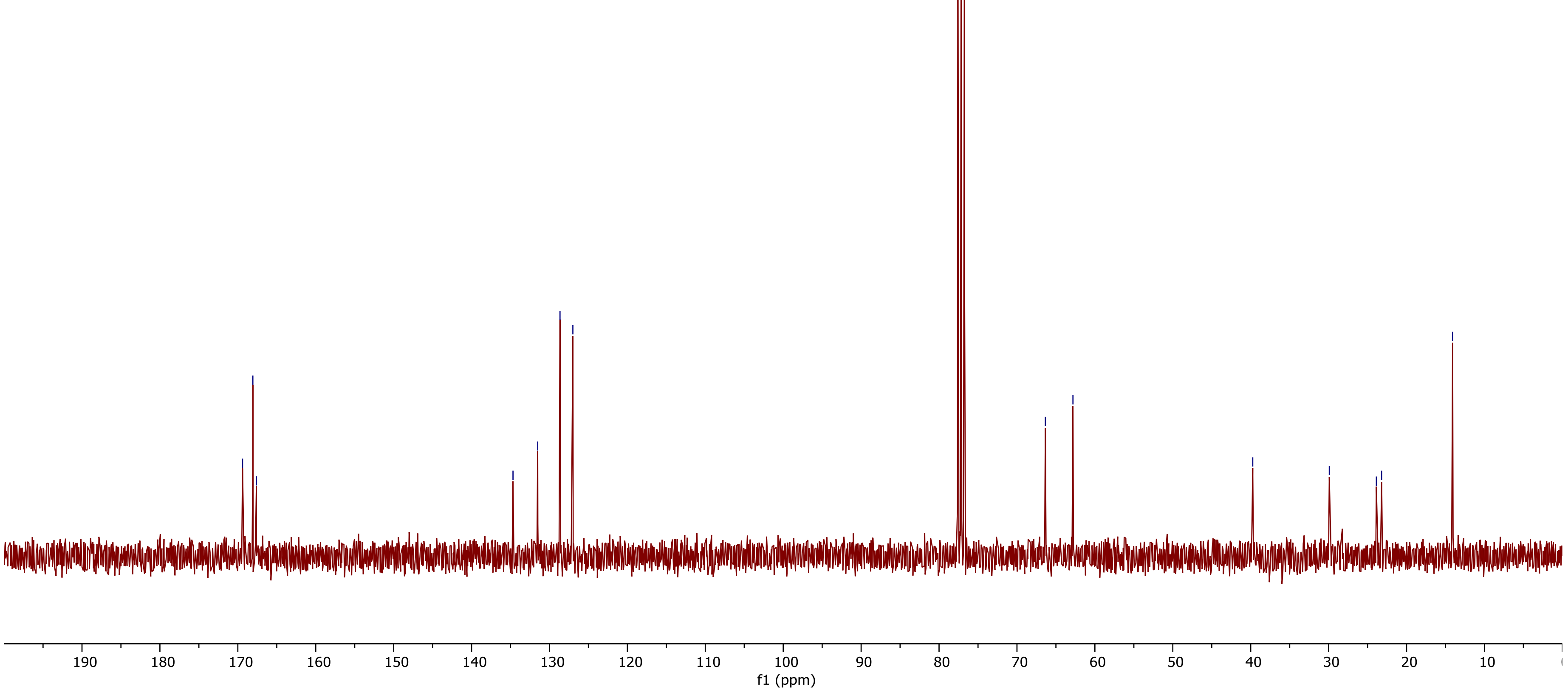

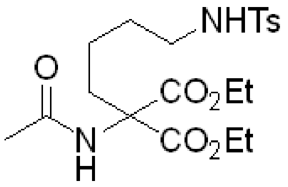

S8c

^{1}H NMR (300 MHz, $CDCl_3$)

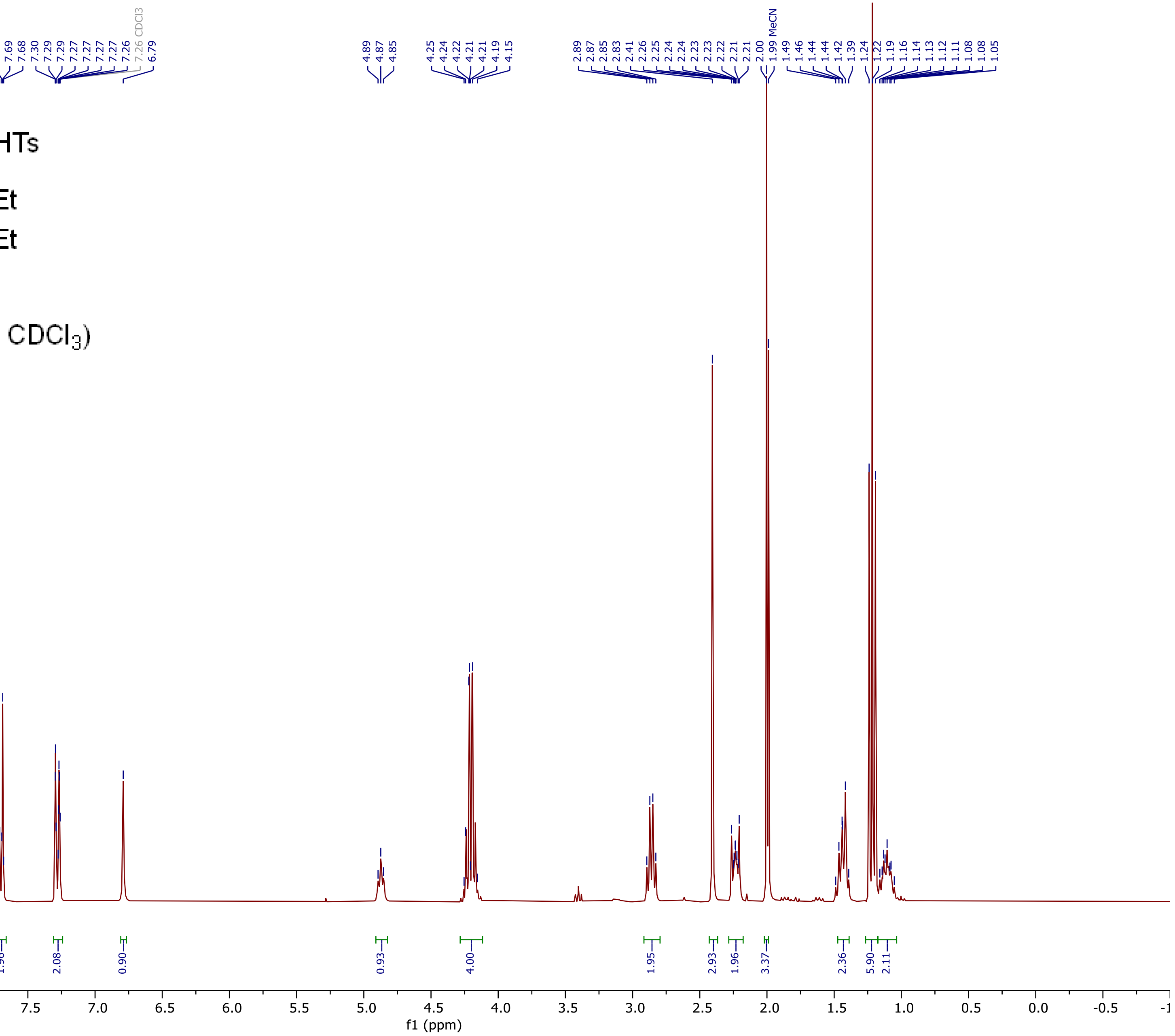

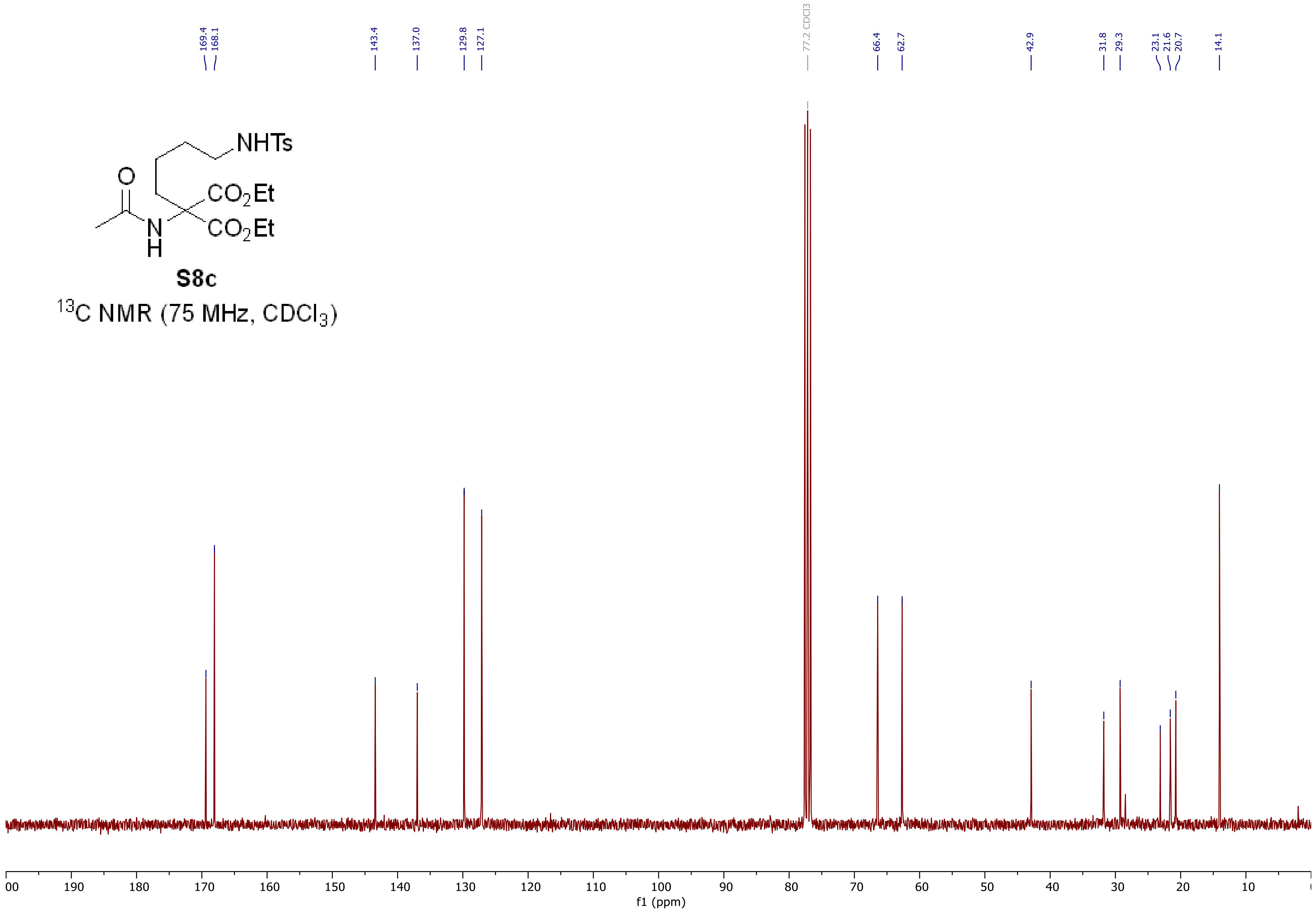

S8c

^{13}C NMR (75 MHz, $CDCl_3$)

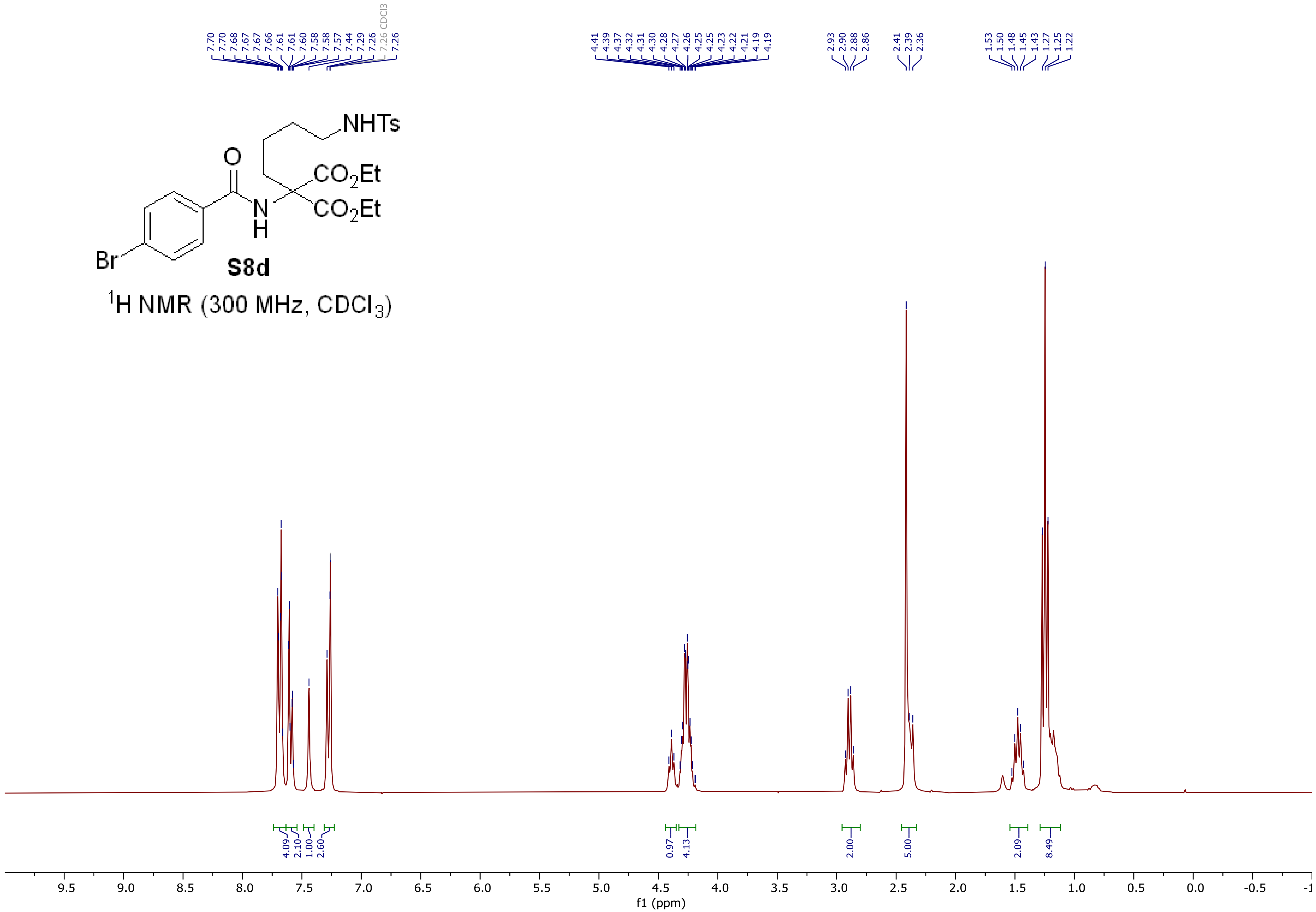
7.70
7.70
7.68
7.67
7.67
7.66
7.61
7.61
7.60
7.58
7.58
7.57
7.44
7.29
7.26
7.26 CDCl3
7.26
4.41
4.39
4.37
4.32
4.31
4.30
4.28
4.27
4.26
4.25
4.25
4.23
4.22
4.21
4.19
4.19
2.93
2.90
2.88
2.86
2.41
2.39
2.36
1.53
1.50
1.48
1.45
1.43
1.27
1.25
1.22
NHTs
O
CO2Et
CO2Et
N
H
Br
S8d
1H NMR (300 MHz, CDCl3)
4.09
2.10
1.00
2.60
0.97
4.13
2.00
5.00
2.09
8.49
f1 (ppm)

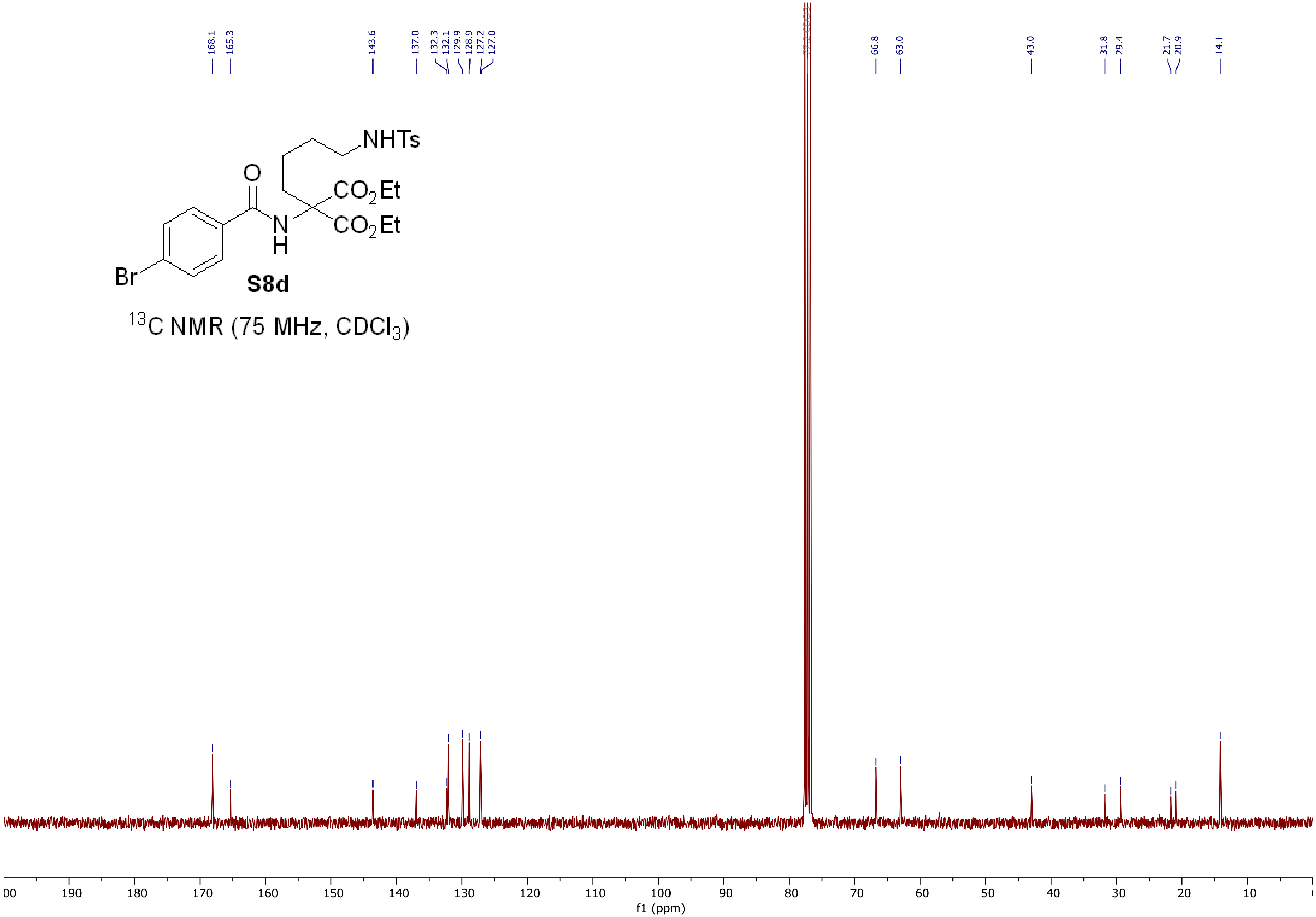
168.1
165.3
143.6
137.0
132.3
132.1
129.9
128.9
127.2
127.0
66.8
63.0
43.0
31.8
29.4
21.7
20.9
14.1
NHTs
O
CO_2Et
CO_2Et
N
H
Br
S8d
^{13}C NMR (75 MHz, $CDCl_3$)
f1 (ppm)

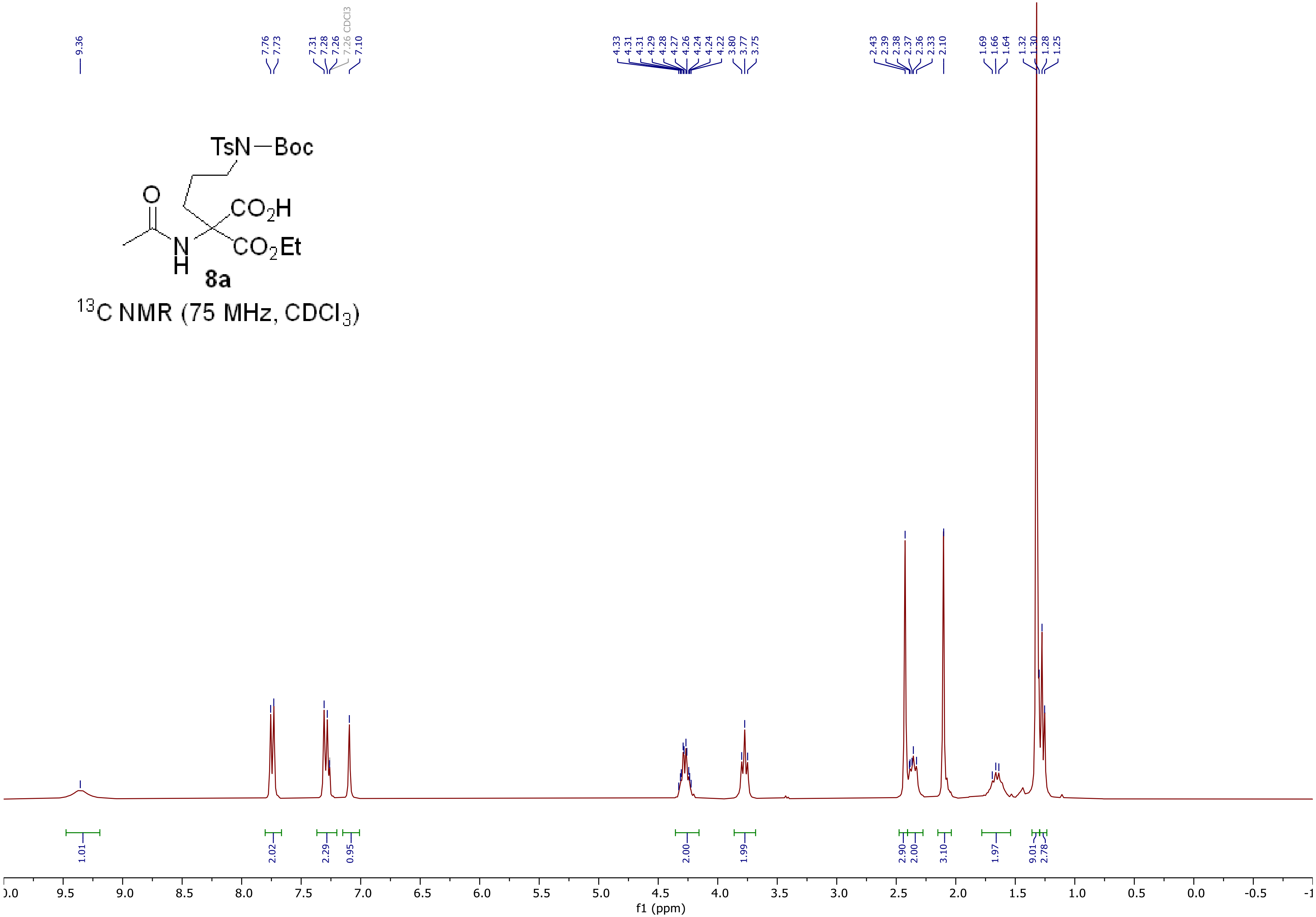

8a
13C NMR (75 MHz, CDCl3)
9.36
7.76
7.73
7.31
7.28
7.26
7.26 CDCl3
7.10
4.33
4.31
4.31
4.29
4.28
4.27
4.26
4.24
4.24
4.22
3.80
3.77
3.75
2.43
2.39
2.38
2.37
2.36
2.33
2.10
1.69
1.66
1.64
1.32
1.30
1.28
1.25
1.01
2.02
2.29
0.95
2.00
1.99
2.90
2.00
3.10
1.97
9.01
2.78
f1 (ppm)

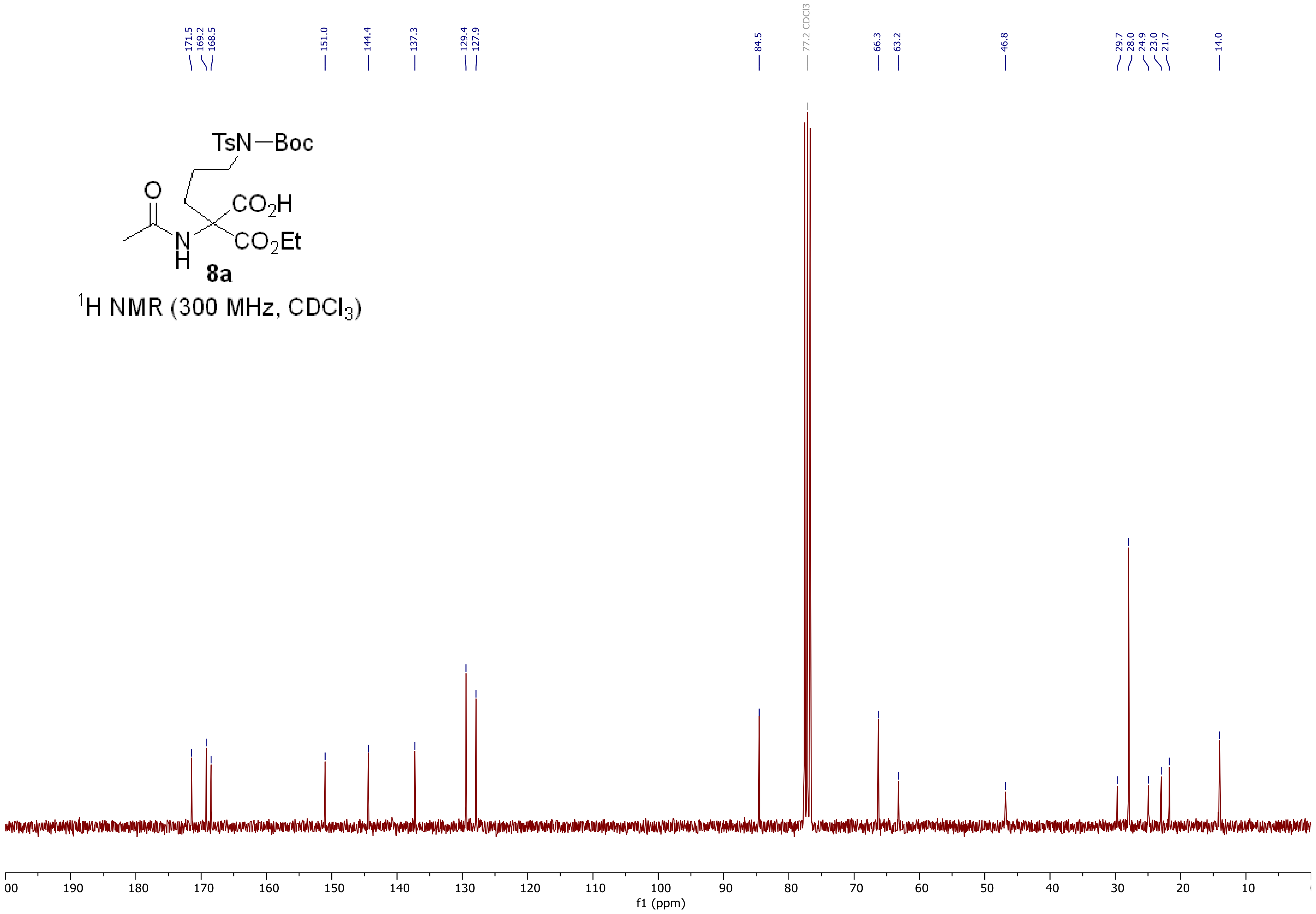
171.5
169.2
168.5
151.0
144.4
137.3
129.4
127.9
84.5
77.2 CDCl3
66.3
63.2
46.8
29.7
28.0
24.9
23.0
21.7
14.0
TsN—Boc
O
N
H
CO2H
CO2Et
8a
1H NMR (300 MHz, CDCl3)
190
180
170
160
150
140
130
120
110
100
90
80
70
60
50
40
30
20
10
f1 (ppm)

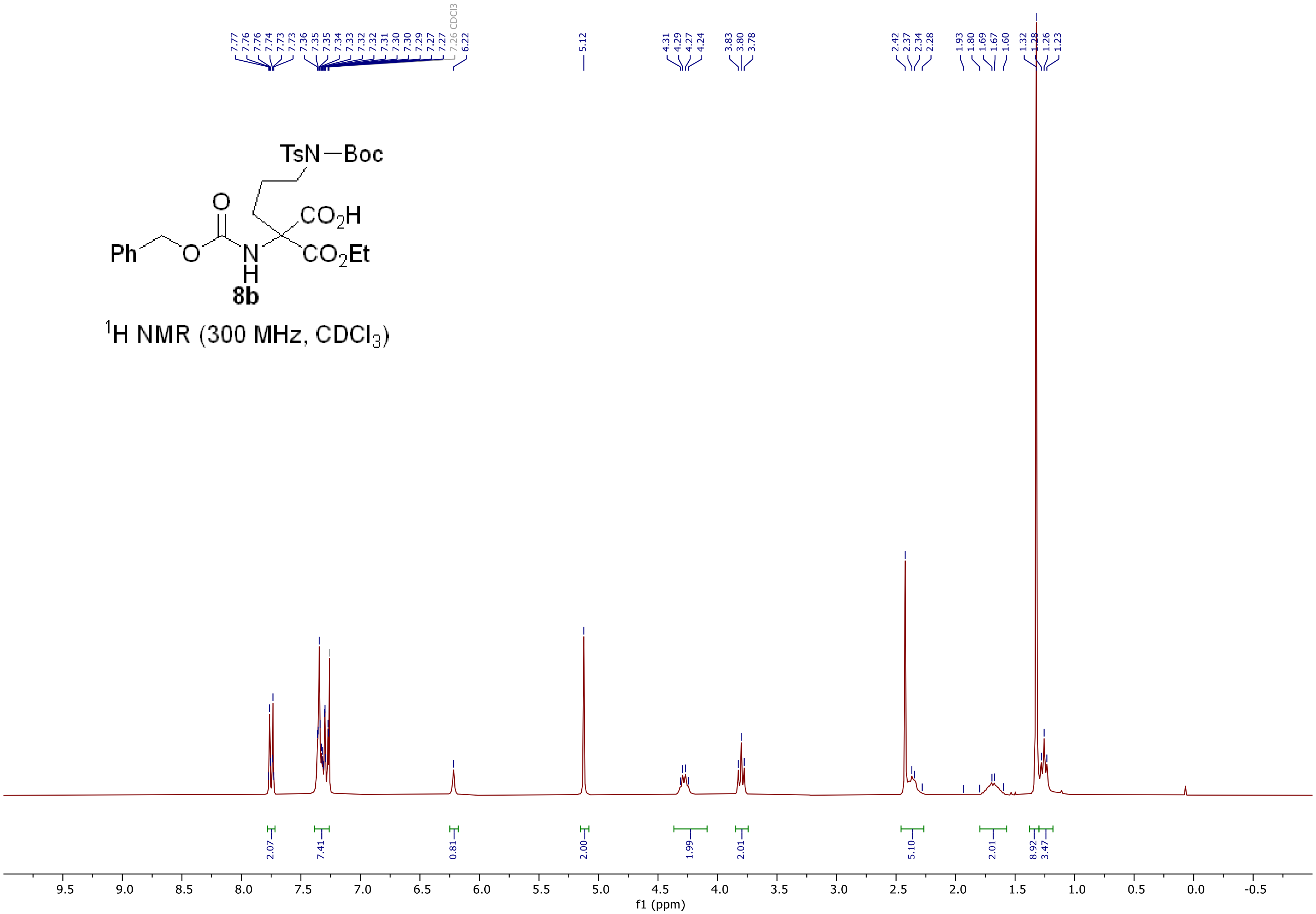

TsN—Boc
CO2H
CO2Et
Ph O N H O
8b
1H NMR (300 MHz, CDCl3)
7.77 7.76 7.76 7.74 7.73 7.73 7.36 7.35 7.35 7.34 7.33 7.32 7.32 7.31 7.30 7.30 7.29 7.27 7.27 7.26 CDCl3 6.22
5.12
4.31 4.29 4.27 4.24 3.83 3.80 3.78
2.42 2.37 2.34 2.28 1.93 1.80 1.69 1.67 1.60 1.32 1.28 1.26 1.23
2.07 7.41 0.81 2.00 1.99 2.01 5.10 2.01 8.92 3.47
9.5 9.0 8.5 8.0 7.5 7.0 6.5 6.0 5.5 5.0 4.5 4.0 3.5 3.0 2.5 2.0 1.5 1.0 0.5 0.0 -0.5
f1 (ppm)

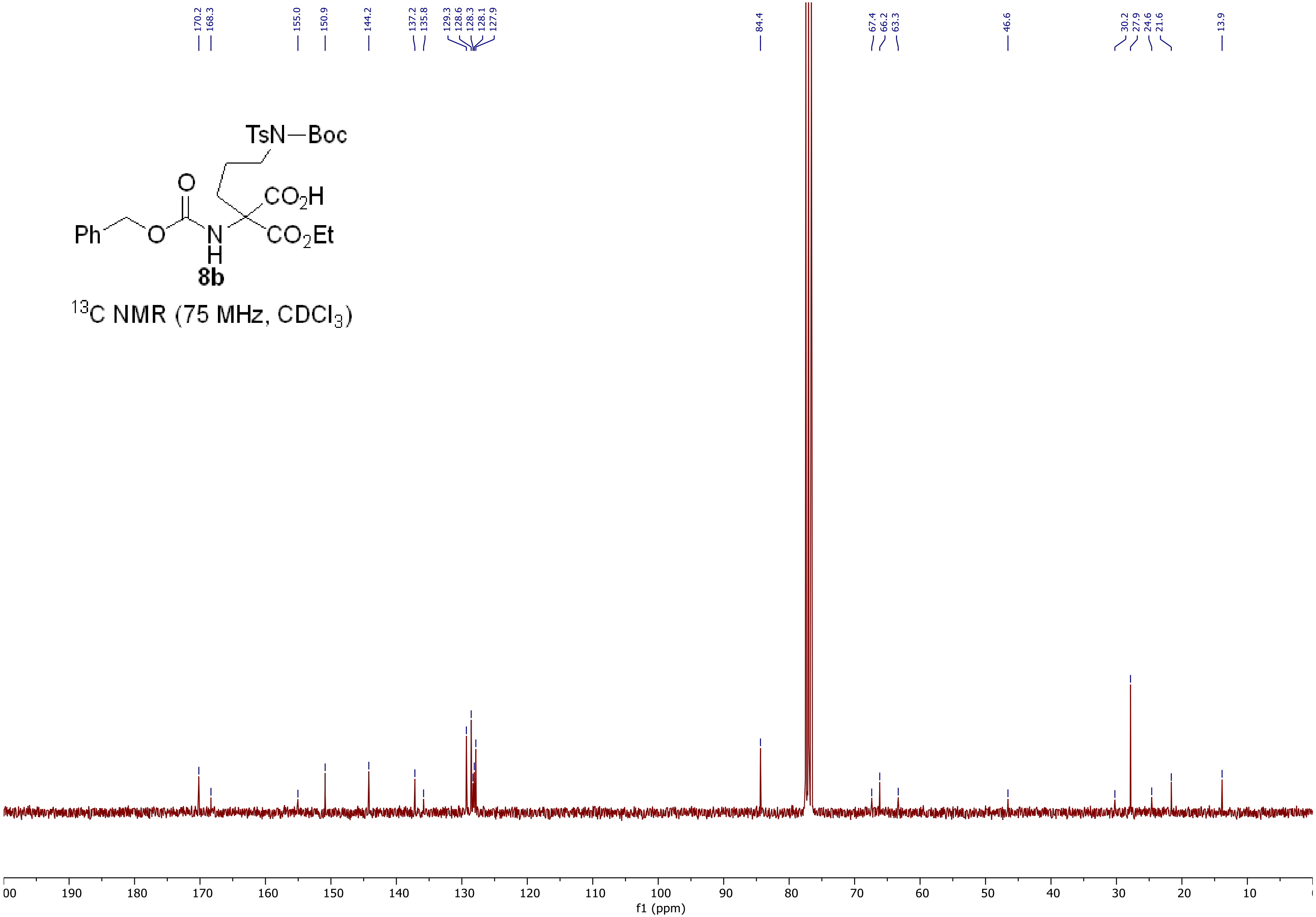
TsN—Boc
CO2H
CO2Et
Ph
O
N
H
8b
13C NMR (75 MHz, CDCl3)
170.2
168.3
155.0
150.9
144.2
137.2
135.8
129.3
128.6
128.3
128.1
127.9
84.4
67.4
66.2
63.3
46.6
30.2
27.9
24.6
21.6
13.9
00
190
180
170
160
150
140
130
120
110
100
90
80
70
60
50
40
30
20
10
f1 (ppm)

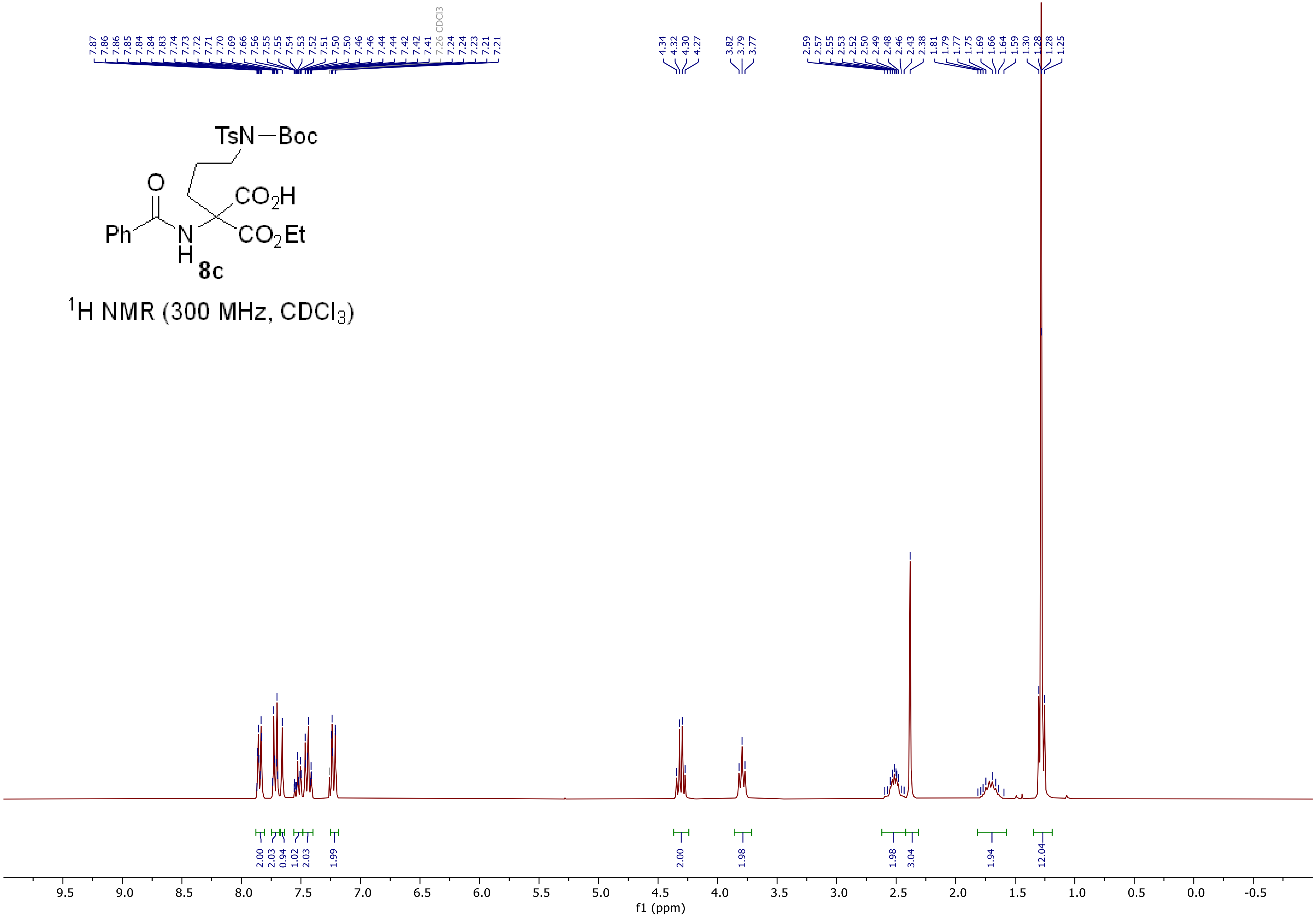
TsN—Boc
O
CO2H
Ph
N
H
CO2Et
8c
1H NMR (300 MHz, CDCl3)
f1 (ppm)

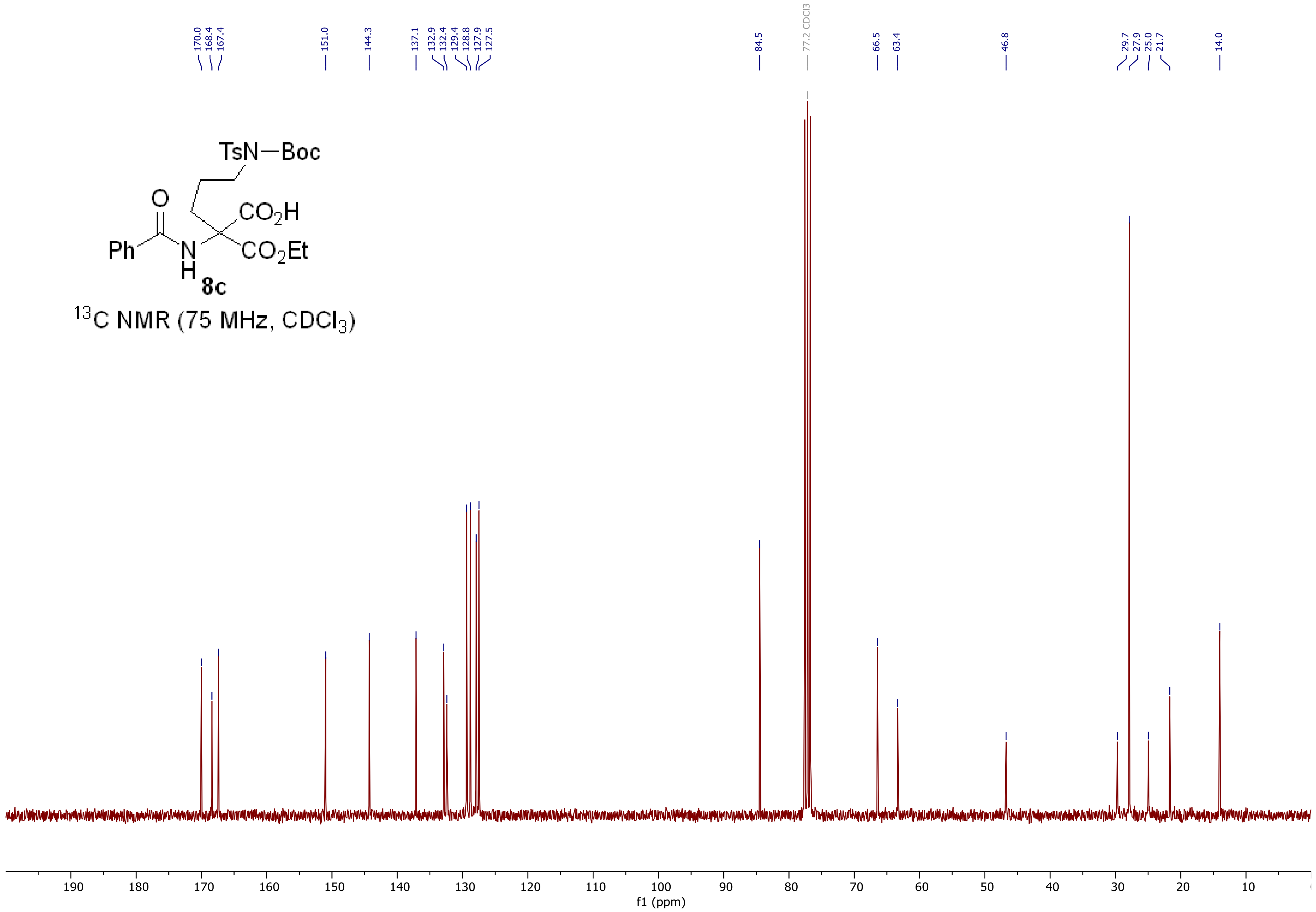
TsN—Boc
O
Ph
N
H
CO2H
CO2Et
8c
13C NMR (75 MHz, CDCl3)
170.0
168.4
167.4
151.0
144.3
137.1
132.9
132.4
129.4
128.8
127.9
127.5
84.5
77.2 CDCl3
66.5
63.4
46.8
29.7
27.9
25.0
21.7
14.0
190
180
170
160
150
140
130
120
110
100
90
80
70
60
50
40
30
20
10
f1 (ppm)

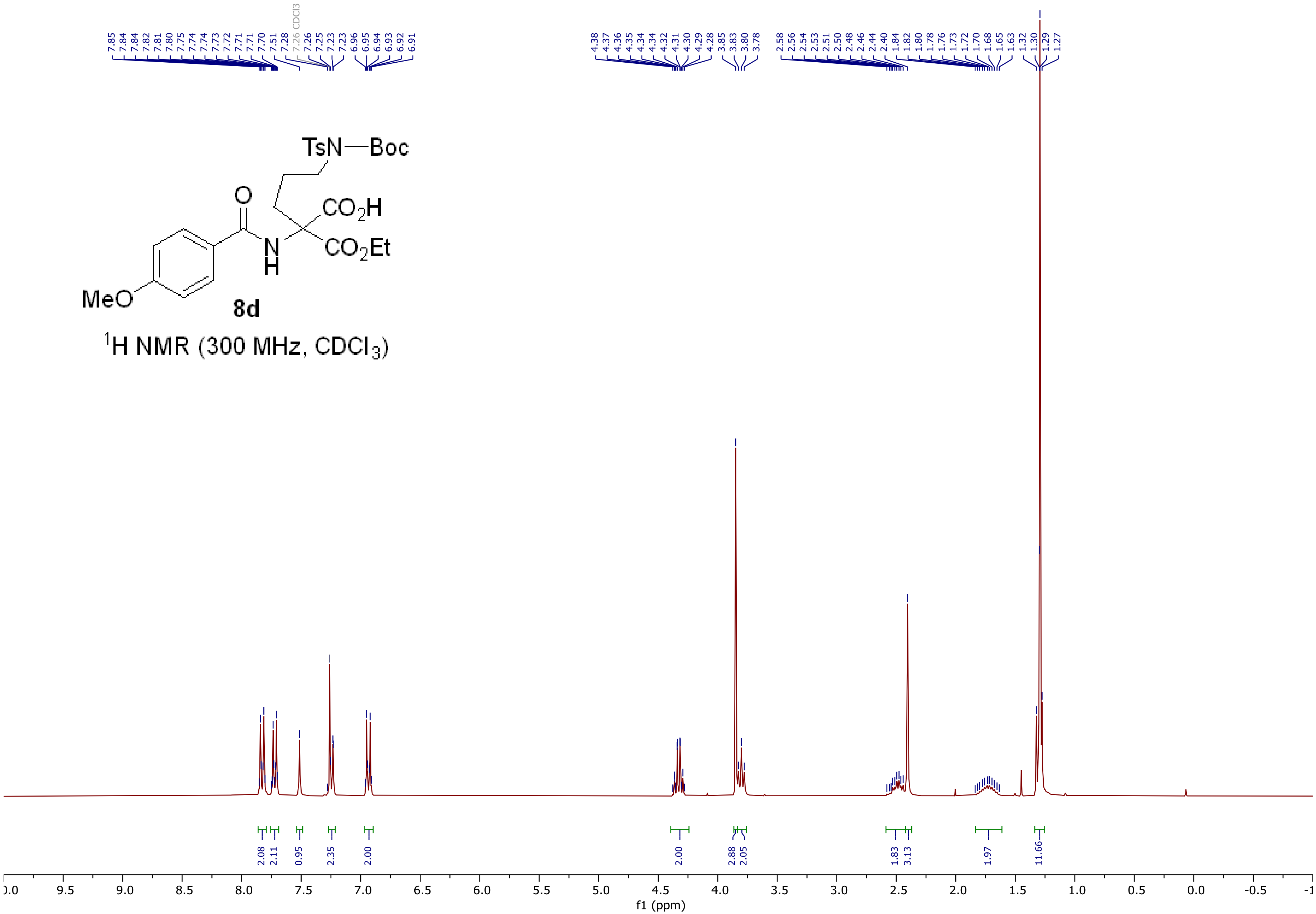
TsN—Boc
CO2H
CO2Et
MeO
8d
1H NMR (300 MHz, CDCl3)
f1 (ppm)

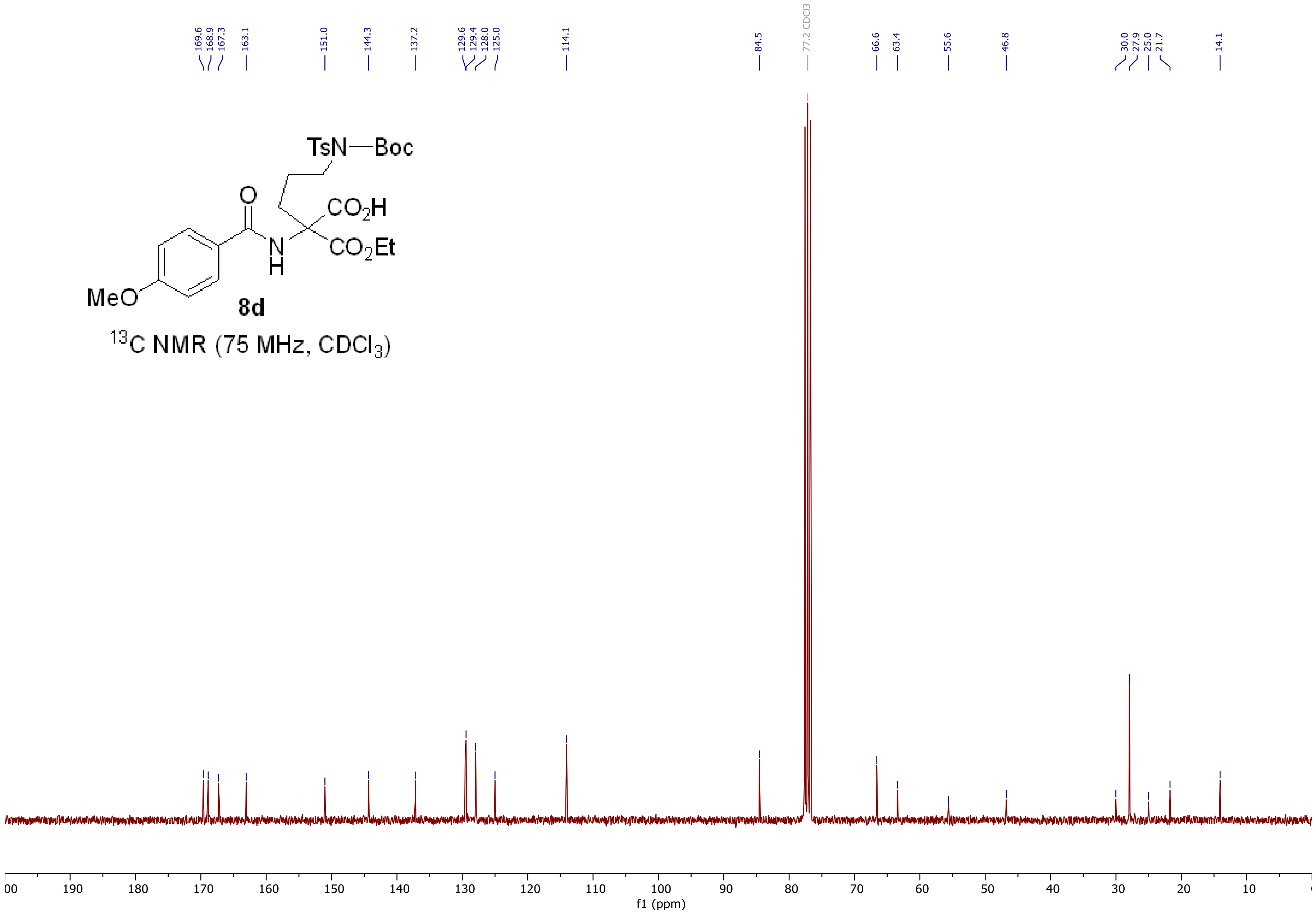

169.6
168.9
167.3
163.1
151.0
144.3
137.2
129.6
129.4
128.0
125.0
114.1
84.5
77.2 CDCl3
66.6
63.4
55.6
46.8
30.0
27.9
25.0
21.7
14.1
TsN—Boc
CO2H
CO2Et
MeO
8d
13C NMR (75 MHz, CDCl3)
f1 (ppm)

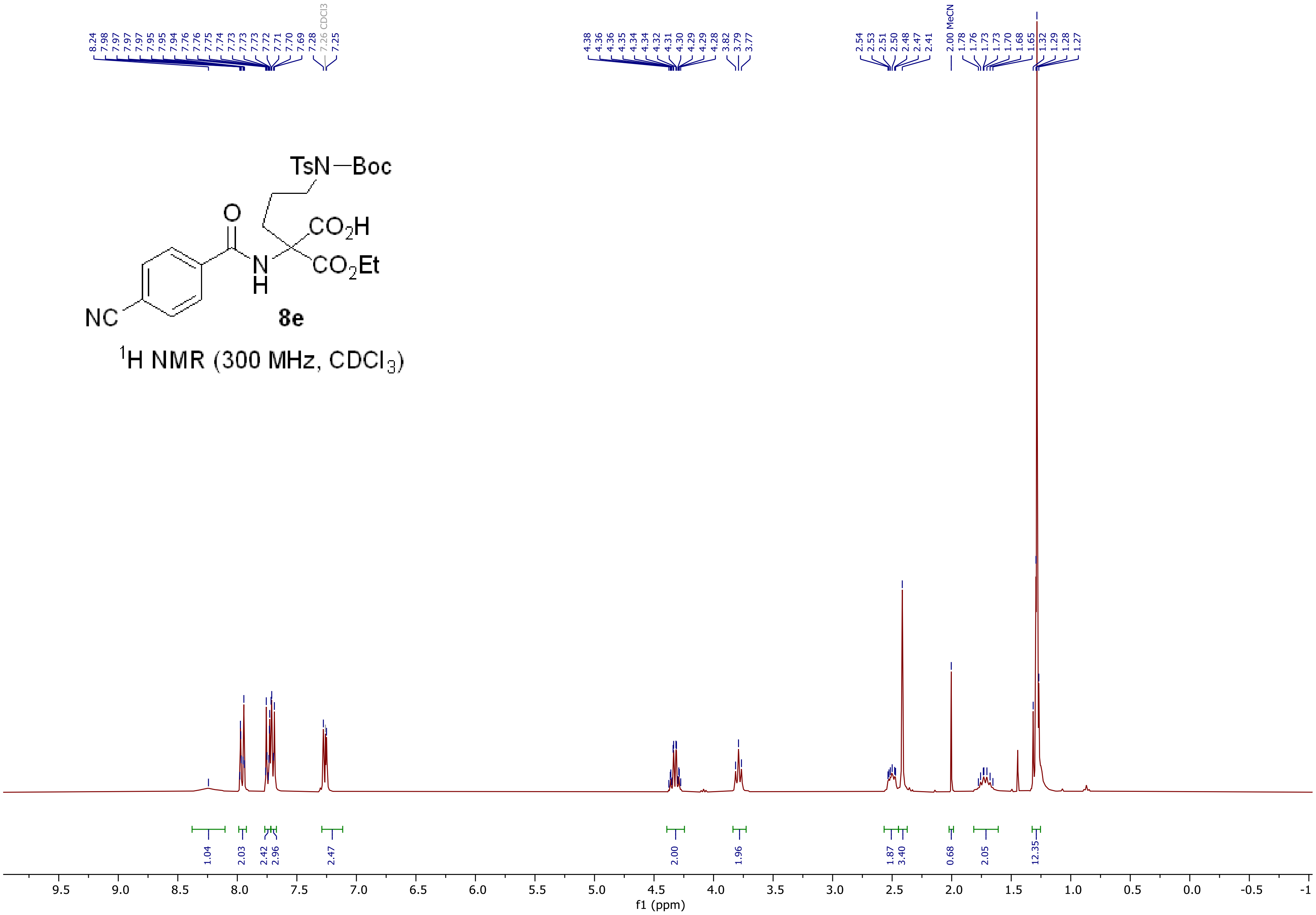
TsN—Boc
O
N
H
CO_2H
CO_2Et
NC
8e
^{1}H NMR (300 MHz, $CDCl_3$)
8.24 7.98 7.97 7.97 7.97 7.95 7.94 7.76 7.75 7.74 7.73 7.73 7.73 7.72 7.71 7.70 7.69 7.28 7.26 CDCl3 7.25
4.38 4.36 4.36 4.35 4.34 4.34 4.32 4.31 4.30 4.29 4.29 4.28 3.82 3.79 3.77
2.54 2.53 2.51 2.50 2.48 2.47 2.41 2.00 MeCN 1.78 1.76 1.73 1.73 1.70 1.68 1.65 1.32 1.29 1.28 1.27
1.04 2.03 2.42 2.96 2.47 2.00 1.96 1.87 3.40 0.68 2.05 12.35
9.5 9.0 8.5 8.0 7.5 7.0 6.5 6.0 5.5 5.0 4.5 4.0 3.5 3.0 2.5 2.0 1.5 1.0 0.5 0.0 -0.5 -1
f1 (ppm)

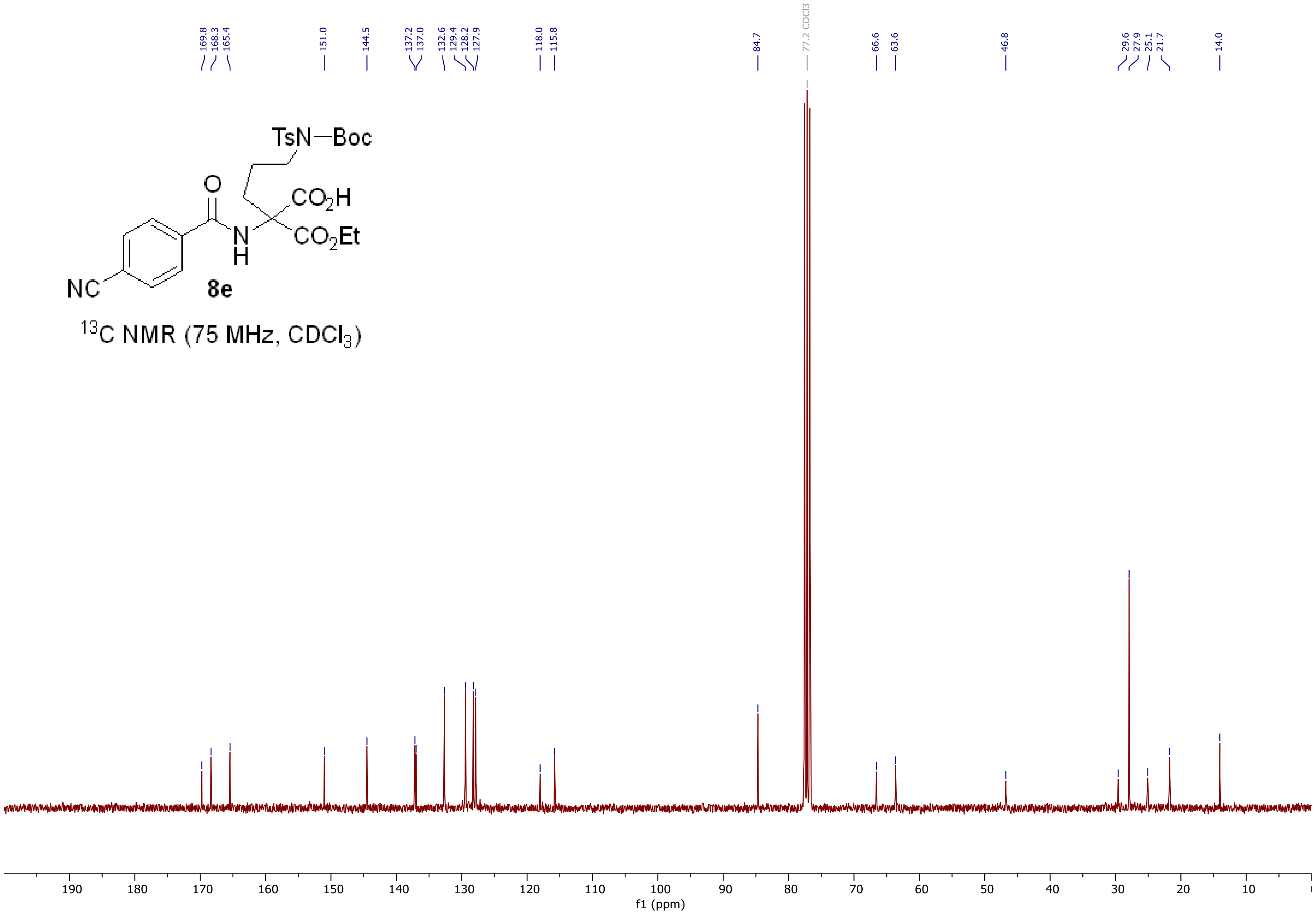
TsN—Boc
O
CO2H
N
H
CO2Et
NC
8e
13C NMR (75 MHz, CDCl3)
169.8
168.3
165.4
151.0
144.5
137.2
137.0
132.6
129.4
128.2
127.9
118.0
115.8
84.7
77.2 CDCl3
66.6
63.6
46.8
29.6
27.9
25.1
21.7
14.0
f1 (ppm)

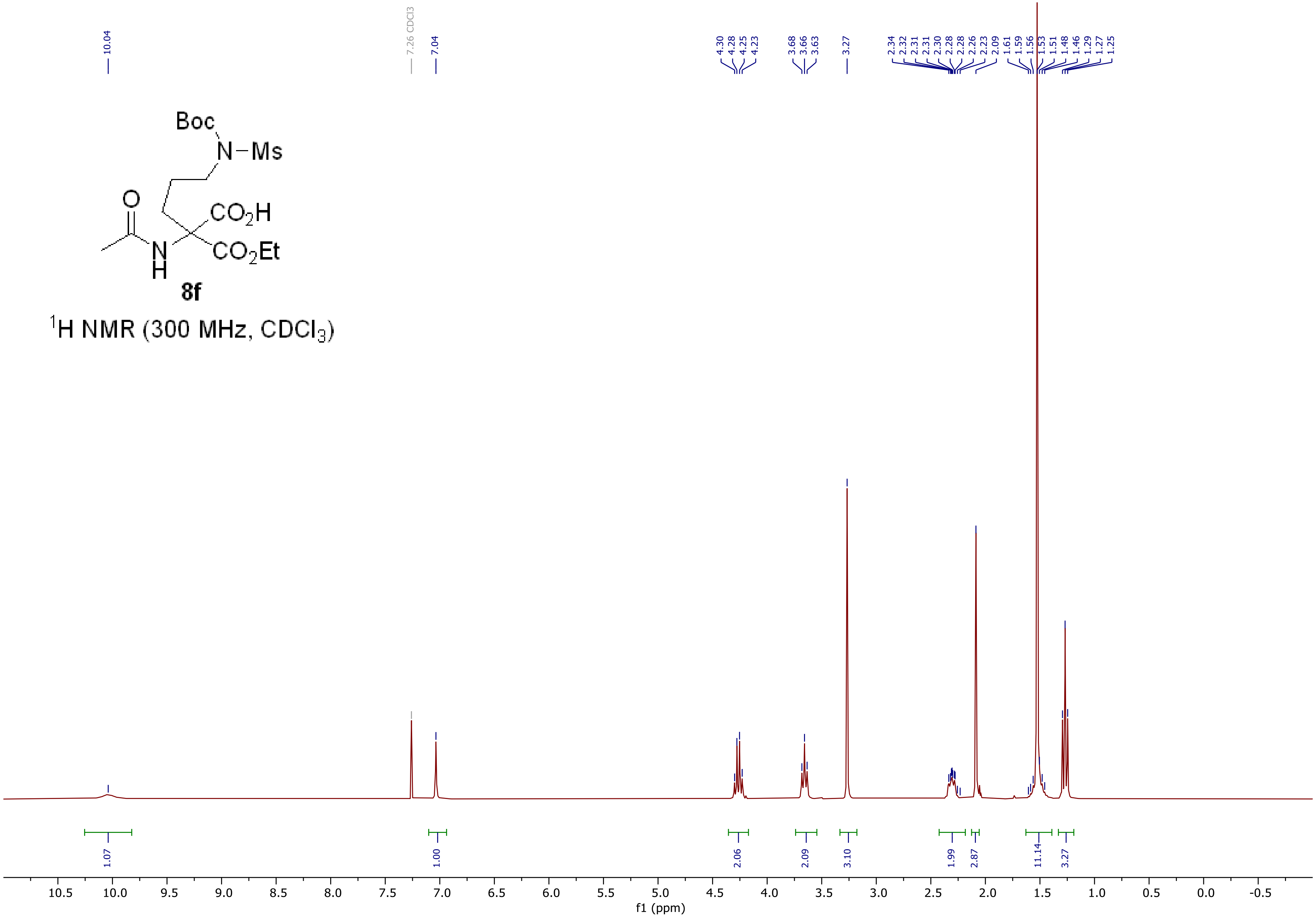

Boc
N−Ms
O
CO2H
N
H
CO2Et
8f
1H NMR (300 MHz, CDCl3)
10.04
7.26 CDCl3
7.04
4.30
4.28
4.25
4.23
3.68
3.66
3.63
3.27
2.34
2.32
2.31
2.31
2.30
2.28
2.28
2.26
2.23
2.09
1.61
1.59
1.56
1.53
1.51
1.48
1.46
1.29
1.27
1.25
1.07
1.00
2.06
2.09
3.10
1.99
2.87
11.14
3.27
10.5 10.0 9.5 9.0 8.5 8.0 7.5 7.0 6.5 6.0 5.5 5.0 4.5 4.0 3.5 3.0 2.5 2.0 1.5 1.0 0.5 0.0 -0.5
f1 (ppm)

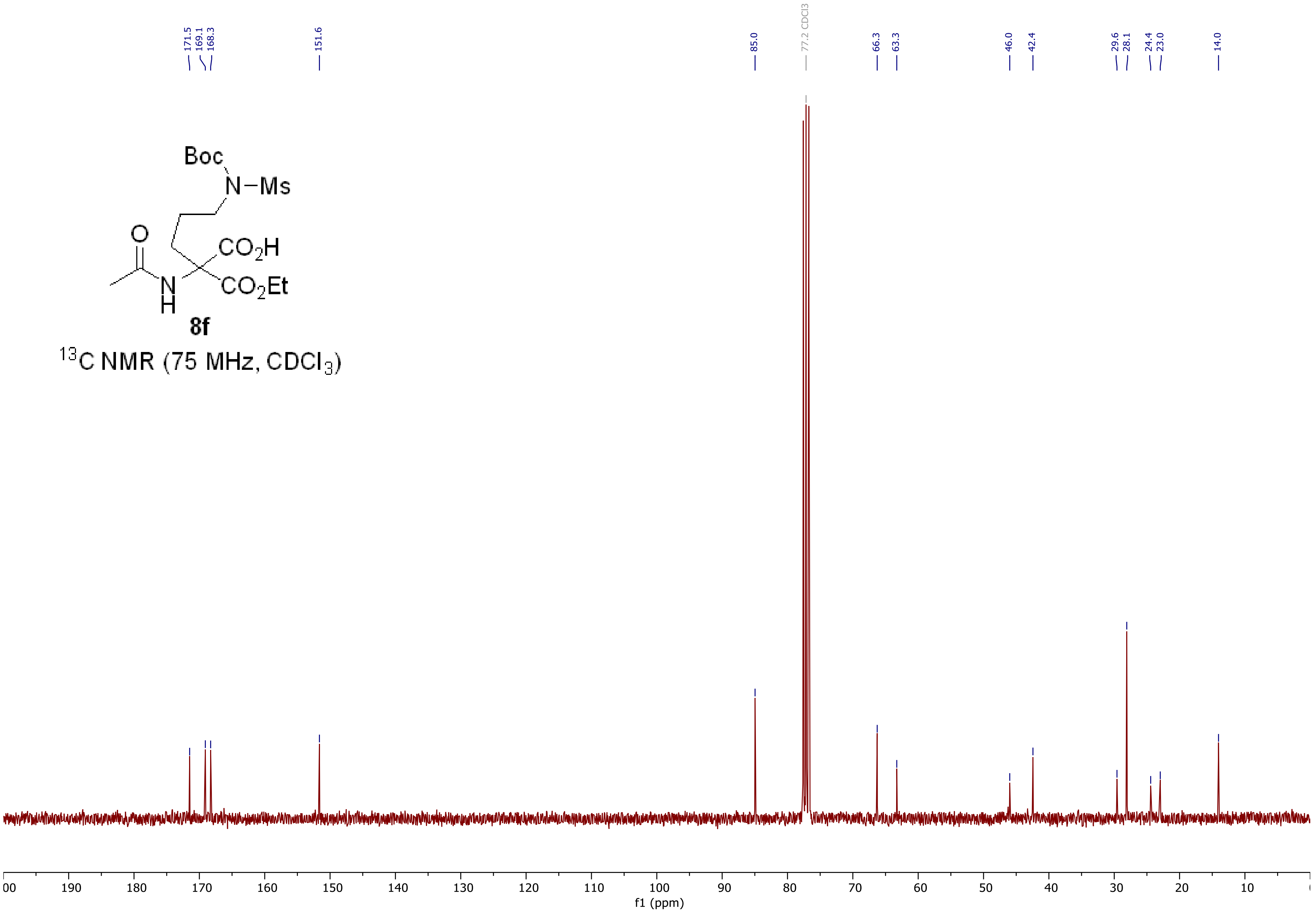

171.5
169.1
168.3
151.6
85.0
77.2 CDCl3
66.3
63.3
46.0
42.4
29.6
28.1
24.4
23.0
14.0
Boc
N−Ms
O
CO2H
N
H
CO2Et
8f
13C NMR (75 MHz, CDCl3)
190
180
170
160
150
140
130
120
110
100
90
80
70
60
50
40
30
20
10
f1 (ppm)

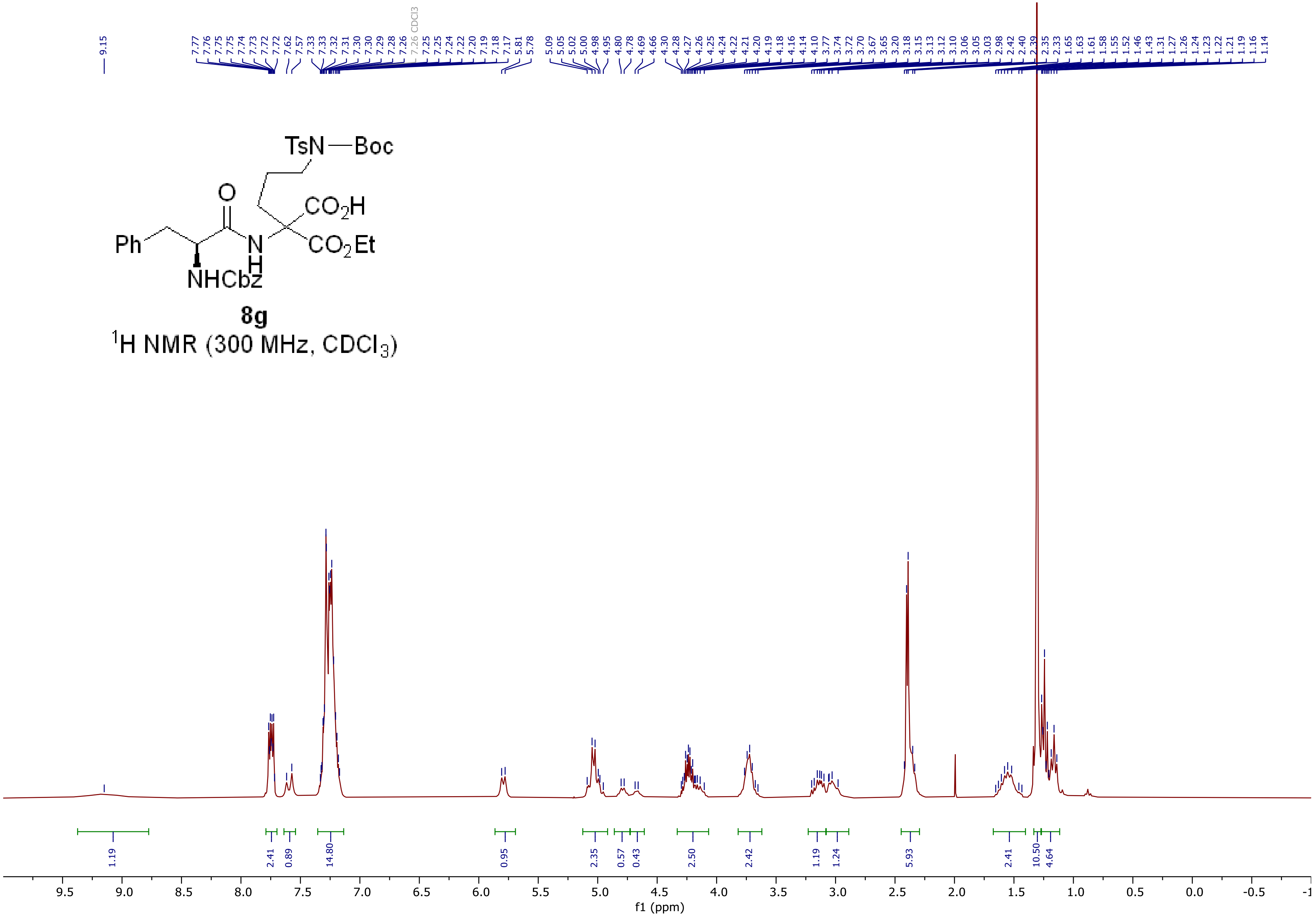
TsN—Boc
CO2H
CO2Et
Ph
N
H
O
NHCbz
8g
1H NMR (300 MHz, CDCl3)
f1 (ppm)

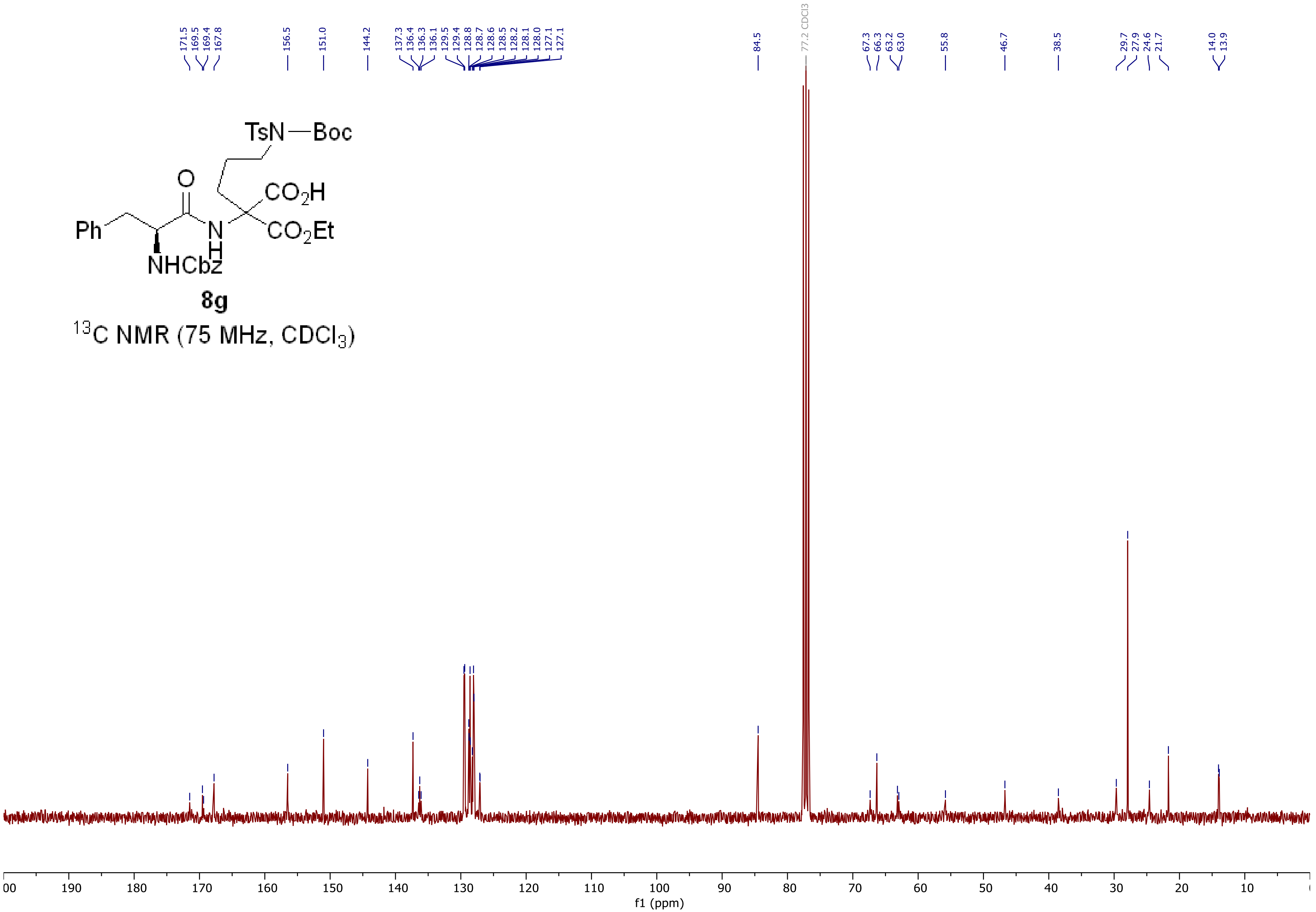
171.5
169.5
169.4
167.8
156.5
151.0
144.2
137.3
136.4
136.3
136.1
129.5
129.4
128.8
128.7
128.6
128.5
128.2
128.1
128.0
127.1
127.1
84.5
77.2 CDCl3
67.3
66.3
63.2
63.0
55.8
46.7
38.5
29.7
27.9
24.6
21.7
14.0
13.9
TsN—Boc
O
CO2H
Ph
N
H
CO2Et
NHCbz
8g
13C NMR (75 MHz, CDCl3)
00
190
180
170
160
150
140
130
120
110
100
90
80
70
60
50
40
30
20
10
f1 (ppm)

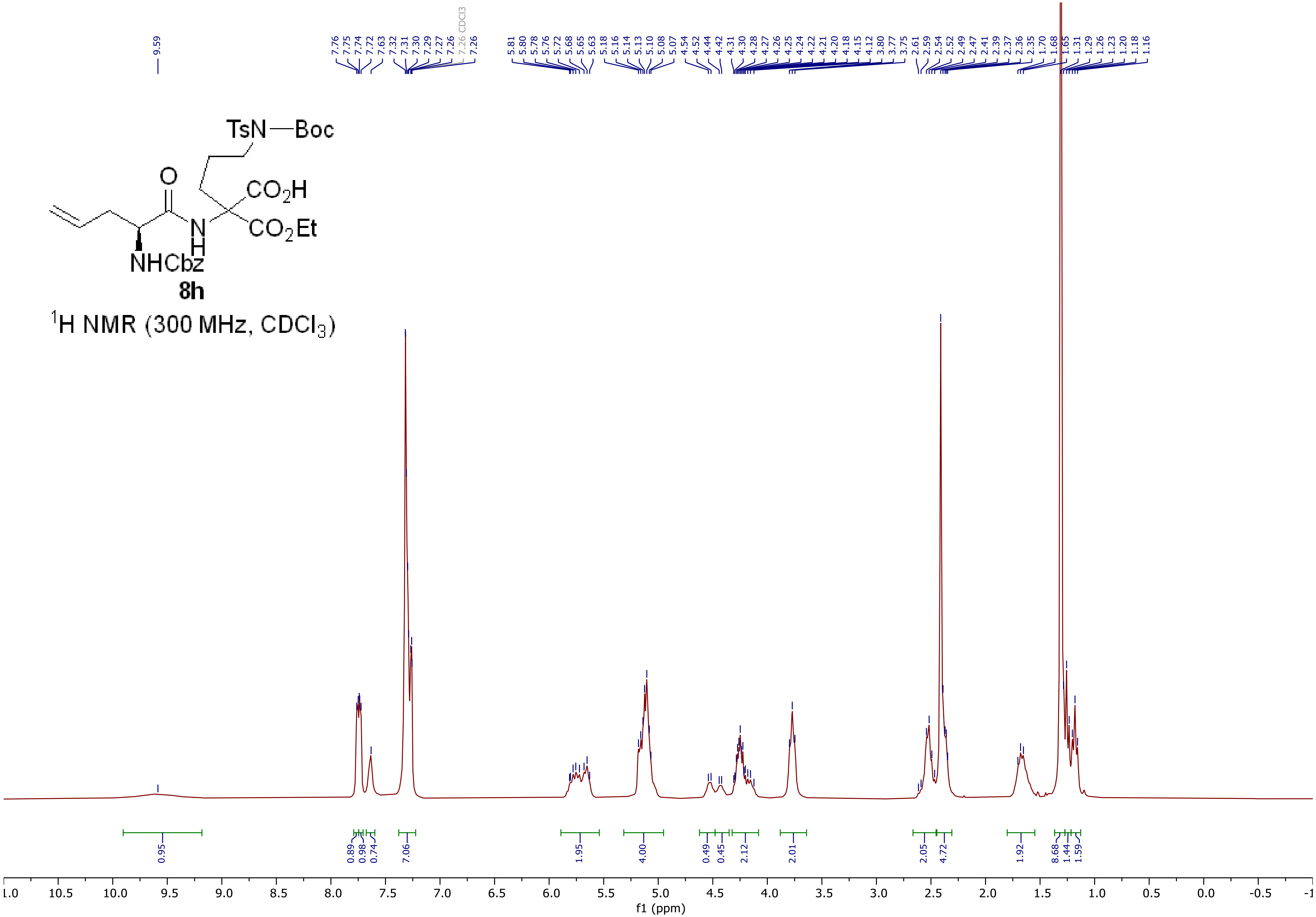
TsN—Boc
CO2H
CO2Et
NHCbz
8h
1H NMR (300 MHz, CDCl3)
f1 (ppm)

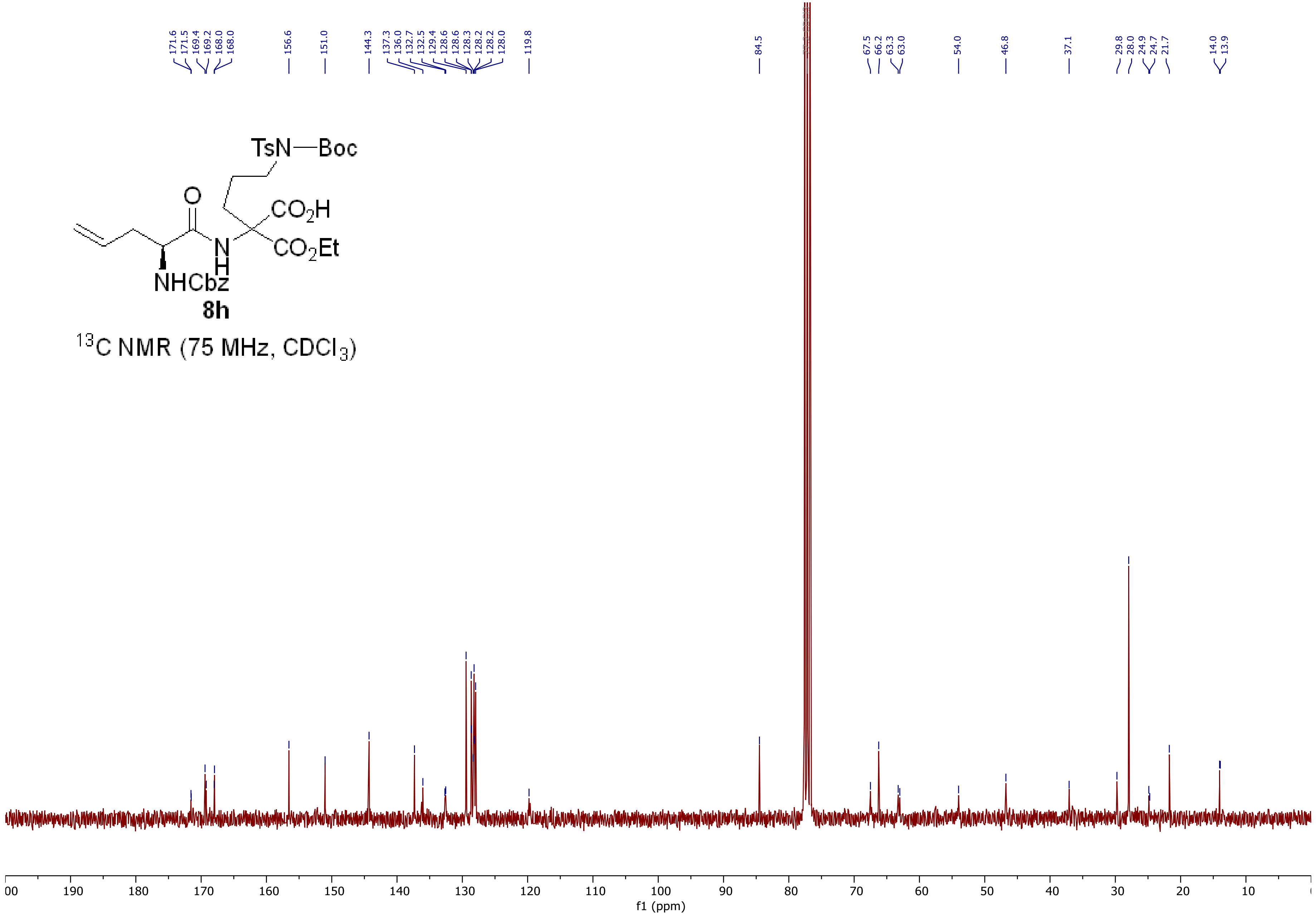
171.6
171.5
169.4
169.2
168.0
168.0
156.6
151.0
144.3
137.3
136.0
132.7
132.5
129.4
128.6
128.6
128.3
128.2
128.2
128.0
119.8
84.5
67.5
66.2
63.3
63.0
54.0
46.8
37.1
29.8
28.0
24.9
24.7
21.7
14.0
13.9
TsN—Boc
O
CO2H
N
H
CO2Et
NHCbz
8h
13C NMR (75 MHz, CDCl3)
00
190
180
170
160
150
140
130
120
110
100
90
80
70
60
50
40
30
20
10
f1 (ppm)

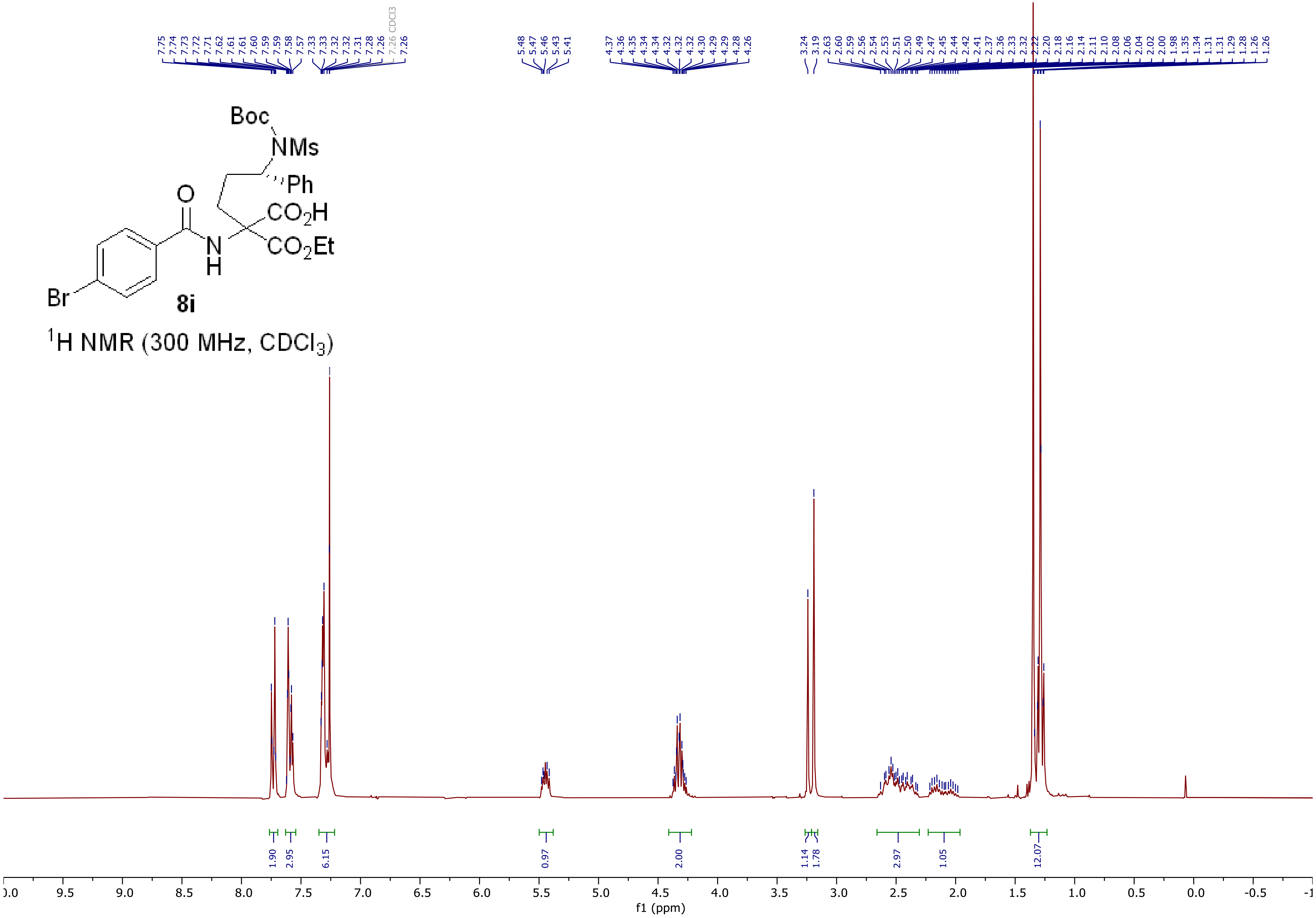
Boc
NMs
Ph
O
CO2H
N
H
CO2Et
Br
8i
1H NMR (300 MHz, CDCl3)
f1 (ppm)

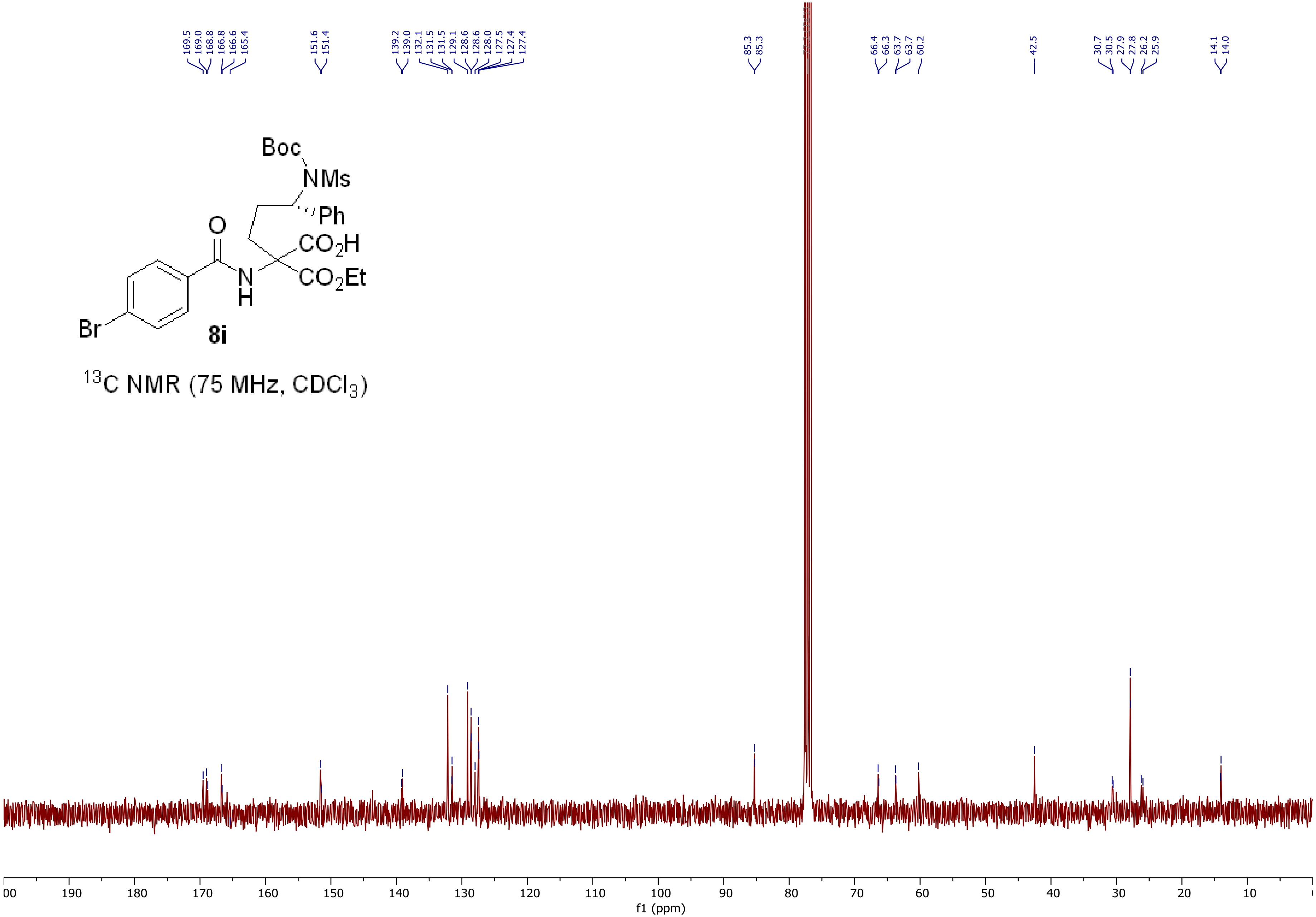

169.5
169.0
168.8
166.8
166.6
165.4
151.6
151.4
139.2
139.0
132.1
131.5
131.5
129.1
128.6
128.6
128.0
127.5
127.4
127.4
85.3
85.3
66.4
66.3
63.7
63.7
60.2
42.5
30.7
30.5
27.9
27.8
26.2
25.9
14.1
14.0
Boc
NMs
Ph
O
CO_2H
CO_2Et
N
H
Br
8i
^{13}C NMR (75 MHz, $CDCl_3$)
f1 (ppm)

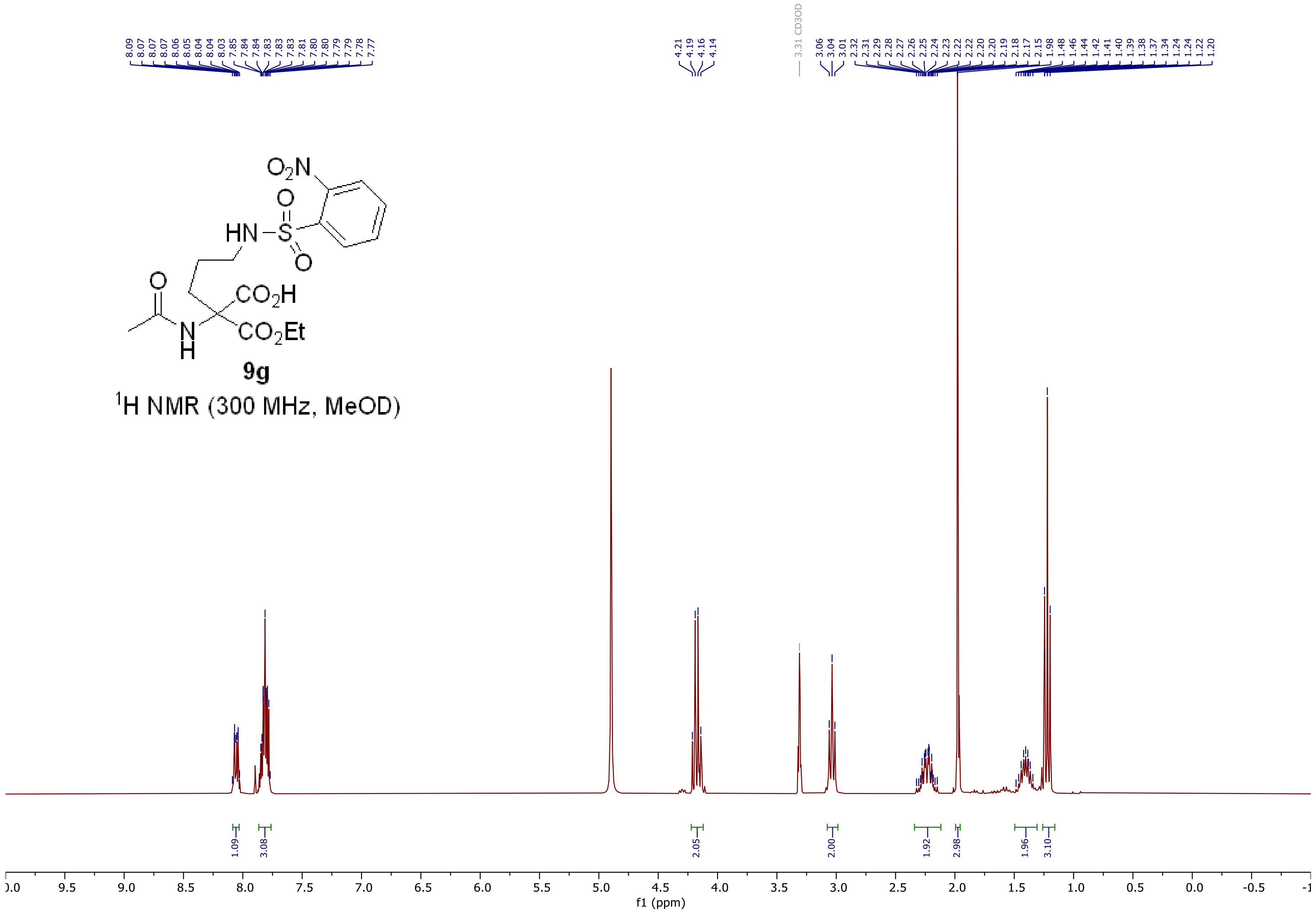
O2N
HN
S
O
CO2H
CO2Et
N
H
9g
1H NMR (300 MHz, MeOD)
8.09 8.07 8.07 8.06 8.05 8.04 8.04 8.03 7.85 7.84 7.83 7.83 7.83 7.81 7.80 7.79 7.79 7.78 7.77
4.21 4.19 4.16 4.14
3.31 CD3OD
3.06 3.04 3.01 2.32 2.31 2.29 2.28 2.27 2.26 2.25 2.24 2.23 2.22 2.22 2.22 2.20 2.20 2.19 2.18 2.17 2.15 1.98 1.48 1.46 1.44 1.42 1.41 1.40 1.39 1.38 1.37 1.34 1.24 1.24 1.22 1.20
1.09 3.08 2.05 2.00 1.92 2.98 1.96 3.10
f1 (ppm)

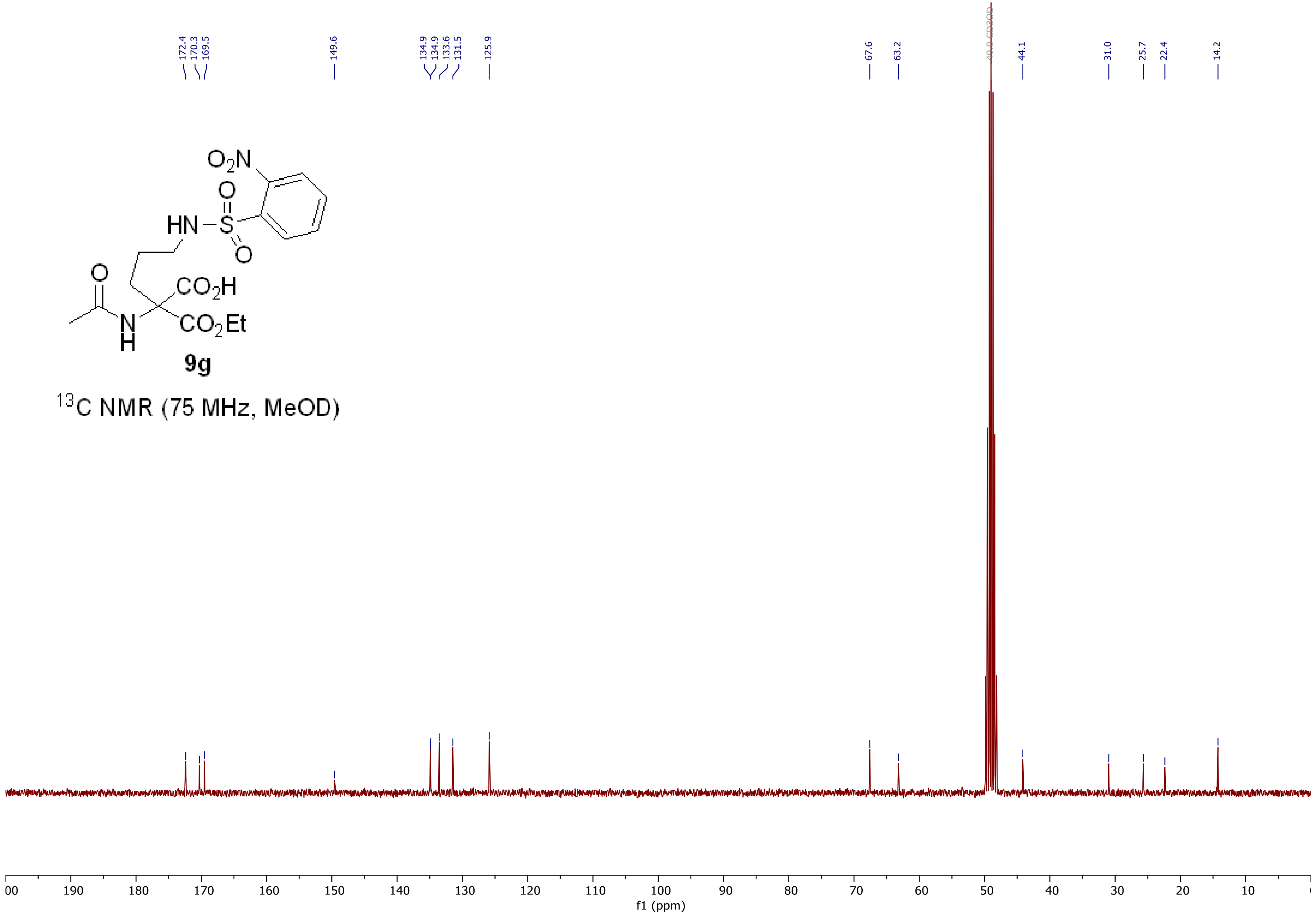
172.4
170.3
169.5
149.6
134.9
134.9
133.6
131.5
125.9
67.6
63.2
49.0 CD3OD
44.1
31.0
25.7
22.4
14.2
O_2N
HN
S
O
O
CO_2H
CO_2Et
N
H
9g
^{13}C NMR (75 MHz, MeOD)
00
190
180
170
160
150
140
130
120
110
100
90
80
70
60
50
40
30
20
10
f1 (ppm)

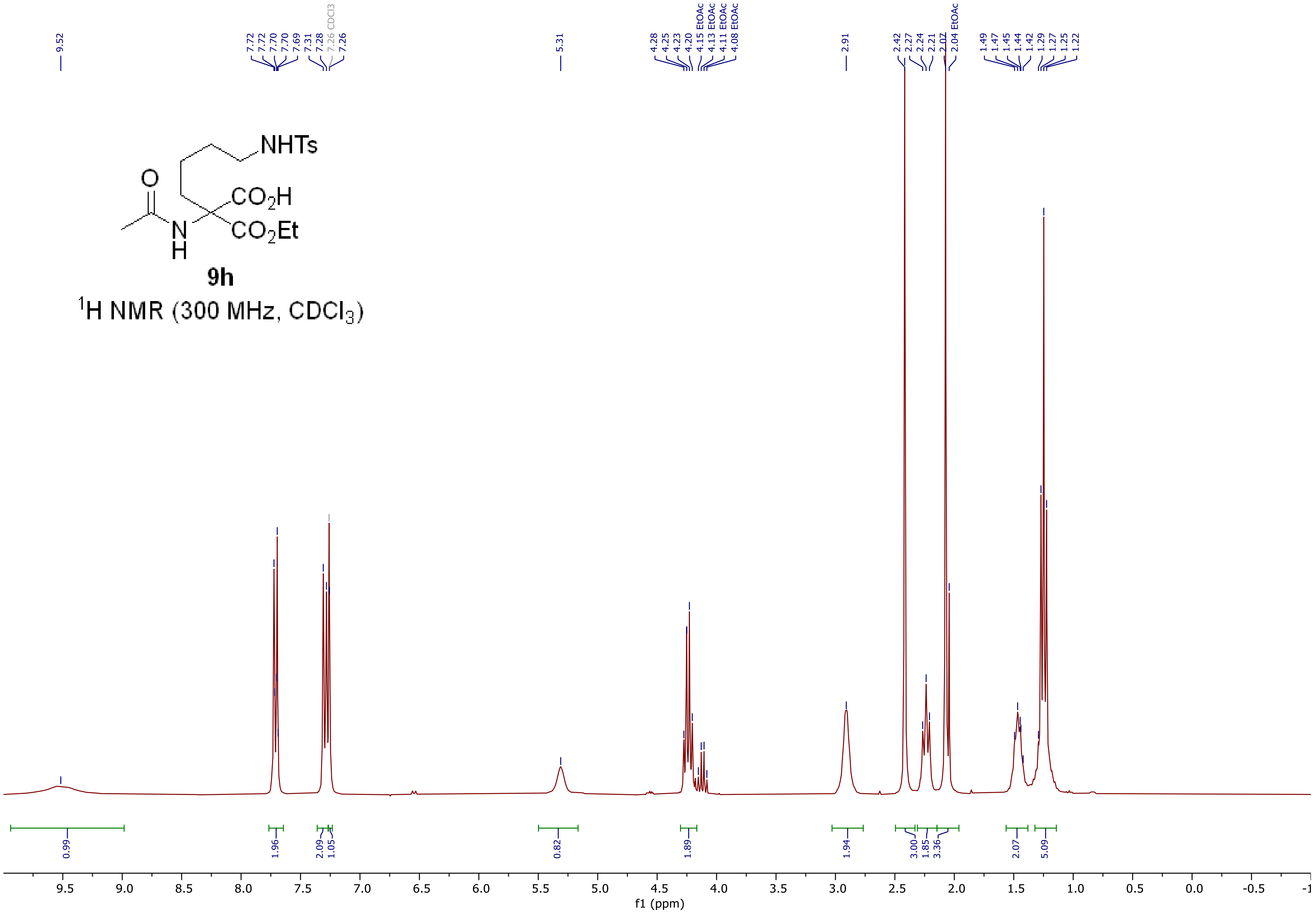

NHTs
O
CO_2H
N
H
CO_2Et
9h
^1H NMR (300 MHz, $CDCl_3$)
f1 (ppm)

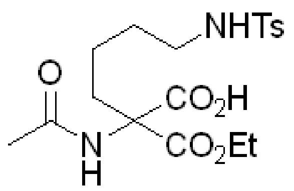

9h

^{13}C NMR (75 MHz, $CDCl_3$)

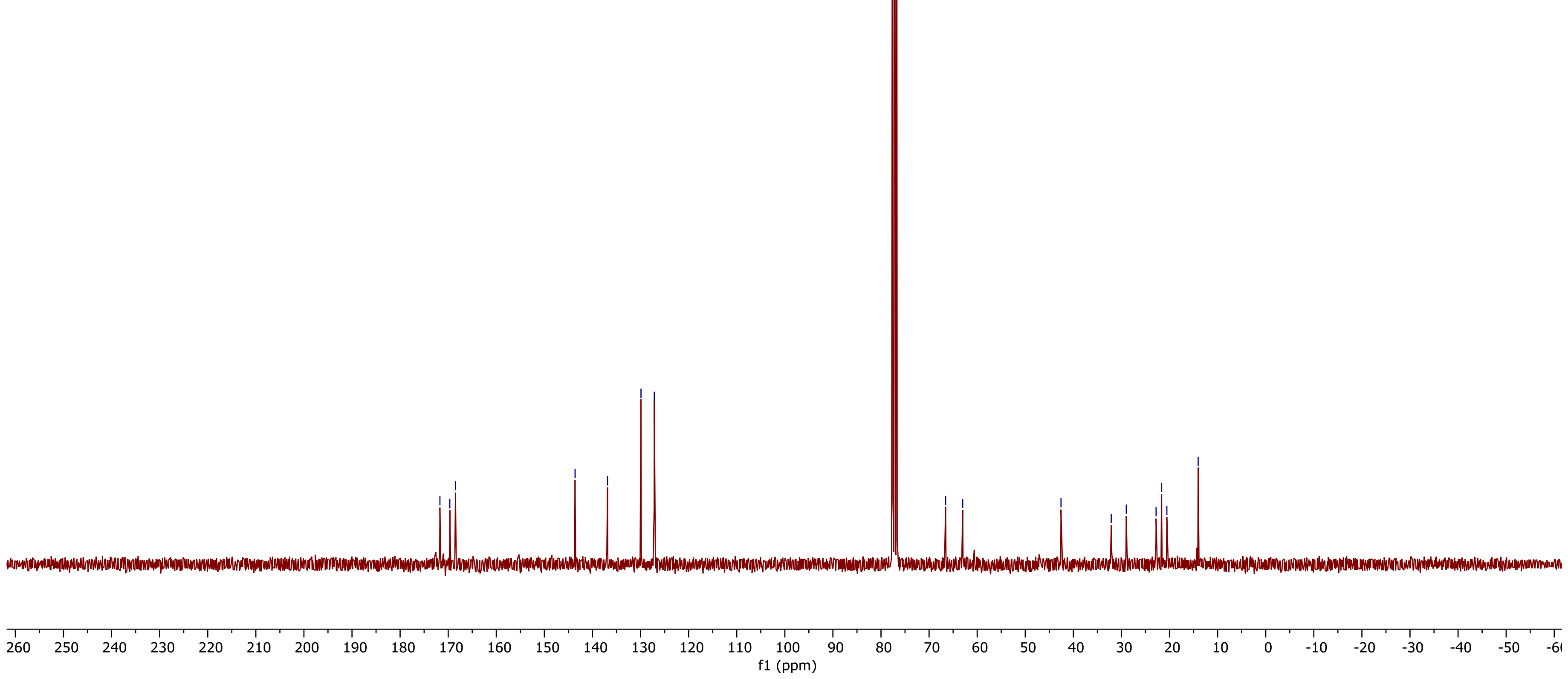

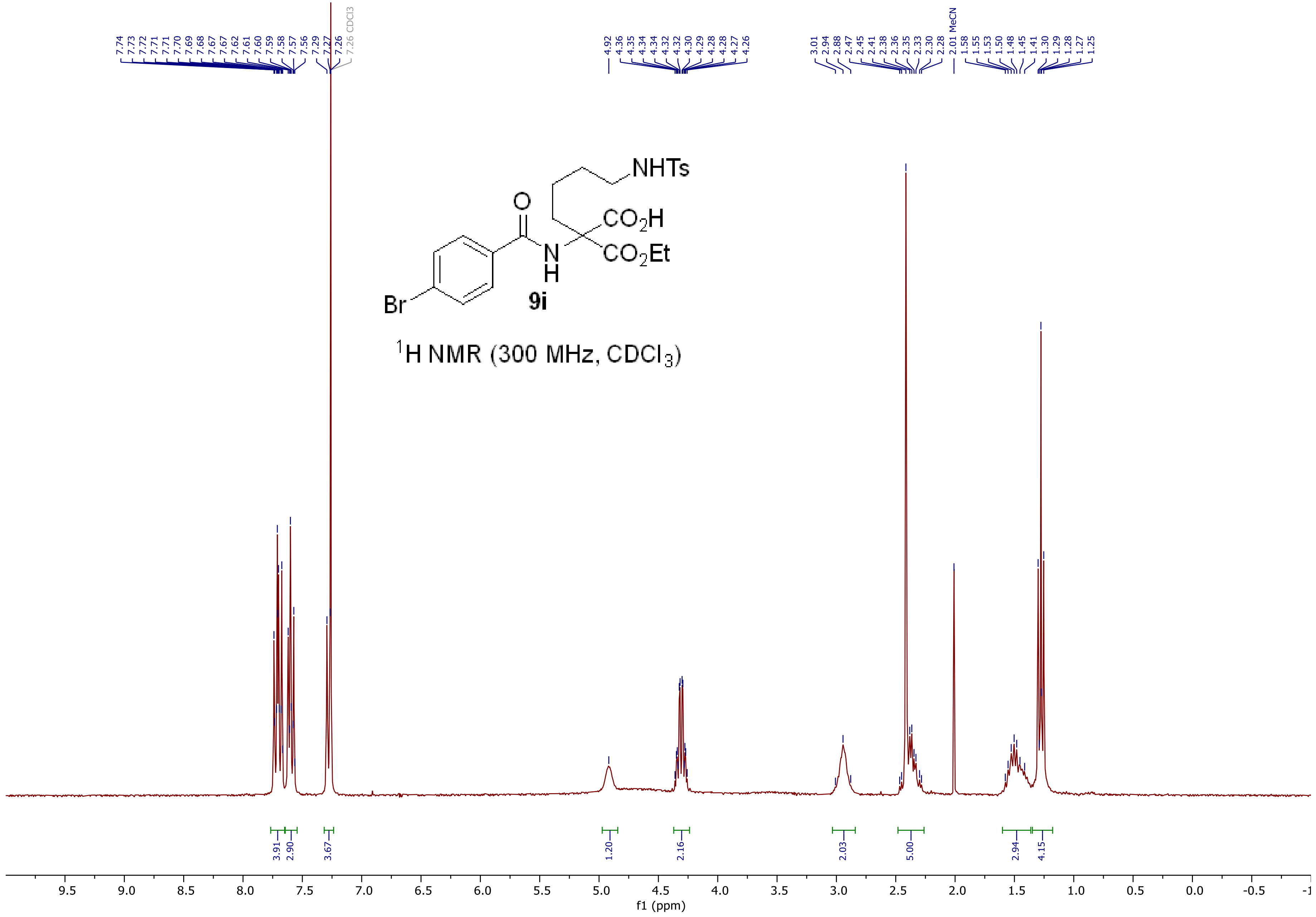
NHTs
CO2H
CO2Et
Br
9i
1H NMR (300 MHz, CDCl3)
f1 (ppm)

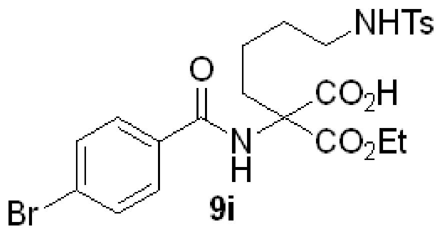

9i

^{13}C NMR (75 MHz, $CDCl_3$)

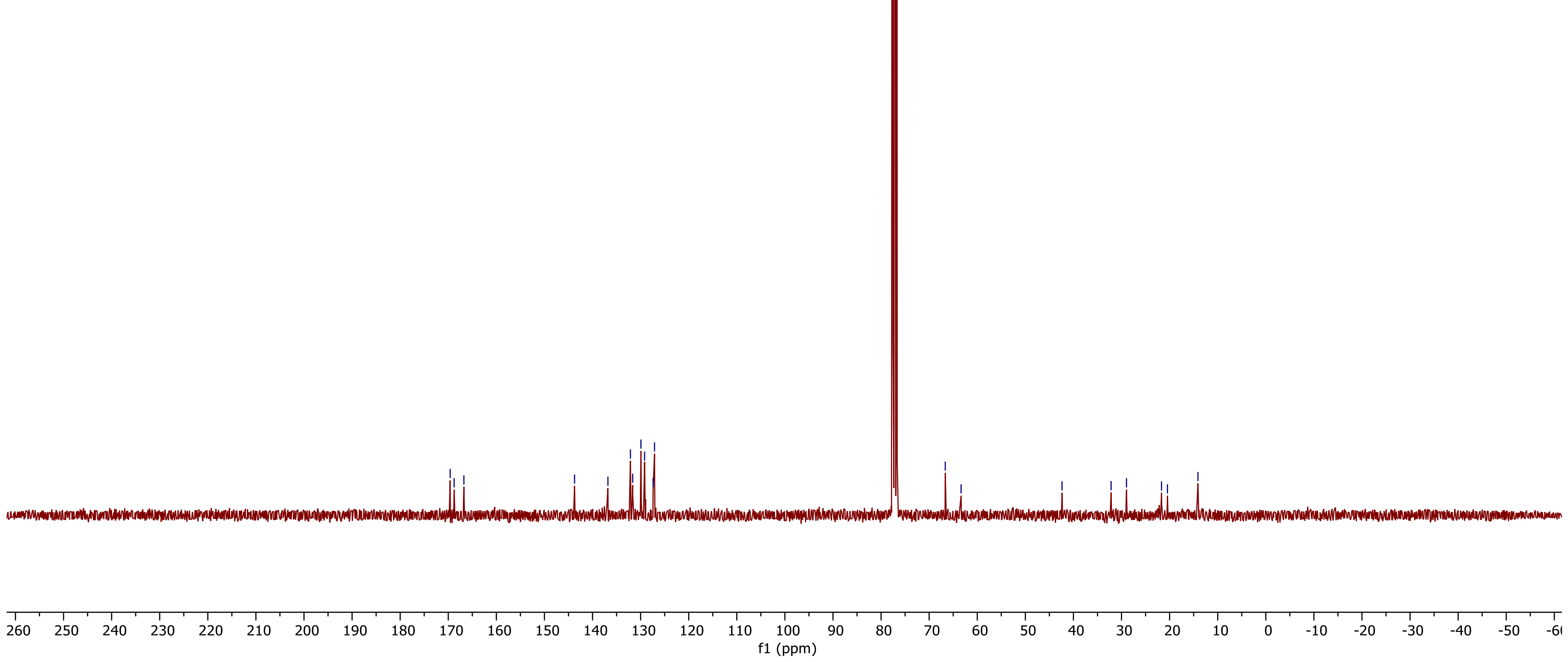

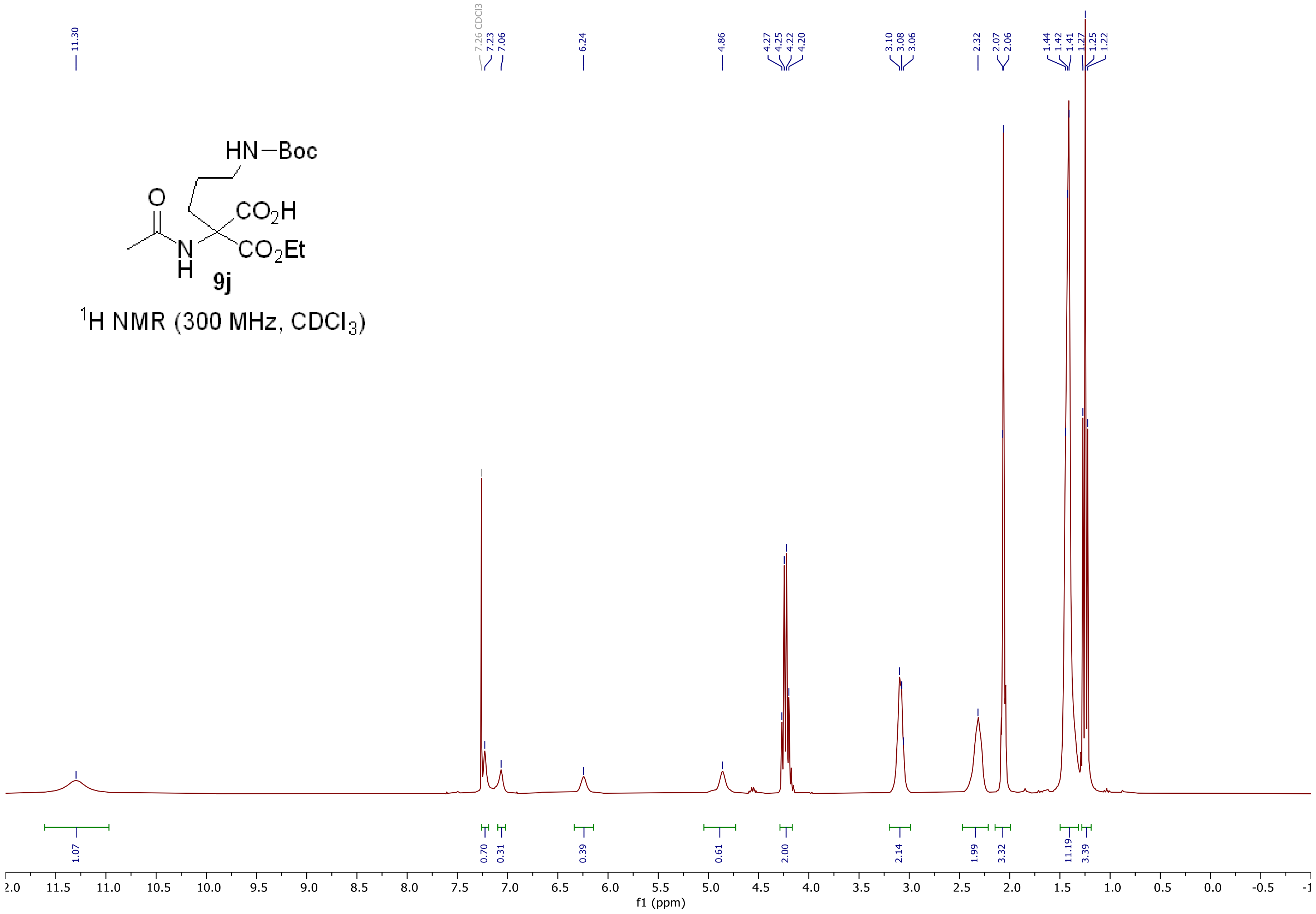

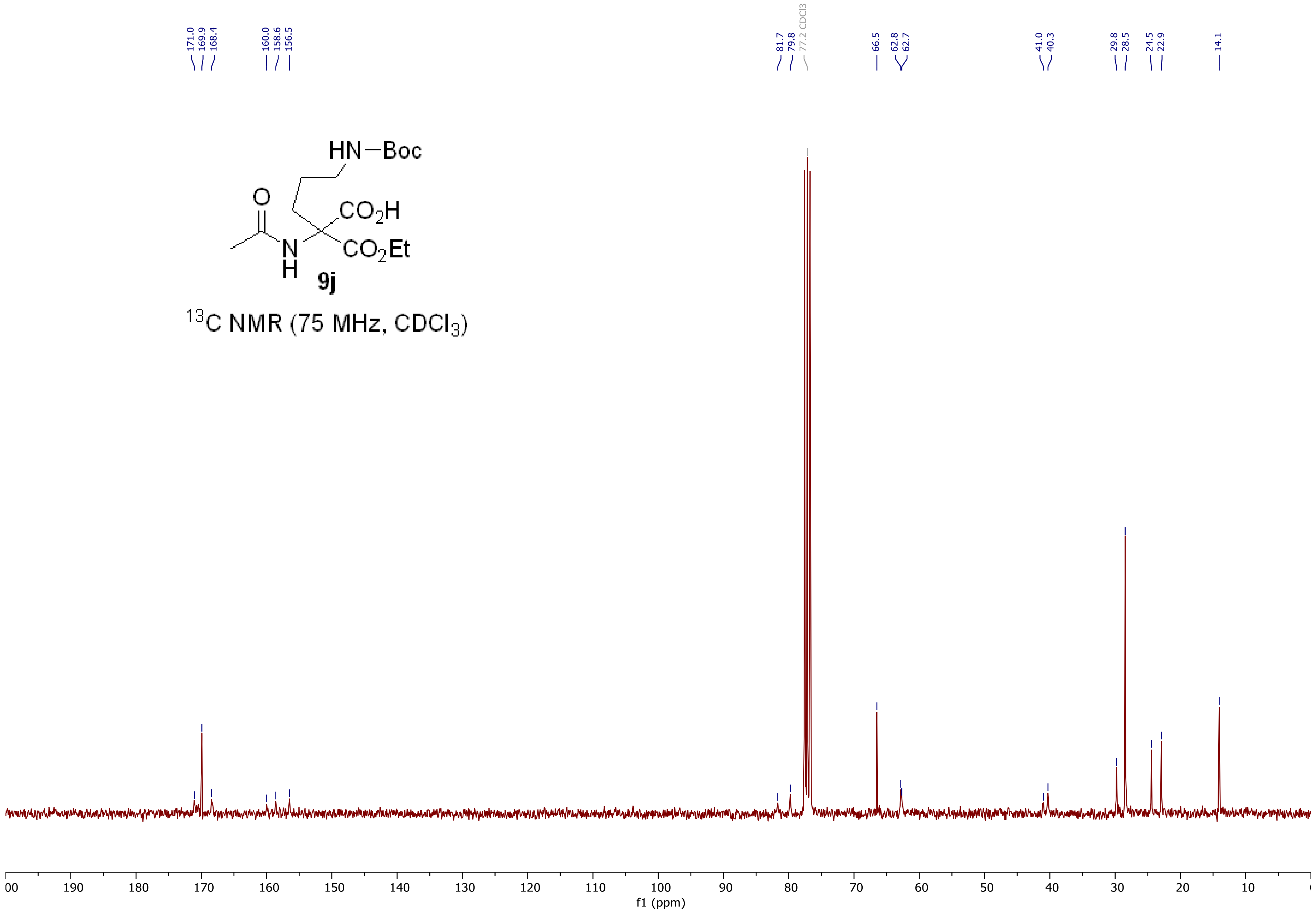

^{13}C NMR (75 MHz, $CDCl_3$)

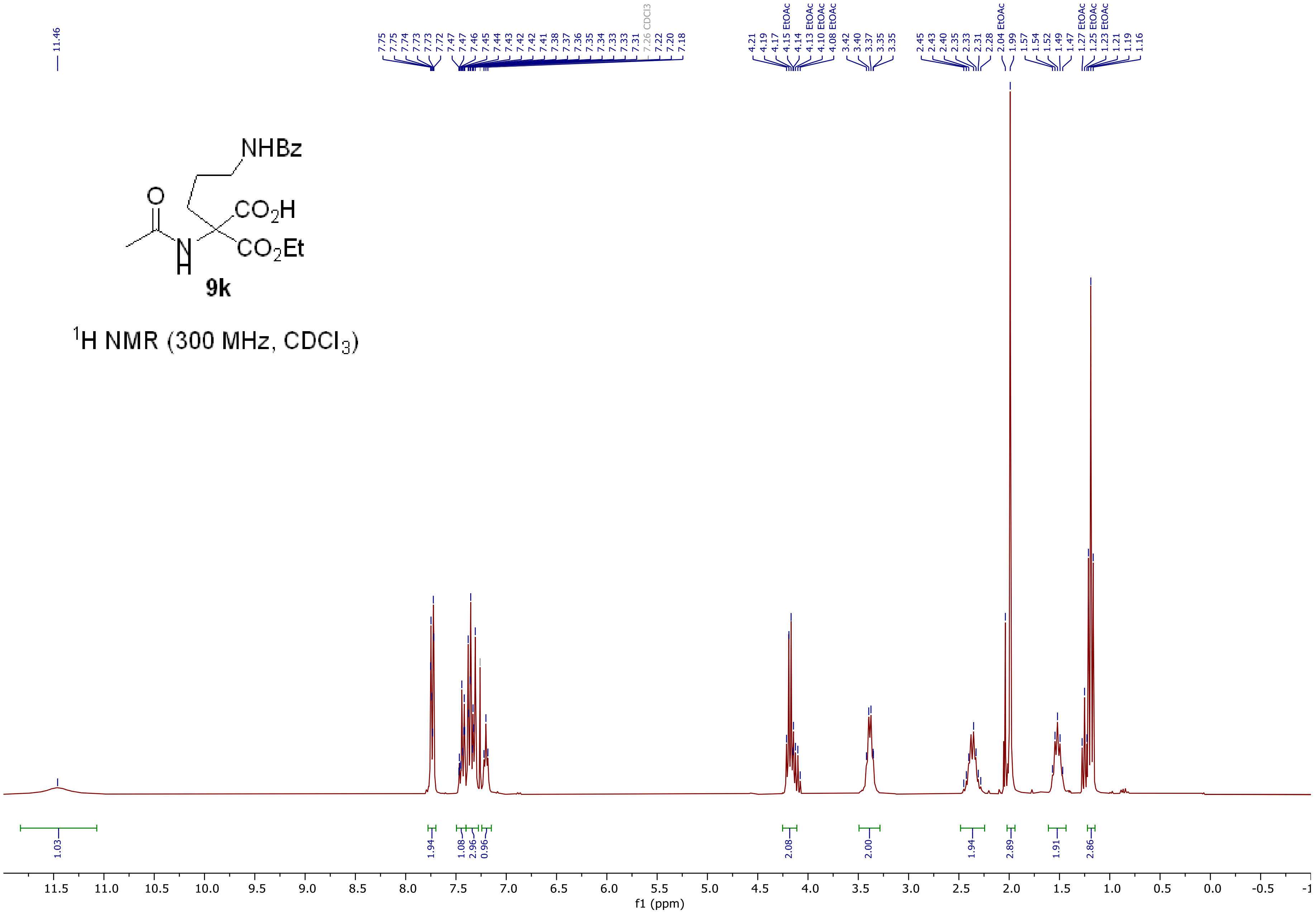
NHBz
CO_2H
CO_2Et
9k
^{1}H NMR (300 MHz, $CDCl_3$)
f1 (ppm)

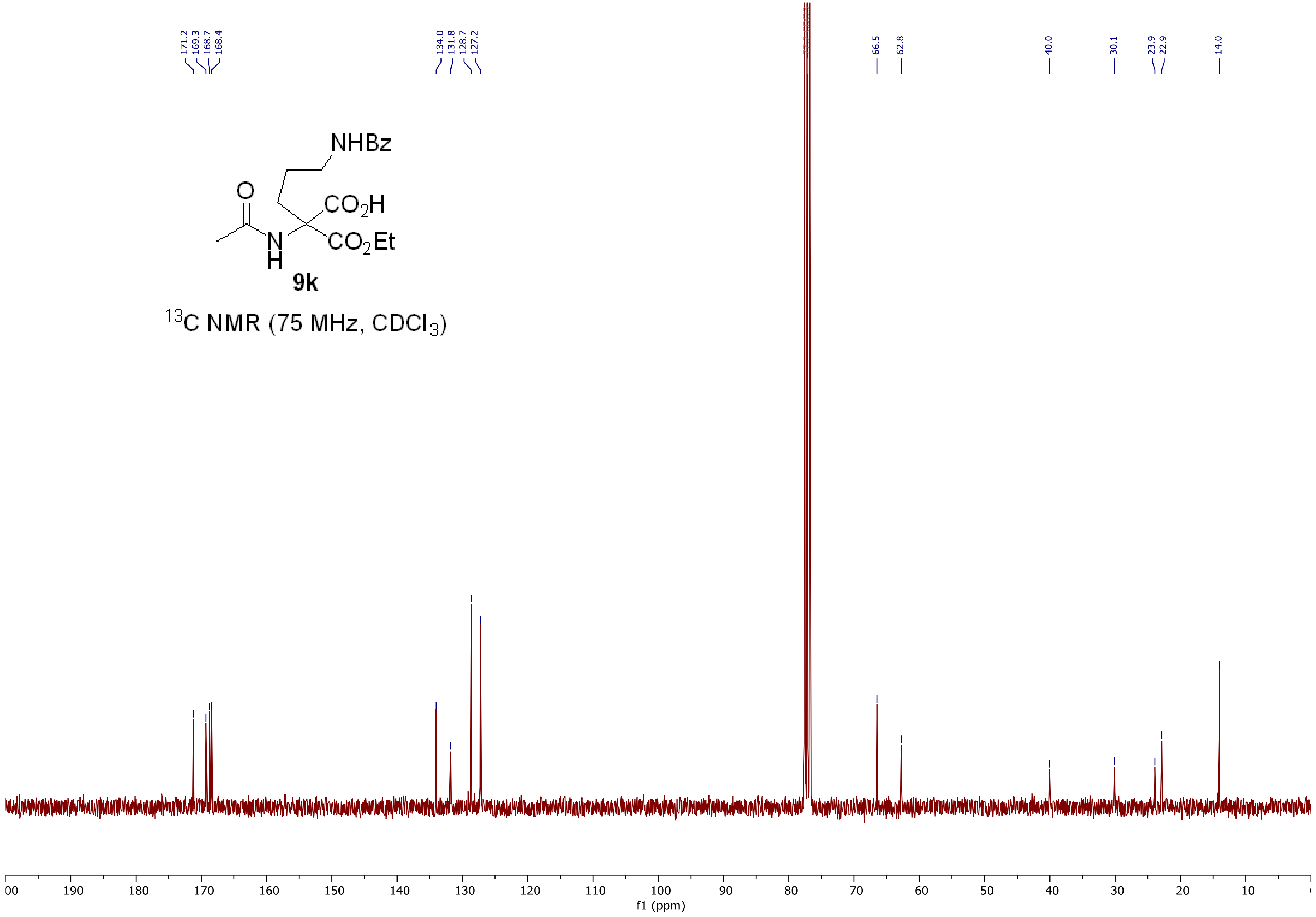
171.2
169.3
168.7
168.4
134.0
131.8
128.7
127.2
66.5
62.8
40.0
30.1
23.9
22.9
14.0
NHBz
O
CO2H
N
H
CO2Et
9k
13C NMR (75 MHz, CDCl3)
200
190
180
170
160
150
140
130
120
110
100
90
80
70
60
50
40
30
20
10
0
f1 (ppm)

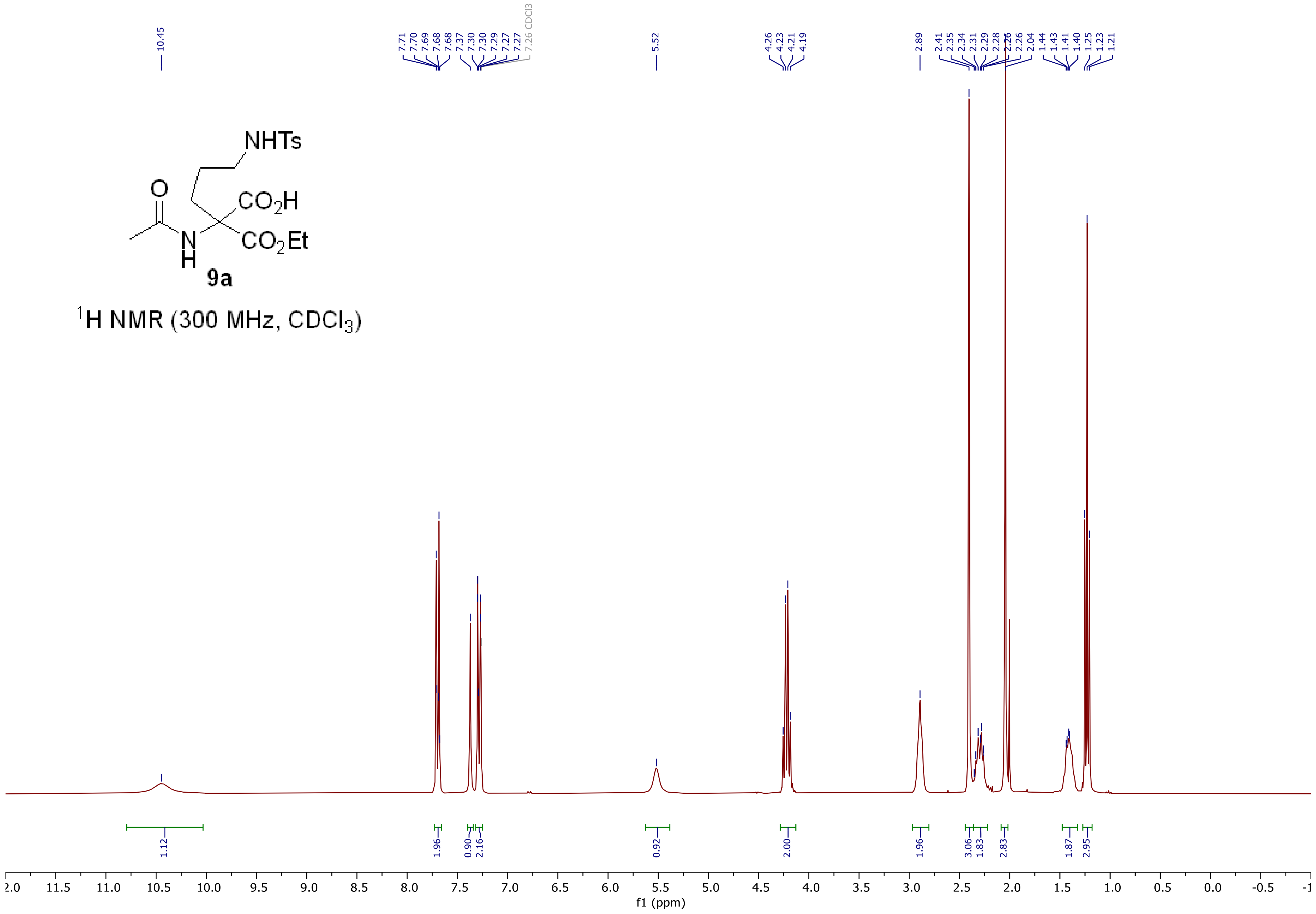
NHTs
CO_2H
CO_2Et
O
N
H
9a
^1H NMR (300 MHz, $CDCl_3$)
f1 (ppm)

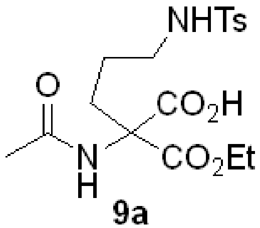

9a

^{13}C NMR (75 MHz, DMSO)

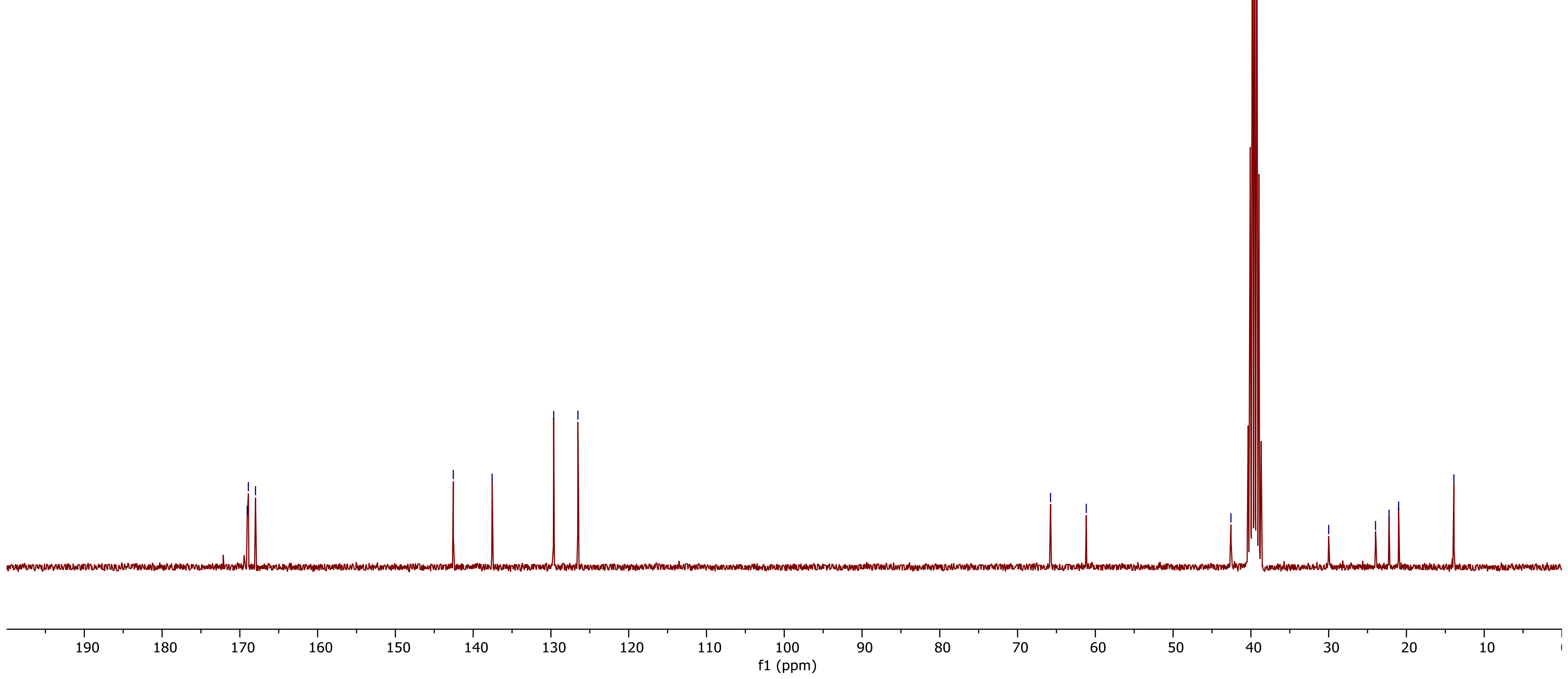

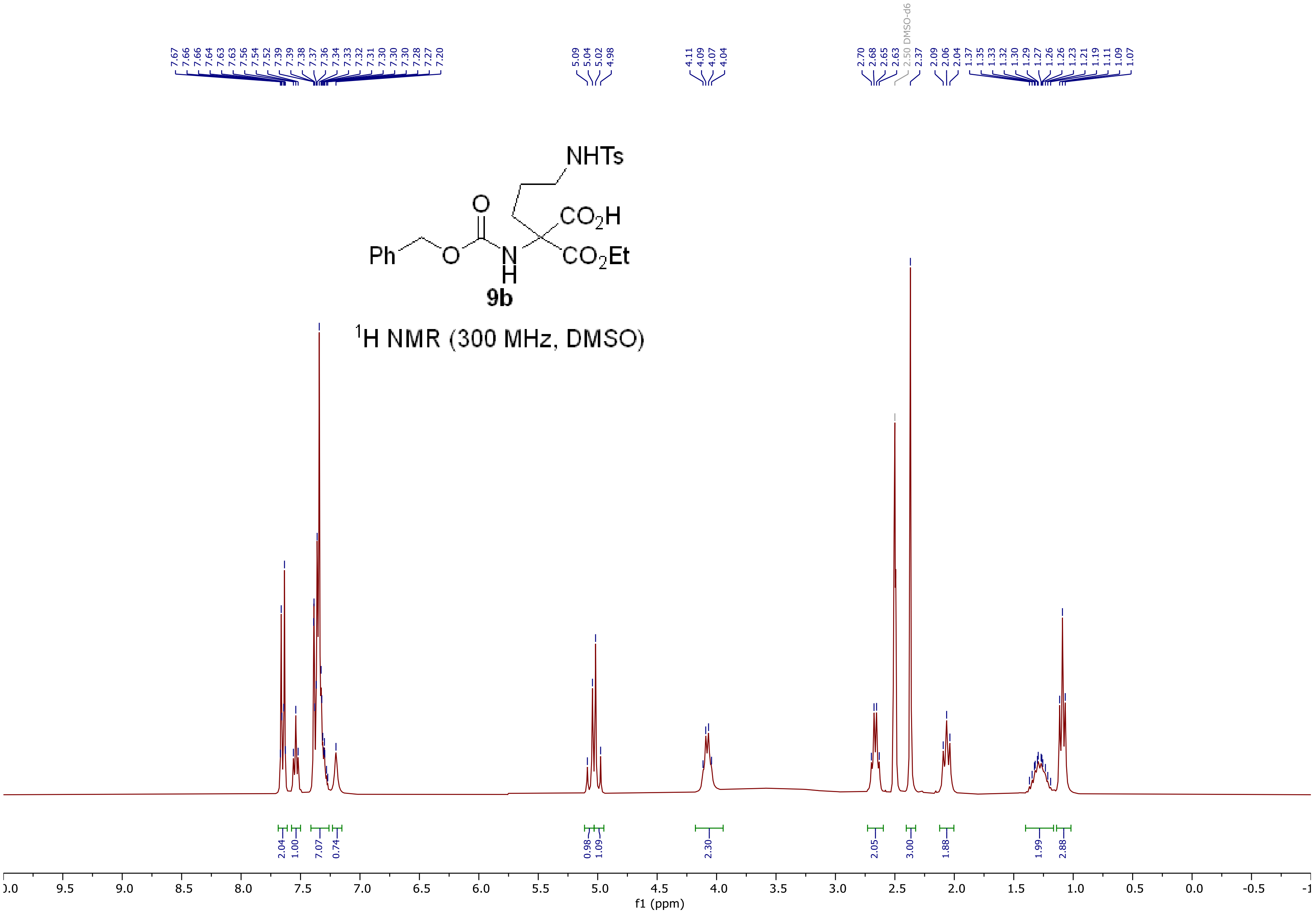
NHTs
CO_2H
CO_2Et
Ph
O
N
H
9b
^1H NMR (300 MHz, DMSO)
2.50 DMSO-d6
f1 (ppm)

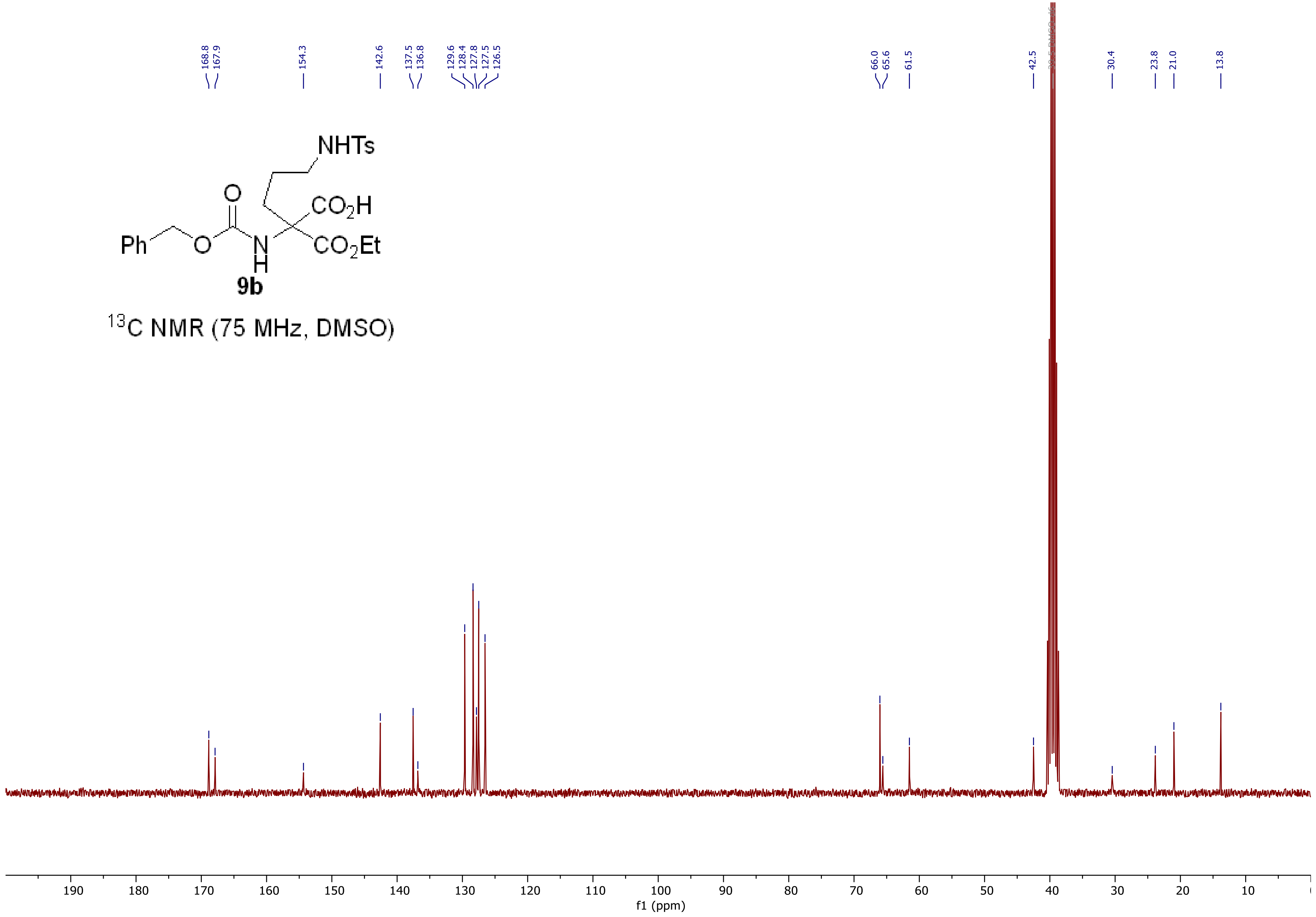
168.8
167.9
154.3
142.6
137.5
136.8
129.6
128.4
127.8
127.5
126.5
66.0
65.6
61.5
42.5
30.4
23.8
21.0
13.8
NHTs
CO2H
CO2Et
Ph
O
N
H
9b
13C NMR (75 MHz, DMSO)
190 180 170 160 150 140 130 120 110 100 90 80 70 60 50 40 30 20 10
f1 (ppm)

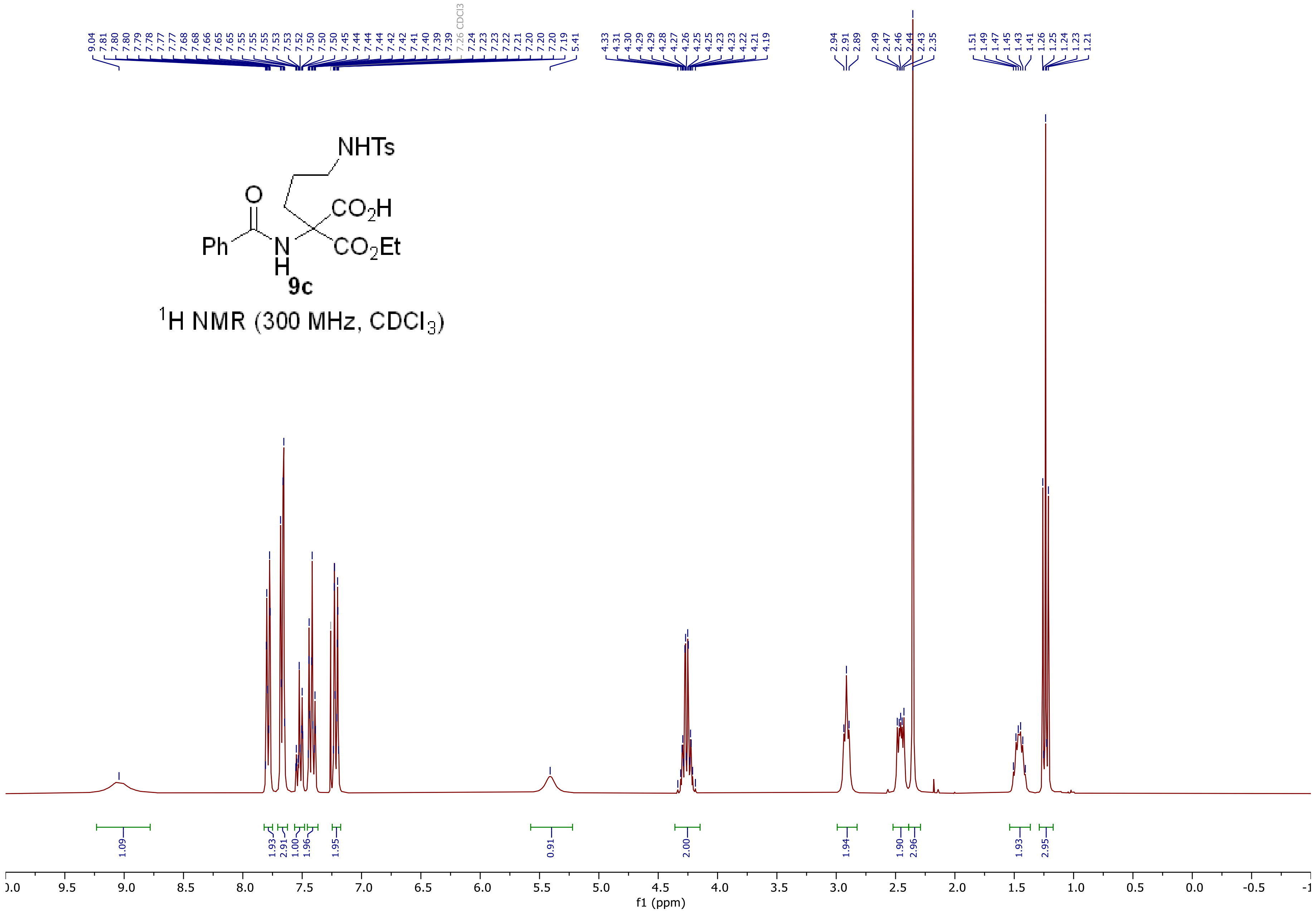
NHTs
O
CO2H
Ph
N
H
CO2Et
9c
1H NMR (300 MHz, CDCl3)
f1 (ppm)

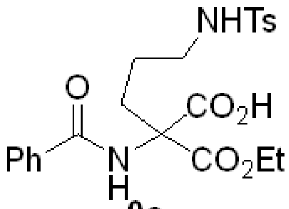

9c

^{13}C NMR (75 MHz, $CDCl_3$)

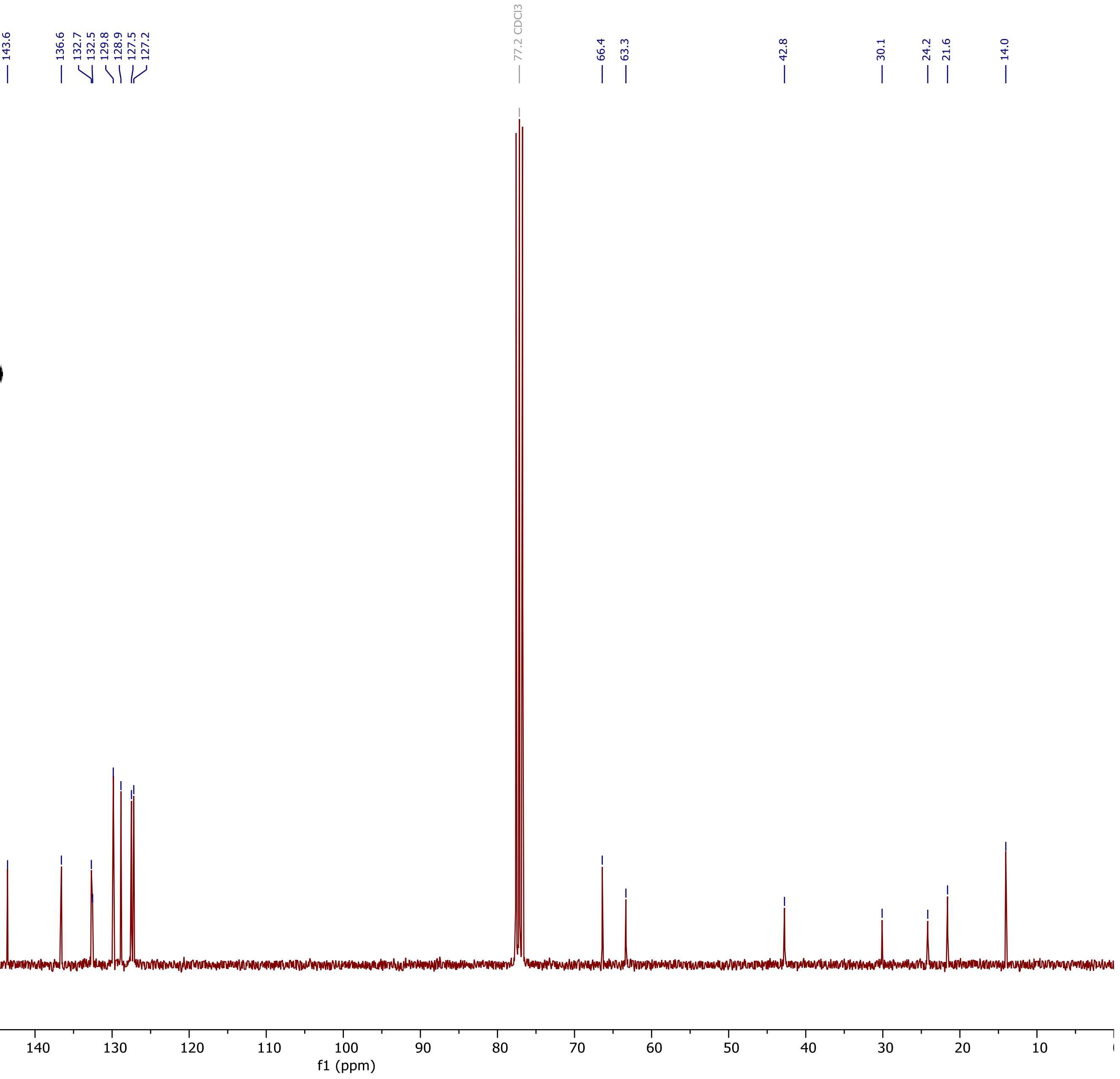

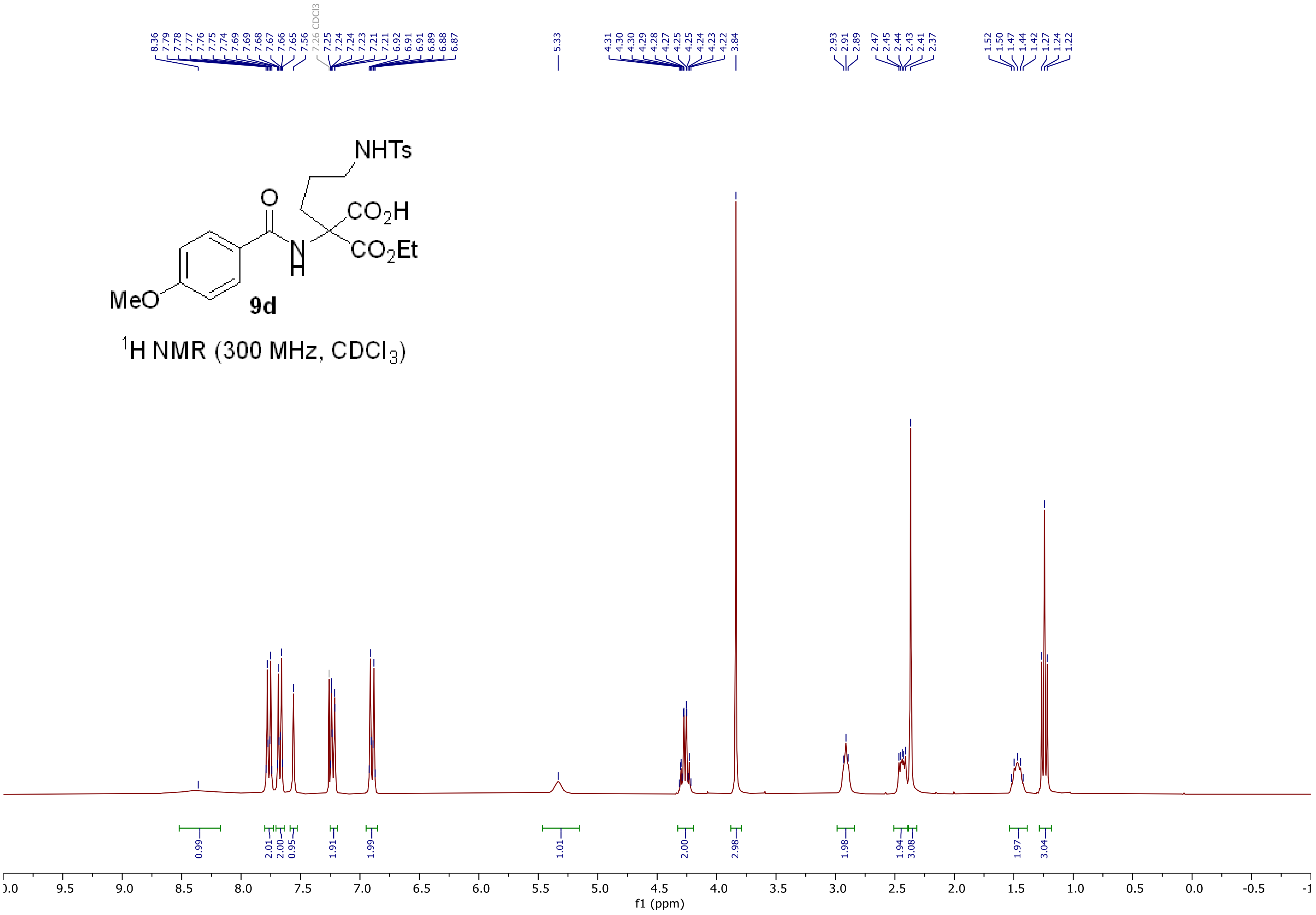
NHTs
CO_2H
CO_2Et
MeO
9d
^1H NMR (300 MHz, $CDCl_3$)
f1 (ppm)

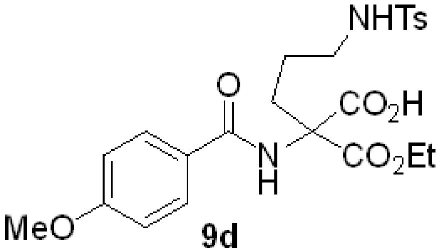

9d

^{13}C NMR (75 MHz, $CDCl_3$)

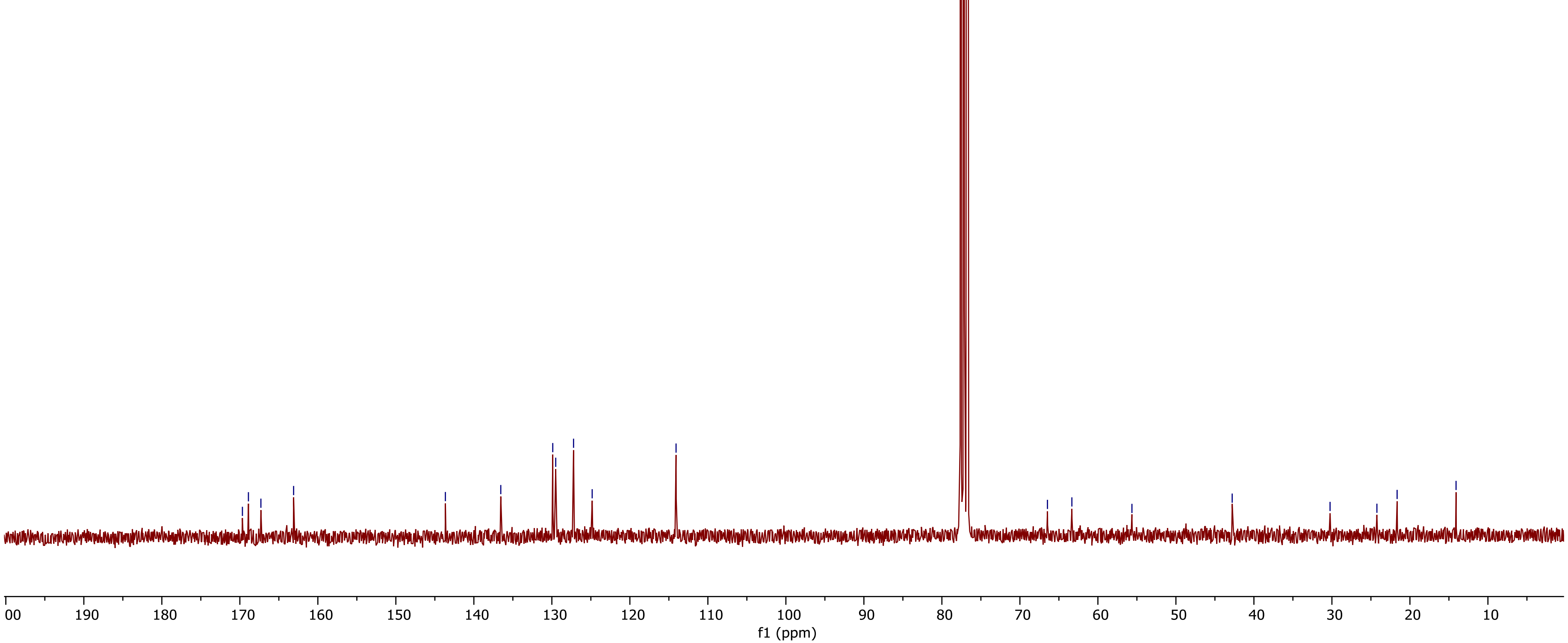

9e

^{1}H NMR (300 MHz, $CDCl_3$)

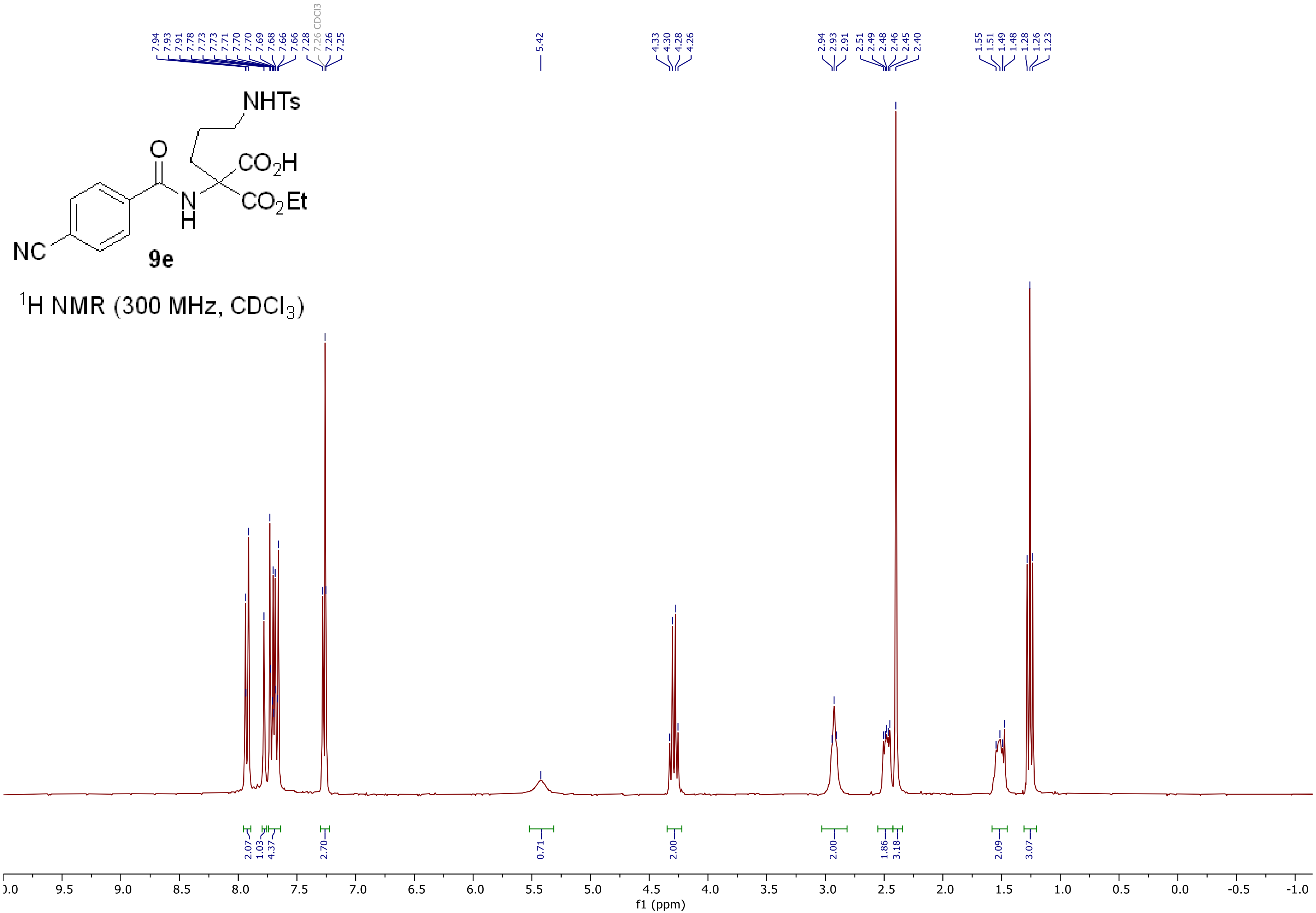

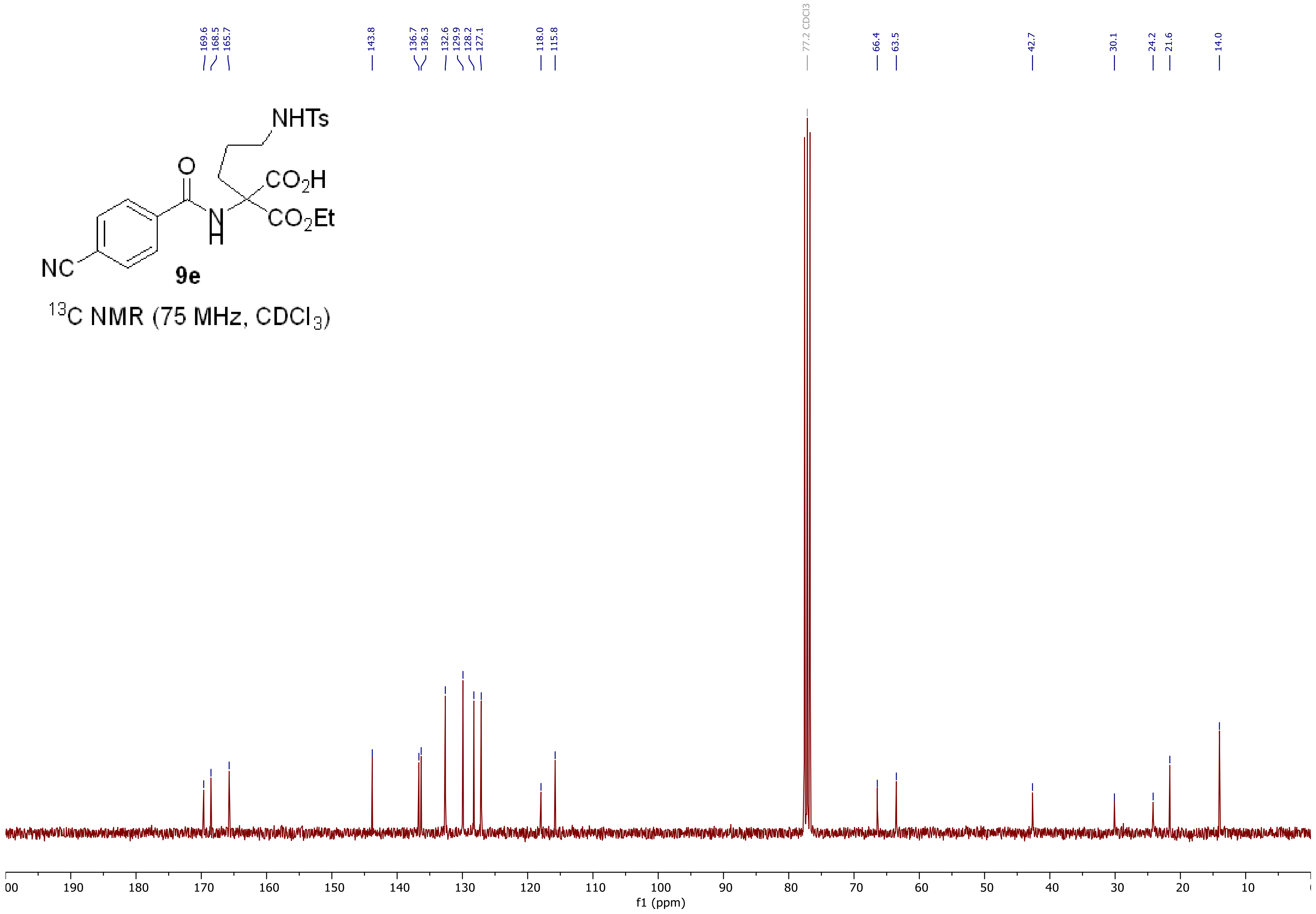
169.6
168.5
165.7
143.8
136.7
136.3
132.6
129.9
128.2
127.1
118.0
115.8
77.2 CDCl3
66.4
63.5
42.7
30.1
24.2
21.6
14.0
NHTs
O
CO2H
CO2Et
N
H
NC
9e
13C NMR (75 MHz, CDCl3)
f1 (ppm)

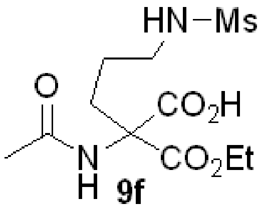

9f

^{1}H NMR (300 MHz, MeOD)

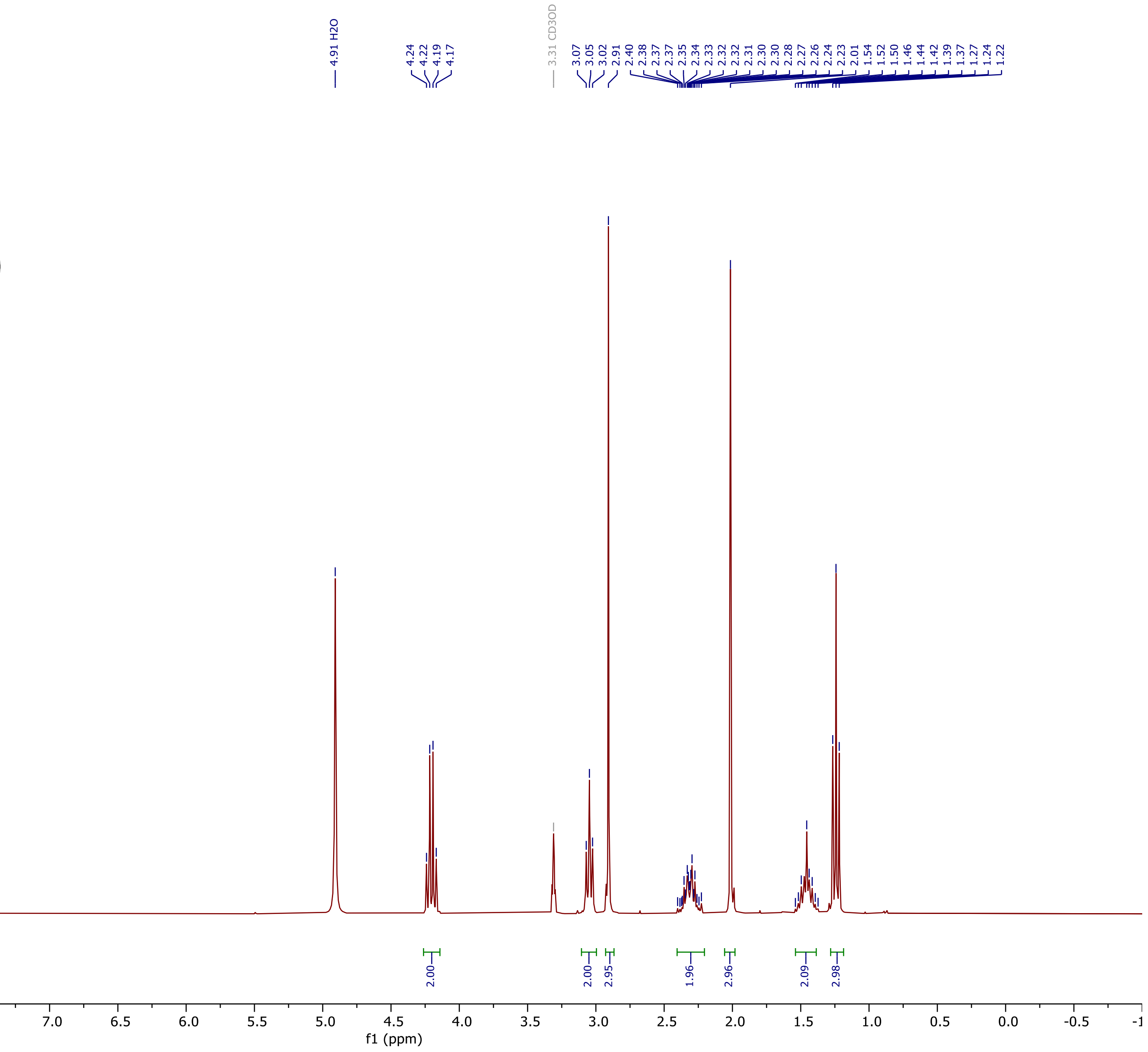

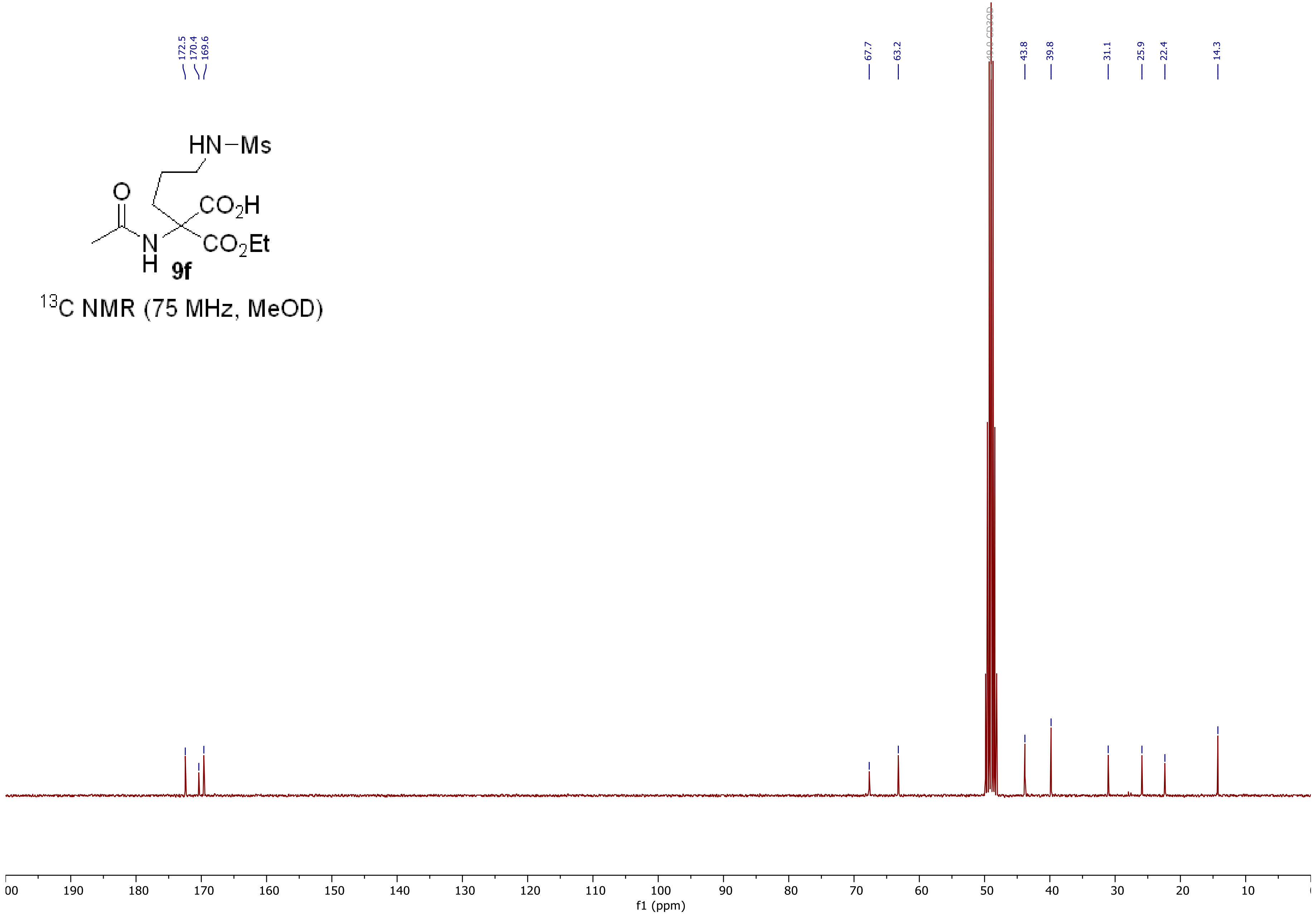
HN−Ms
O
CO_2H
N
H
CO_2Et
9f
^{13}C NMR (75 MHz, MeOD)
172.5
170.4
169.6
67.7
63.2
49.0 CD3OD
43.8
39.8
31.1
25.9
22.4
14.3
f1 (ppm)

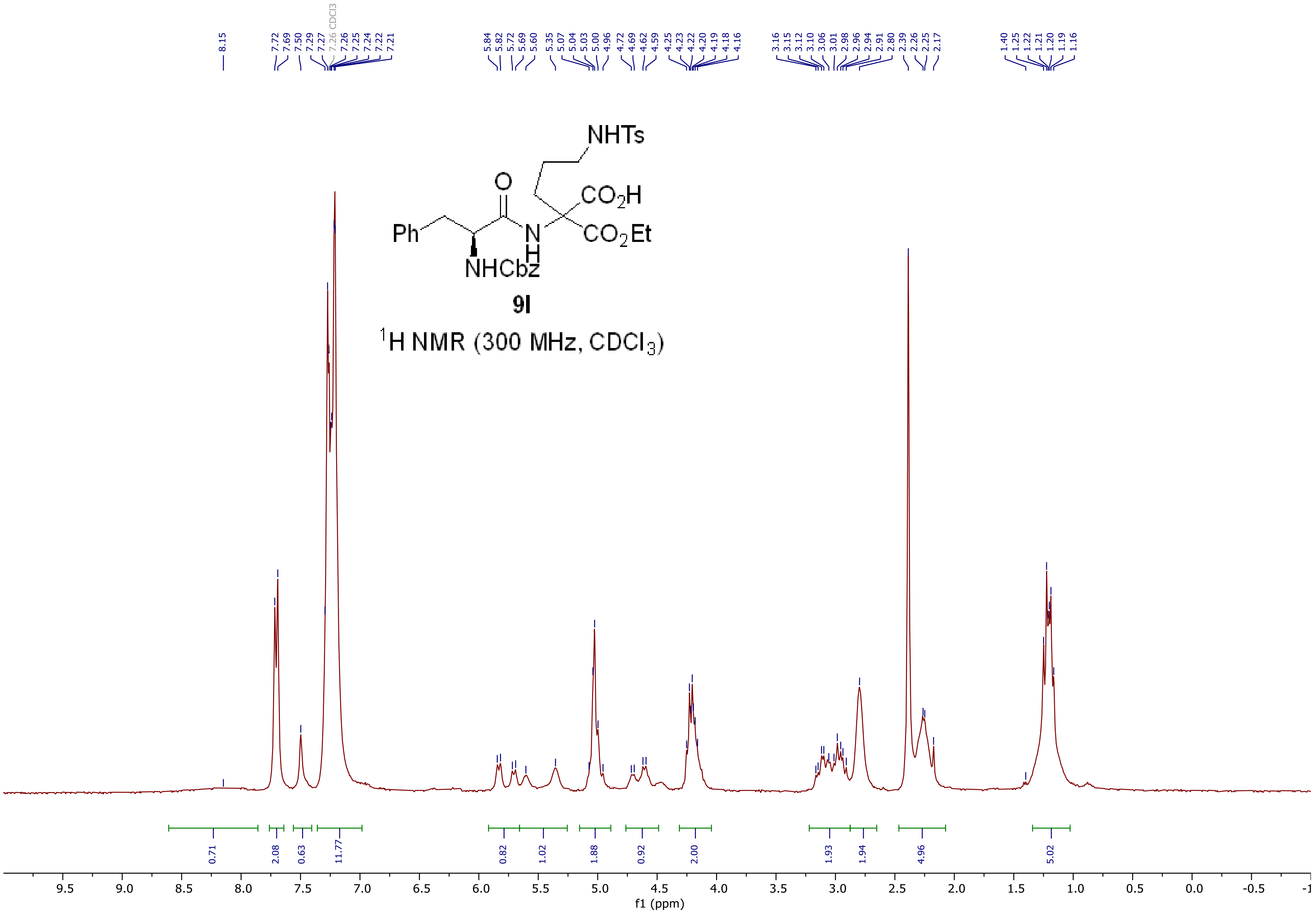

NHTs
O
CO2H
Ph
N
H
CO2Et
NHCbz
9l
1H NMR (300 MHz, CDCl3)
f1 (ppm)

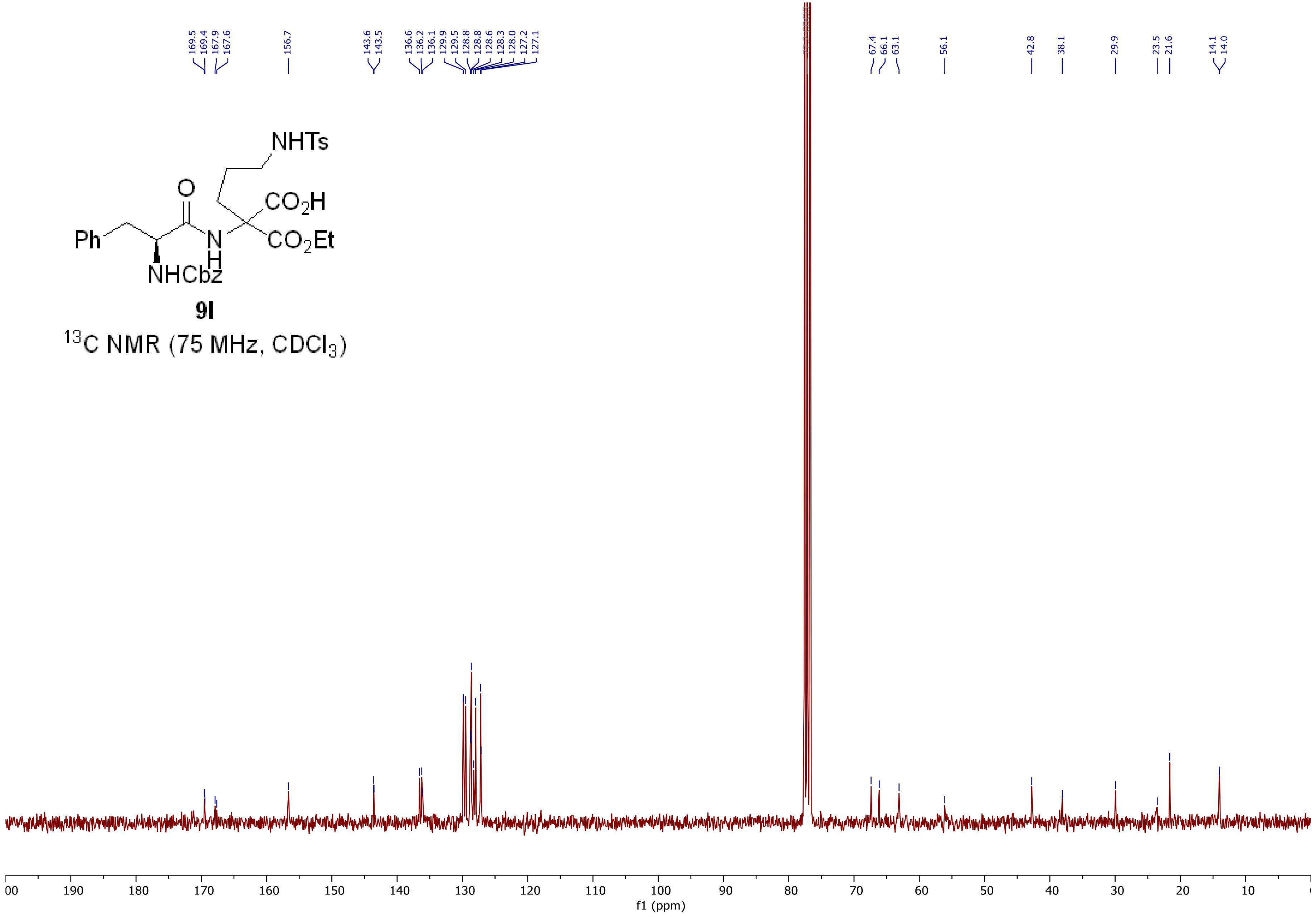

9l

^{13}C NMR (75 MHz, $CDCl_3$)

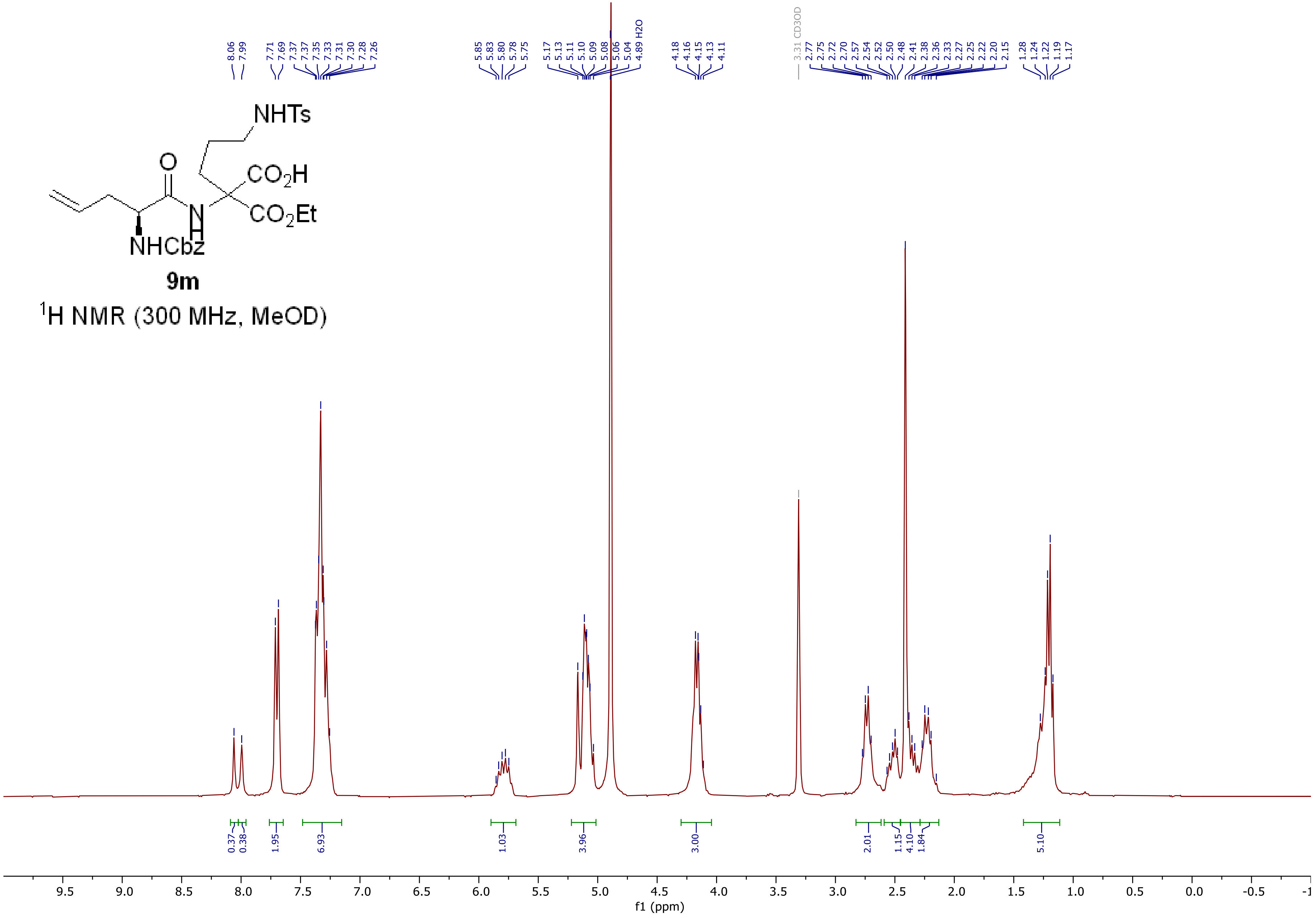

9m

^{1}H NMR (300 MHz, MeOD)

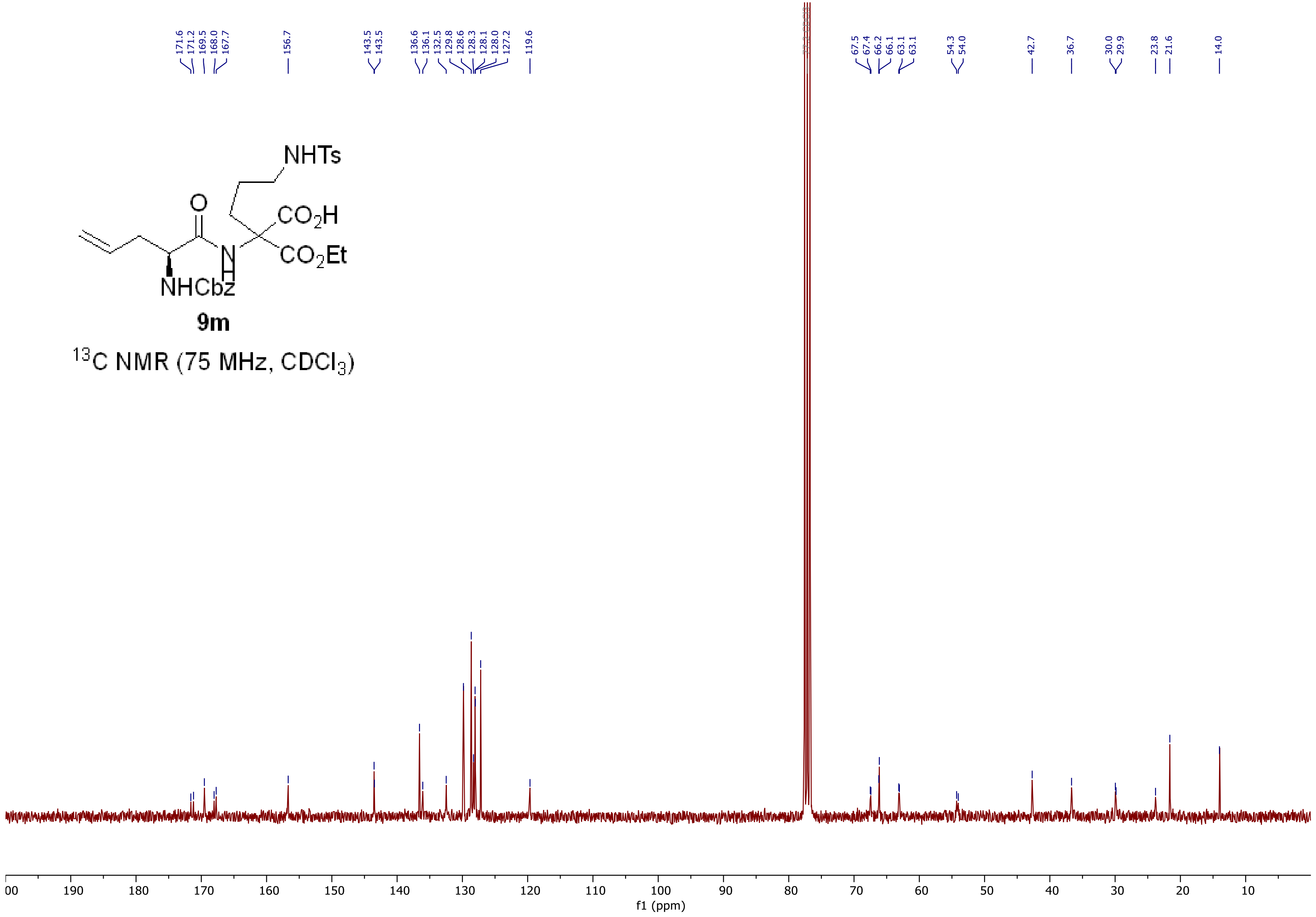

9m

^{13}C NMR (75 MHz, $CDCl_3$)

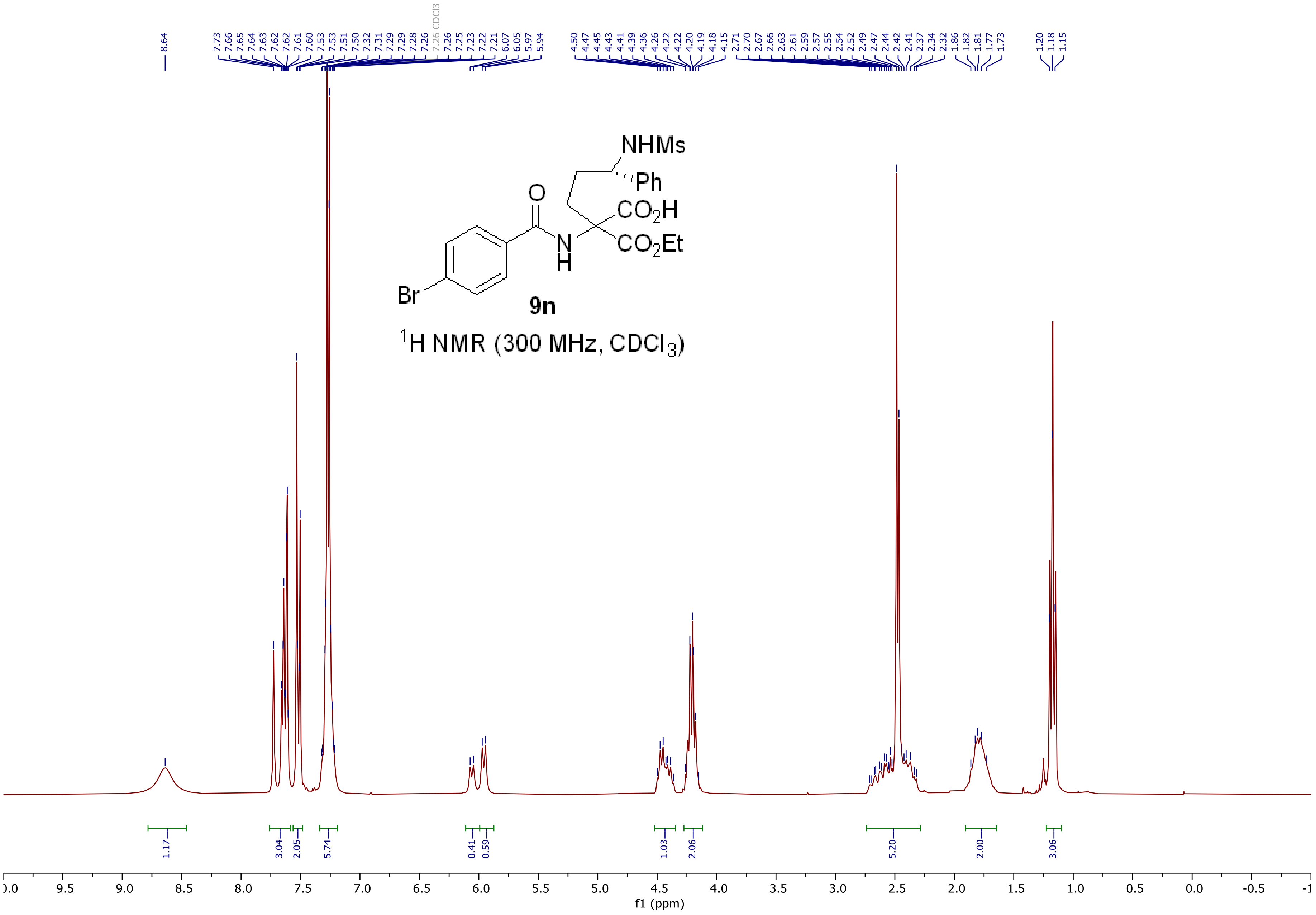
NHMs
Ph
CO_2H
CO_2Et
O
N
H
Br
9n
^1H NMR (300 MHz, $CDCl_3$)
f1 (ppm)

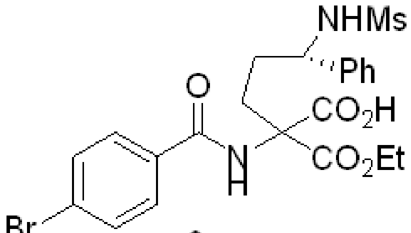

9n

^{13}C NMR (75 MHz, $CDCl_3$)

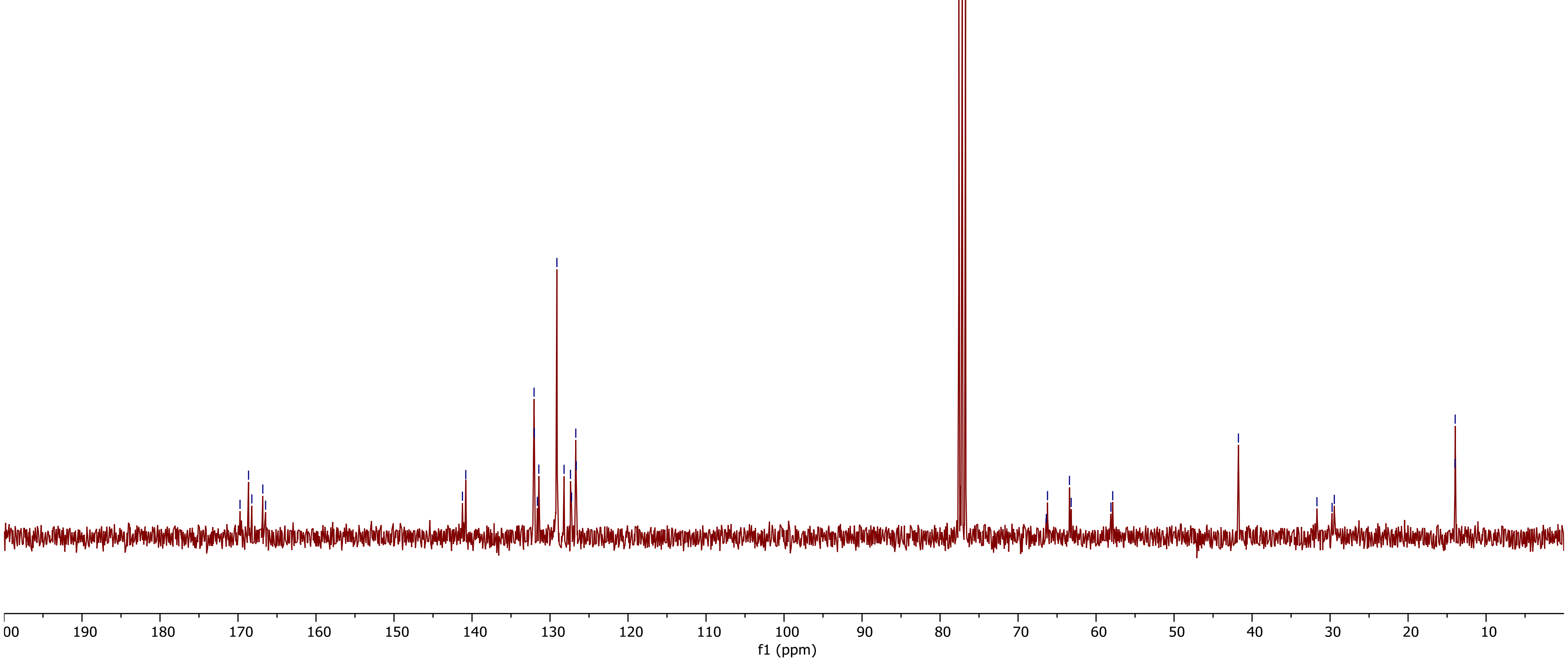

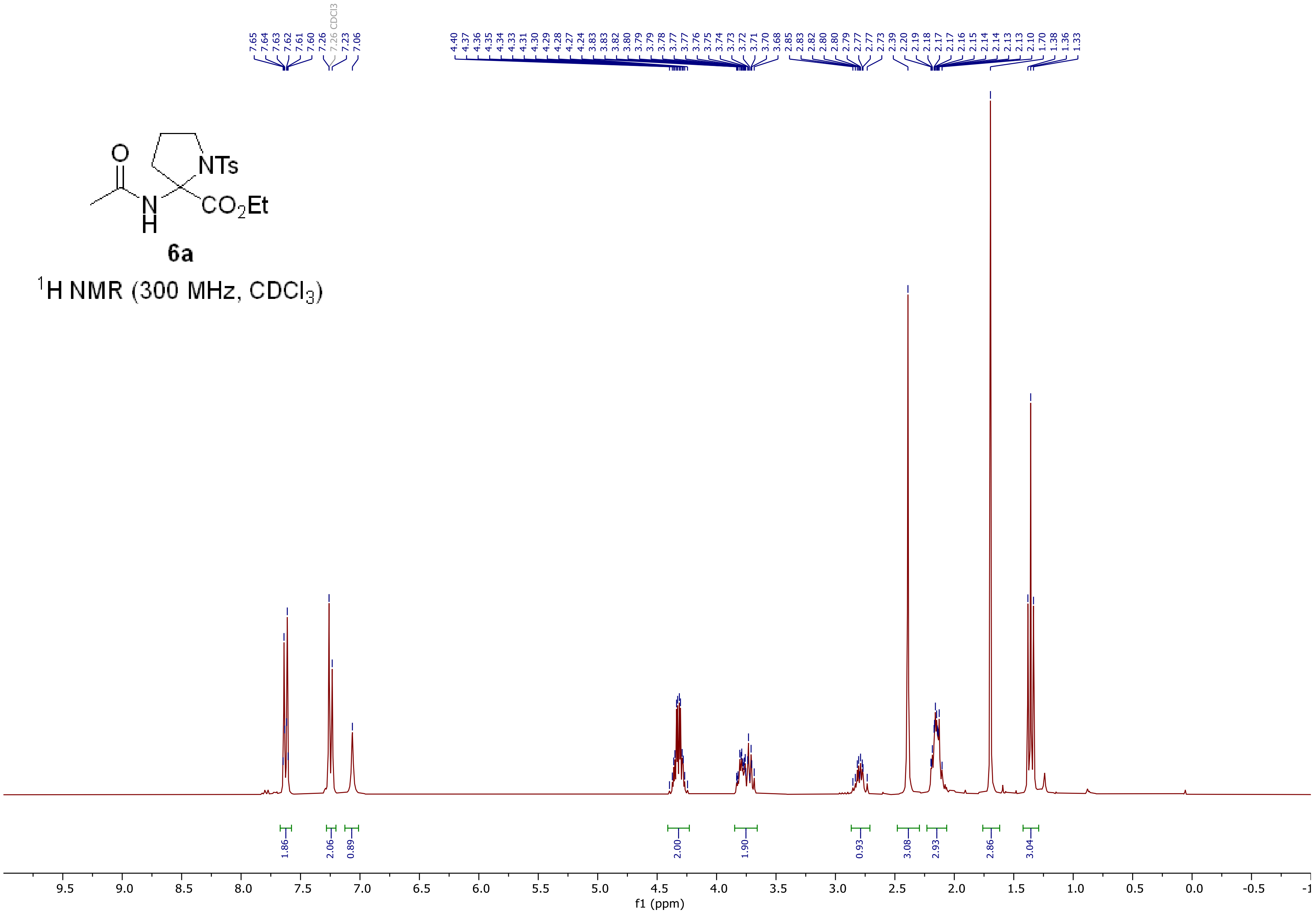
NTs
CO2Et
N
H
O
6a
1H NMR (300 MHz, CDCl3)
f1 (ppm)

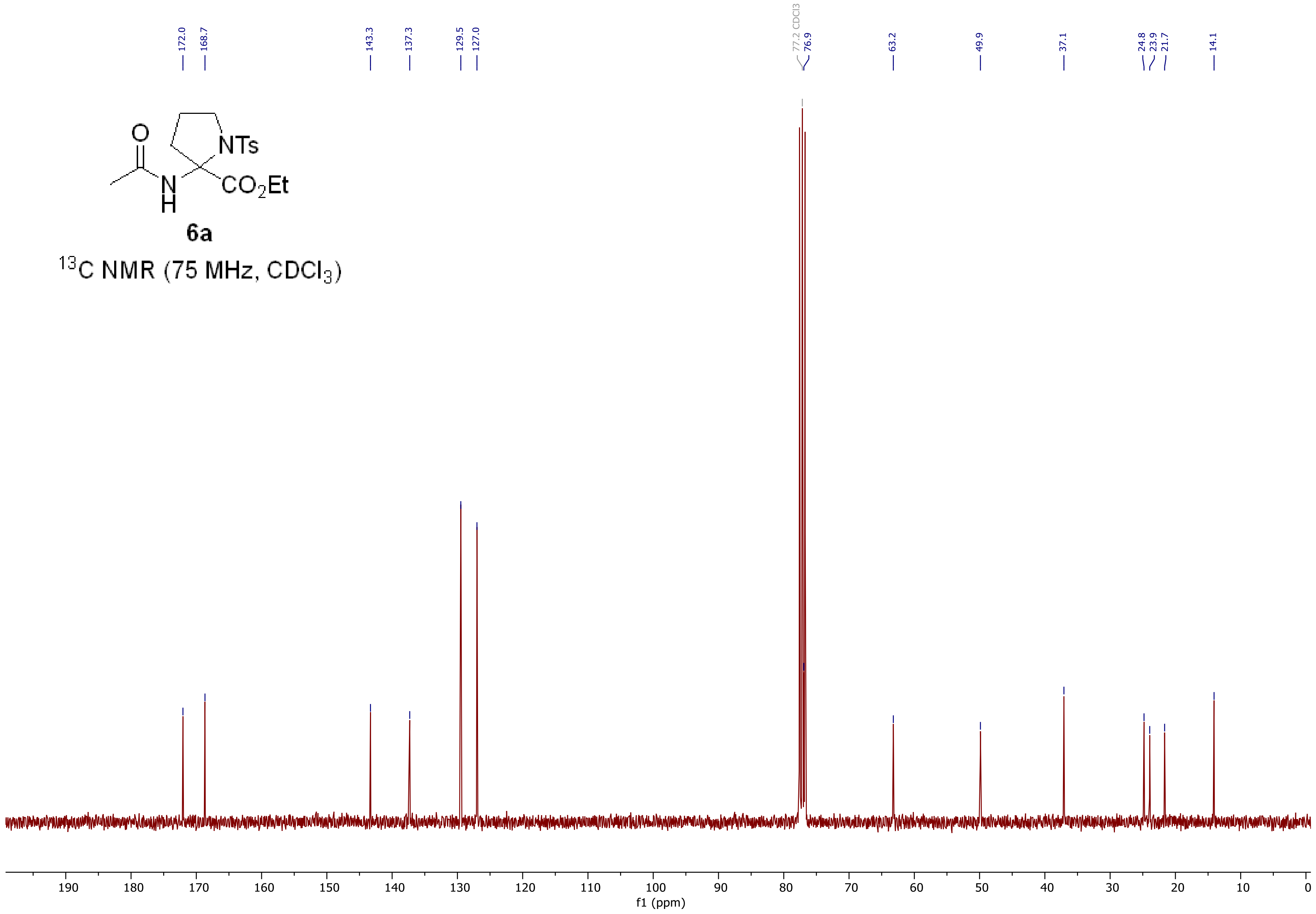

172.0
168.7
143.3
137.3
129.5
127.0
77.2 CDCl3
76.9
63.2
49.9
37.1
24.8
23.9
21.7
14.1
O
NTs
N
H
CO2Et
6a
13C NMR (75 MHz, CDCl3)
190
180
170
160
150
140
130
120
110
100
90
80
70
60
50
40
30
20
10
0
f1 (ppm)

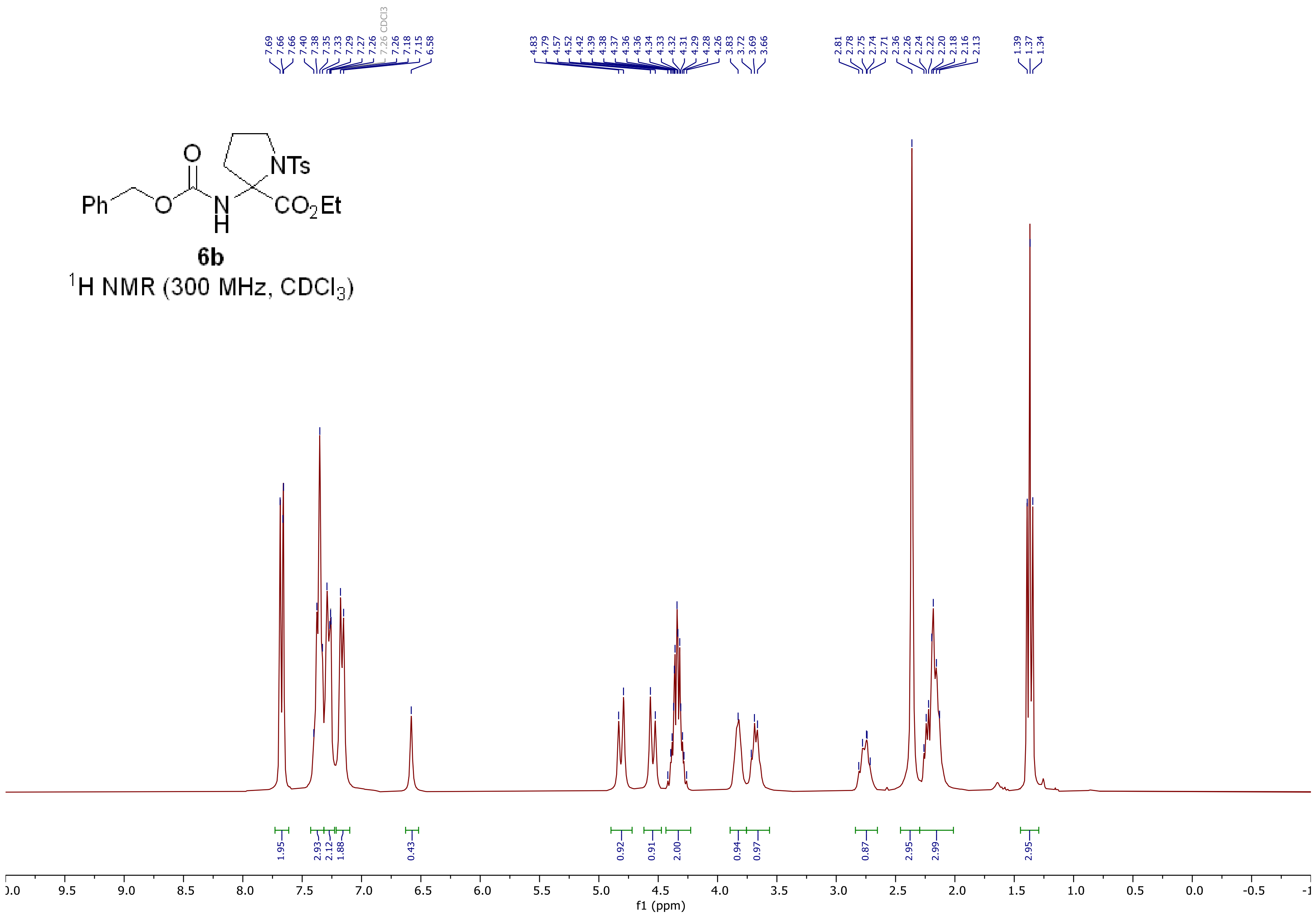
Ph O O N H NTs CO2Et
6b
1H NMR (300 MHz, CDCl3)
f1 (ppm)

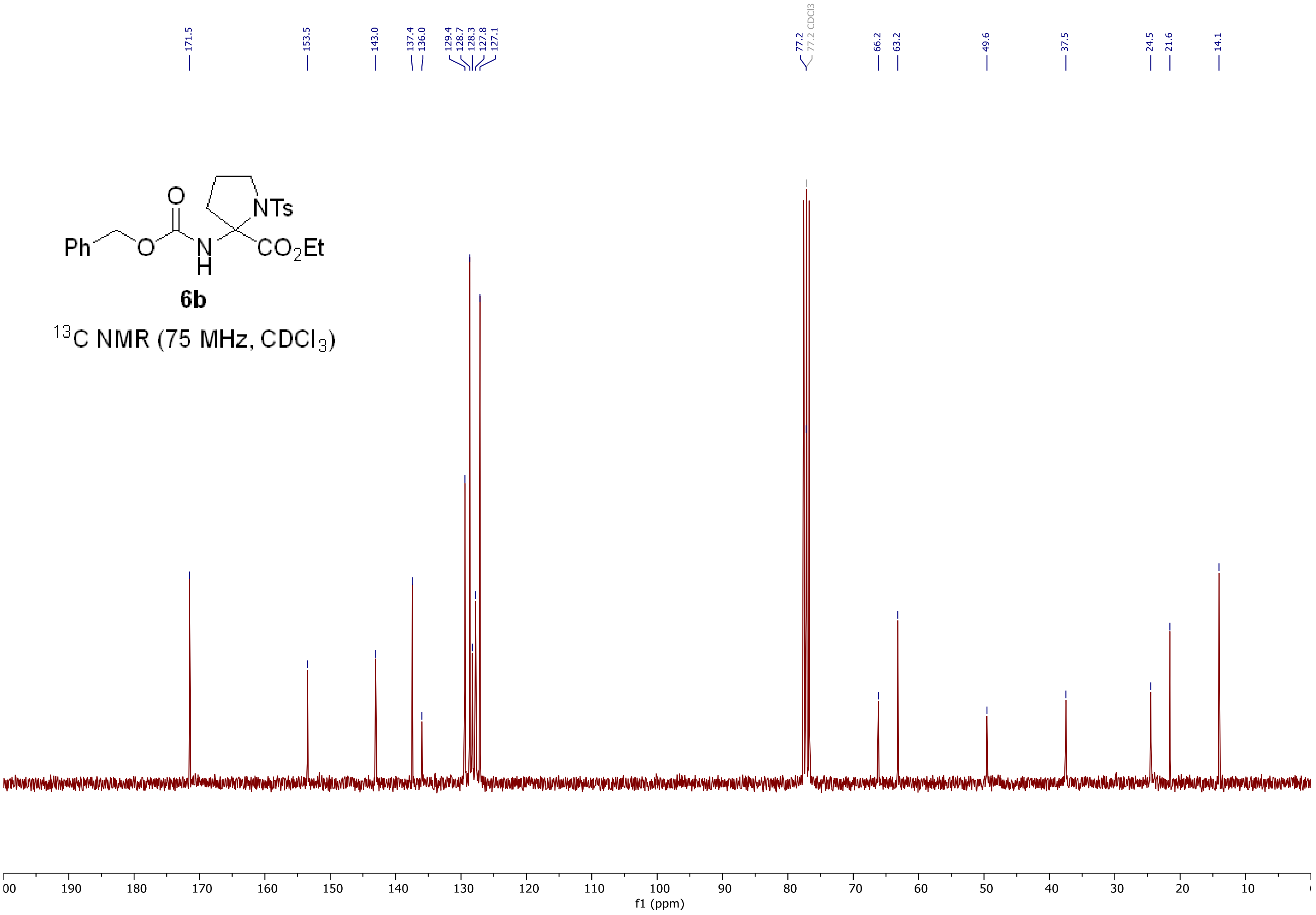
Ph
O
O
N
H
NTs
CO2Et
6b
13C NMR (75 MHz, CDCl3)
171.5
153.5
143.0
137.4
136.0
129.4
128.7
128.3
127.8
127.1
77.2
77.2 CDCl3
66.2
63.2
49.6
37.5
24.5
21.6
14.1
f1 (ppm)

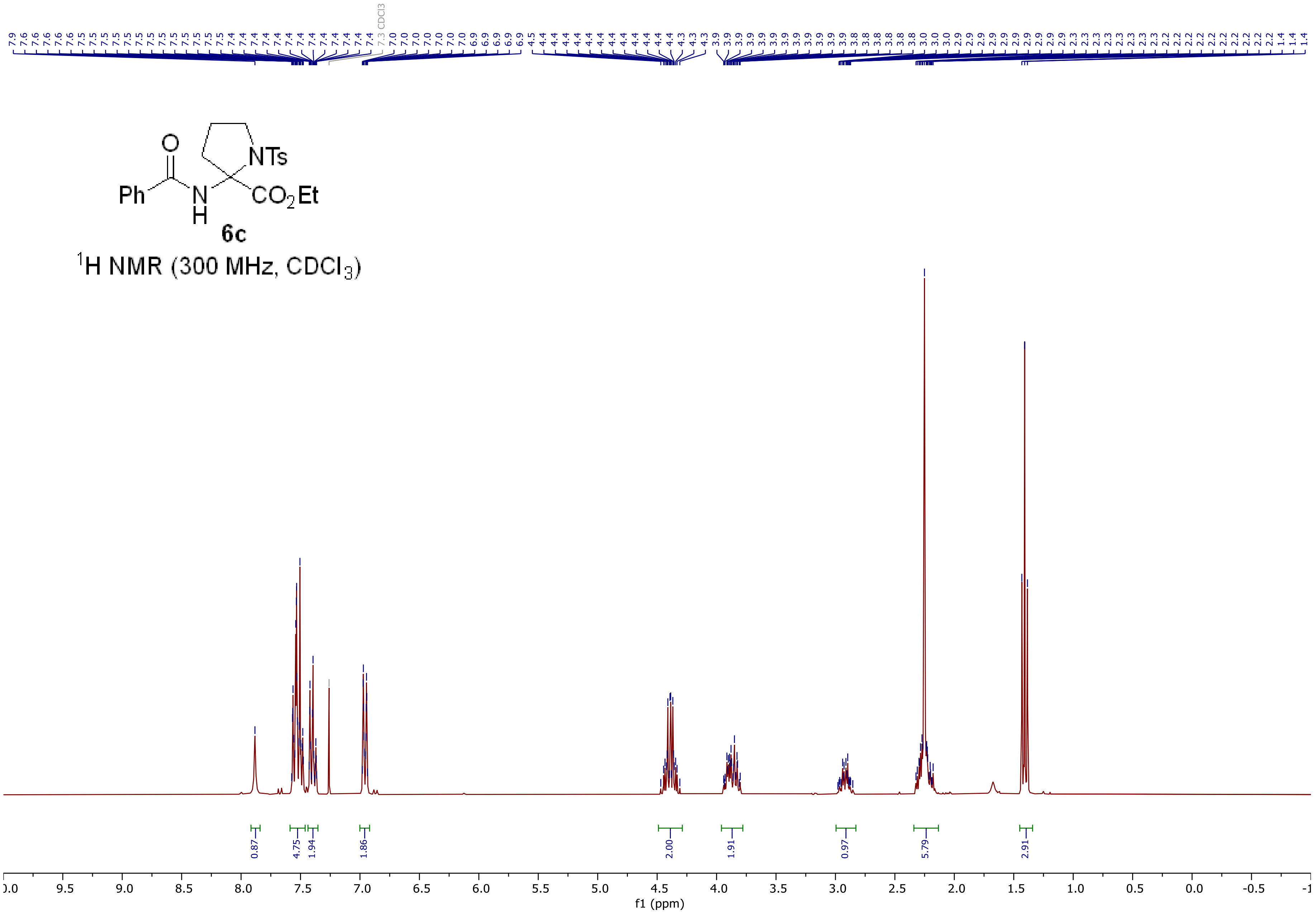
6c
¹H NMR (300 MHz, CDCl3)
0.87
4.75
1.94
1.86
2.00
1.91
0.97
5.79
2.91
f1 (ppm)

6c

^{13}C NMR (75 MHz, $CDCl_3$)

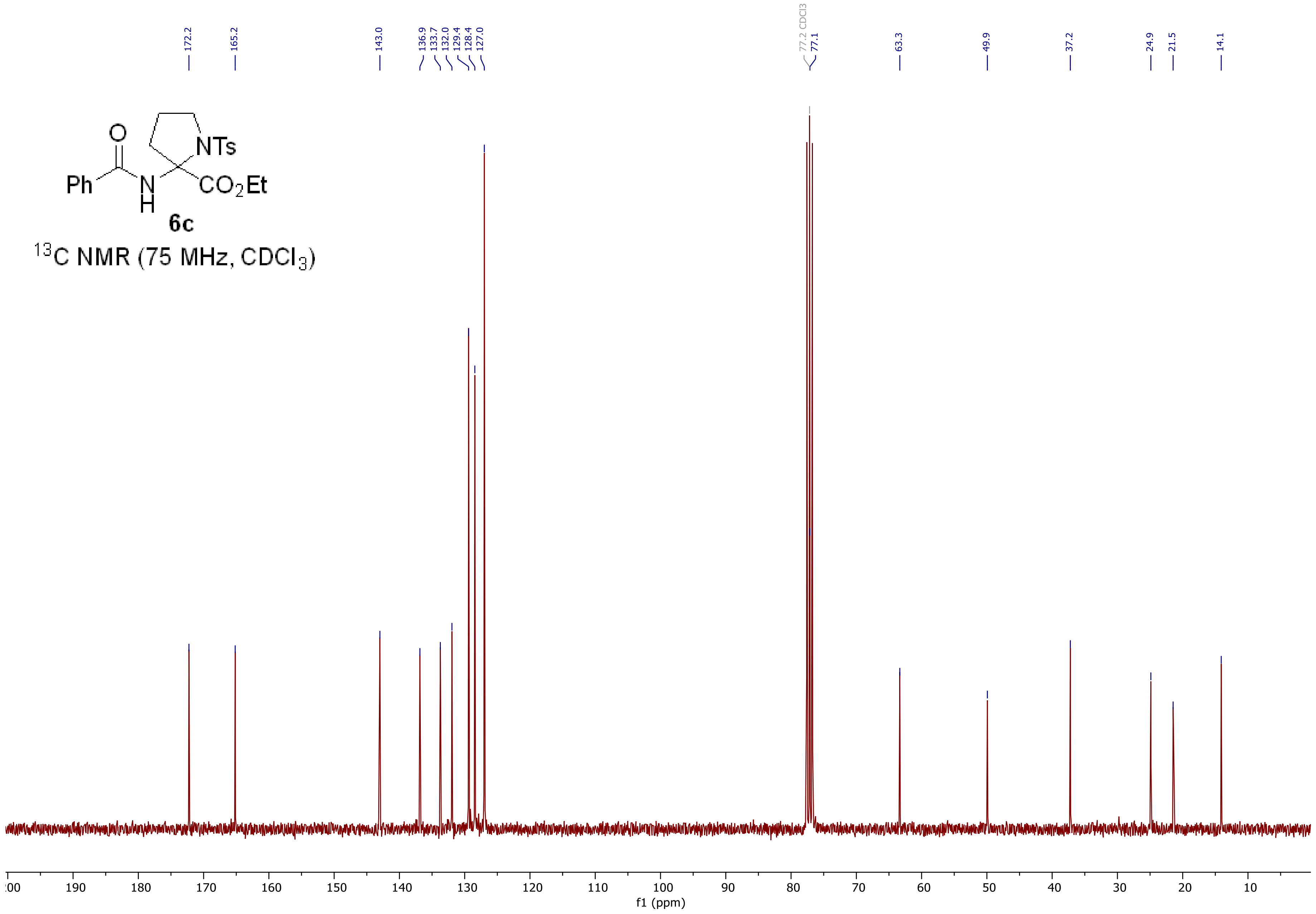

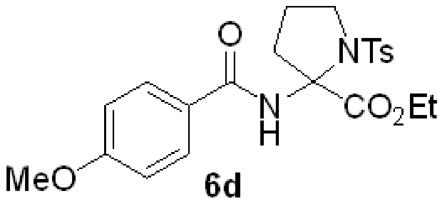

6d

^{1}H NMR (300 MHz, $CDCl_3$)

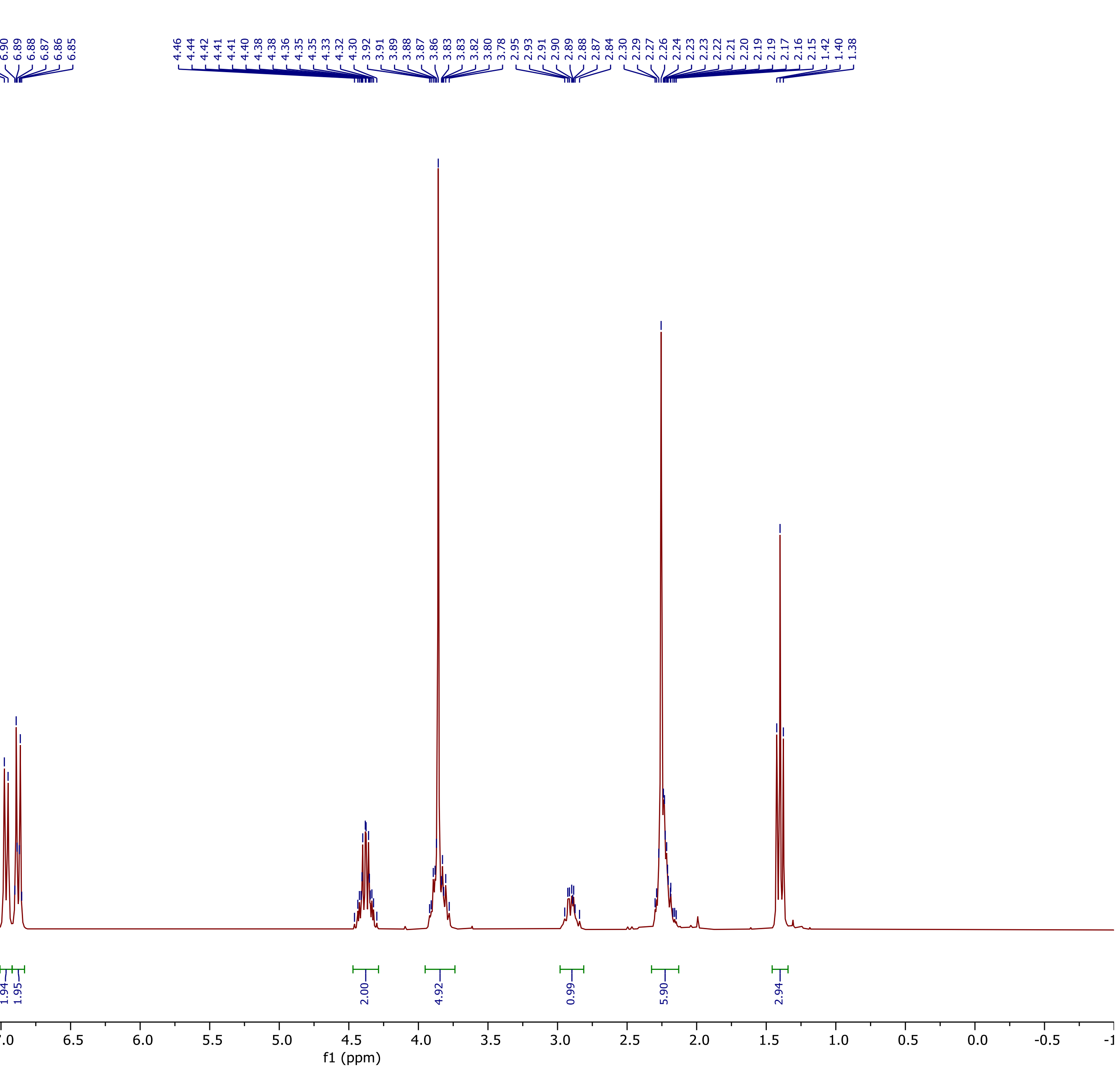

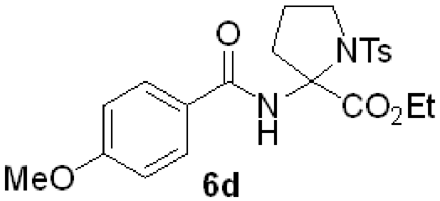

6d

^{13}C NMR (75 MHz, $CDCl_3$)

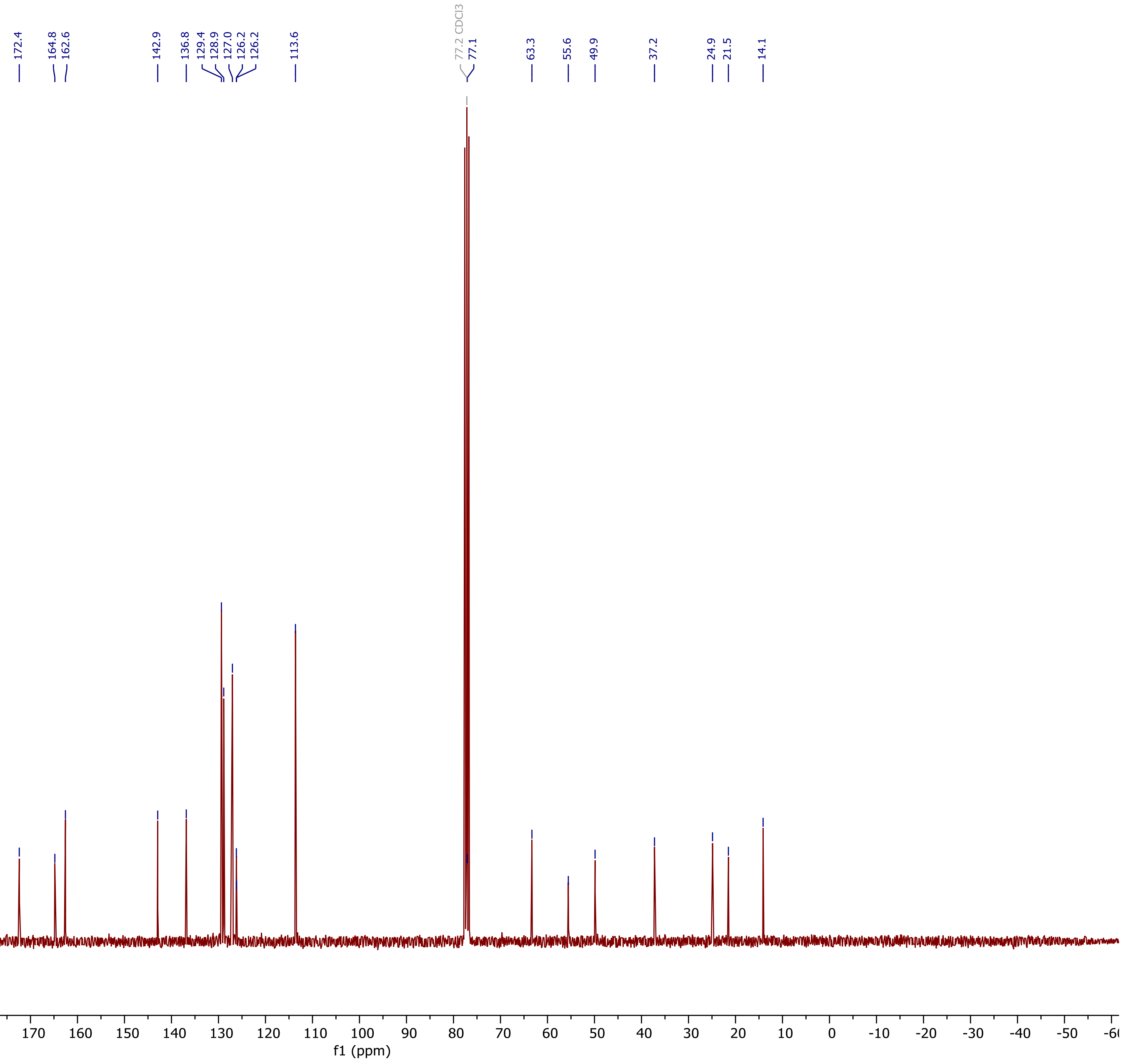

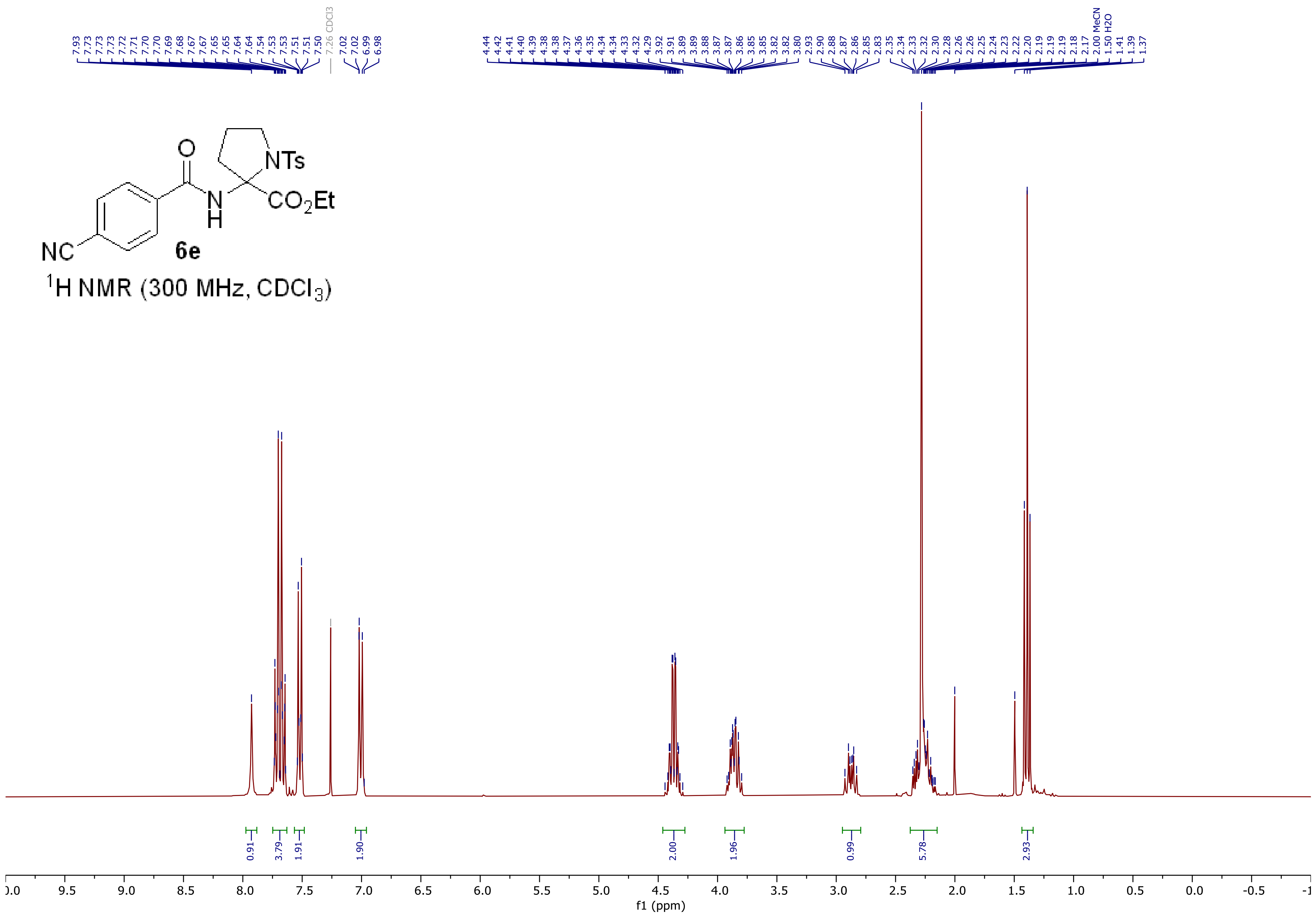
NTs
CO_2Et
NC
6e
^{1}H NMR (300 MHz, $CDCl_3$)
f1 (ppm)
7.26 CDCl3
2.00 MeCN
1.50 H2O

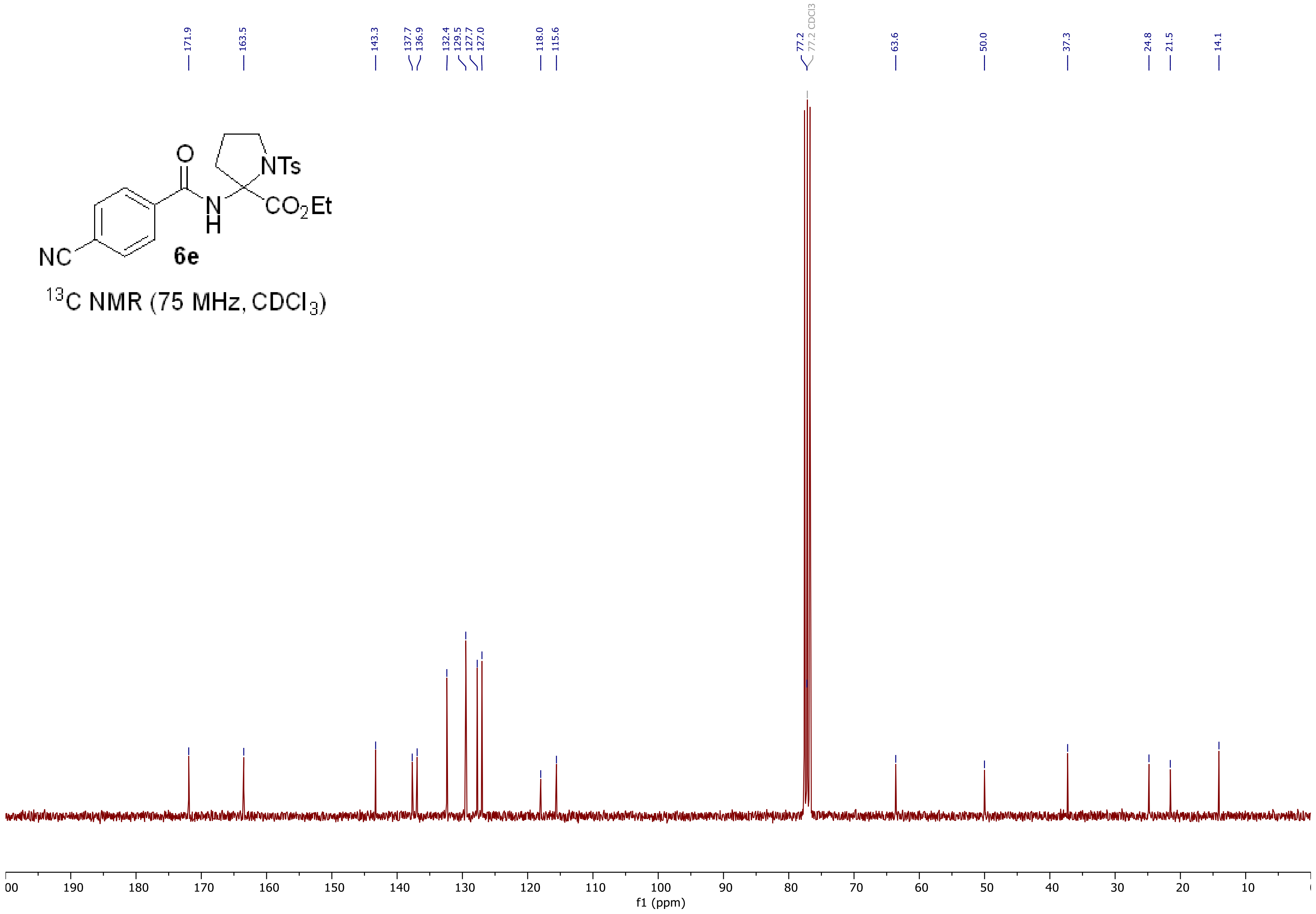
NTs
CO_2Et
N
H
NC
6e
^{13}C NMR (75 MHz, $CDCl_3$)
171.9
163.5
143.3
137.7
136.9
132.4
129.5
127.7
127.0
118.0
115.6
77.2
77.2 CDCl3
63.6
50.0
37.3
24.8
21.5
14.1
00
190
180
170
160
150
140
130
120
110
100
90
80
70
60
50
40
30
20
10
f1 (ppm)

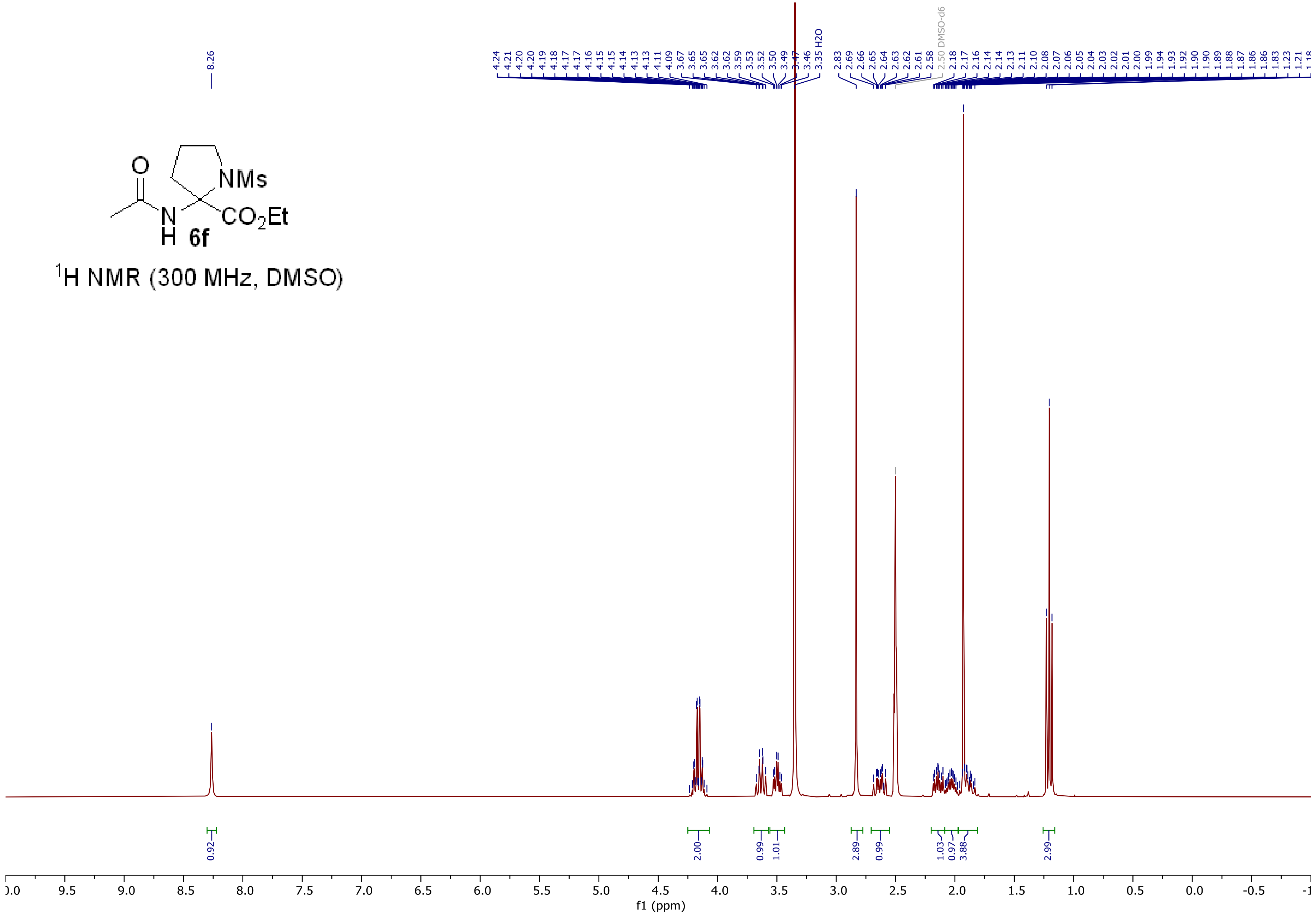
NMs
CO2Et
N
H
O
6f
1H NMR (300 MHz, DMSO)
f1 (ppm)

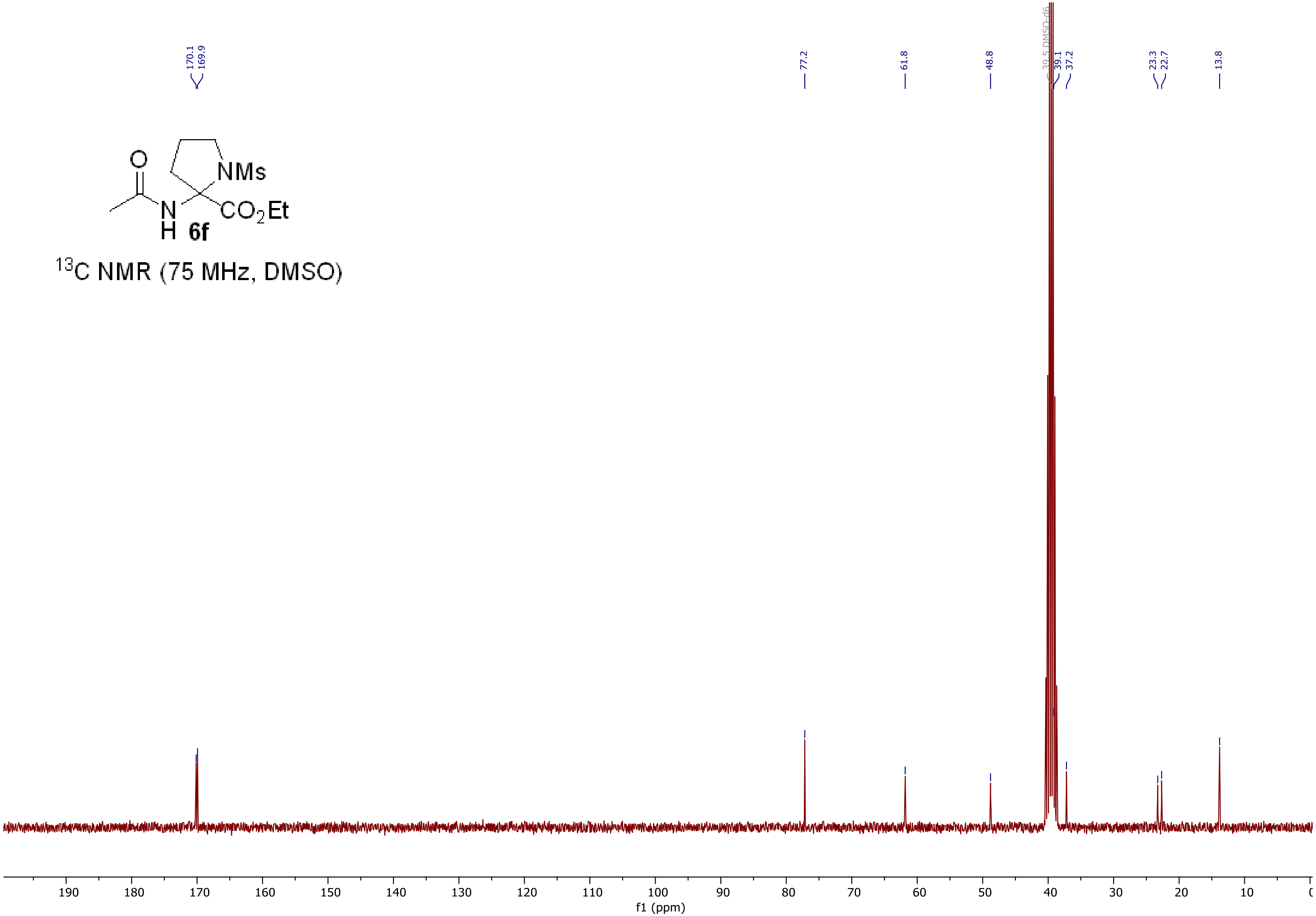

^{13}C NMR (75 MHz, DMSO)

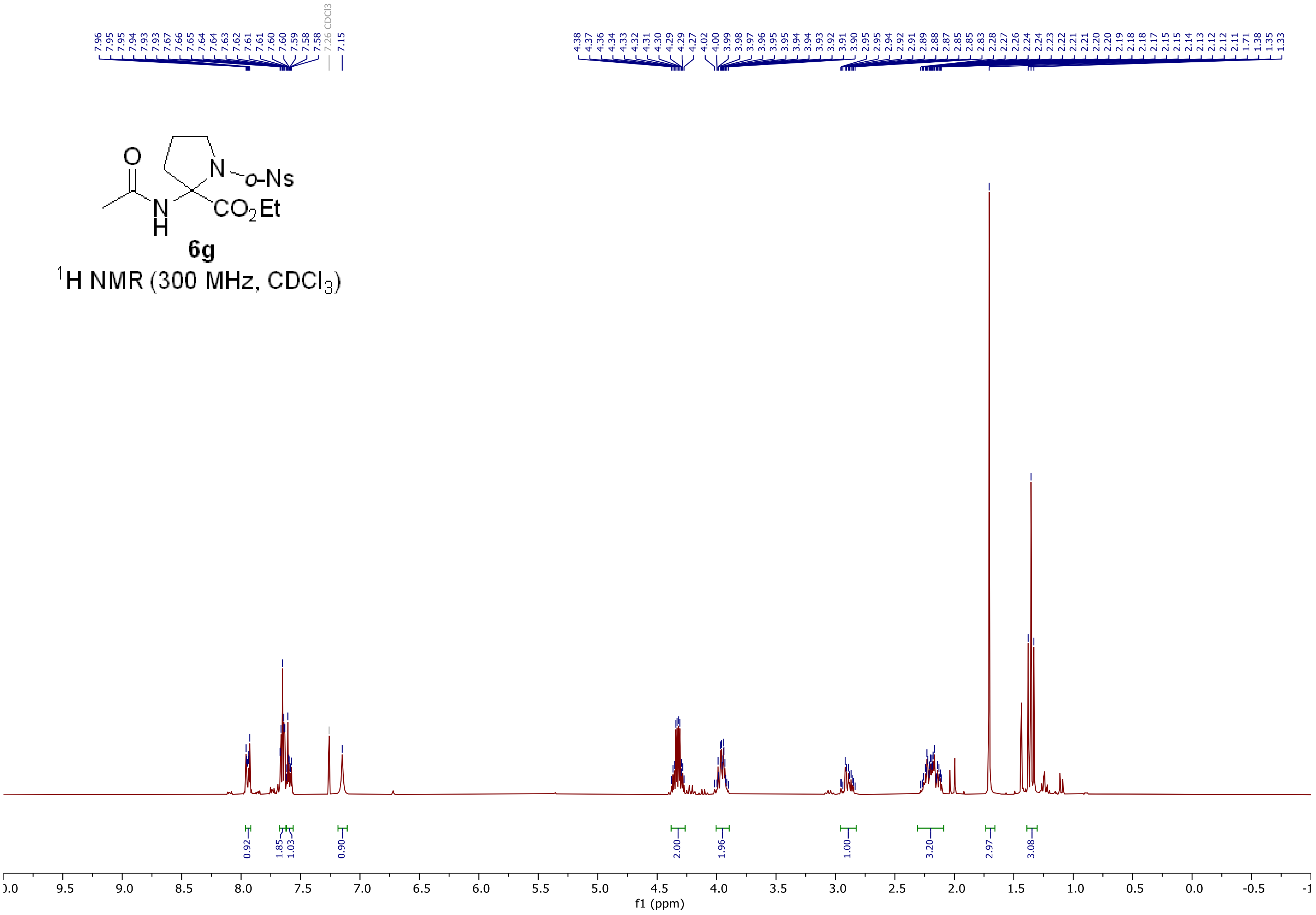

6g

^{1}H NMR (300 MHz, $CDCl_3$)

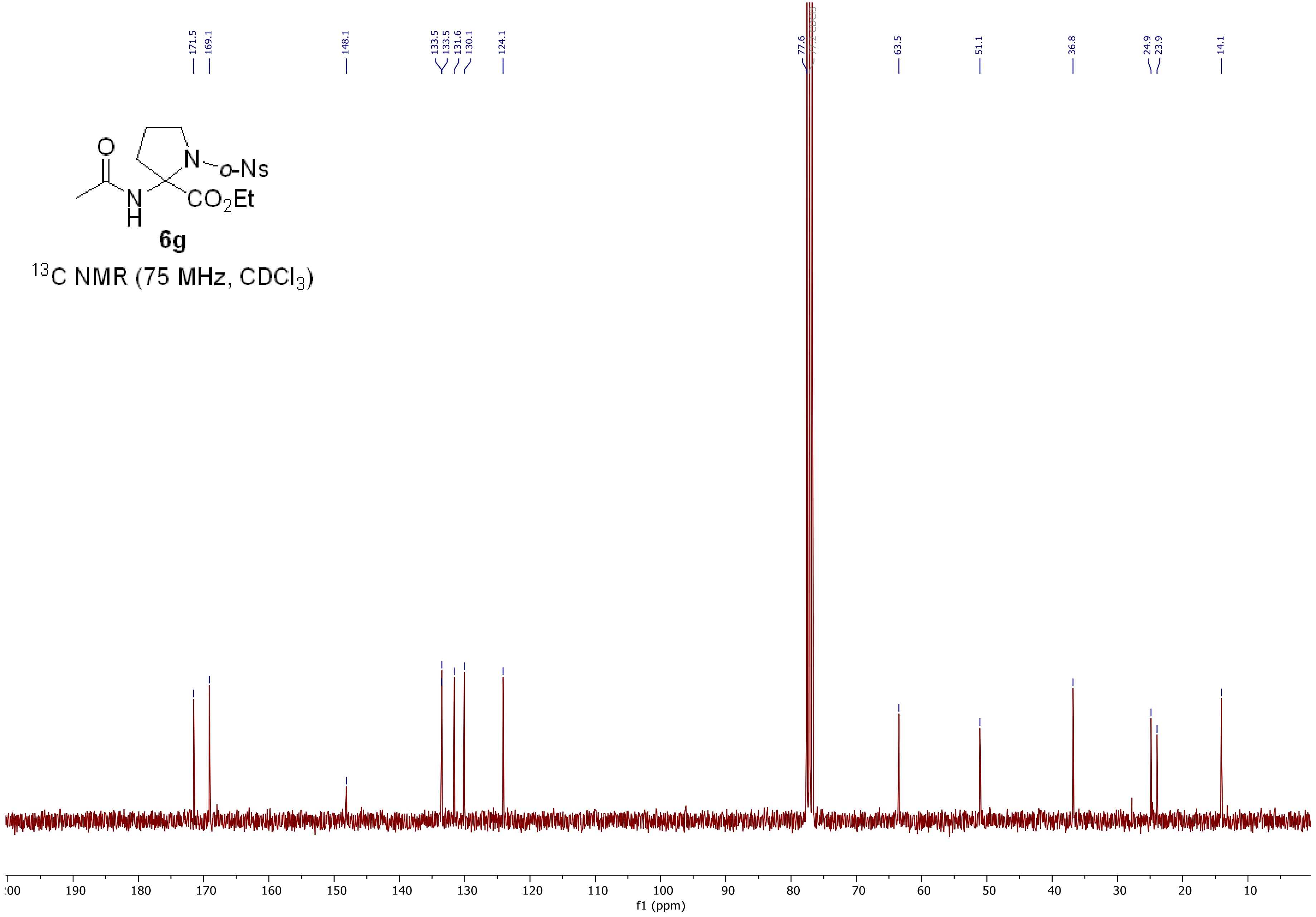

171.5
169.1
148.1
133.5
133.5
131.6
130.1
124.1
77.6
63.5
51.1
36.8
24.9
23.9
14.1
N
o-Ns
CO2Et
N
H
O
6g
13C NMR (75 MHz, CDCl3)
190
180
170
160
150
140
130
120
110
100
90
80
70
60
50
40
30
20
10
f1 (ppm)

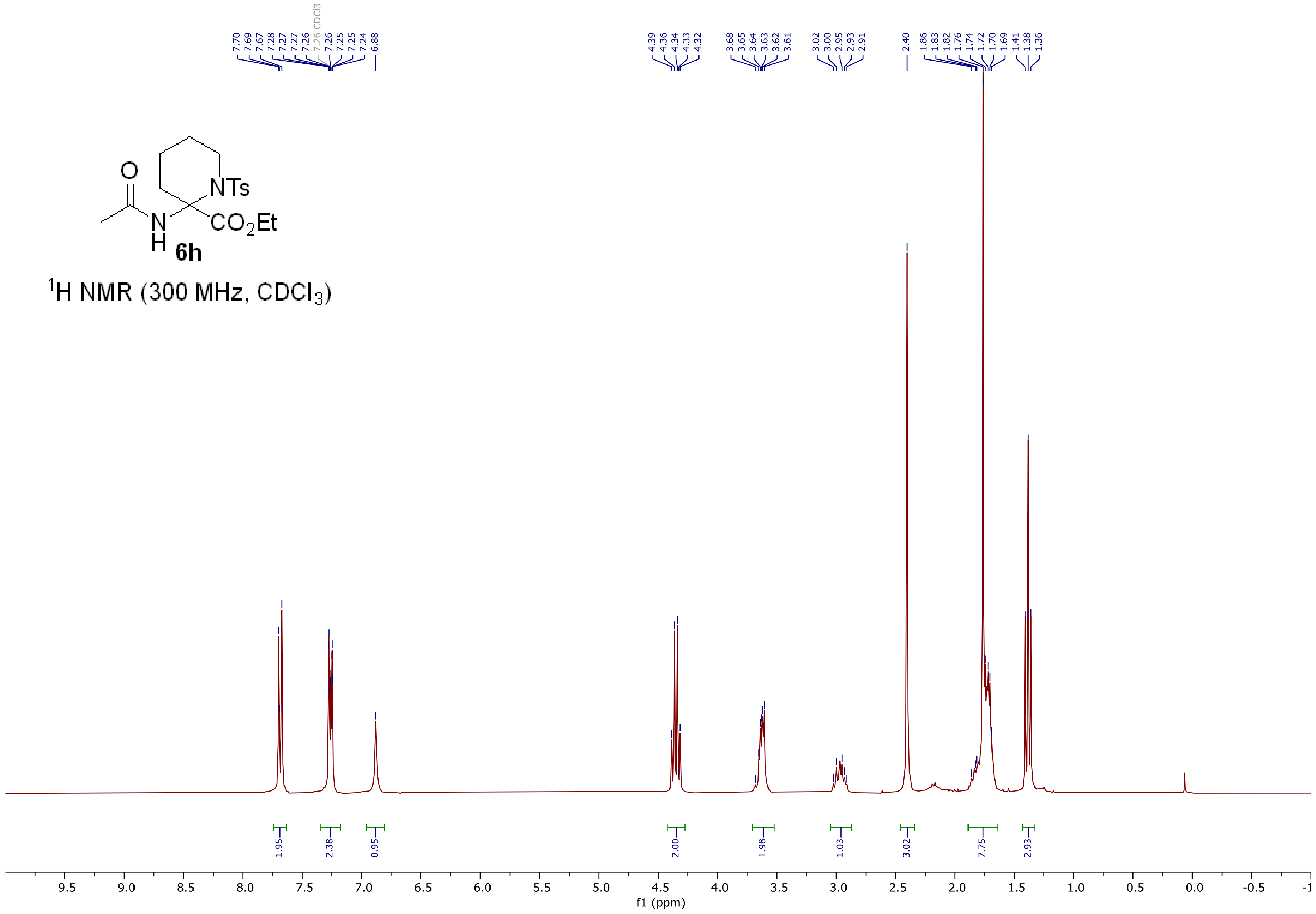
NTs
CO2Et
N
H
6h
^{1}H NMR (300 MHz, CDCl3)
7.70 7.69 7.67 7.28 7.27 7.27 7.26 7.26 CDCl3 7.26 7.25 7.25 7.24 6.88
4.39 4.36 4.34 4.33 4.32
3.68 3.65 3.64 3.63 3.62 3.61
3.02 3.00 2.95 2.93 2.91
2.40
1.86 1.83 1.82 1.76 1.74 1.72 1.70 1.69 1.41 1.38 1.36
1.95 2.38 0.95 2.00 1.98 1.03 3.02 7.75 2.93
f1 (ppm)

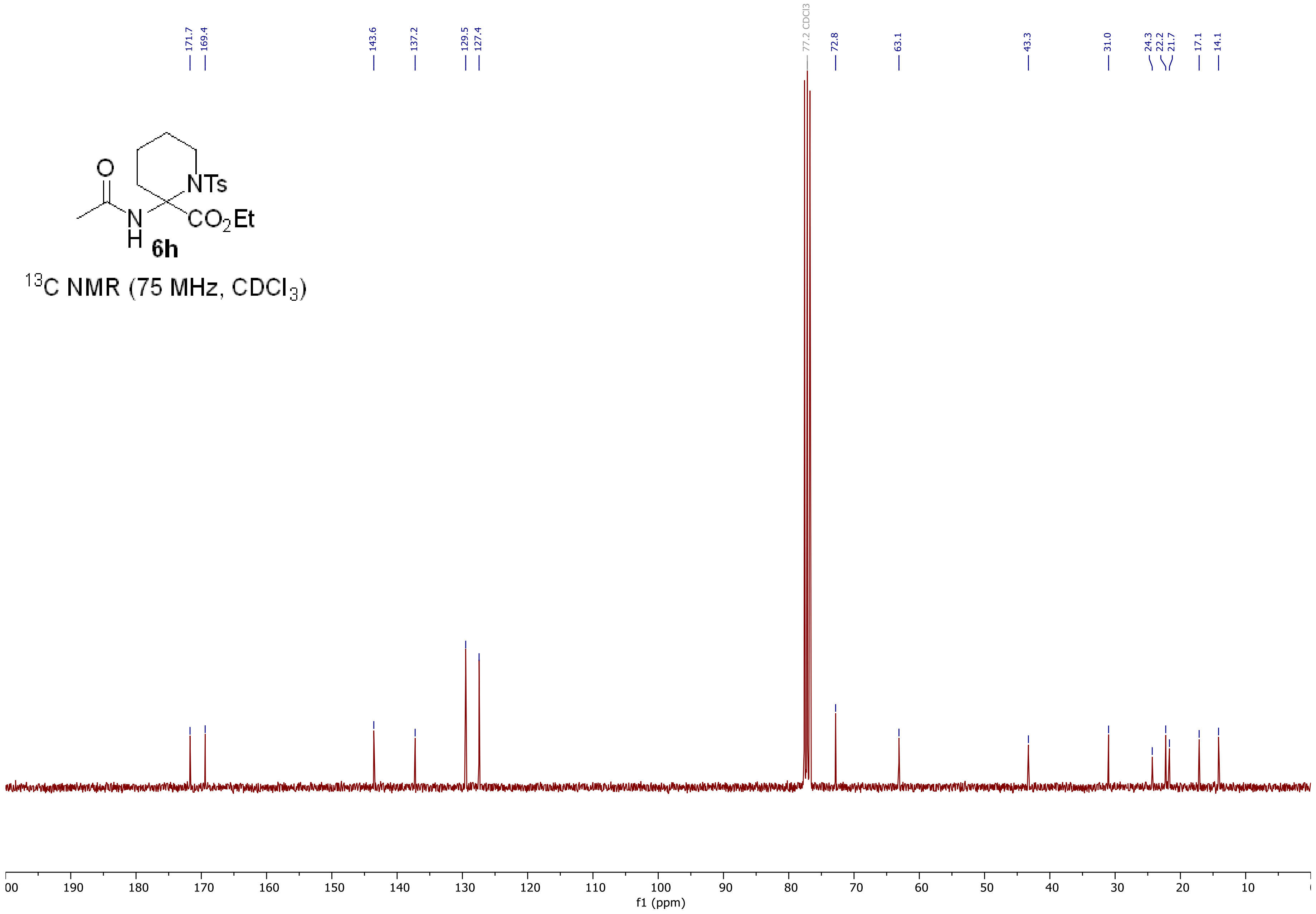

6h

^{13}C NMR (75 MHz, $CDCl_3$)

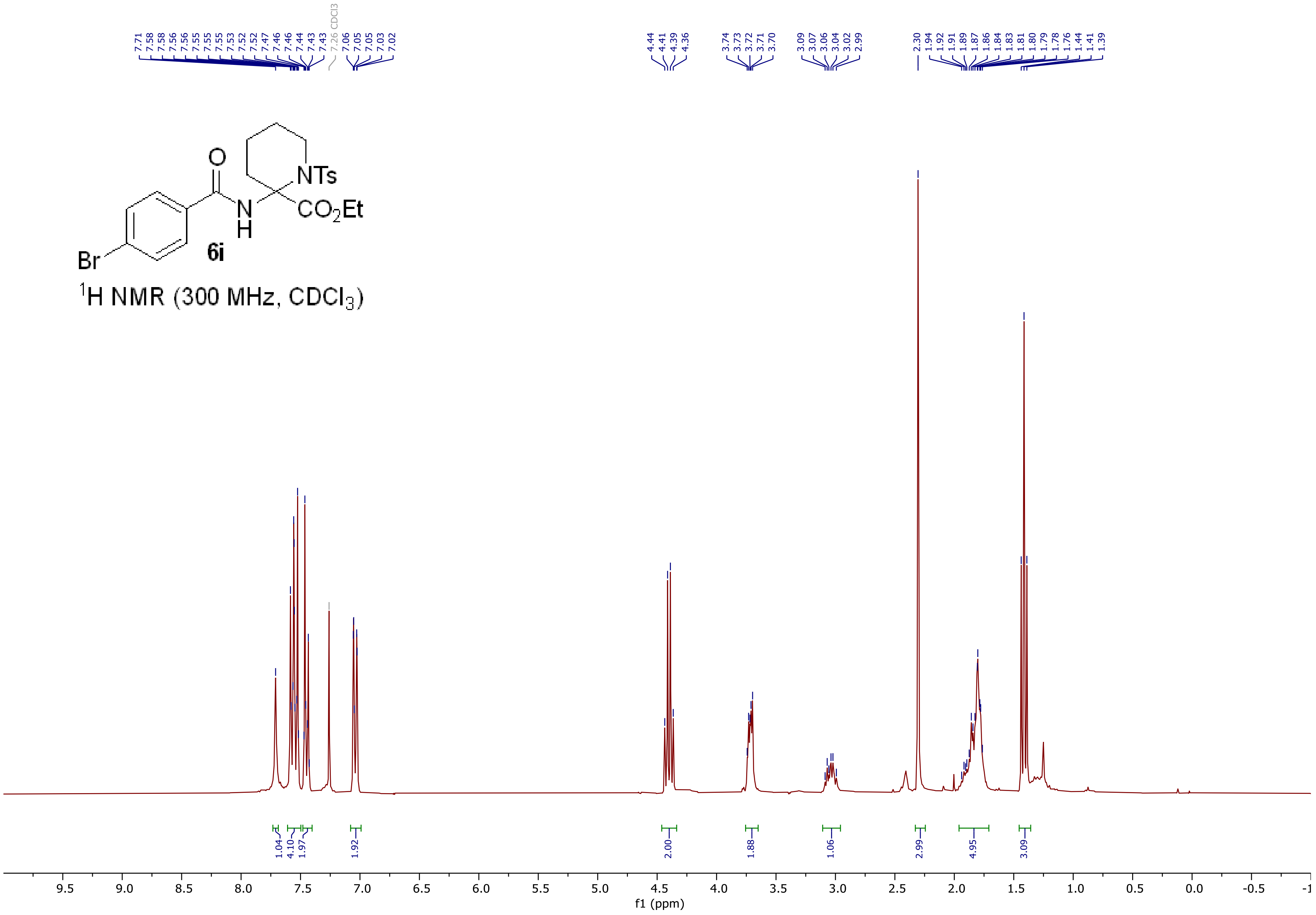

7.71 7.58 7.58 7.56 7.56 7.55 7.55 7.55 7.53 7.52 7.52 7.47 7.46 7.46 7.44 7.43 7.43 7.26 CDCl3 7.06 7.05 7.05 7.03 7.02
4.44 4.41 4.39 4.36
3.74 3.73 3.72 3.71 3.70
3.09 3.07 3.06 3.04 3.02 2.99
2.30 1.94 1.92 1.91 1.89 1.87 1.86 1.84 1.83 1.81 1.80 1.79 1.78 1.76 1.44 1.41 1.39
O
NTs
N
H
CO2Et
Br
6i
1H NMR (300 MHz, CDCl3)
1.04 4.10 1.97 1.92 2.00 1.88 1.06 2.99 4.95 3.09
9.5 9.0 8.5 8.0 7.5 7.0 6.5 6.0 5.5 5.0 4.5 4.0 3.5 3.0 2.5 2.0 1.5 1.0 0.5 0.0 -0.5 -1
f1 (ppm)

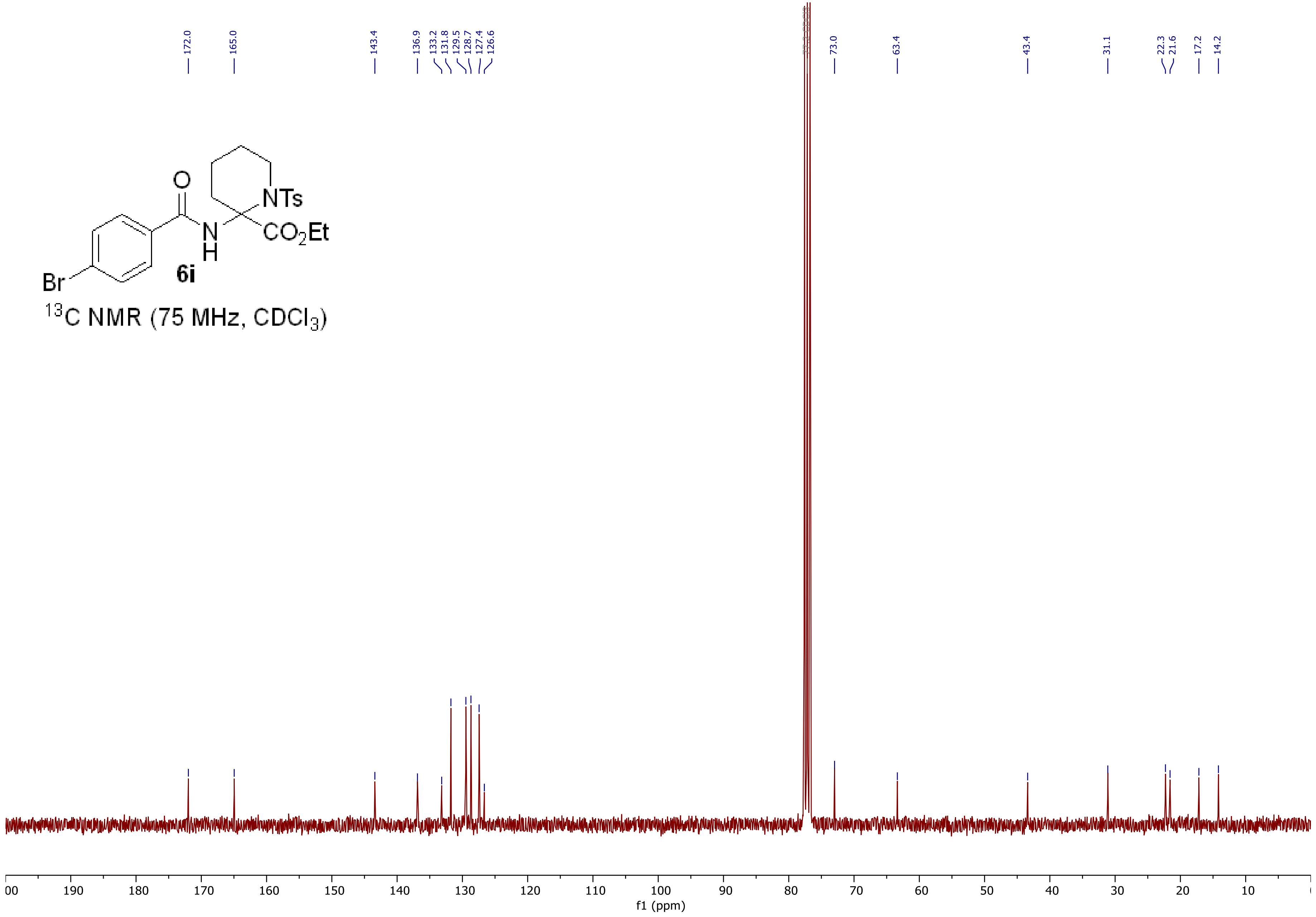

6i

^{13}C NMR (75 MHz, $CDCl_3$)

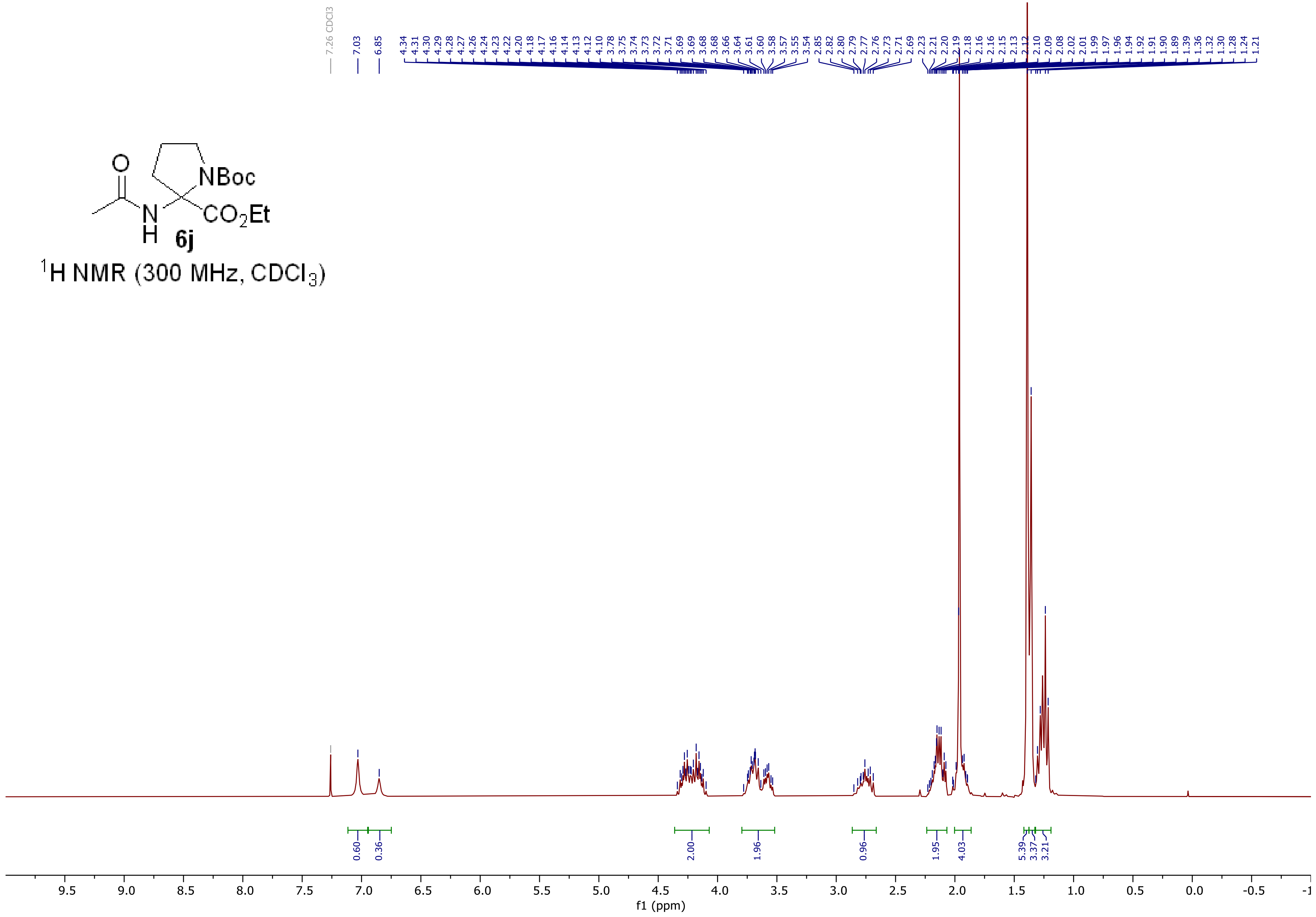
NBoc
CO2Et
N
H
6j
1H NMR (300 MHz, CDCl3)
f1 (ppm)

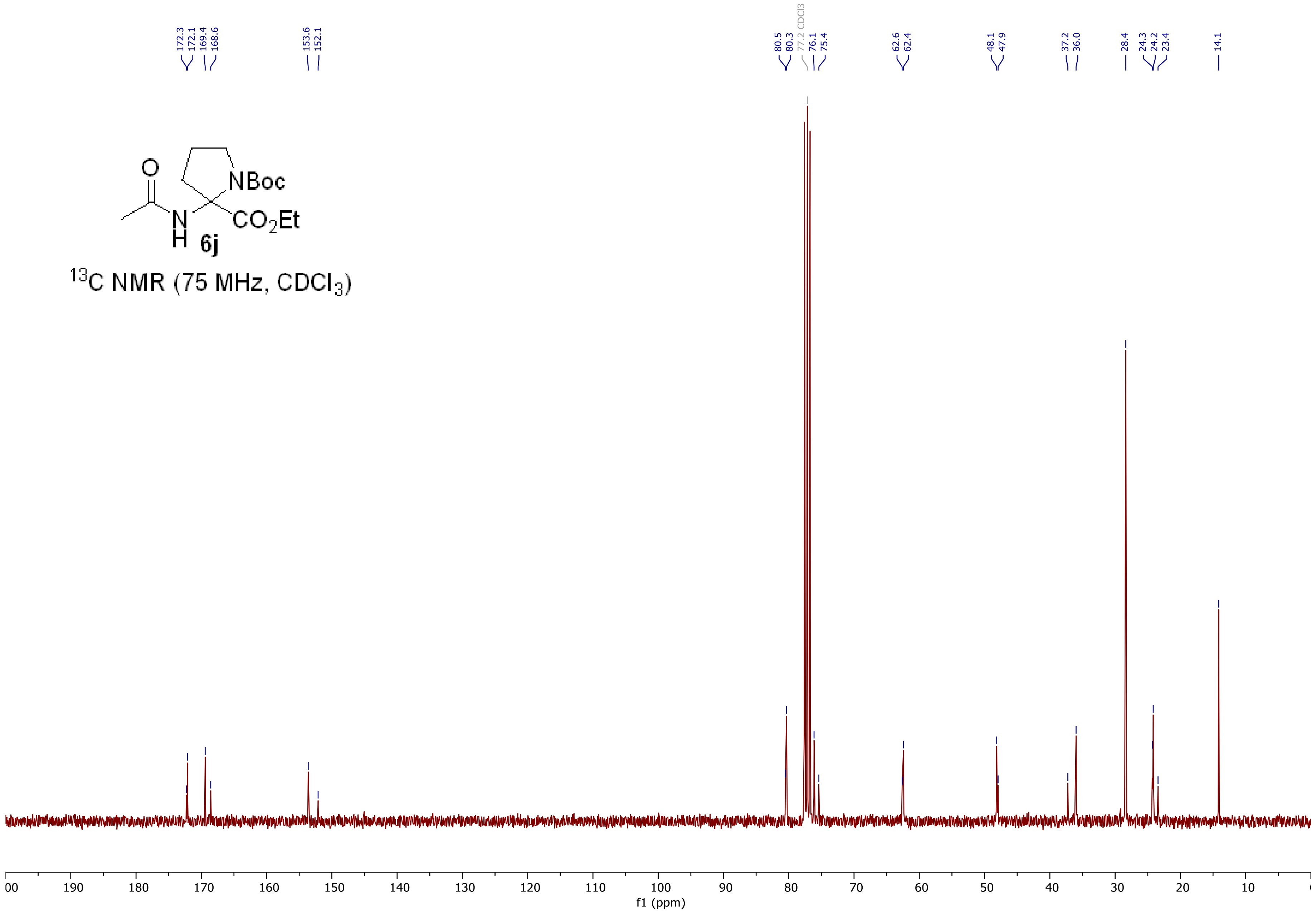
172.3
172.1
169.4
168.6
153.6
152.1
80.5
80.3
77.2 CDCl3
76.1
75.4
62.6
62.4
48.1
47.9
37.2
36.0
28.4
24.3
24.2
23.4
14.1
NBoc
N
H
CO_2Et
6j
^{13}C NMR (75 MHz, $CDCl_3$)
f1 (ppm)

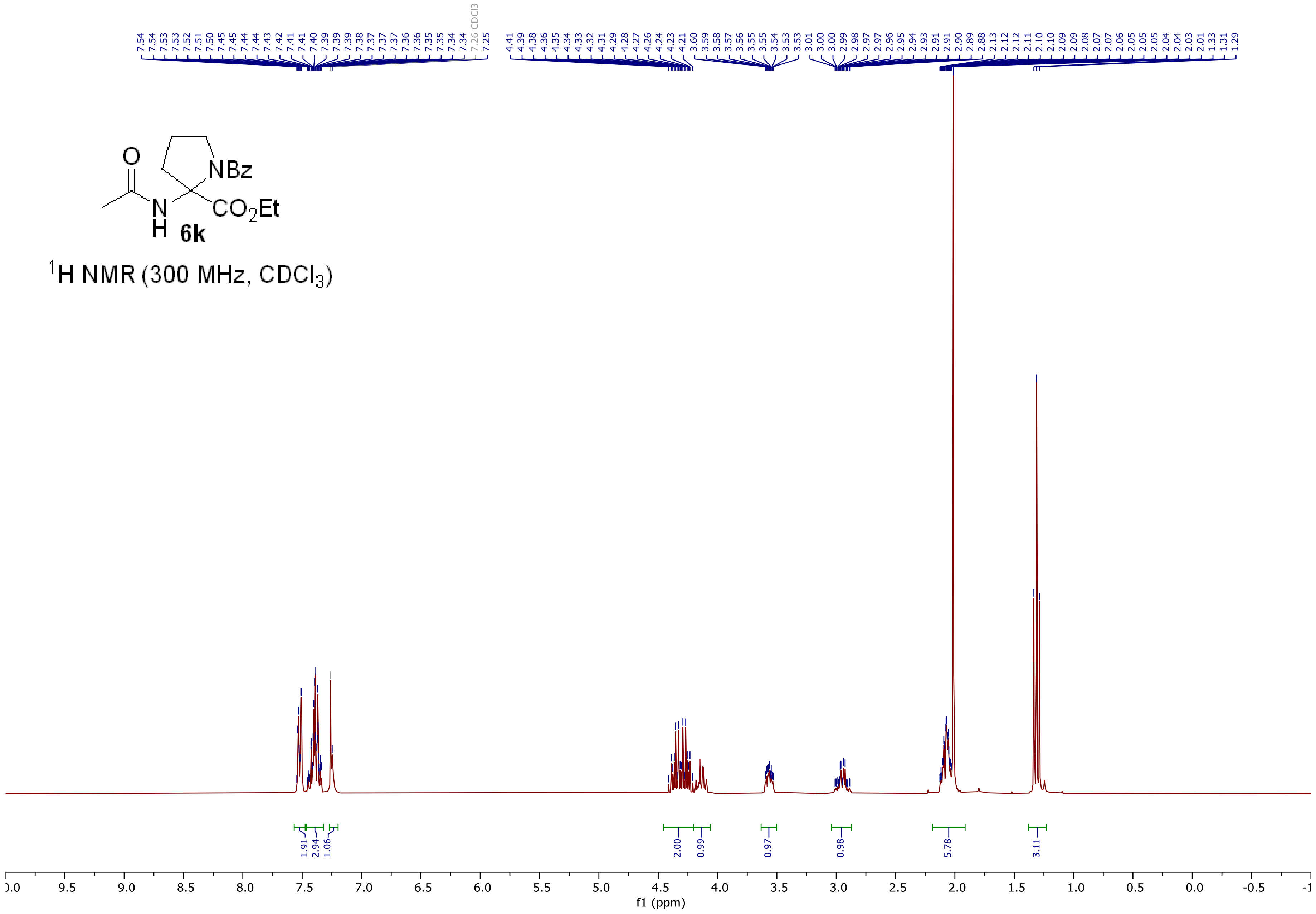

6k

^{1}H NMR (300 MHz, $CDCl_3$)

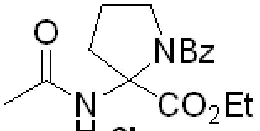

6k

^{13}C NMR (75 MHz, MeCN-d_3)

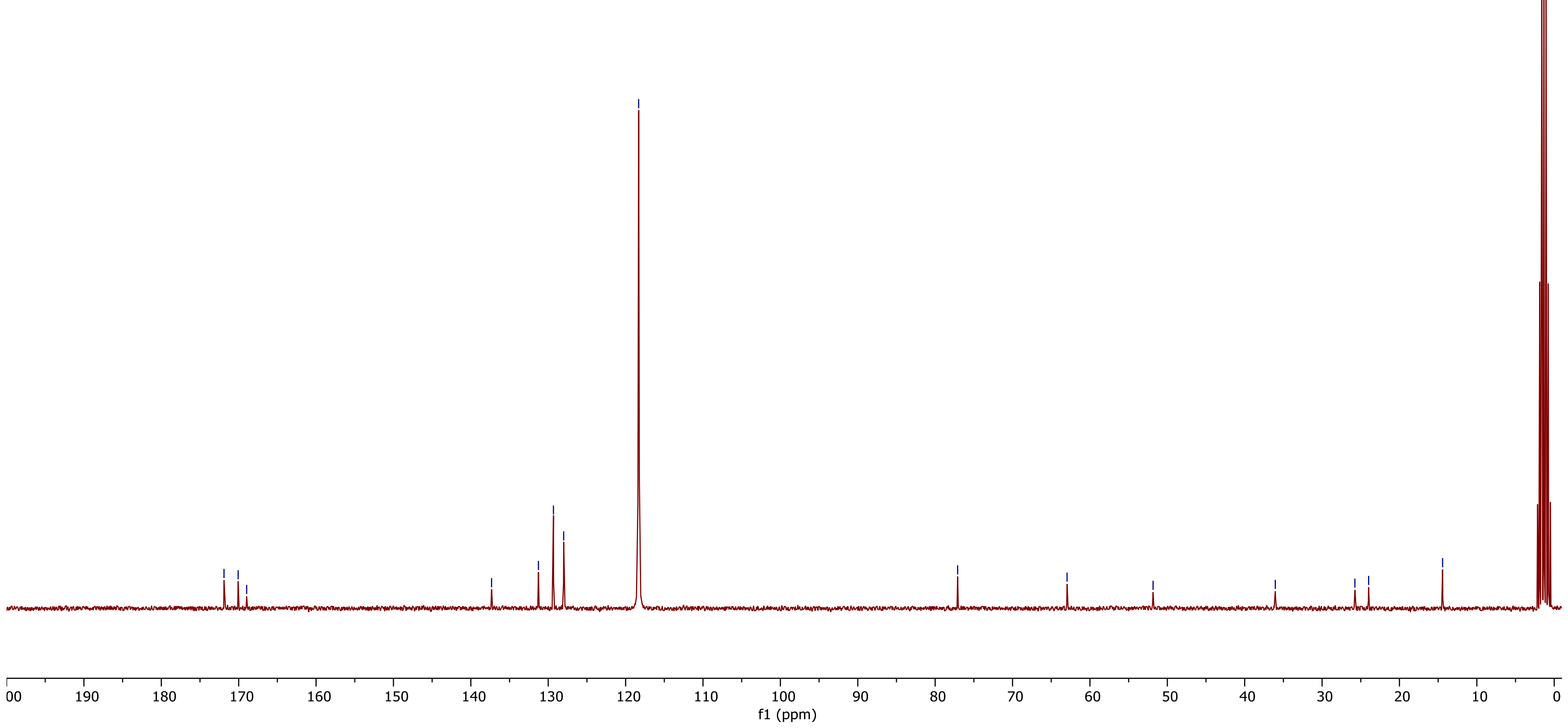

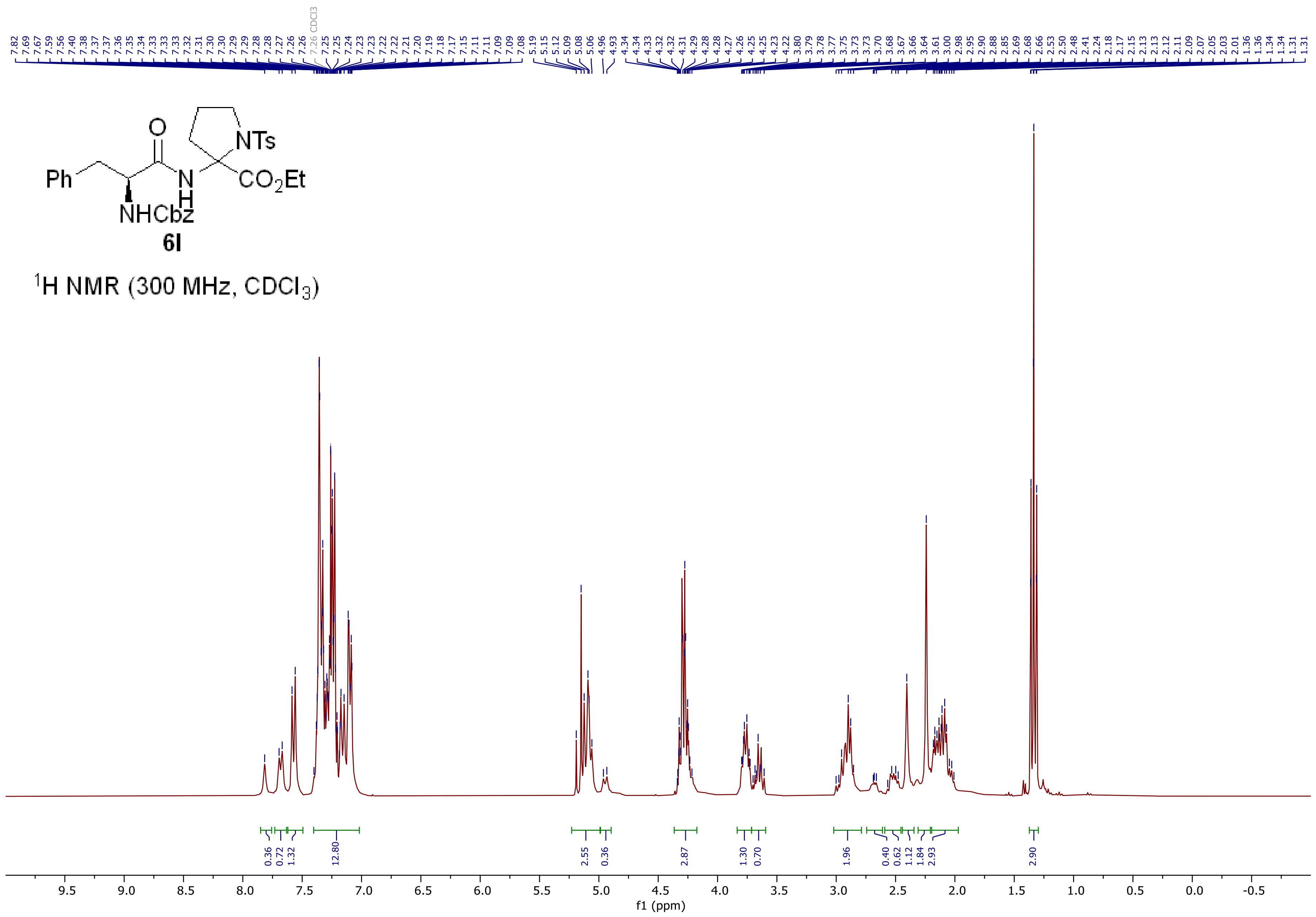

6l

^1H NMR (300 MHz, $CDCl_3$)

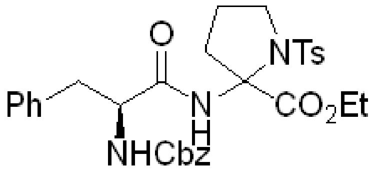

6l

^{13}C NMR (75 MHz, $CDCl_3$)

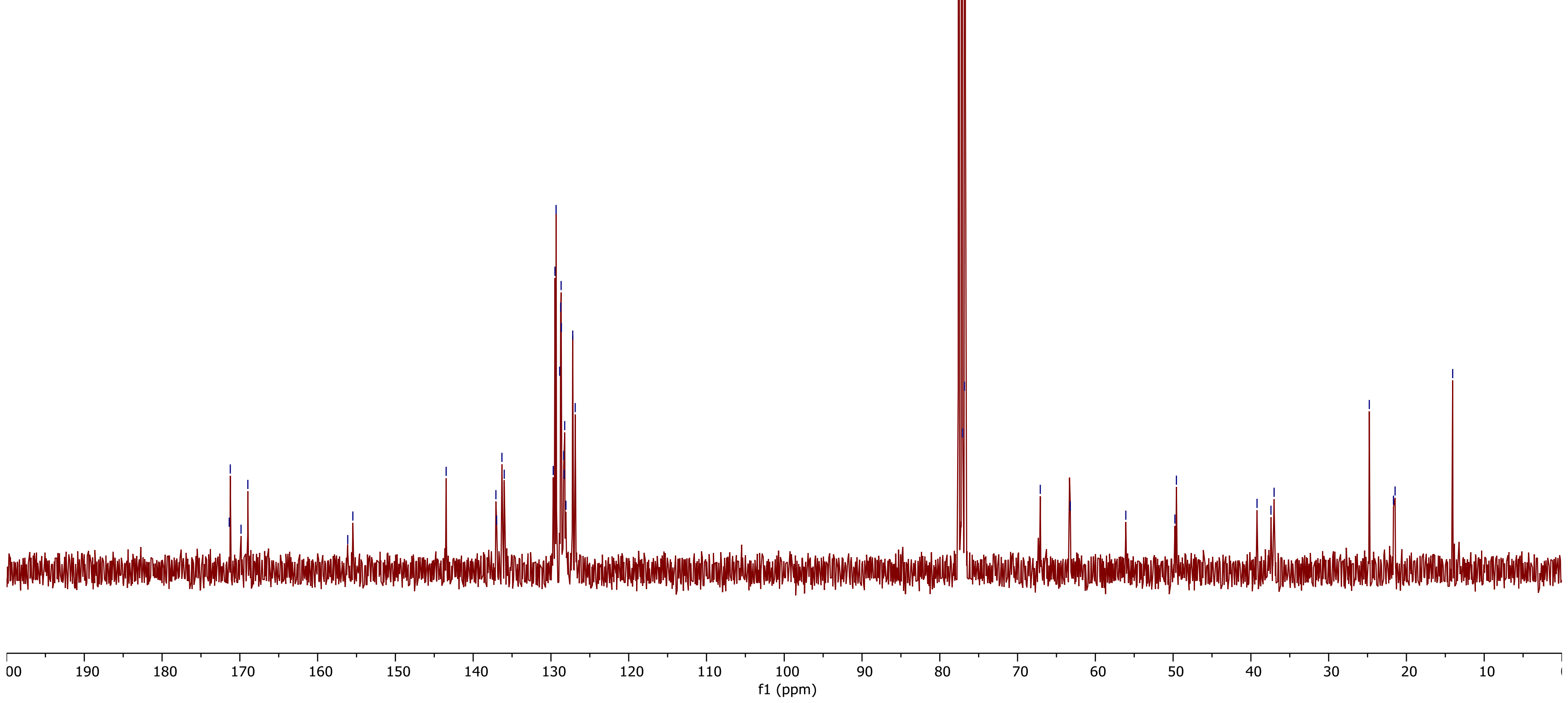

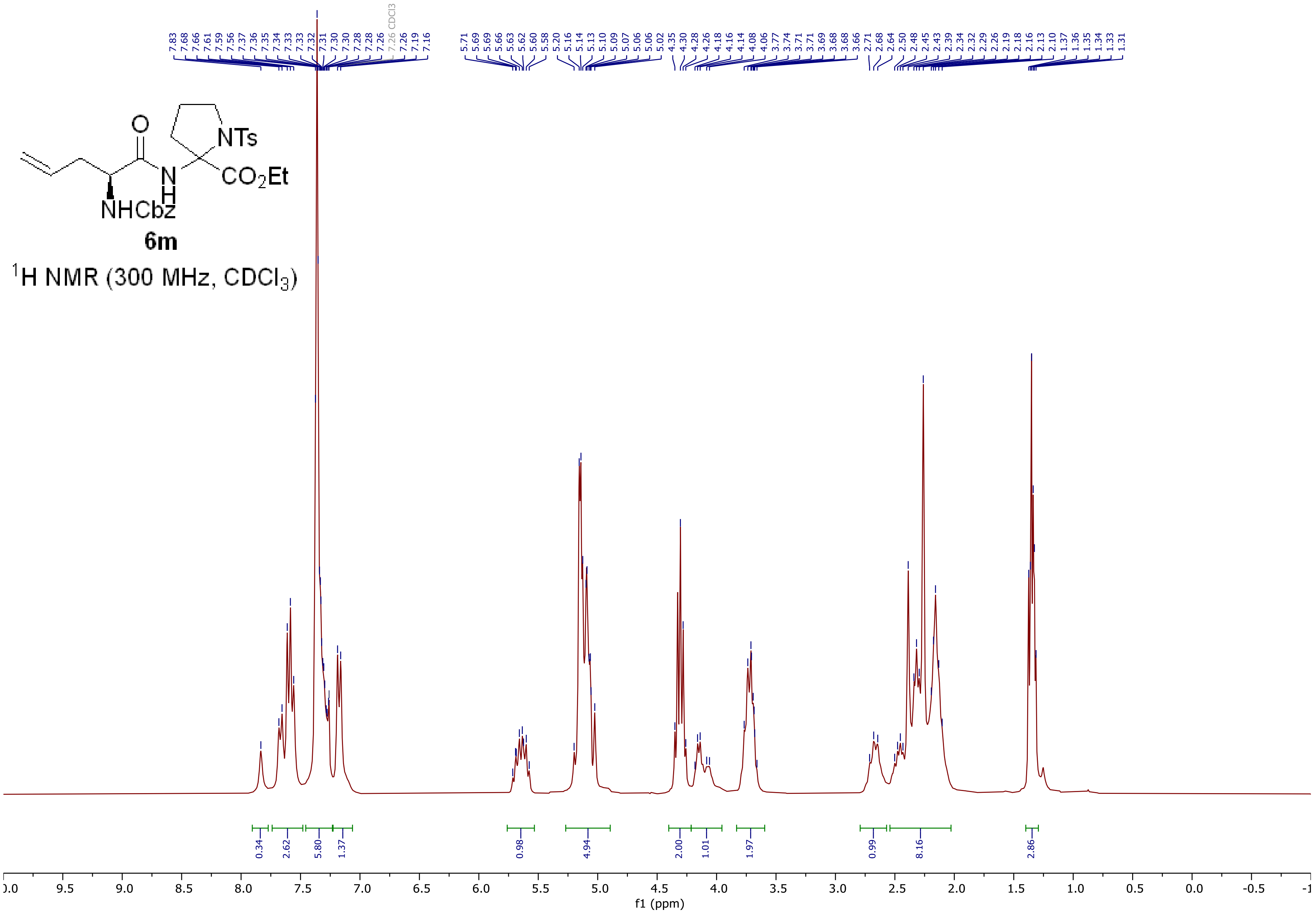

6m

^{1}H NMR (300 MHz, $CDCl_3$)

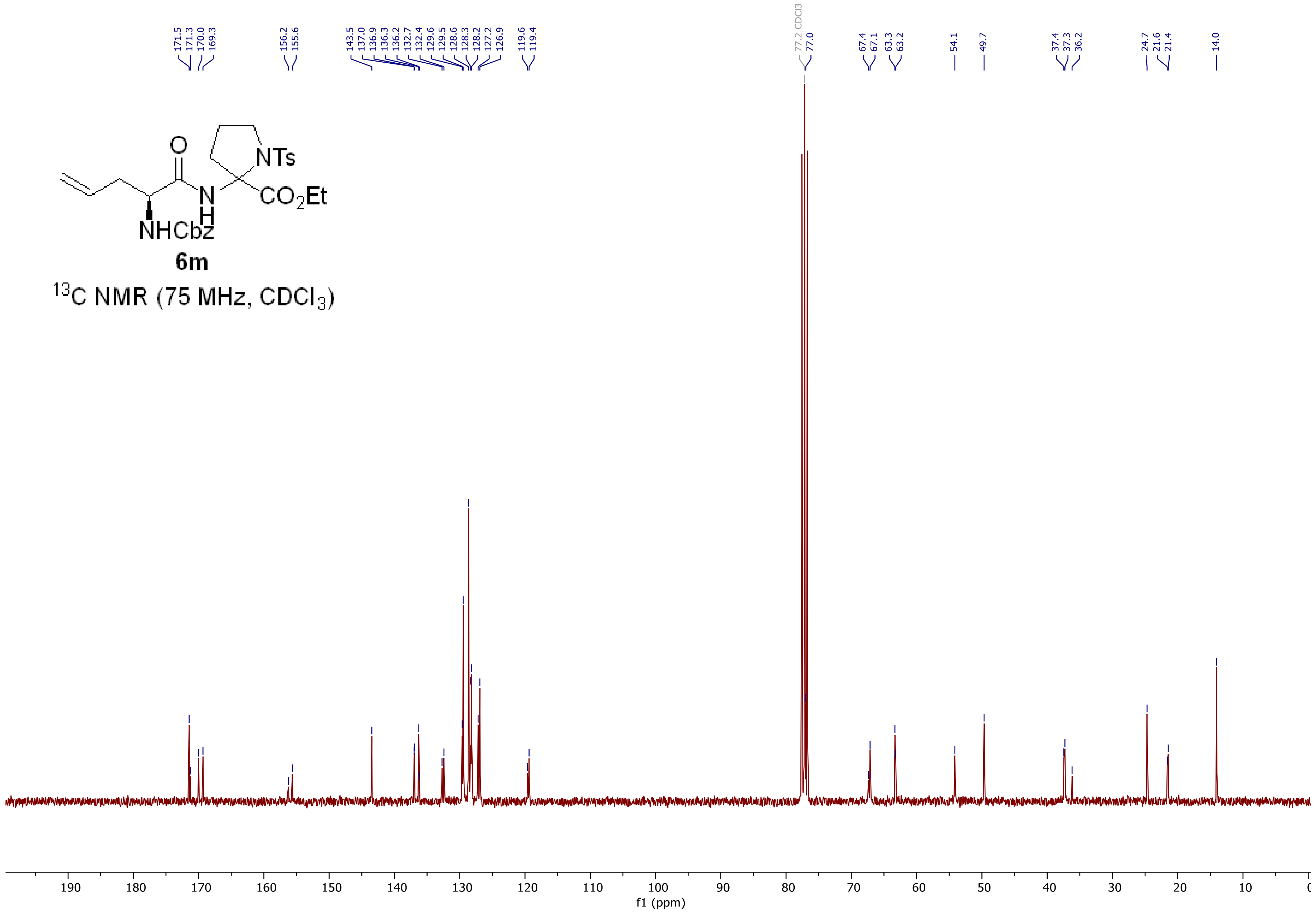
NTs
CO2Et
N
H
O
NHCbz
6m
13C NMR (75 MHz, CDCl3)
171.5
171.3
170.0
169.3
156.2
155.6
143.5
137.0
136.9
136.3
136.2
132.7
132.4
129.6
129.5
128.6
128.3
128.2
127.2
126.9
119.6
119.4
77.2 CDCl3
77.0
67.4
67.1
63.3
63.2
54.1
49.7
37.4
37.3
36.2
24.7
21.6
21.4
14.0
f1 (ppm)

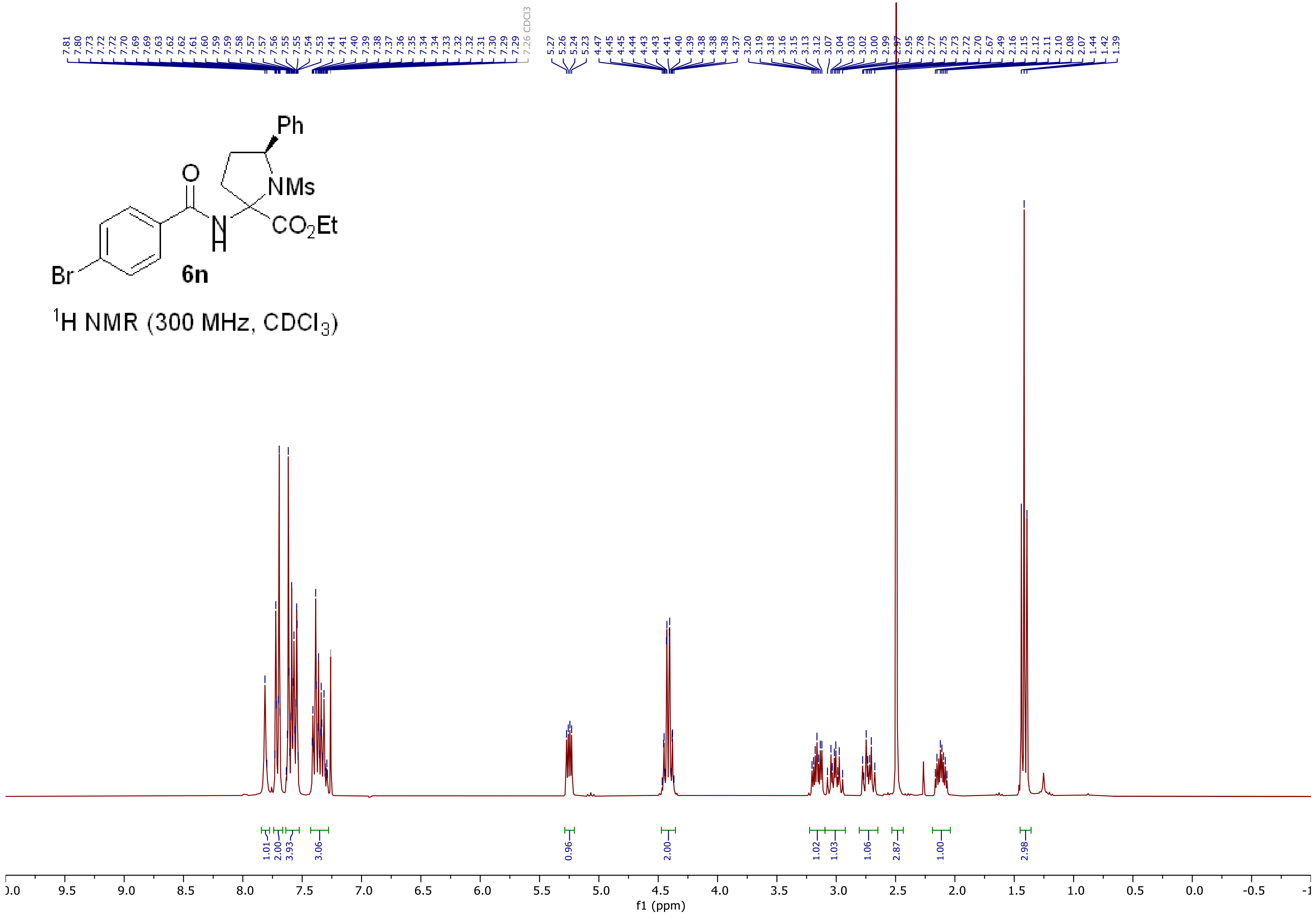

6n

^{1}H NMR (300 MHz, $CDCl_3$)

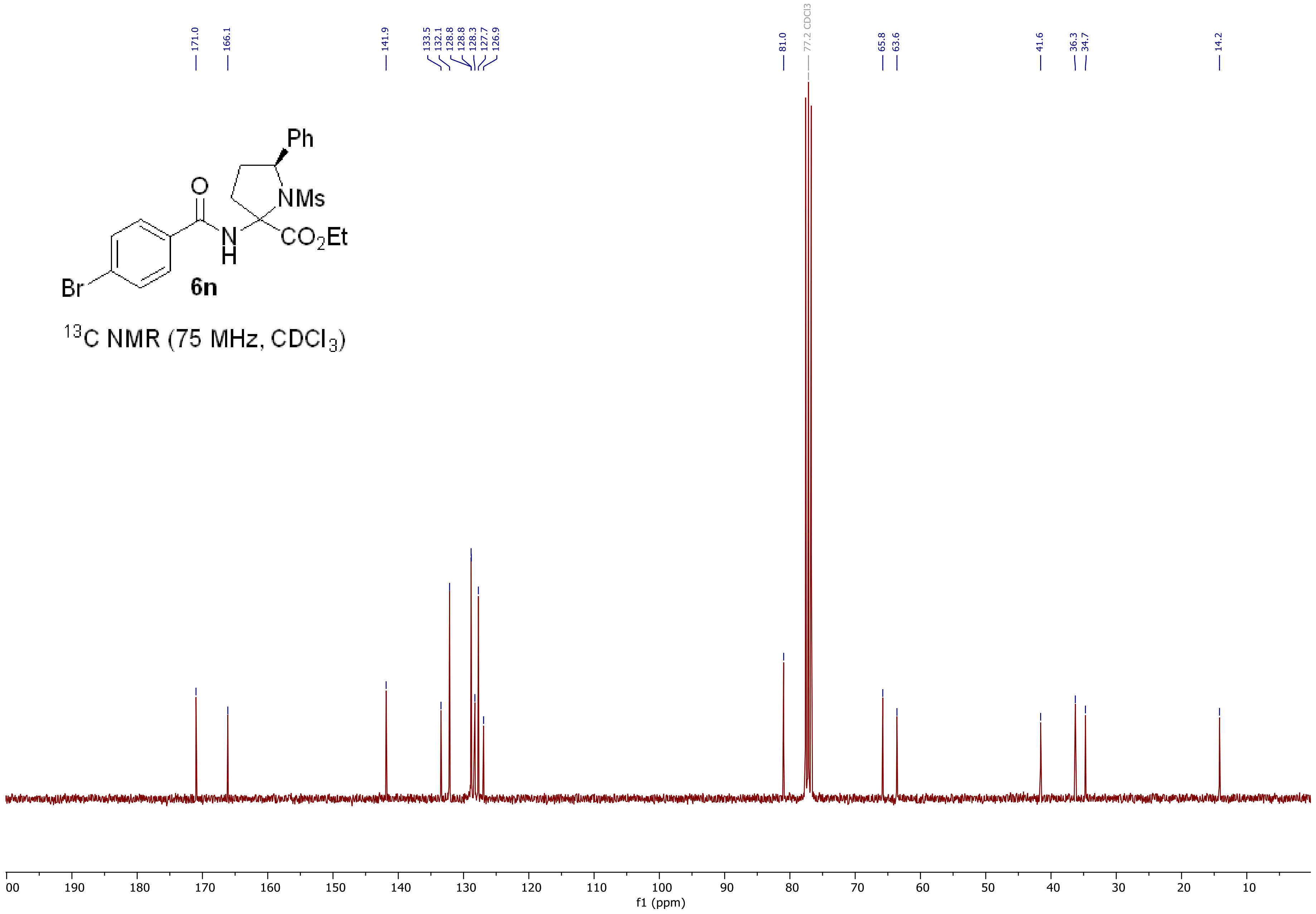
Ph
O
NMs
N
H
CO2Et
Br
6n
13C NMR (75 MHz, CDCl3)
171.0
166.1
141.9
133.5
132.1
128.8
128.8
128.3
127.7
126.9
81.0
77.2 CDCl3
65.8
63.6
41.6
36.3
34.7
14.2
f1 (ppm)

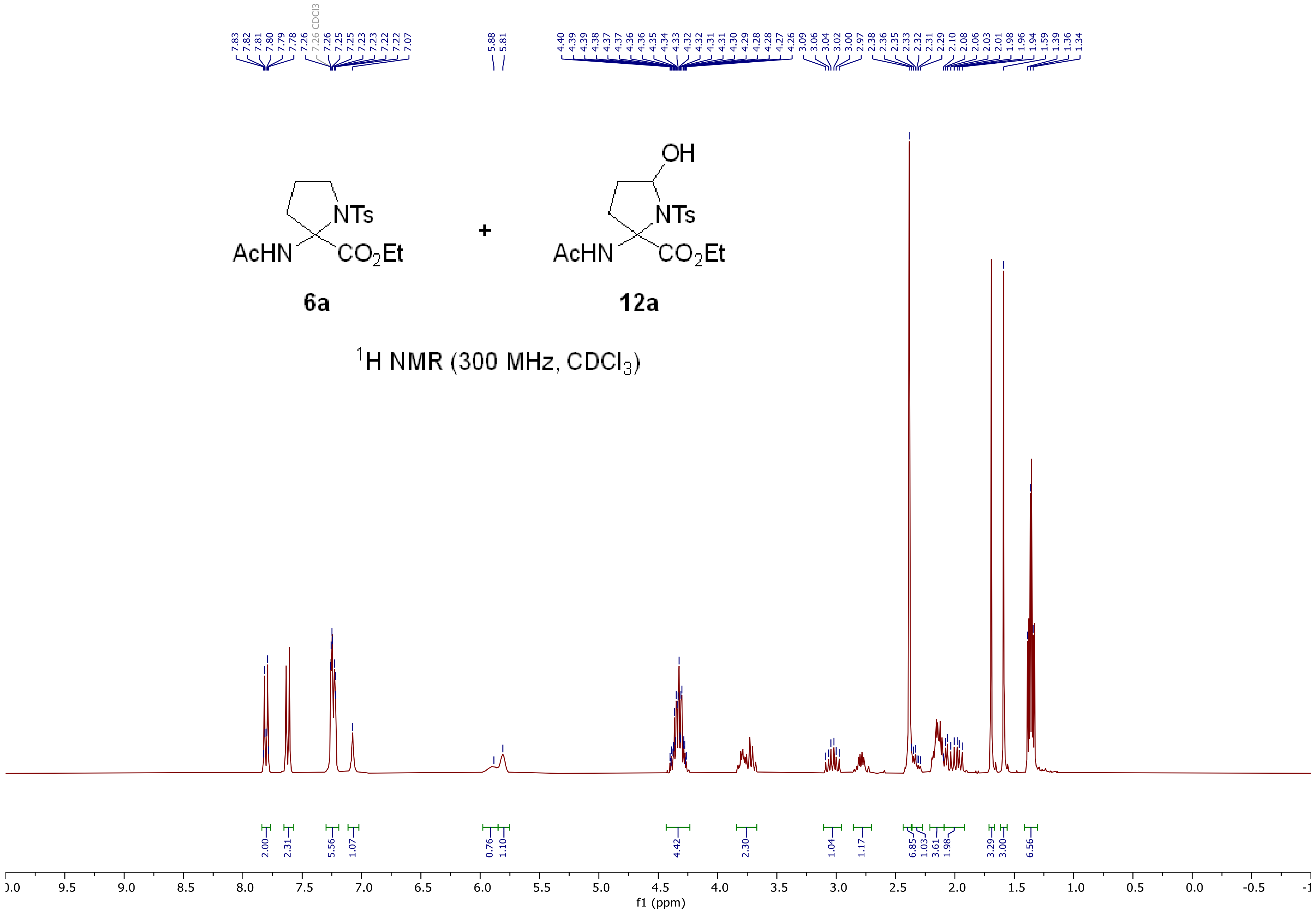

6a
12a
OH
NTs
AcHN
CO2Et
+
1H NMR (300 MHz, CDCl3)
f1 (ppm)

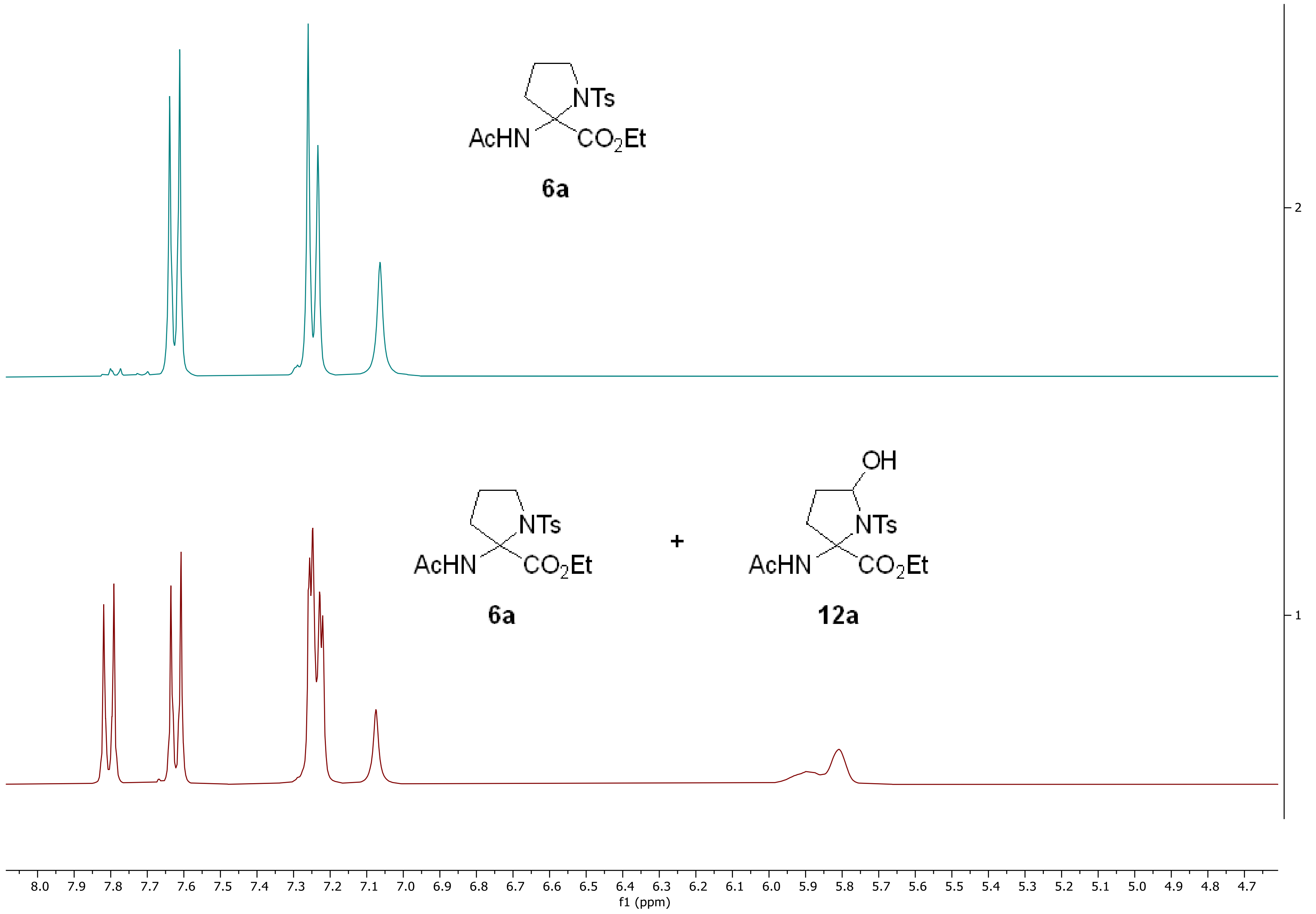
NTs
AcHN
CO2Et
6a
OH
NTs
AcHN
CO2Et
6a
+
12a
2
1
8.0 7.9 7.8 7.7 7.6 7.5 7.4 7.3 7.2 7.1 7.0 6.9 6.8 6.7 6.6 6.5 6.4 6.3 6.2 6.1 6.0 5.9 5.8 5.7 5.6 5.5 5.4 5.3 5.2 5.1 5.0 4.9 4.8 4.7
f1 (ppm)

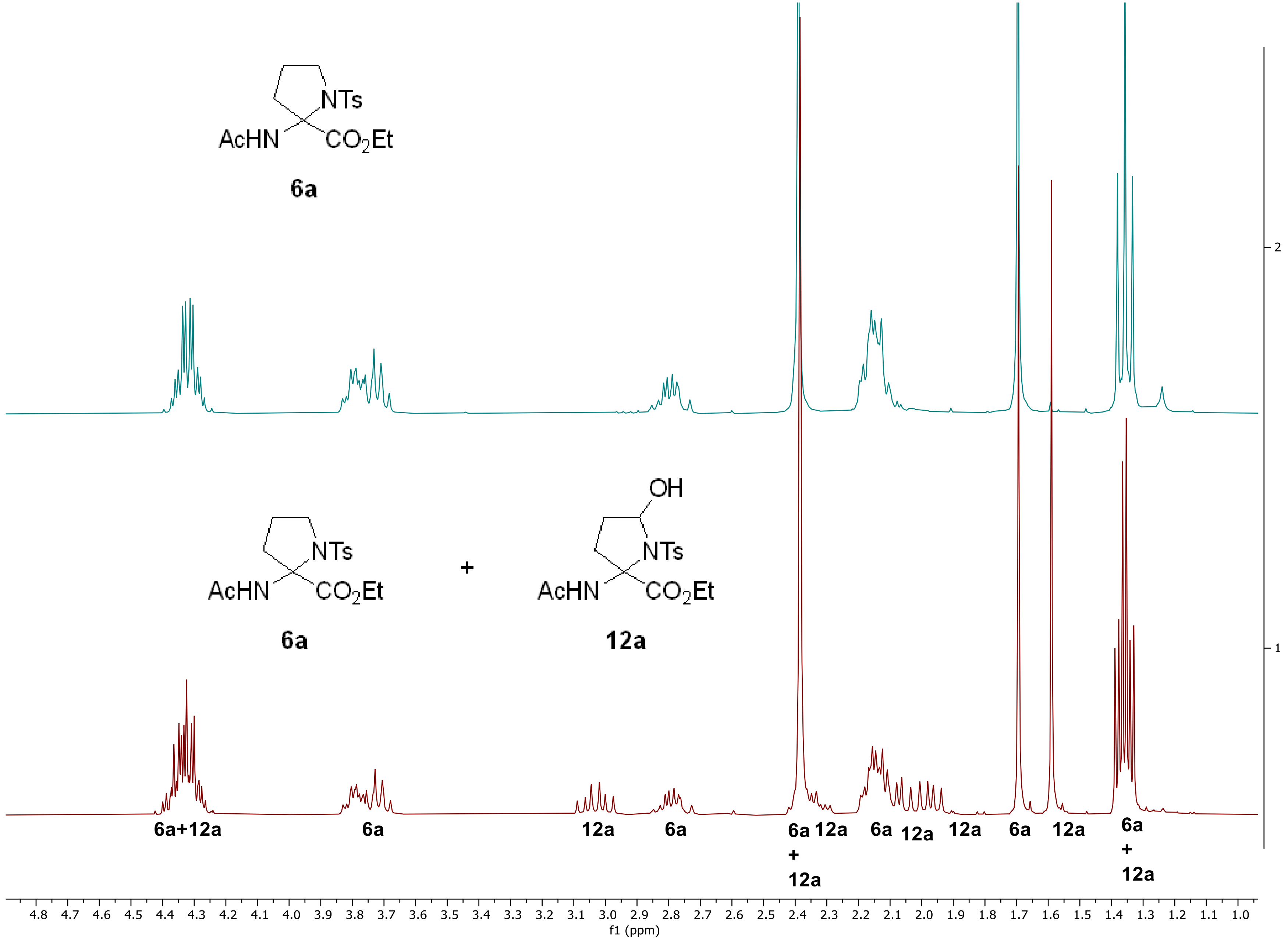
NTs
AcHN
CO2Et
6a
+
OH
12a
6a+12a
6a
12a
6a
6a
+
12a
12a
6a
12a
12a
6a
12a
6a
+
12a
4.8 4.7 4.6 4.5 4.4 4.3 4.2 4.1 4.0 3.9 3.8 3.7 3.6 3.5 3.4 3.3 3.2 3.1 3.0 2.9 2.8 2.7 2.6 2.5 2.4 2.3 2.2 2.1 2.0 1.9 1.8 1.7 1.6 1.5 1.4 1.3 1.2 1.1 1.0
f1 (ppm)
2
1

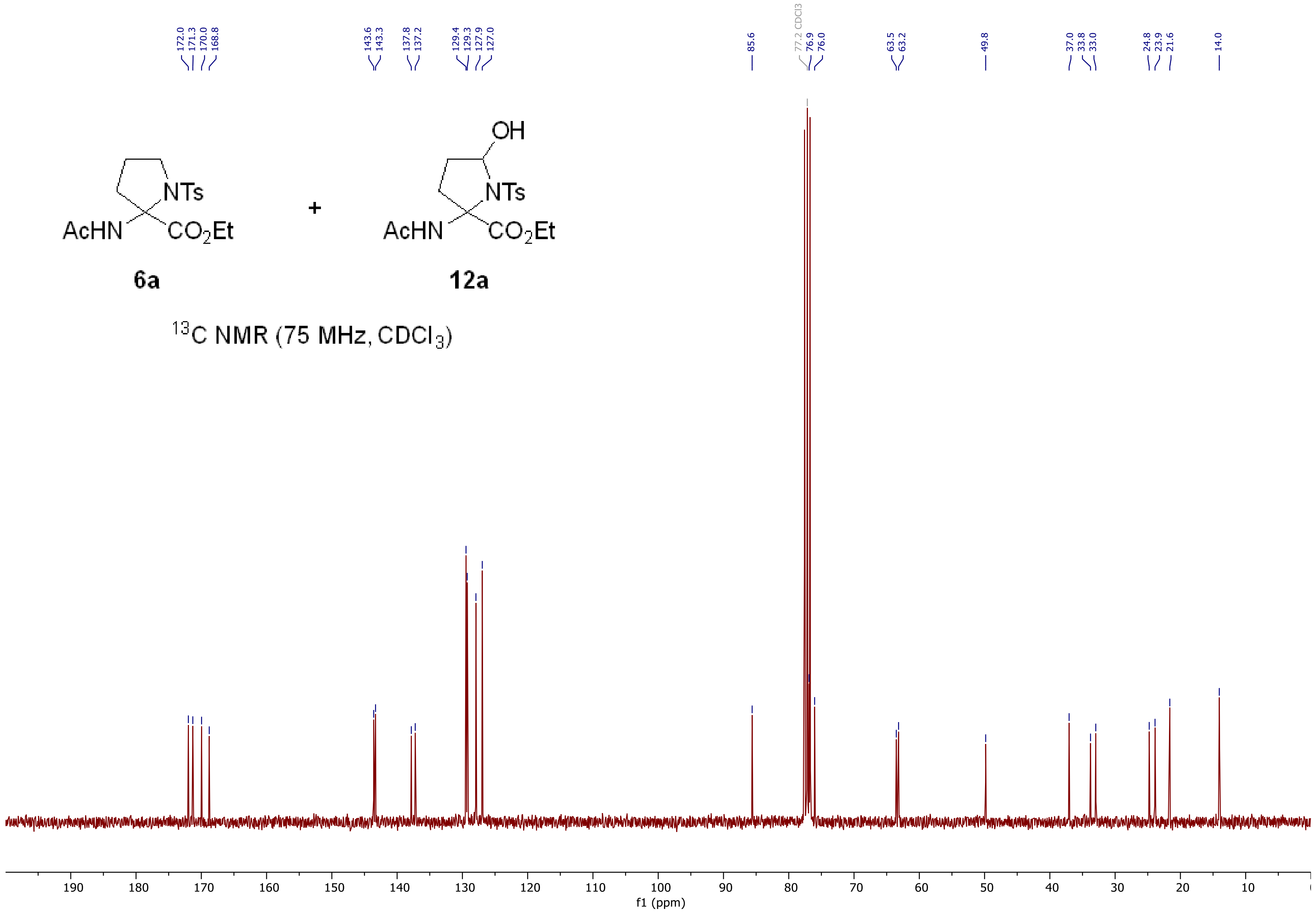

172.0
171.3
170.0
168.8
143.6
143.3
137.8
137.2
129.4
129.3
127.9
127.0
85.6
77.2 CDCl3
76.9
76.0
63.5
63.2
49.8
37.0
33.8
33.0
24.8
23.9
21.6
14.0
OH
NTs
AcHN
CO2Et
+
6a
12a
13C NMR (75 MHz, CDCl3)
190
180
170
160
150
140
130
120
110
100
90
80
70
60
50
40
30
20
10
f1 (ppm)

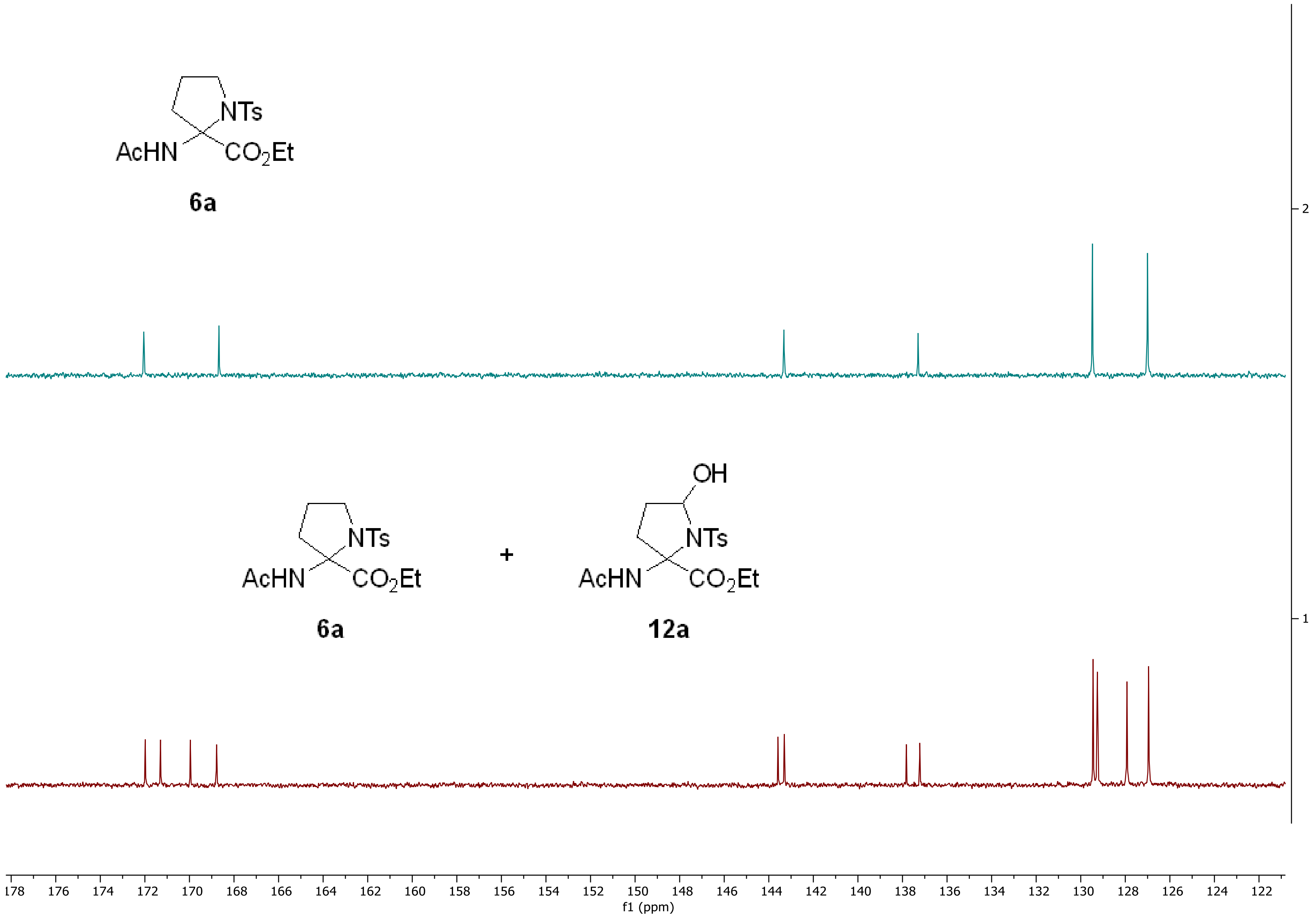
NTs
AcHN
CO2Et
6a
OH
NTs
AcHN
CO2Et
6a
12a
+
2
1
178
176
174
172
170
168
166
164
162
160
158
156
154
152
150
148
146
144
142
140
138
136
134
132
130
128
126
124
122
f1 (ppm)

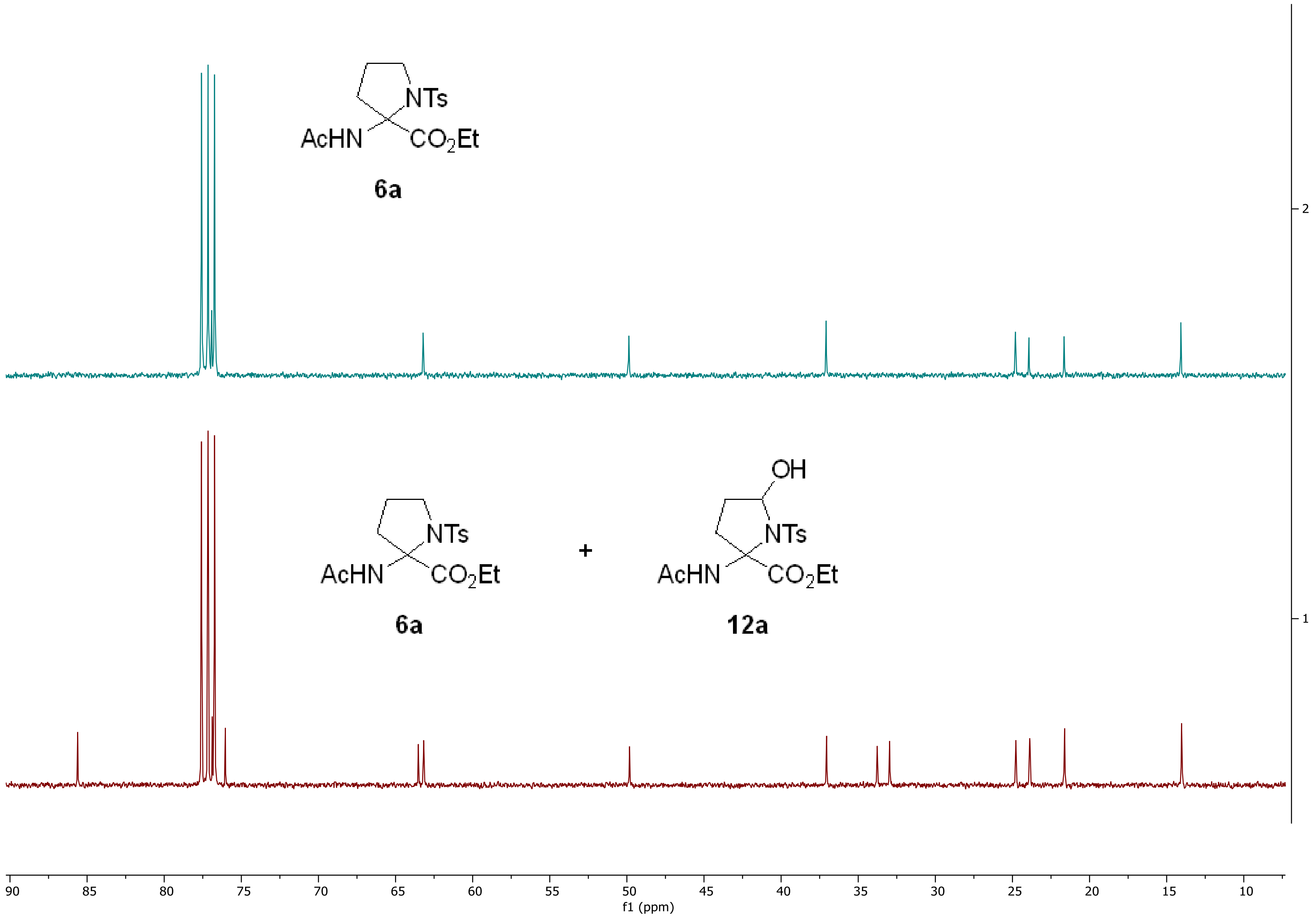
NTs
AcHN
CO_2Et
6a
OH
NTs
AcHN
CO_2Et
6a
+
12a
2
1
90
85
80
75
70
65
60
55
50
45
40
35
30
25
20
15
10
f1 (ppm)

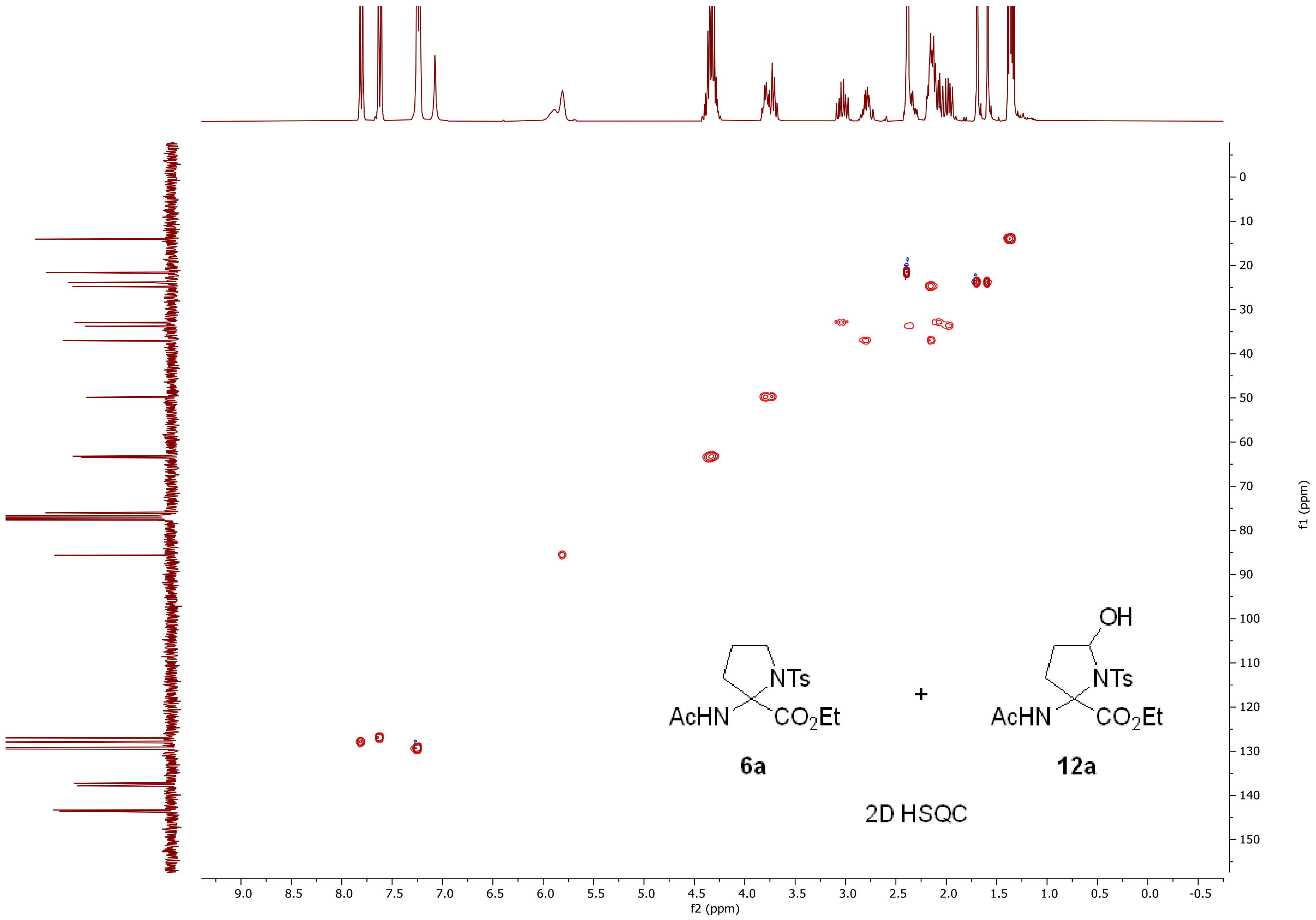
OH
NTs
AcHN
CO2Et
6a
+
12a
2D HSQC
f2 (ppm)
f1 (ppm)

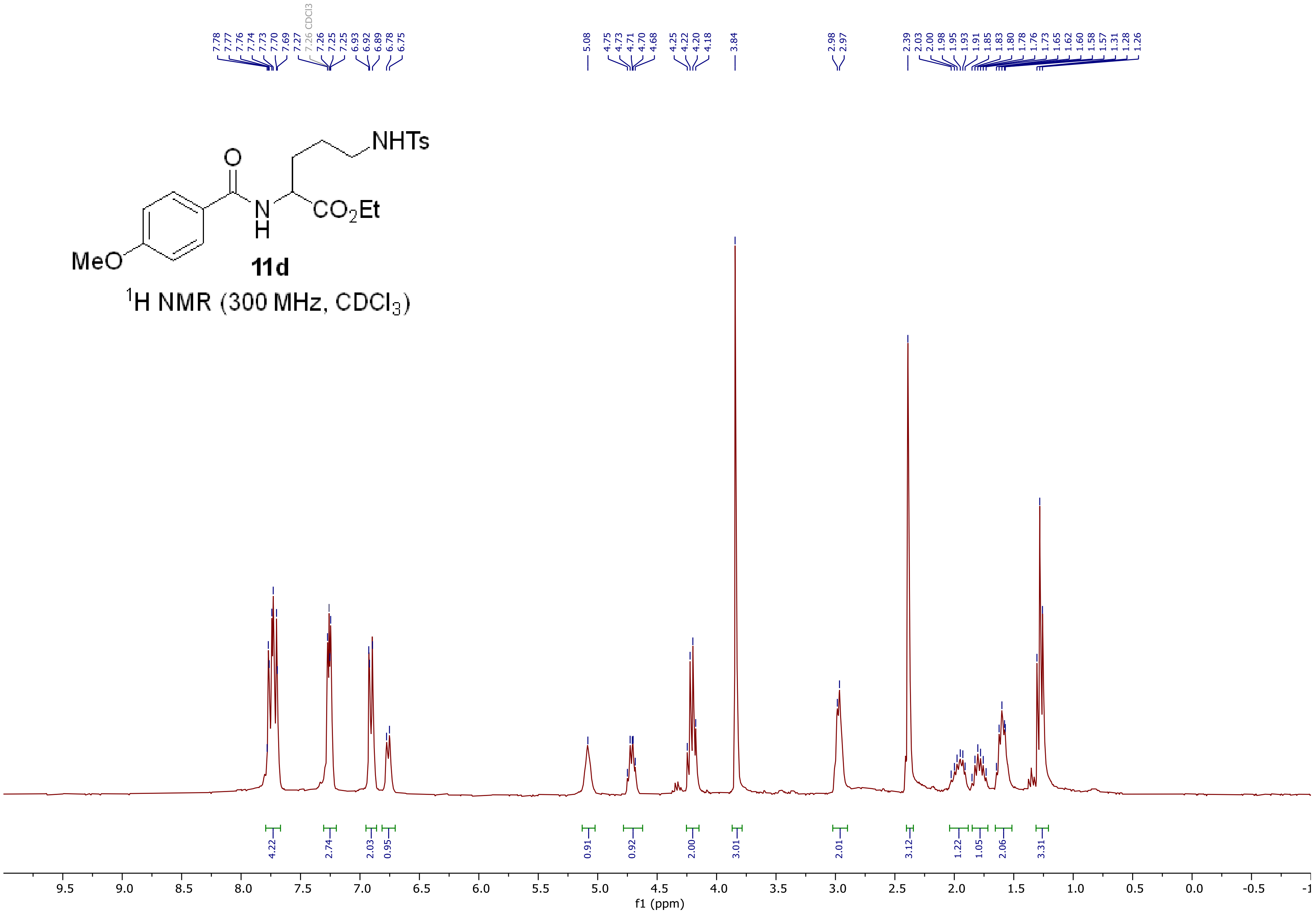

11d

^{1}H NMR (300 MHz, $CDCl_3$)

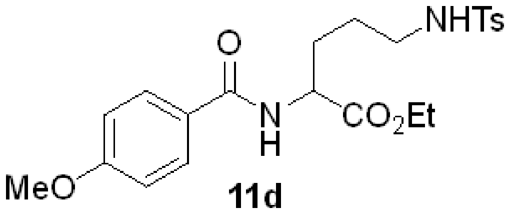

11d

^{13}C NMR (75 MHz, $CDCl_3$)

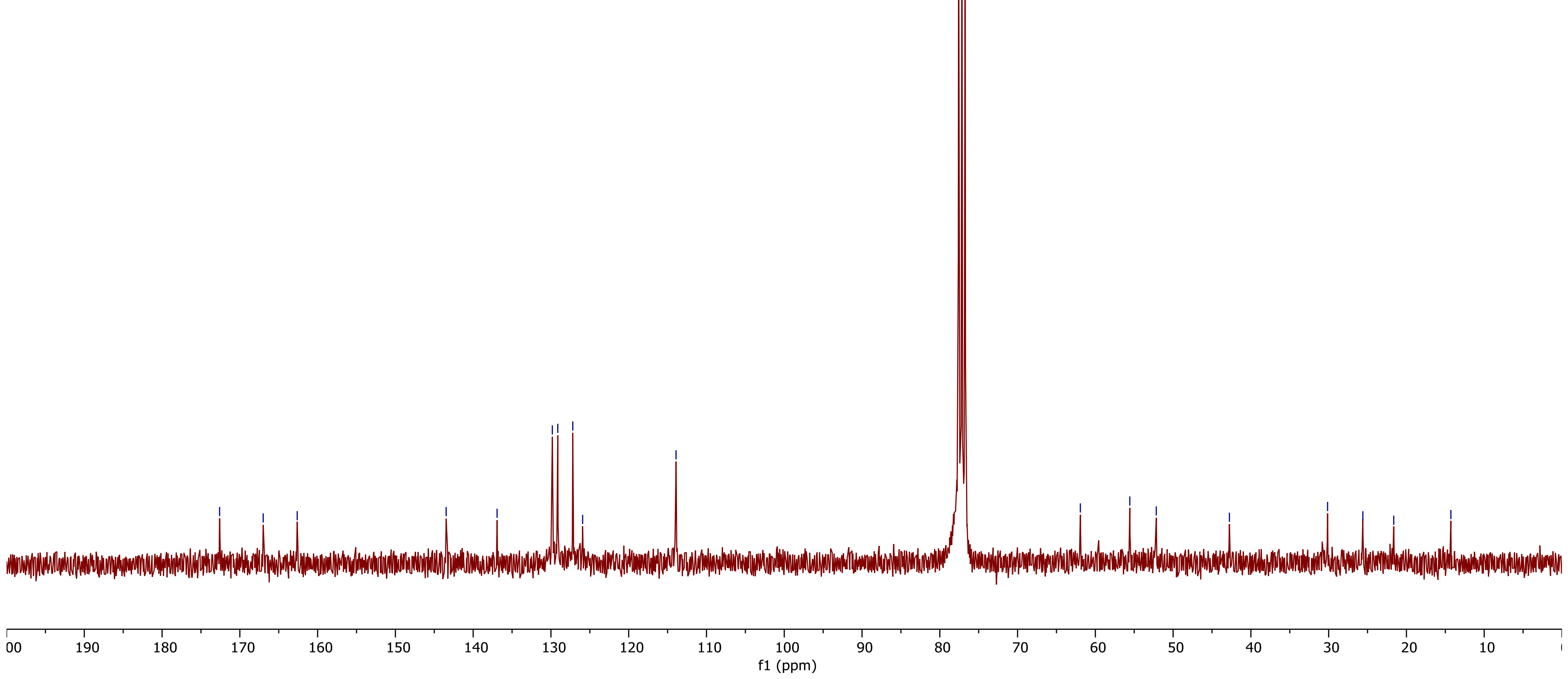

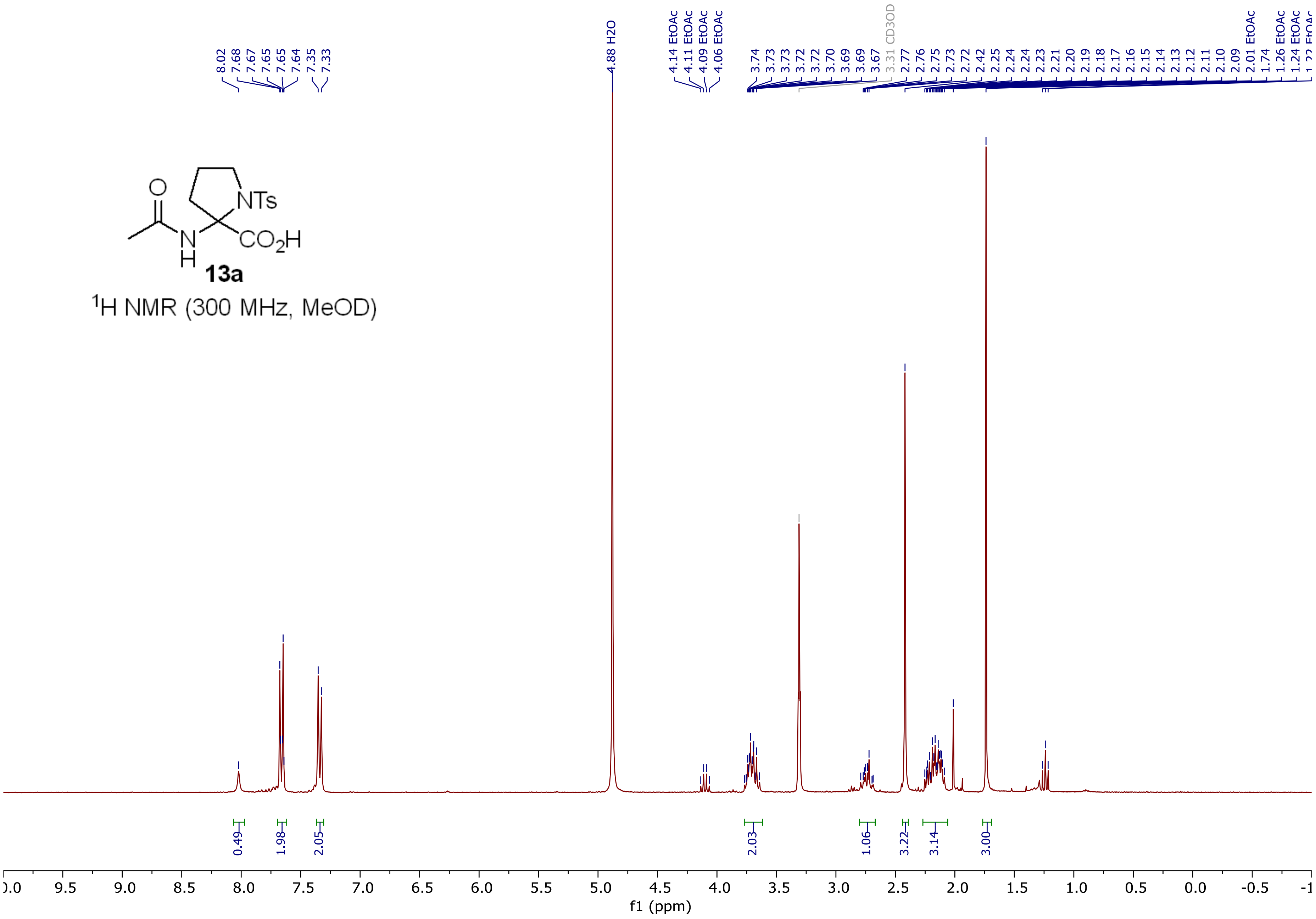
NTs
CO2H
N
H
O
13a
1H NMR (300 MHz, MeOD)
f1 (ppm)

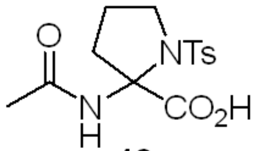

13a

^{13}C NMR (75 MHz, DMSO)

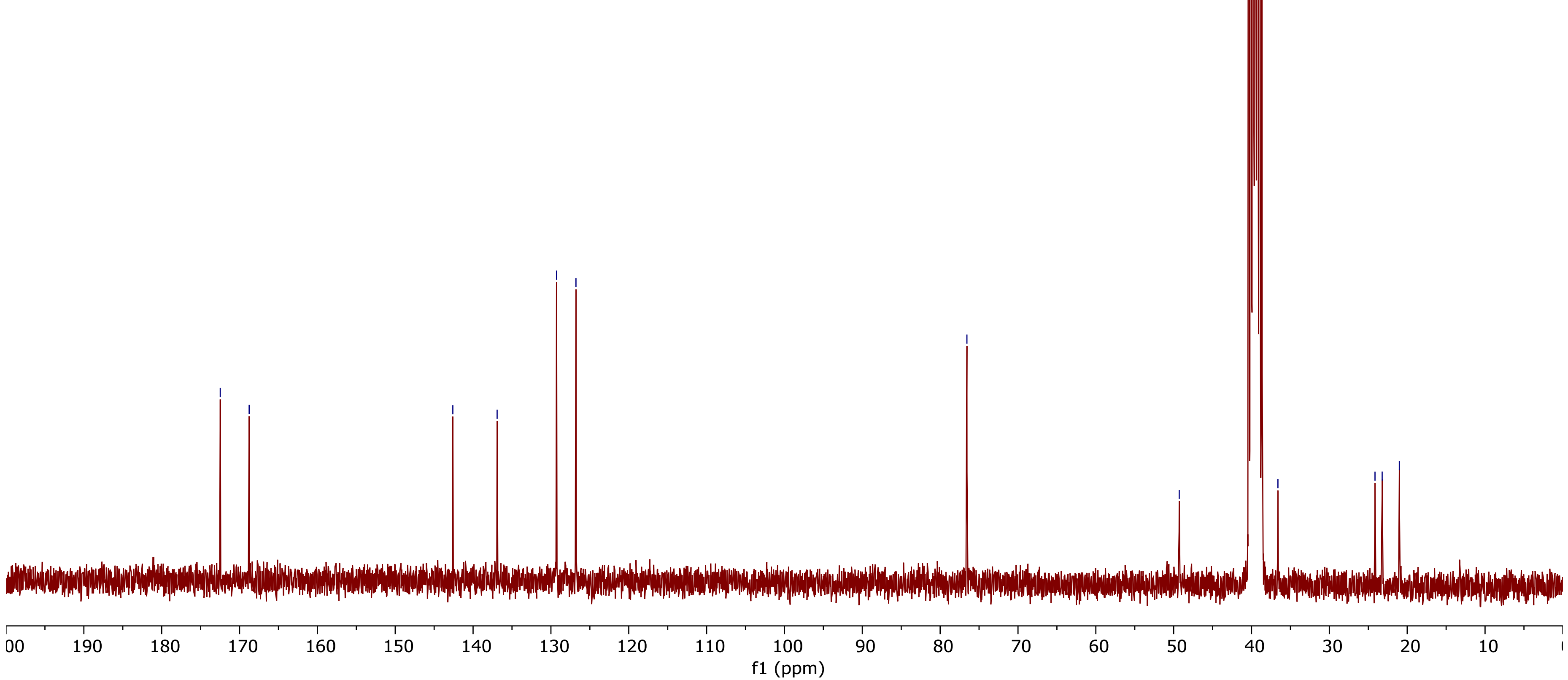

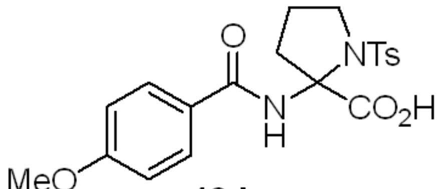

13d

^{1}H NMR (300 MHz, DMSO)

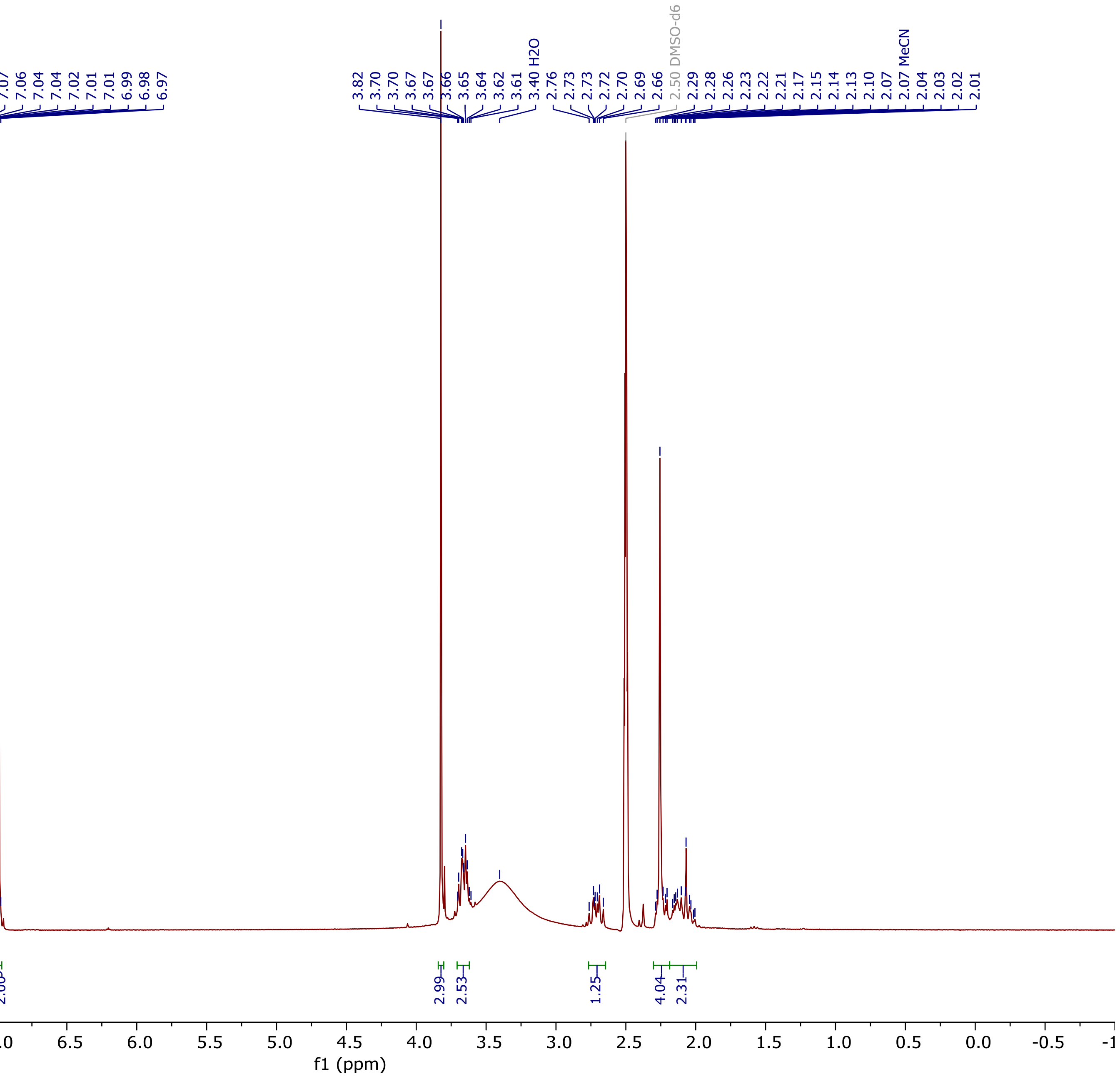

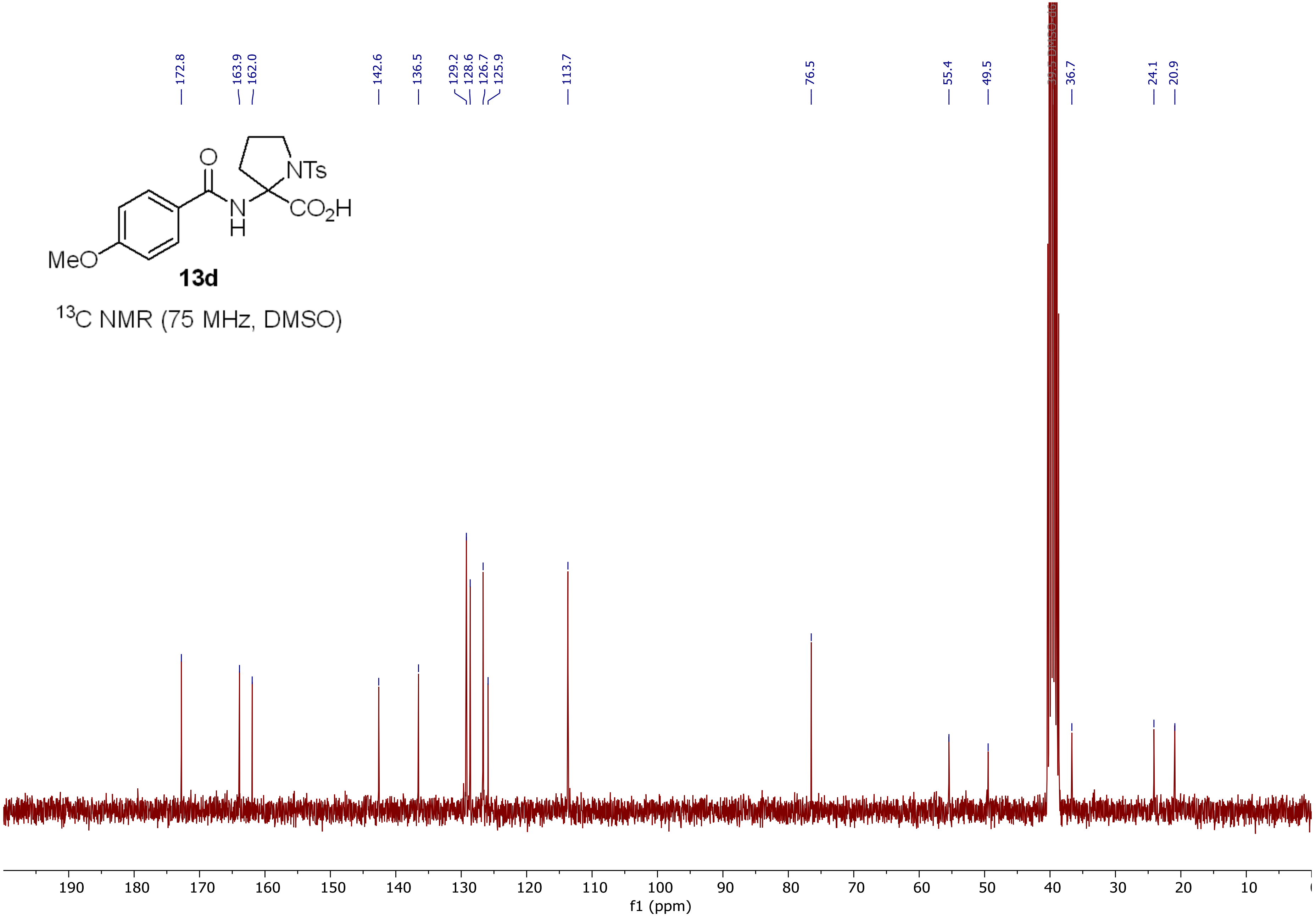
172.8
163.9
162.0
142.6
136.5
129.2
128.6
126.7
125.9
113.7
76.5
55.4
49.5
39.5 DMSO-d6
36.7
24.1
20.9
O
NTs
N
H
CO2H
MeO
13d
13C NMR (75 MHz, DMSO)
190 180 170 160 150 140 130 120 110 100 90 80 70 60 50 40 30 20 10
f1 (ppm)

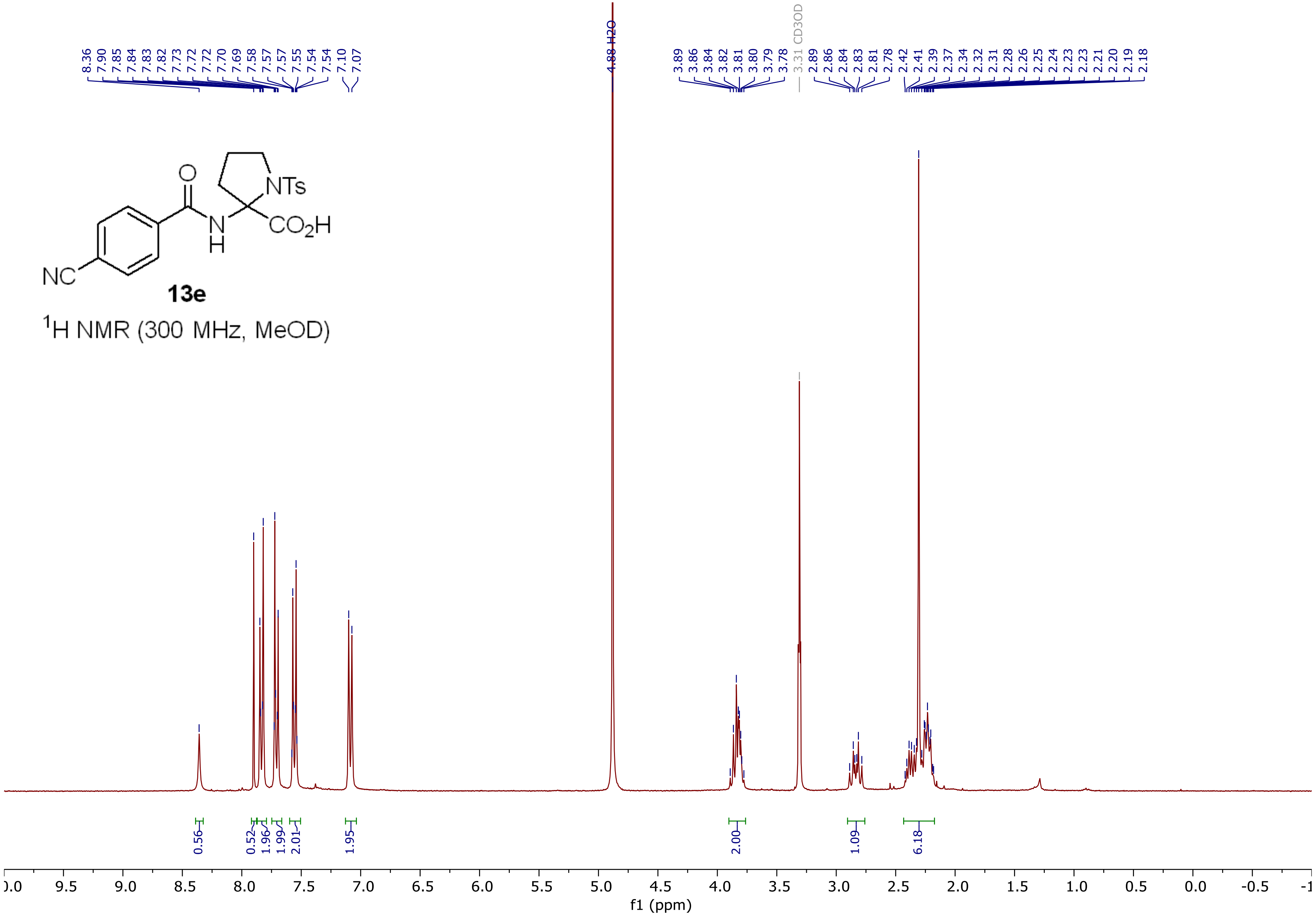
8.36
7.90
7.85
7.84
7.83
7.82
7.73
7.72
7.72
7.70
7.69
7.58
7.57
7.57
7.55
7.54
7.54
7.10
7.07
4.88 H2O
3.89
3.86
3.84
3.82
3.81
3.80
3.79
3.78
3.31 CD3OD
2.89
2.86
2.84
2.83
2.81
2.78
2.42
2.41
2.39
2.37
2.34
2.32
2.31
2.28
2.26
2.25
2.24
2.23
2.23
2.21
2.20
2.19
2.18
O
NTs
N
H
CO2H
NC
13e
1H NMR (300 MHz, MeOD)
0.56
0.52
1.96
1.99
2.01
1.95
2.00
1.09
6.18
9.5
9.0
8.5
8.0
7.5
7.0
6.5
6.0
5.5
5.0
4.5
4.0
3.5
3.0
2.5
2.0
1.5
1.0
0.5
0.0
-0.5
f1 (ppm)

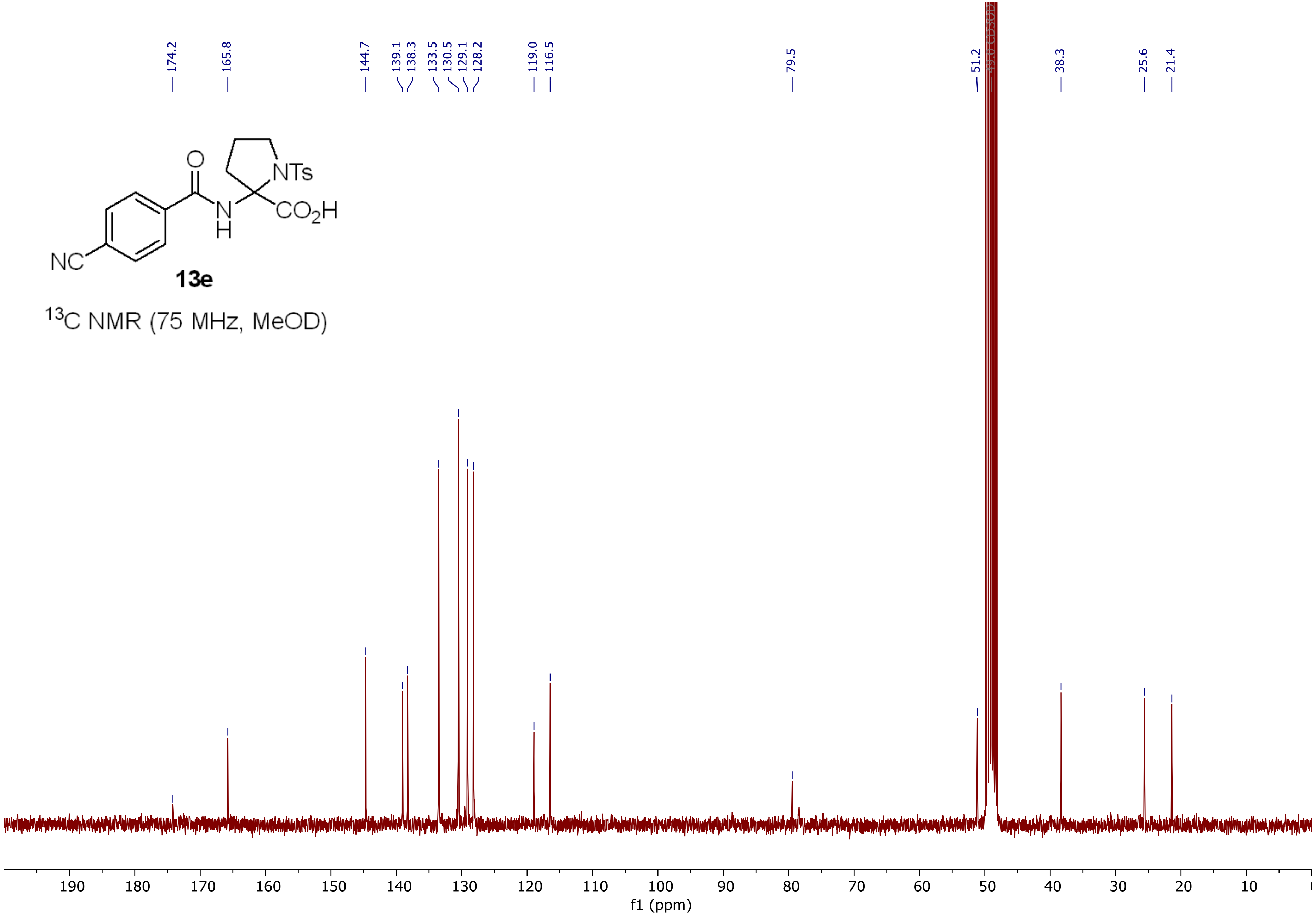
174.2
165.8
144.7
139.1
138.3
133.5
130.5
129.1
128.2
119.0
116.5
79.5
51.2
49.0 CD3OD
38.3
25.6
21.4
NTs
CO2H
N
H
O
NC
13e
13C NMR (75 MHz, MeOD)
190
180
170
160
150
140
130
120
110
100
90
80
70
60
50
40
30
20
10
f1 (ppm)

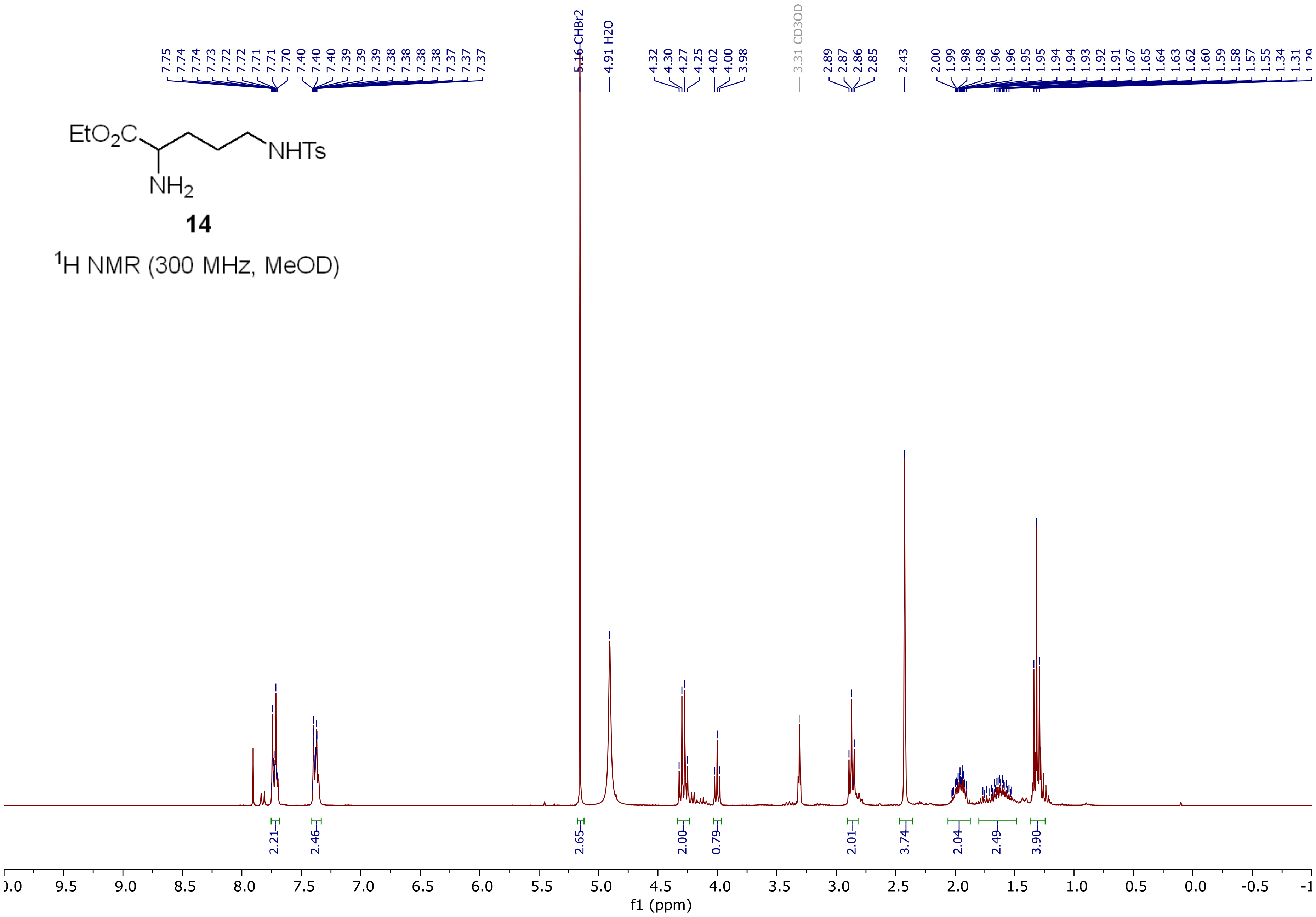
EtO2C
NHTs
NH2
14
1H NMR (300 MHz, MeOD)
7.75 7.74 7.74 7.73 7.72 7.72 7.71 7.71 7.70 7.40 7.40 7.39 7.39 7.39 7.38 7.38 7.38 7.38 7.37 7.37 7.37
5.16 CHBr2
4.91 H2O
4.32 4.30 4.27 4.25 4.02 4.00 3.98
3.31 CD3OD
2.89 2.87 2.86 2.85
2.43
2.00 1.99 1.98 1.98 1.96 1.96 1.95 1.95 1.94 1.94 1.93 1.92 1.91 1.67 1.65 1.64 1.63 1.62 1.60 1.59 1.58 1.57 1.55 1.34 1.31
2.21 2.46 2.65 2.00 0.79 2.01 3.74 2.04 2.49 3.90
9.5 9.0 8.5 8.0 7.5 7.0 6.5 6.0 5.5 5.0 4.5 4.0 3.5 3.0 2.5 2.0 1.5 1.0 0.5 0.0 -0.5
f1 (ppm)

14

^{13}C NMR (75 MHz, MeOD)

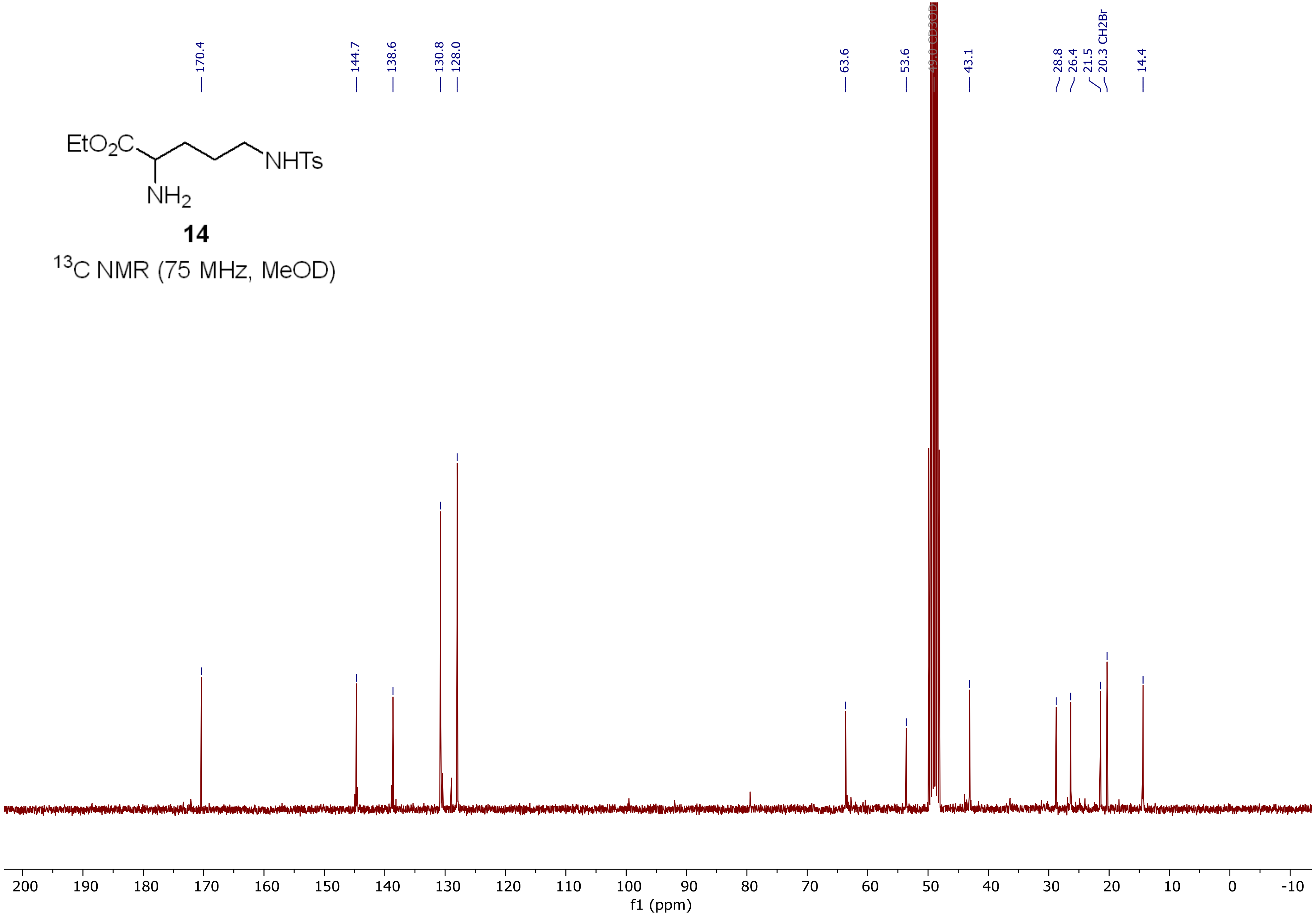

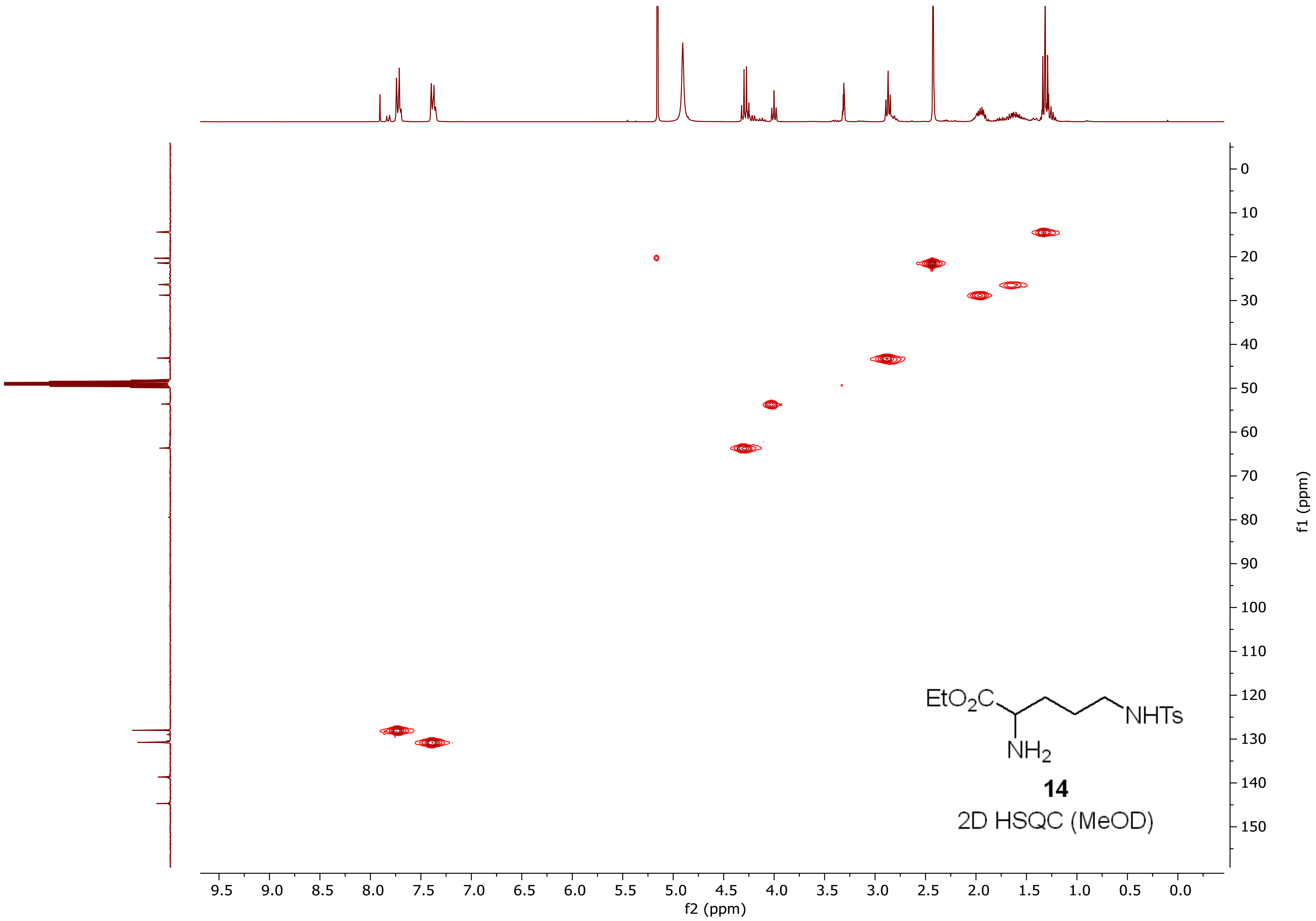

14

2D HSQC (MeOD)

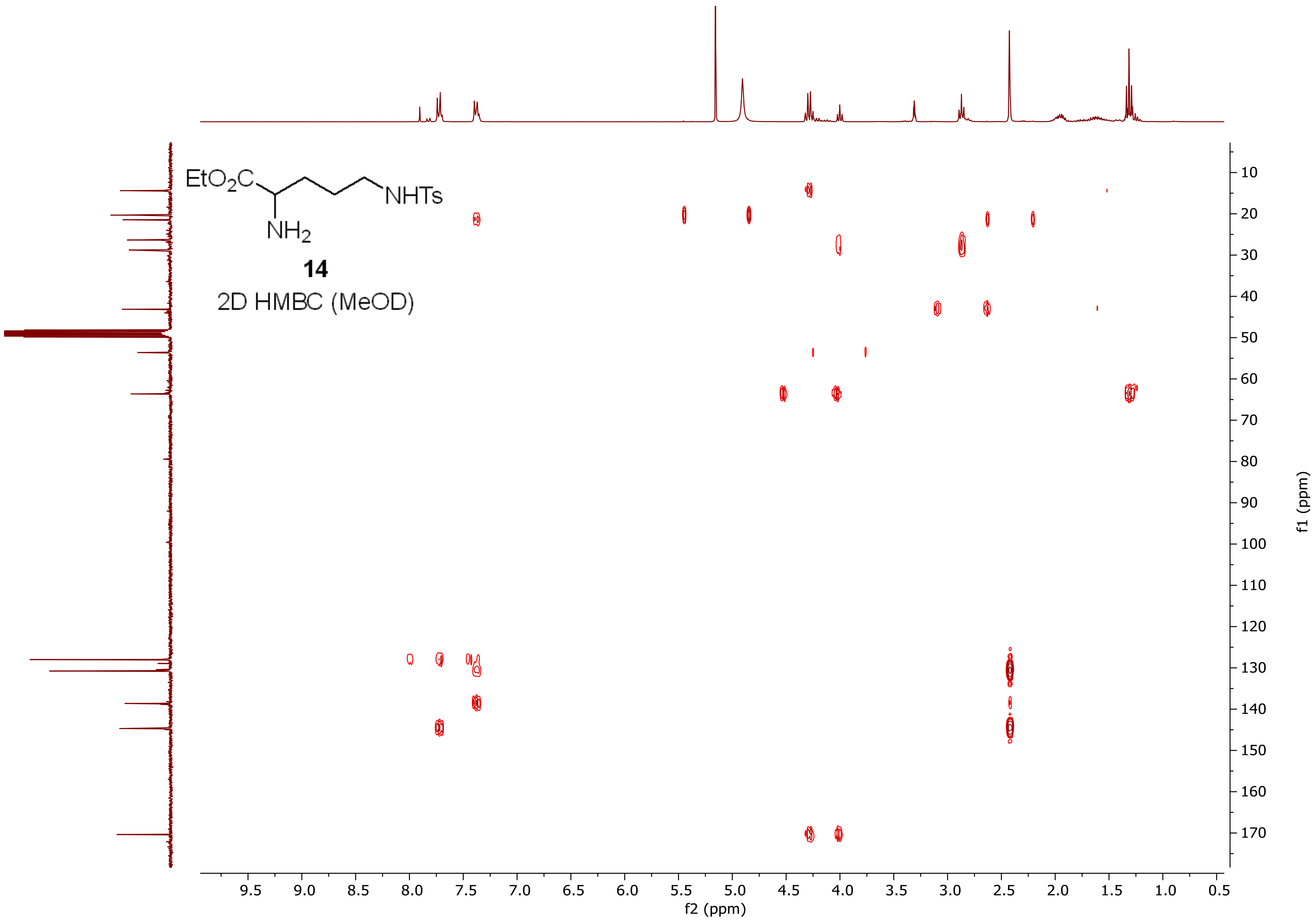
EtO2C
NH2
NHTs
14
2D HMBC (MeOD)
f2 (ppm)
f1 (ppm)
9.5 9.0 8.5 8.0 7.5 7.0 6.5 6.0 5.5 5.0 4.5 4.0 3.5 3.0 2.5 2.0 1.5 1.0 0.5
10 20 30 40 50 60 70 80 90 100 110 120 130 140 150 160 170

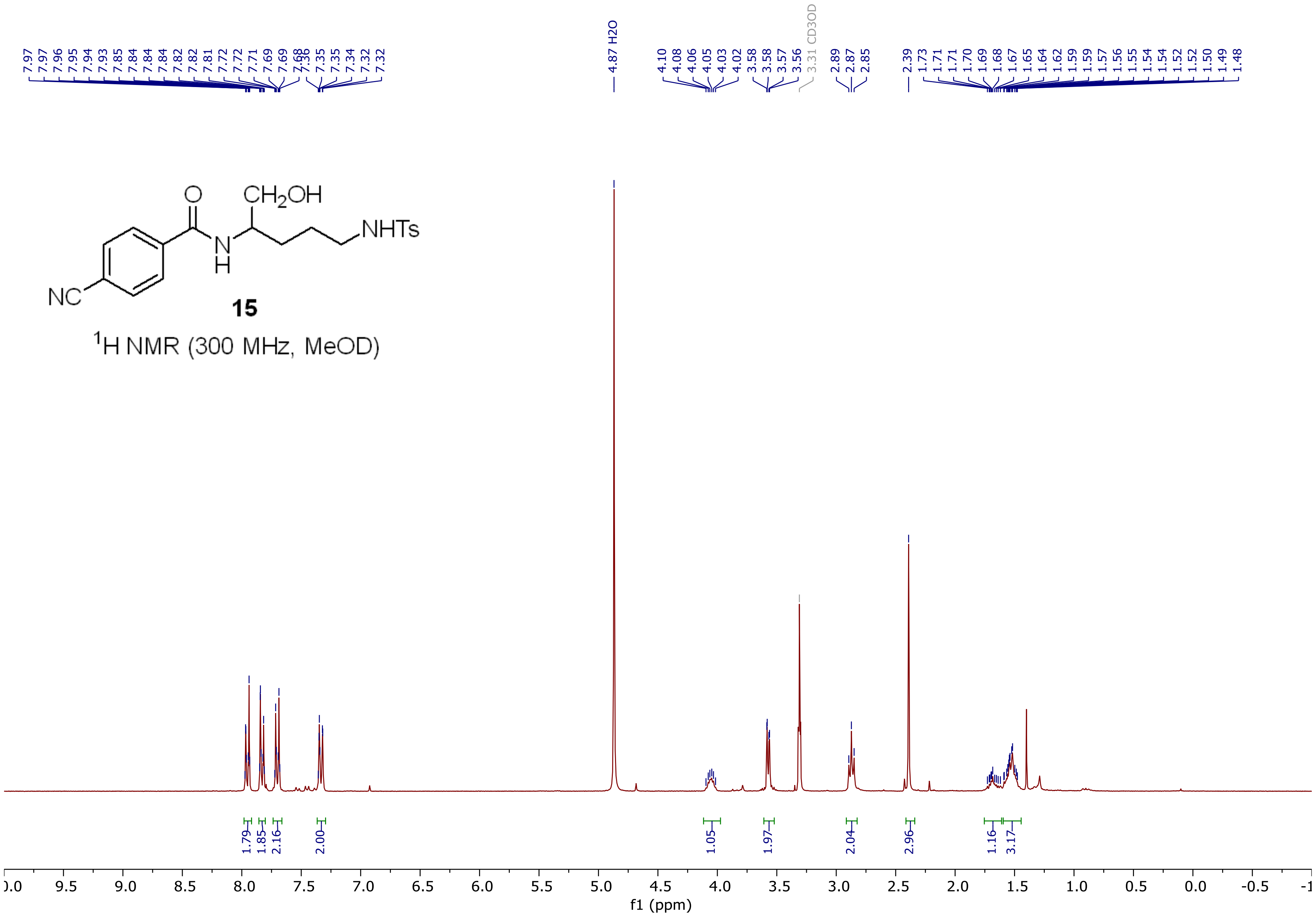

15

^{1}H NMR (300 MHz, MeOD)

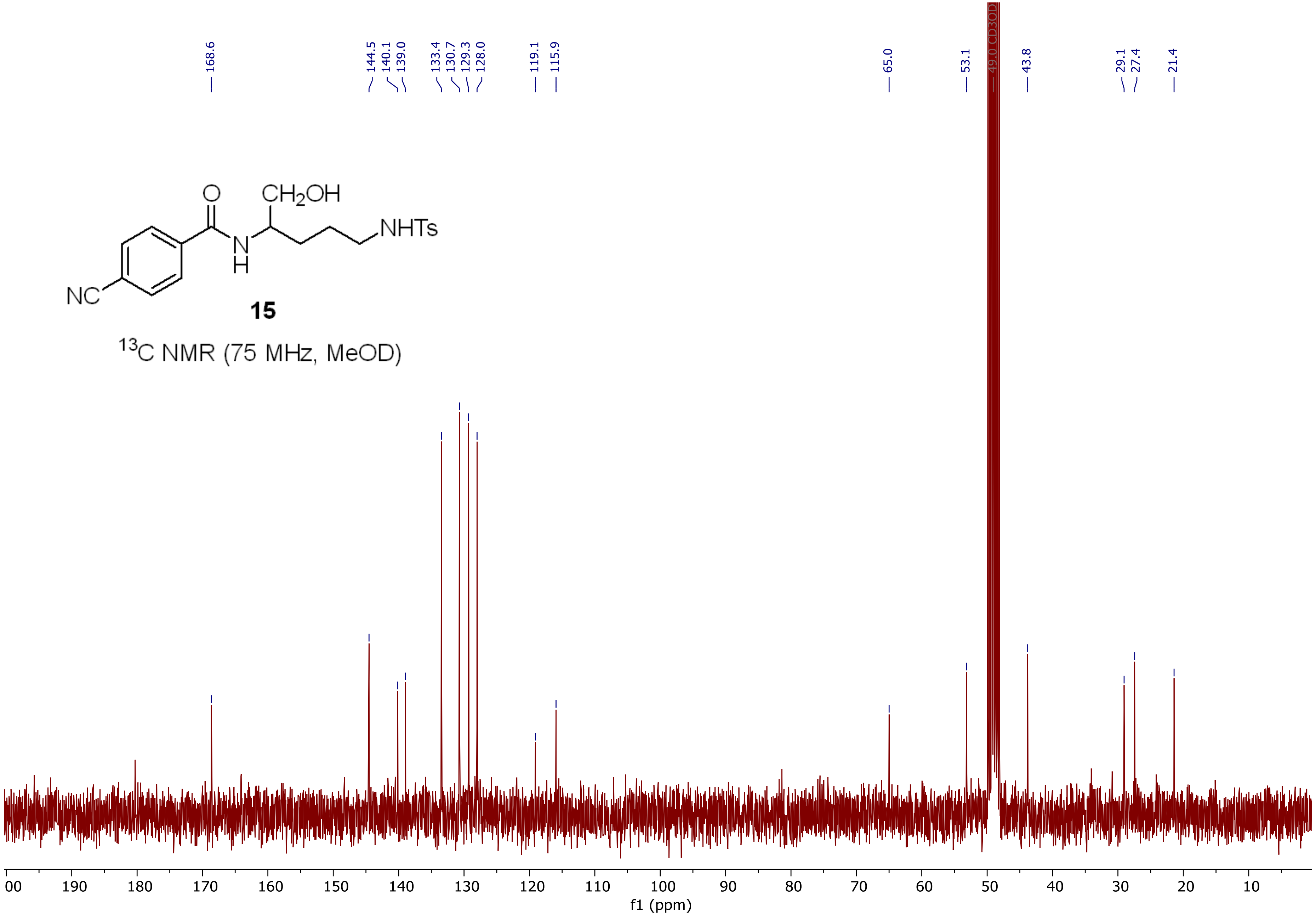

15

^{13}C NMR (75 MHz, MeOD)

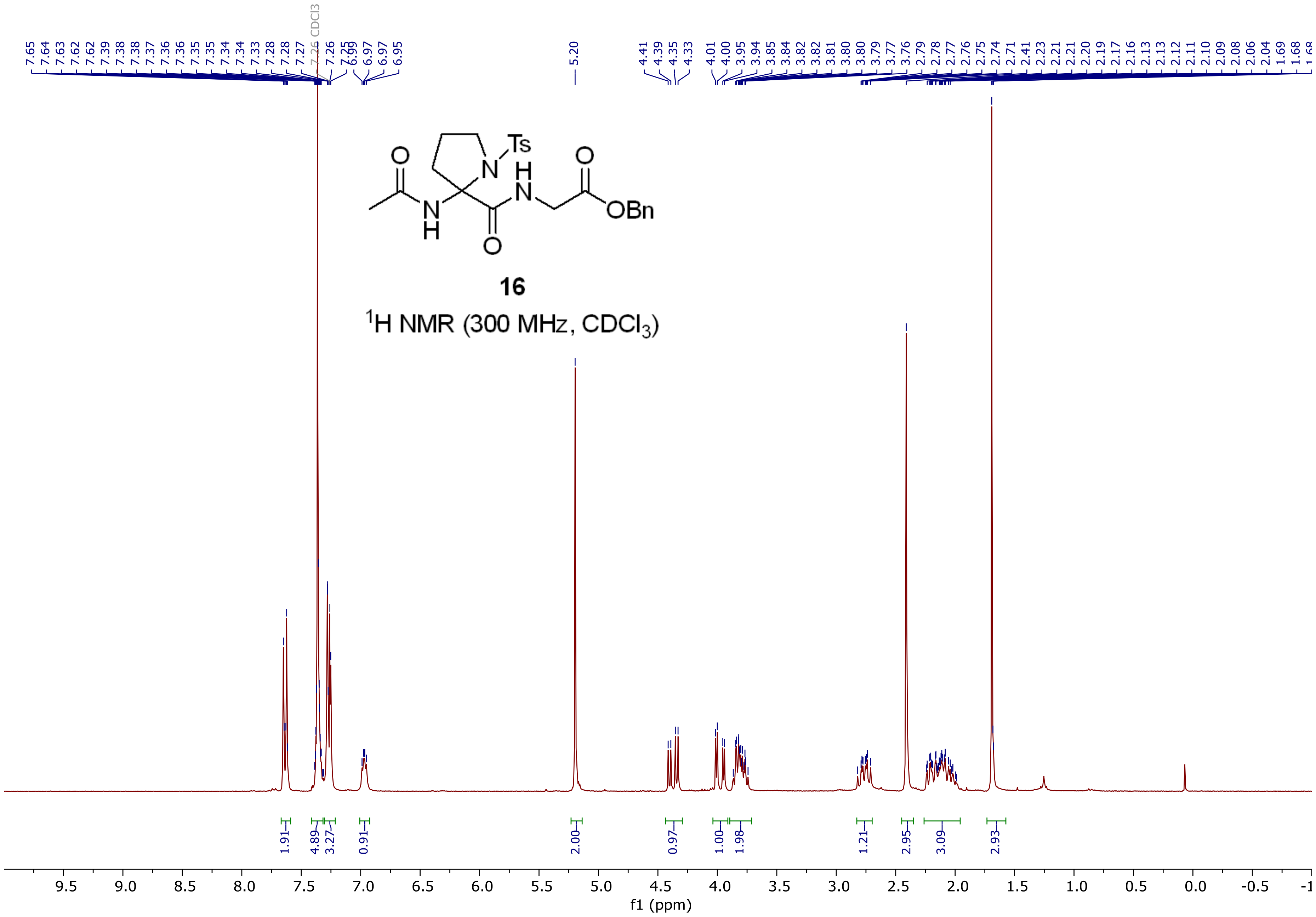
16
^{1}H NMR (300 MHz, CDCl$_3$)
f1 (ppm)

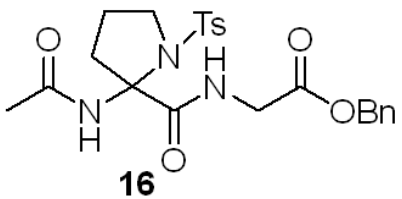

16

^{13}C NMR (75 MHz, $CDCl_3$)

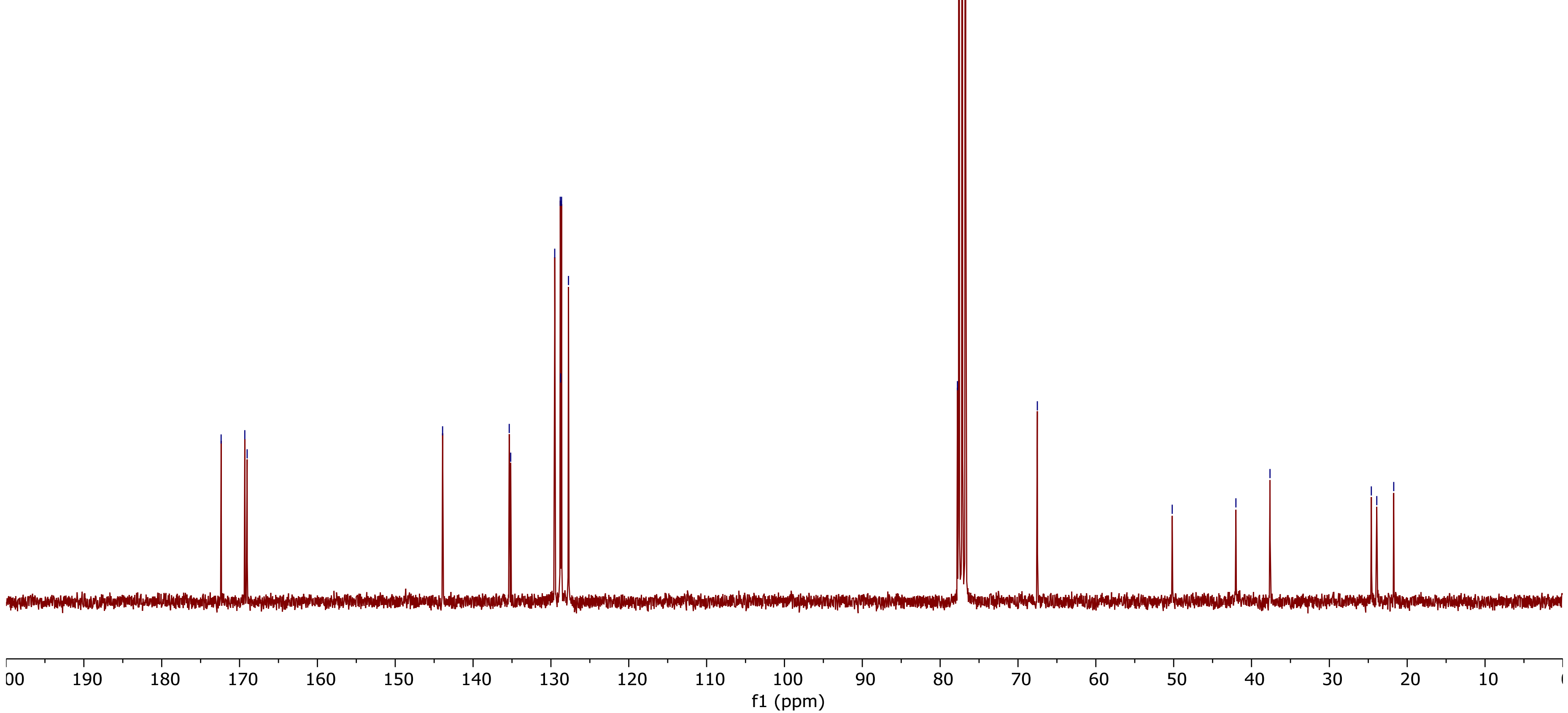

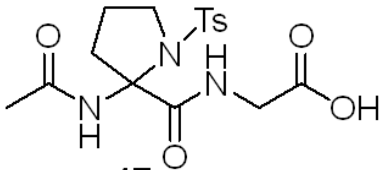

17

^{1}H NMR (300 MHz, MeOD-d_4)

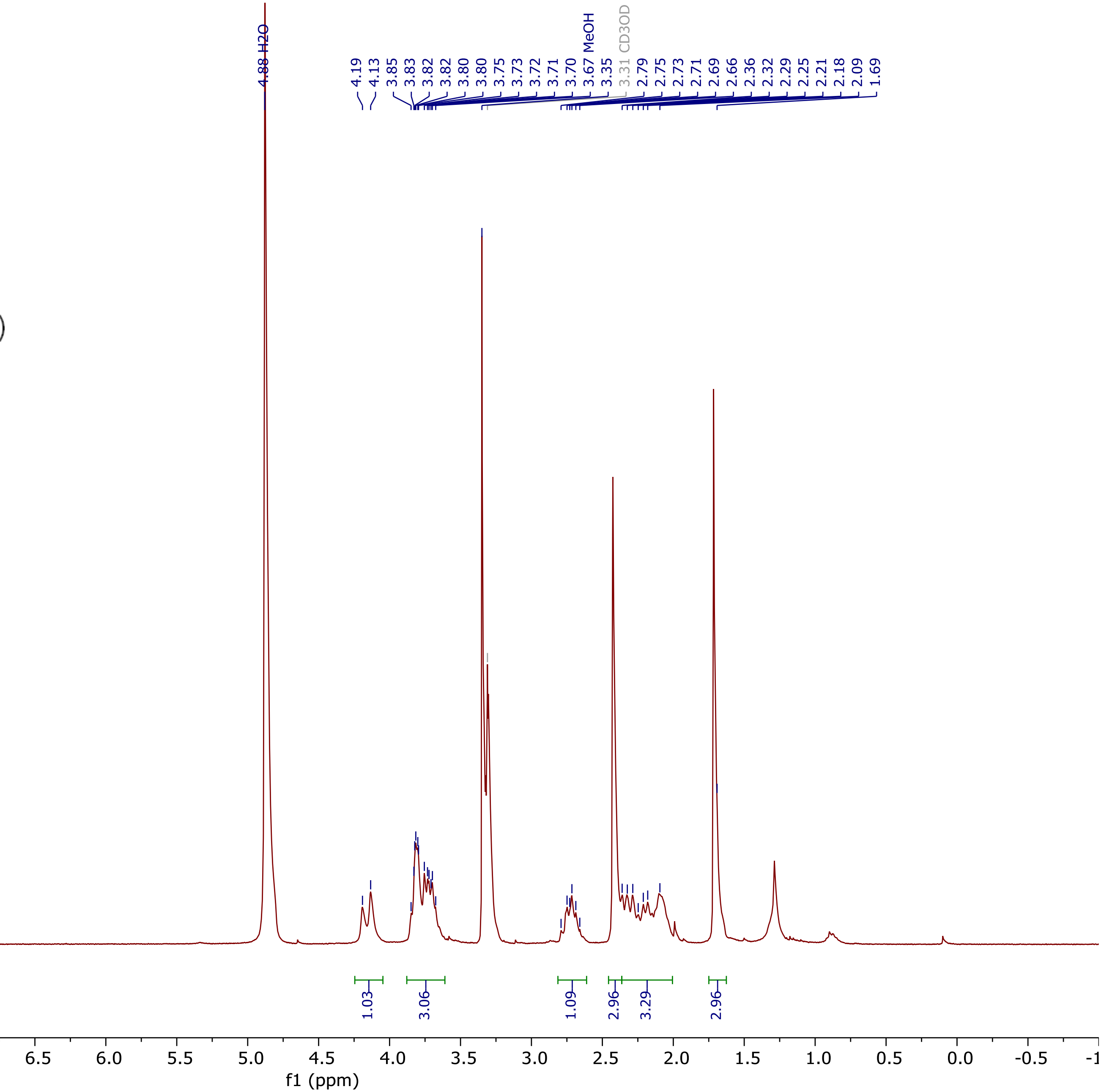

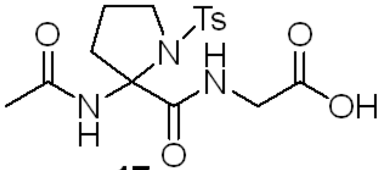

17

^{13}C NMR (75 MHz, MeOD-d_4)

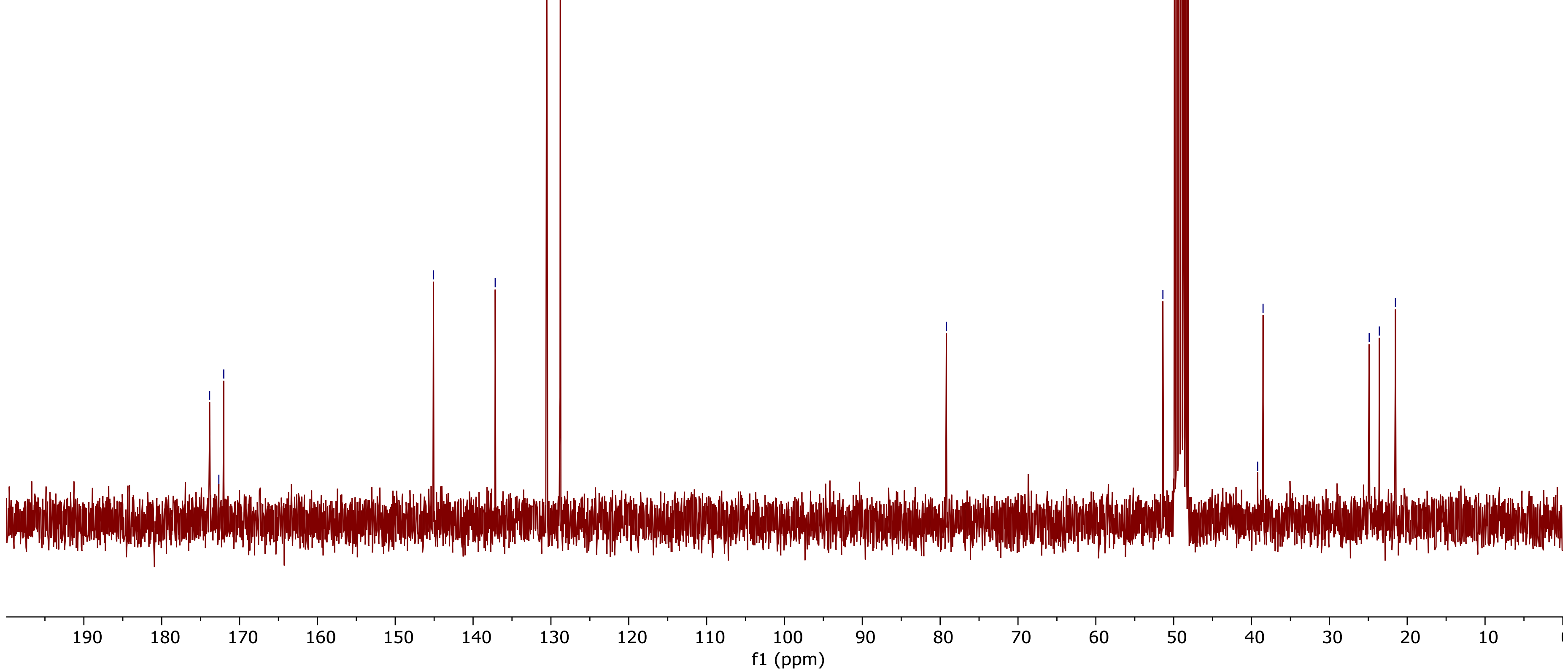

Supplement: File 1 — Detailed experimental procedures, analytical and spectroscopic data for the synthesized compounds, and copies of NMR spectra. [file Beilstein_J_Org_Chem-21-630-s001.pdf]
